# A user‐friendly guide to using distance measures to compare time series in ecology

**DOI:** 10.1002/ece3.10520

**Published:** 2023-10-05

**Authors:** Shawn Dove, Monika Böhm, Robin Freeman, Sean Jellesmark, David J. Murrell

**Affiliations:** ^1^ Centre for Biodiversity and Environment Research University College London London UK; ^2^ Institute of Zoology, Zoological Society of London London UK; ^3^ Global Center for Species Survival, Indianapolis Zoo Indianapolis Indiana USA

**Keywords:** classification, clustering, dissimilarity measures, distance measure selection, time series analysis, time series comparison

## Abstract

Time series are a critical component of ecological analysis, used to track changes in biotic and abiotic variables. Information can be extracted from the properties of time series for tasks such as classification (e.g., assigning species to individual bird calls); clustering (e.g., clustering similar responses in population dynamics to abrupt changes in the environment or management interventions); prediction (e.g., accuracy of model predictions to original time series data); and anomaly detection (e.g., detecting possible catastrophic events from population time series). These common tasks in ecological research all rely on the notion of (dis‐) similarity, which can be determined using distance measures. A plethora of distance measures have been described, predominantly in the computer and information sciences, but many have not been introduced to ecologists. Furthermore, little is known about how to select appropriate distance measures for time‐series‐related tasks. Therefore, many potential applications remain unexplored. Here, we describe 16 properties of distance measures that are likely to be of importance to a variety of ecological questions involving time series. We then test 42 distance measures for each property and use the results to develop an objective method to select appropriate distance measures for any task and ecological dataset. We demonstrate our selection method by applying it to a set of real‐world data on breeding bird populations in the UK and discuss other potential applications for distance measures, along with associated technical issues common in ecology. Our real‐world population trends exhibit a common challenge for time series comparisons: a high level of stochasticity. We demonstrate two different ways of overcoming this challenge, first by selecting distance measures with properties that make them well suited to comparing noisy time series and second by applying a smoothing algorithm before selecting appropriate distance measures. In both cases, the distance measures chosen through our selection method are not only fit‐for‐purpose but are consistent in their rankings of the population trends. The results of our study should lead to an improved understanding of, and greater scope for, the use of distance measures for comparing ecological time series and help us answer new ecological questions.

## Graphical abstract

Distance measures are often used in ecology to perform common time‐series‐related tasks such as classification, clustering, prediction, and anomaly detection, but little is known about how to select appropriate distance measures for specific tasks. We present a selection method for choosing appropriate distance measures, then demonstrate the method on a real‐world dataset and discuss common challenges and ways of overcoming them. The results of our study should lead to an improved understanding of, and greater scope for, the use of distance measures for comparing ecological time series, and help answer new ecological questions.

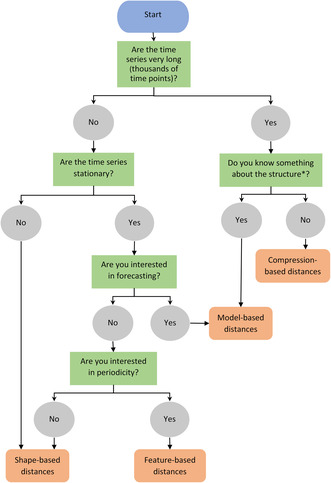

## 1 INTRODUCTION

Time series are a critical component of ecological analysis: Ecologists use time series to track changes in biotic variables, such as population sizes and mean growth rates of individuals, as well as abiotic variables, such as temperature and atmospheric carbon dioxide. Time series provide insights into food web and ecosystem function and the causes and effects of environmental change, and are vital to any scientific approach to environmental management (Boero et al., 2015). Ecologists can make inferences through time series comparisons, for example, looking for similarities (differences) in climate change response between populations within or across geographic or taxonomic groups. However, examining and analyzing each time series by hand is often unwieldy because comparisons may be across thousands or even millions of time series (e.g., BioTIME—Dornelas et al., 2018; the Continuous Plankton Recorder Survey—Edwards et al., 2016; the British Trust for Ornithology Breeding Bird Survey—Harris et al., 2020; the North American Breeding Bird Survey—Pardieck et al., 2020; and The Living Planet Index—WWF, 2020).

Data mining of time series is the process of extracting information from the properties of time series for tasks such as classification, clustering, prediction, and anomaly detection (Esling & Agon, 2012). These tasks are common in ecology, for example, clustering time series of parasite counts to identify infection patterns (Marques et al., 2018); predicting the emergence of fruiting bodies by classifying time series of environmental drivers (Capinha, 2019); identifying insect species by classifying wingbeat frequency signals (Potamitis et al., 2015); surveying bird population sizes by classifying recorded calls (Priyadarshani et al., 2020); and predicting species distributions based on time series of environmental variables (Capinha et al., 2020). These tasks all rely on the notion of (dis‐) similarity. For example, clustering involves grouping similar time series together by maximizing the similarity within groups and minimizing the similarity between groups (Aghabozorgi et al., 2015; Esling & Agon, 2012; Liao, 2005). Classification is like clustering, except labels are predefined and new time series are assigned to existing clusters to which they are most similar (Keogh & Kasetti, 2003). For example, time series for individual song/call could be classified into known species. Prediction may rely on similarity to determine accuracy of a predictive model by comparing output time series against the original data (Capinha, 2019; Esling & Agon, 2012). Finally, anomaly detection involves comparing time series against an anomaly‐free model to determine whether they fall outside of a similarity threshold (Esling & Agon, 2012; Teng, 2010).

Similarity between time series can be determined by using distance measures to measure its inverse: dissimilarity. Dissimilarity is more intuitive as a measurement because a value of zero occurs when two time series are identical (while similarity has a scale‐dependent maximum value). Applications for distance measures typically fall into the four categories defined above. However, there are other less well‐known applications, such as content queries, hypothesis testing, accuracy assessment, and comparison of time series models (i.e., using comparison methods on model outputs to aid model selection). Distance measures can also be used for pattern matching against databases to identify animal species or biological or ecological events from recorded or streaming data sources, such as video, audio, photographs, motion capture, temperature monitors, or other types of sensors. In addition, there are many other types of time series that one might wish to compare, such as activity patterns, biomass, nutrient uptake, growth rates, and entropy.

The choice of distance measure for any task should depend on the properties of the data to be analyzed and the nature of the task (Esling & Agon, 2012). In practice, choosing a distance measure often becomes a matter of convenience. For example, the well‐known and easy‐to‐use Euclidean distance is among the most widely used distance measures, although there are often better choices (Paparrizos et al., 2020; Wang et al., 2013). When investigating the performance of five distance measures for comparing animal movement trajectories, Cleasby et al. (2019) found that the most used measure was the least appropriate choice. One problem for ecologists is that many distance measures originate within computer science, information science, systems science, and mathematics, and few are currently in common use within ecology. Another problem is that information on the strengths, weaknesses, and appropriate uses of distance measures is limited and often difficult to find. Some reviews of distance measures have been published (Esling & Agon, 2012; Lhermitte et al., 2011; Liao, 2005; Montero & Vilar, 2014; Mori et al., 2016a), but are not generally aimed at ecologists (but see Lhermitte et al., 2011); analysis of the properties of distance measures is limited, and guidance on how to choose an appropriate distance measure is either missing or very general and not within the context of ecological problems. Other studies have analyzed the classification accuracy of multiple distance measures across a variety of datasets (Bagnall et al., 2017; Paparrizos et al., 2020; Pree et al., 2014; Wang et al., 2013), but pooled the results to give overall performance scores. This ignores the fact that different distance measures perform better on different datasets and for different tasks. Kocher and Savoy (2017) tested 24 distance measures for six properties and then compared their effectiveness in classification on 13 real‐world datasets. However, the study focused on a single task (author profiling, i.e., determining demographic information about the author of a document based on the document itself), and did not present a general method for selecting distance measures for other tasks. Furthermore, the distance measures that demonstrated all proposed properties did not perform best on real‐world datasets. Mori et al. (2016b) developed an automated process for selecting distance measures based on nine quantifiable properties of datasets. However, their classifier is limited to clustering tasks, and only includes five common distance measures. We are not aware of any more generalized method of distance measure selection.

In this study, we present a generalized, objective, user‐driven method of choosing fit‐for‐purpose distance measures for time series comparison tasks. We evaluate 42 distance measures for 16 properties related to time series comparison, and use the results in combination with existing literature to develop our selection method. We then demonstrate the method by applying it to a set of real‐world UK bird population trends from a study of the effectiveness of conservation measures (Jellesmark et al., 2021). Finally, we discuss potential applications for using distance measures to compare time series and describe how to use our selection method to choose an appropriate distance measure for any time series dataset and task.

## 2 METHODS

Distance measures can be broadly categorized into four different types: (1) shape‐based, (2) feature‐based, (3) model‐based, and (4) compression‐based. Shape‐based distances compare the shapes of time series by measuring differences in the raw data values (Aghabozorgi et al., 2015; Esling & Agon, 2012) and can be further divided into lock‐step measures and elastic measures. Lock‐step measures compare each time point of one time series to the corresponding time point of another time series, while elastic measures allow a single point to be matched with multiple points or no points (Wang et al., 2013). Elastic measures fall into two groups. The first, dynamic time warping (DTW), computes an optimal match between two time series by allowing single points to be matched with multiple points, thus allowing local distortion or “warping” of the time dimension (Esling & Agon, 2012). The second comprises edit distances, which compare the minimum number of “edits,” or changes, required to transform one time series into another (Esling & Agon, 2012). These are based on the concept of transforming one string into another by changing one letter at a time, with each “edit” being an insertion, deletion, or substitution. Feature‐based distances compute some feature of time series, such as discrete Fourier transforms or autocorrelation coefficients, and use either a specialized or common distance function (e.g., the Euclidean distance) to determine the distance between the computed features (Mori et al., 2016a). Model‐based distances compare the parameters of models fitted to the time series, such as autoregressive moving average (ARMA) models, with the advantage that they can incorporate knowledge about the process used to generate the time series data (Esling & Agon, 2012). Finally, compression‐based distances assess the similarity of two digital objects according to how well they can be “compressed” when connected (Cilibrasi & Vitanyi, 2005; Esling & Agon, 2012); the more similar the objects, the better they compress when joined in series (Esling & Agon, 2012). Although there are comparatively few model‐based and compression‐based distance measures, there are many shape‐based and feature‐based measures available.

We selected 42 distance measures from the literature (see Table A1 in Appendix A), choosing measures that had already been implemented in publicly accessible R packages, and that represented each of the types of measures defined above, as well as a variety of potential use cases. Of these, 18 are implemented in the R package “TSclust” (version 1.3.1) and have been studied for use in clustering time series (Montero & Vilar, 2014), and the remaining 24 are implemented in the R package “philentropy” (version 0.5.0; Drost, 2018).

We defined a set of 16 properties of distance measures that may be of interest in time series comparison in ecological problems: four metric properties, six value‐based properties, five time‐based properties, and one uncategorized property. Metric properties define whether dissimilarity is measured in metric space (a space that has real physical meaning). Distance measures that do not demonstrate all the metric properties (semi‐metrics and non‐metrics; McCune & Grace, 2002) are useful, but less intuitive (e.g., negative distances, or non‐zero distances between identical objects). Value‐based properties focus on dissimilarities on the *y*‐axis (differences in values), while time‐based properties focus on dissimilarities on the *x*‐axis (differences in time).

### 2.1 Metric properties (adapted from McCune & Grace, 2002)

- M1: Reflexivity: *d*(*X*,*X*) = 0. The dissimilarity value between a time series *X* and itself is zero.
- M2: Symmetry: *d*(*X*,*Y*) = *d*(*Y*,*X*). The dissimilarity value is the same regardless of the order in which time series are compared, *X* to *Y* or *Y* to *X*. A distance measure without symmetry might cluster a collection of time series differently depending on how the time series are ordered. In the real world, distances within city road networks are often non‐symmetric due to one‐way streets. Animal migration times might be non‐symmetric if they are moving uphill in one direction and downhill in the other.
- M3: Triangle inequality: *d*(*X*,*Z*) + *d*(*Z*,*Y*) ≥ *d*(*X*,*Y*). Given three time series, the distance between any pair of them is never larger than the sum of the distances between the other two pairs of time series. This property is related to Euclidean geometry (one side of a triangle cannot be longer than the other two combined). A non‐metric or semi‐metric that does not satisfy the triangle inequality can cause errors for many clustering algorithms (Jacobs et al., 2000). On the contrary, some time series classification problems require a distance measure that does *not* satisfy the triangle inequality, for example, when it is important to ignore outliers or whole subsets of observations (Weinshall et al., 1998). Matching many points to a single point, which allows for warping invariance (T3 below) would not be possible with a metric distance. Therefore, comparing animal calls or movement patterns or other time series that may have a similar pattern but with one time series stretched relative to the other may require a semi‐metric (e.g., DTW) or non‐metric for accurate classification.
- M4: Non‐negativity: *d*(*X*,*Y*) ≥ 0. The dissimilarity value is never less than zero. Mathematically, this must be true if properties M1, M2, and M3 are true. However, some distance measures that do not satisfy the triangle inequality can return negative dissimilarity values.

### 2.2 Value‐based properties

**FIGURE 1:**
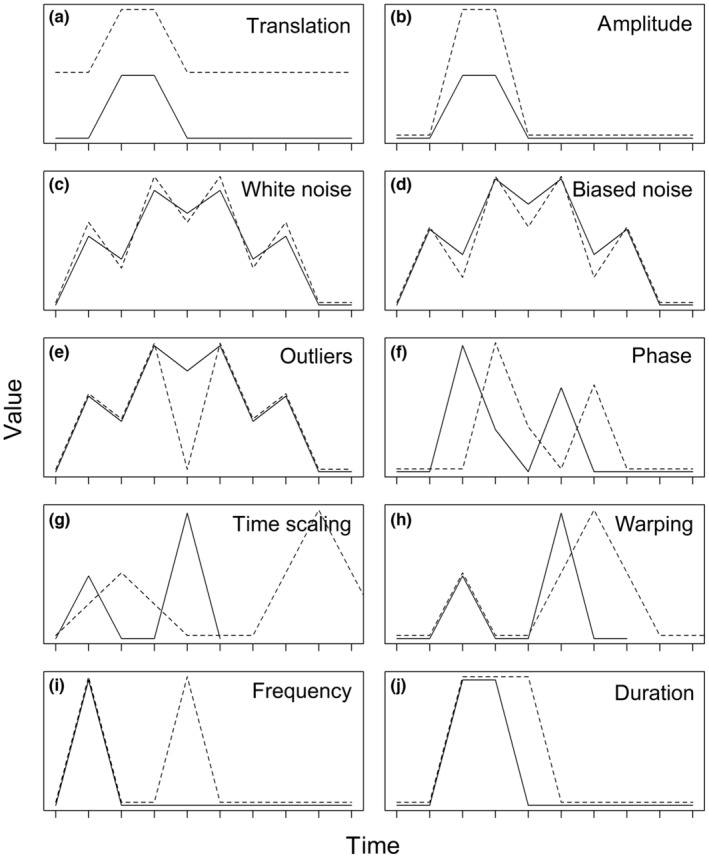
Illustration of time series distortions used to demonstrate sensitivities or invariances of distance measures to: (a) translation; (b) amplitude; (c) white noise; (d) biased noise; (e) outliers; (f) phase; (g) time scaling; (h) warping; (i) frequency; and (j) duration. A dissimilarity value of zero (or equivalent, for any distance measure not demonstrating reflexivity) between any of the illustrated pairs of time series would indicate an invariance to that type of distortion.

**FIGURE 2:**
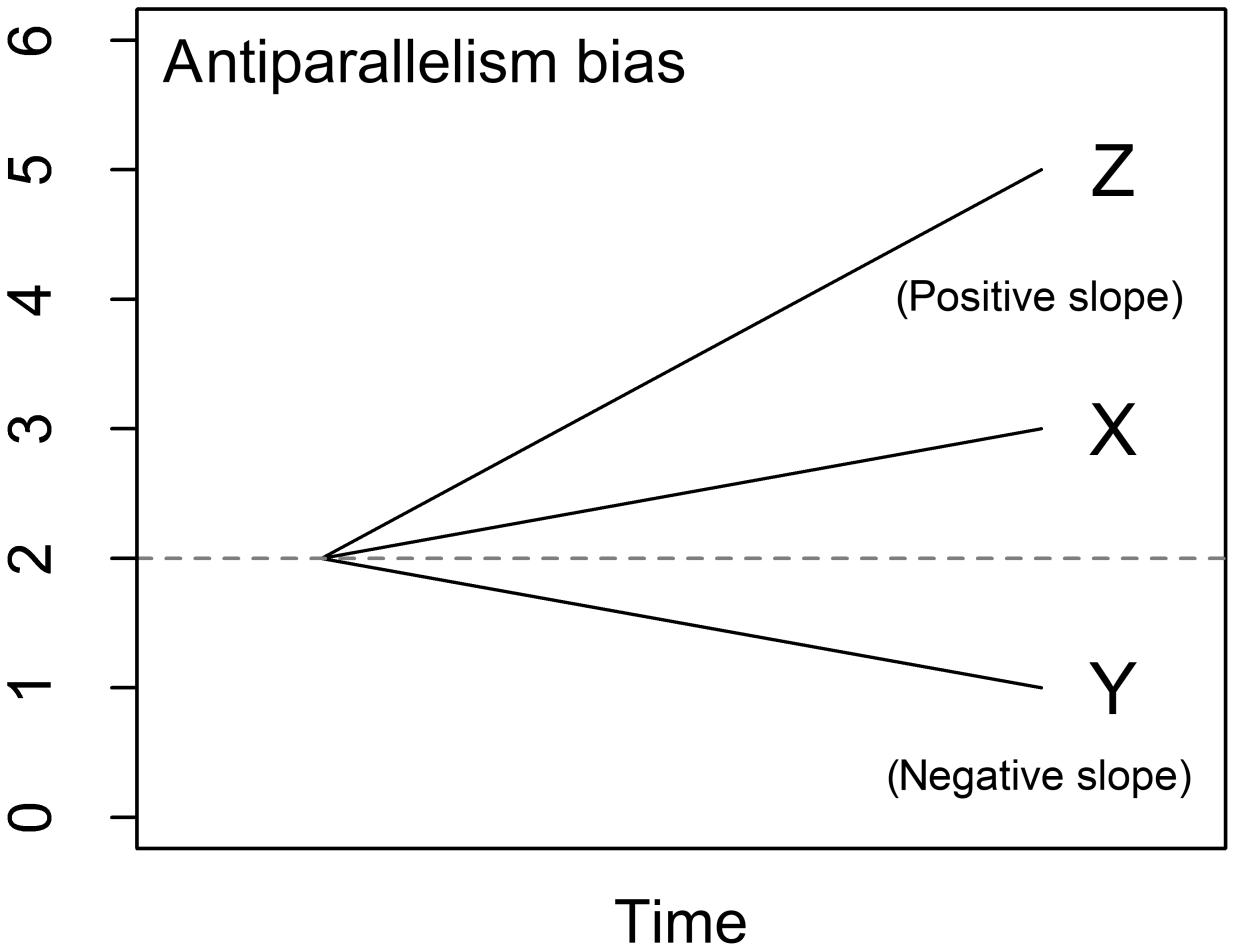
Illustration of antiparallelism bias. Time series X and Y are antiparallel (Y has the same slope as X, but in the opposite direction), while Z has a different slope than X, but in the same direction. The total difference in values between X and Z is the same as that between X and Y. Distance measures with positive antiparallelism bias rate time series X as more dissimilar to time series Z than to time series Y, while the opposite is true for those with negative antiparallelism bias. Distance measures with neutral antiparallelism bias rate the time series pairs as equally dissimilar.

- V1: Translation sensitivity (Figure 1a; adapted from Batyrshin et al., 2016). Translation refers to increasing the value of all observations of one time series by the same amount. It may be desirable to have a distance measure that is invariant to translation (the dissimilarity value does *not* change when one time series is translated) when time series have different starting values, for example, time series of carbon‐14 with different background levels.
- V2: Amplitude sensitivity (Figure 1b). Translation sensitivity on a local scale (sensitivity to translation of a section of a time series) will be referred to as amplitude sensitivity. This could be important, for example, in determining deviations in the strength of seasonal temperature patterns.
- V3: White noise sensitivity (sensitivity to random noise; Figure 1c; adapted from Lhermitte et al., 2011). Robustness against white noise (low sensitivity) might be desirable, for example, when comparing trends of stochastic processes such as population growth, where for example it could be assumed that sampling variation might be causing uncorrelated randomness in the time series.
- V4: Biased noise sensitivity (sensitivity to non‐random noise, i.e., noise in a single direction; Figure 1d; adapted from Lhermitte et al., 2011). An invariance or low sensitivity to biased noise might be important, for example, if comparing time series of vegetation density calculated from satellite images biased by differential cloud cover.
- V5: Outlier sensitivity (Figure 1e). Sensitivity to outliers is useful for detecting anomalies or disruptive events such as drought or storms, but robustness may be preferred where outliers represent measurement errors or irrelevant anomalies (e.g., sharp drops in abundance at survey sites due to predator presence).
- V6: Antiparallelism bias (Figure 2). Antiparallelism refers to line segments or trends which have slopes with the same value but opposite signs, while parallelism refers to those which have identical slopes in both value and sign. A distance measure with positive antiparallelism bias ignores the sign of the slope and treats antiparallel and parallel trend curves the same. A distance measure with negative antiparallelism bias treats trend curves with opposite signs as more dissimilar than those with identical signs. Distance measures with no antiparallelism bias (neutral) measure absolute differences on the *y*‐axis, without respect to slope or direction. Whether and which kind of antiparallelism bias is desirable depends on the application. For example, it might be important to differentiate between positive and negative fluctuations from a baseline value of energy flow, which would require a distance measure with a positive or negative antiparallelism bias; conversely, if the only concern was the magnitude of fluctuation, a neutral distance measure might be preferred.

### 2.3 Time‐based properties

- T1: Phase sensitivity (Figure 1f; adapted from Lhermitte et al., 2011). Phase sensitivity is the *x*‐axis equivalent of translation sensitivity; it describes how a dissimilarity value is affected by temporal shifting of all values of a time series by the same amount. Phase invariance may be a desirable property to detect similarities that occur separated in time. For example, when matching audio recordings of bird songs, it is likely that similar songs occur at different time points in different recordings. Conversely, when comparing population trends of different species within a community or geographical area to see which ones responded similarly to a disruptive event occurring at time *t*, phase invariance is not a desirable property as responses should match in time.
- T2: Time scaling sensitivity (Figure 1g; adapted from Esling & Agon, 2012). Time scaling refers to the expansion or compression of a time series along its time axis. Invariance to time scaling is useful for certain applications, such as comparing animal behavior patterns occurring at different speeds.
- T3: Warping sensitivity (Figure 1h; adapted from Batista et al., 2011). Local time scaling, involving the expansion or compression of one or more sections of a time series, rather than the entire series, will be referred to as warping. Invariance to warping is particularly useful when matching similar time series which have plateaus or valleys of uneven lengths. For example, recordings of bird calls may have pauses of different lengths but the same overall call pattern within species.
- T4: Frequency sensitivity (Figure 1i). If a distance measure is sensitive to frequency, increasing the number of differences between two time series should increase the dissimilarity value. This could be important, for example, to rank a set of environmental time series according to the number of deviations from a normal range to determine levels of climate destabilization.
- T5: Duration sensitivity (Figure 1j). This property is a special case of frequency sensitivity. Distance measures which are sensitive to duration must be sensitive to frequency, but the converse is not true. Continuing the example from T4, ranking a set of environmental time series according to the number of deviations from a normal range without respect to the lengths of those deviations would require a distance measure sensitive to frequency but not duration.

See Appendix A for more precise and detailed descriptions of properties V1–V6 and T1–T5.

### 2.4 Other properties

- N1: Non‐positive value handling. Some distance measures will not return results if the data contain negative values or zeros. This has implications for tasks such as classification, where it is common to first rescale time series values to [−1,1].

### 2.5 Properties tests

The metric properties of some distance measures are specified in the literature, but for others, it is unclear. Therefore, we devised a set of tests for metric properties (Appendix B). We confirmed the robustness of our tests by comparing our results to the literature for distance measures with known metric properties.

We performed two types of testing for non‐metric properties in this study. Controlled testing was performed on sets of short, simple time series to clearly demonstrate specific properties. We then measured relative sensitivity for most properties and separated the resulting values into five bins, which we designated as “very low,” “low,” “medium,” “high,” or “very high.” For phase, time scaling, and warping sensitivity tests, relative sensitivity results were not binned. Instead, distance measures were designated “sensitive” for a given property if the distance was directly dependent on the phase difference or degree of scaling or warping. For all sensitivities, distance measures were classified as “invariant” if they returned zero values for all time series pairs, “insensitive” if the same non‐zero value was returned for all time series pairs, or “unpredictable” if distance values varied but did not show a clear relationship. All measures that were unable to handle unequal‐length time series were designated “n/a” for uniform time scaling sensitivity and warping sensitivity.

Antiparallelism bias was tested by comparing pairs of time series that differed by the same relative amount in different directions. Distance measures were designated as “positive” biased if they gave a greater dissimilarity value to pairs of time series differing in opposite directions than to pairs differing in the same direction, “negative” biased if they gave a greater dissimilarity value to those differing in the same direction, or “neutral” if they assigned each pair of time series the same dissimilarity value.

**FIGURE 3:**
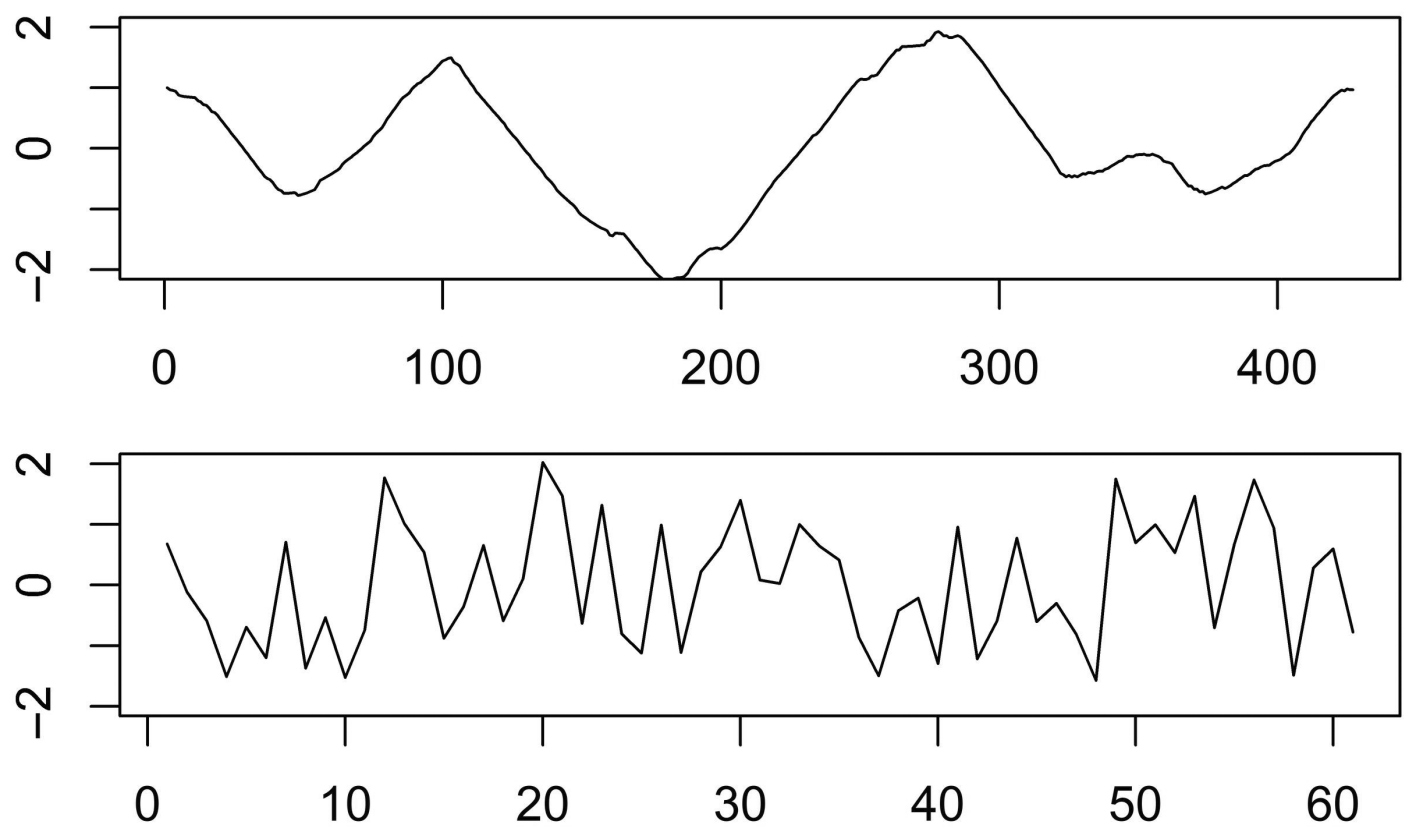
One time series from each of the Yoga (top) and Synthetic Control (bottom) datasets of the UCR Time Series Archive (Dau et al., 2019). Time series in the archive are z‐normalized. Therefore, we applied a translation shift before testing to ensure compatibility with distance measures that are unable to handle zeros or negative values.

To ensure the demonstrated properties translate onto real‐world datasets, we employed uncontrolled testing on two real‐world time series (Figure 3) from the UCR Time Series Classification Archive (Dau et al., 2019). One time series was randomly selected from the Yoga dataset, and represents body movement during pose transitions. Captured images of actors were converted to one‐dimensional time series by calculating the distance between the outline and its center. The other time series was randomly selected from the Synthetic Control dataset and is a synthetically generated pattern designed to be quantifiably similar or dissimilar to other time series in the dataset. Neither of these are ecological time series, but it does not matter for the purpose of generalized testing. We created a function for each property to be tested, which applies a transformation to one or more time points of a real‐world time series. For example, the translation function adds a real number q to every observation of a time series. The transformed time series is then compared with its unaltered counterpart. We applied the functions over a range of parameters, then plotted the resulting curves to show how responses of distance measures vary with magnitude. We did not compare them against a reference or assign sensitivity ratings as they were intended only as a confirmatory check against the results of controlled testing.

For a more detailed and technical explanation of properties testing, see Appendix B.

### 2.6 Correlation between distance measures

We used the relative sensitivity values (before binning) for properties V1–V5, T4, and T5 to test for correlations between distance measures, to determine how similarly related and unrelated distance measures responded to our properties tests. First, we calculated the Pearson correlation between each pair of distance measures. We then separated the results into pairwise correlations of distance measures within the same families and pairwise correlations of unrelated distance measures, and performed a Welch two‐sample *t*‐test to determine if distance measures within the same family or group are more closely correlated than unrelated distance measures.

### 2.7 Selection process

**FIGURE 4:**
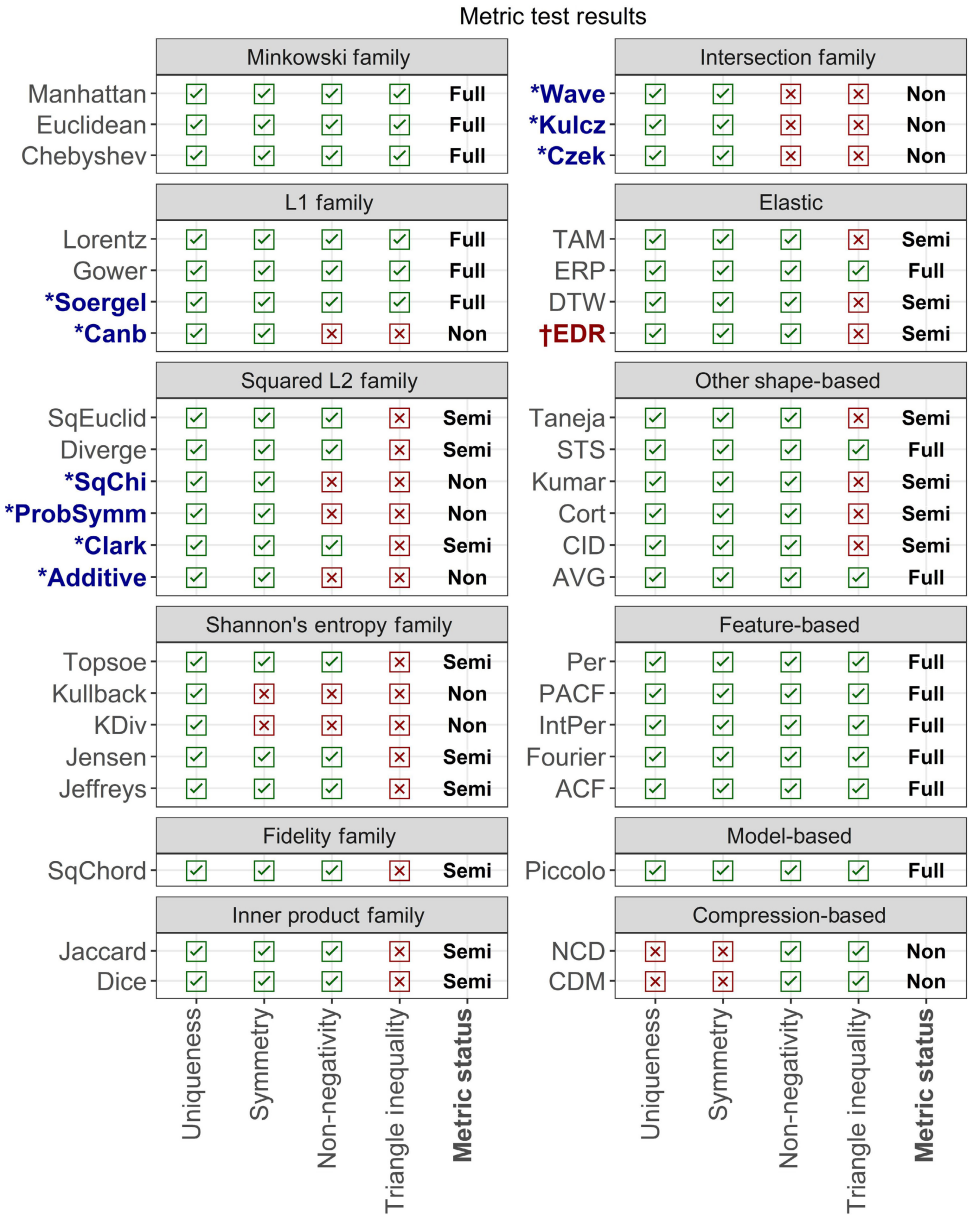
Metric test results for 42 distance measures. Results are arranged by family (for lock‐step shape‐based measures) or type. *These distances respond differently when inputs are constrained to non‐negative real numbers. As we included negative values in our tests, our results for these measures may differ from others (e.g. Kocher and Savoy, 2017). ^†^This distance is a full metric when the threshold value (epsilon) is set at 0.

**FIGURE 5:**
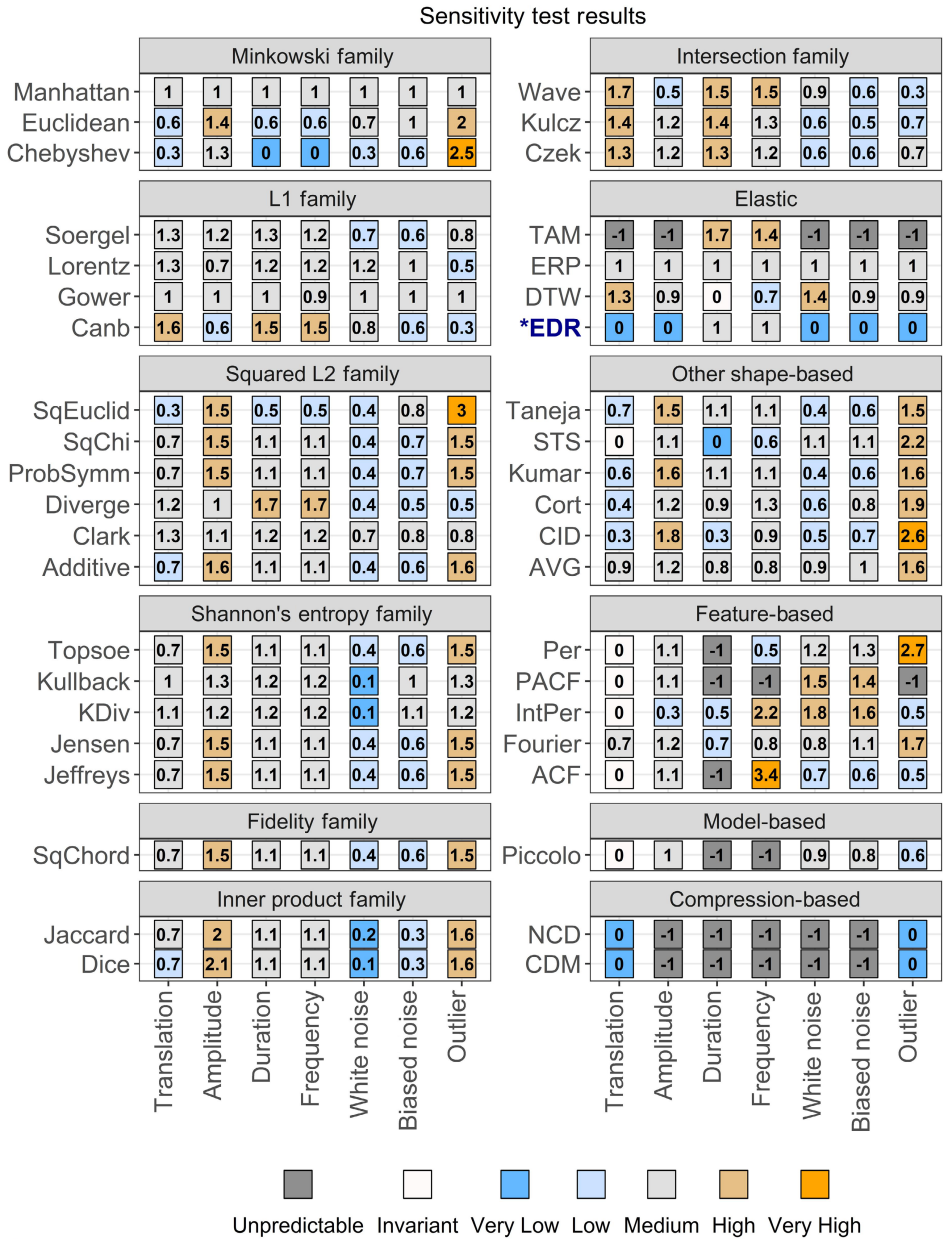
Sensitivity test results for 42 distance measures. Results are arranged by family (shape‐based measures) or type, and color‐coded according to sensitivity value. Sensitivity ranges: very low: <0.2, low: 0.2–0.7, medium: 0.7–1.3, high:1.3–2.5, very high: >2.5. *The results for EDR strongly depended on the threshold setting, epsilon. Here it was set to 0.1.

**FIGURE 6:**
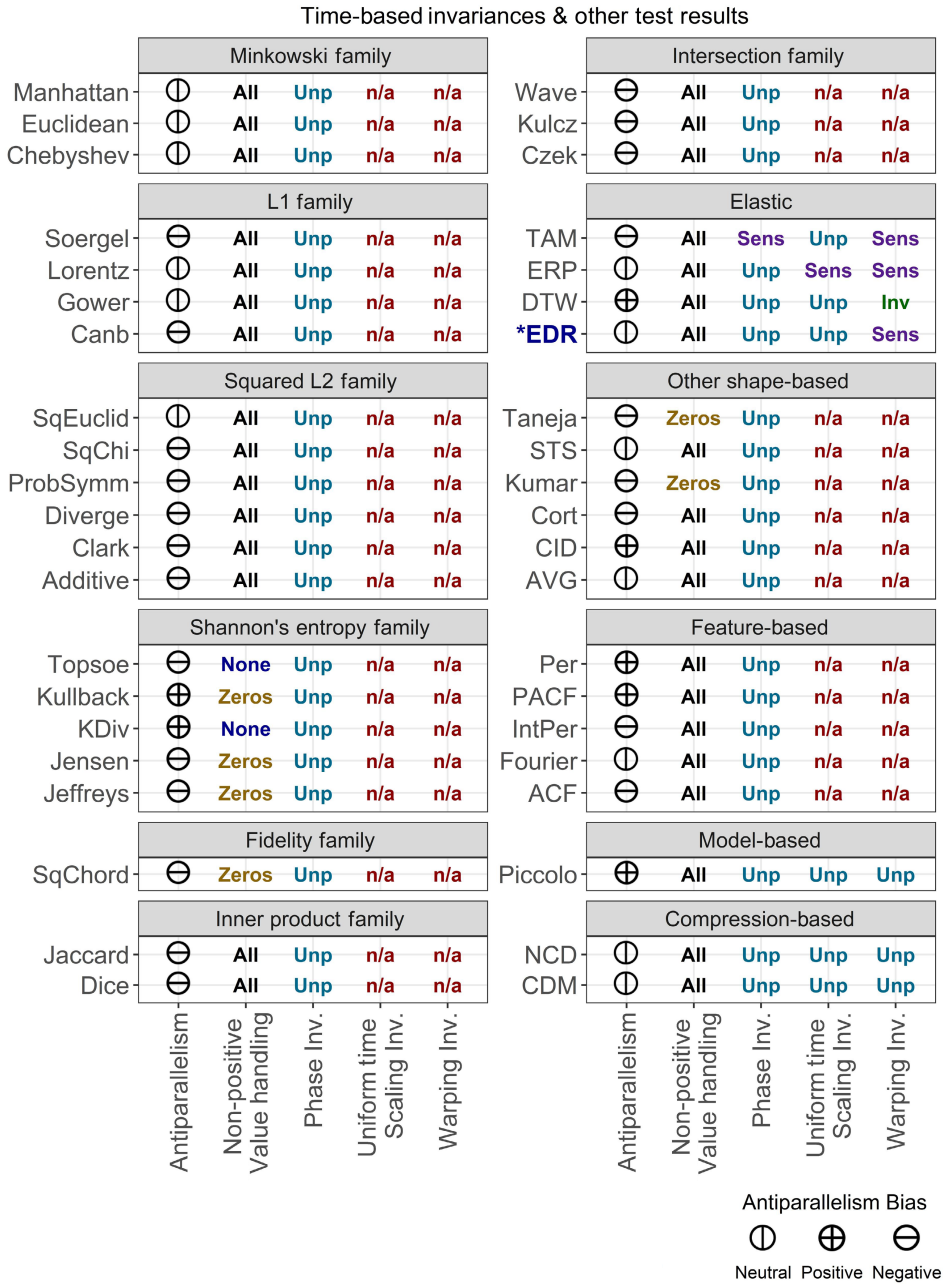
Test results for antiparallelism bias, non‐positive value handling, and time‐related sensitivities for 42 distance measures. Results of “n/a” for uniform time scaling sensitivity and warping sensitivity mean that the distance measure in question is unable to handle unequal‐length time series and therefore could not be tested for those properties. sens = Sensitive, Ins = Insensitive, Inv = Invariant, Unp = Unpredictable. *For this distance measure, results differ depending on the threshold value, epsilon. Here, epsilon was set to 0.1.

We devised a three‐step selection process to guide researchers through determining the most appropriate distance measure(s) for their intended application. The selection process utilizes a set of purpose‐built tools that we created by combining the results of our properties tests with existing knowledge from the literature (especially Esling & Agon, 2012). The first step is to use a decision tree (Figures 8 and 9) to select a general category of distance measures. Step two is to use Table 1 to determine which pre‐processing steps might be necessary to prepare the dataset and/or to further narrow the choice of distance measures. The final step is to determine which properties will be most important to achieve the desired outcome and use Figures 4, 5, 6 to narrow the selection to the distance measure(s) that exhibit these properties.

**TABLE 1:** Solutions to potential issues in the data.

Problem	Pre‐processing solution	Properties‐based solution
Missing data points	Interpolate missing values	Choose an elastic distance. They handle gaps through one‐to‐none or one‐to‐many point matching
Different starting values but similar value scales	Apply a translation shift	Choose a distance measure invariant (or sensitive) to translation
Different value scales	Normalize or standardize data	
Zeroes or negative values	Transform data to obtain positive values	Choose a distance with non‐positive value handling
Noise	Apply a smoothing algorithm	Choose a distance measure robust (or sensitive) to the type of noise that is of concern
Out of phase		Choose a phase invariant (or phase sensitive) distance measure
Unequal lengths	Cut all time series to the same length	Choose an elastic, model‐based, or compression‐based distance measure
Different time scales		Choose a distance measure invariant (or sensitive) to uniform time scaling
Nonuniform sampling intervals	Interpolate intermediate values	Choose a distance measure that incorporates temporal information, such as the STS distance

*Note*: Choice of invariance or sensitivity as a solution should depend on whether the difference in question is important.

### 2.8 Real‐world example dataset

To demonstrate the selection process and add real‐world context, we used a dataset from a study of conservation impact of wet grassland reserves on breeding birds in the UK (Jellesmark et al., 2021). The dataset consists of 25 years of breeding pair count data for five wading bird species, from within and outside of reserves. The within‐reserves data came from 47 RSPB lowland wet grassland reserves, while the counterfactual (outside of reserves) data was selected from the UK Breeding Bird Survey data. Data were matched to select sites that represent how reserve land would look in the absence of conservation measures. The reserve and counterfactual count data were aggregated into species trends and then converted to indices by dividing each annual species count total by the first‐year species count total. Thus, each of the five bird species was represented with a reserve trend index and a matched counterfactual trend index. Jellesmark et al. (2021) compared each pair of indices to determine the effects of conservation efforts on each bird species, by calculating the percentage improvement of reserve indices over counterfactual indices and performing *t*‐tests to determine significance and effect size of the difference. We ranked the results of Jellesmark et al. (2021) according to both percentage improvement and effect size. We then applied our selection method to select appropriate distance measures, ranked the dissimilarity results returned by each selected distance measure, and examined the rankings with respect to Jellesmark et al. (2021). We also ranked the results returned by unselected distance measures for comparison.

## 3 RESULTS

### 3.1 Metric test results

Fourteen out of 42 distance measures were identified as full metrics, meaning they passed the metric tests for reflexivity, symmetry, non‐negativity, and the triangle inequality (Figure 4). Sixteen distance measures were identified as semi‐metrics (failed the triangle inequality test but passed the other three tests) and 12 were identified as non‐metrics (failed at least one of the tests for reflexivity, symmetry, or non‐negativity; Figure 4). However, in some cases results depended on settings or input values (some distance measures passed the triangle inequality and/or non‐negativity tests only when inputs were constrained to non‐negative real numbers). All tested feature‐based and model‐based distances were full metrics, while all tested compression‐based distances were non‐metrics. Shape‐based measures showed mixed results, even within families and groups.

### 3.2 Sensitivity test results

**FIGURE C1:**
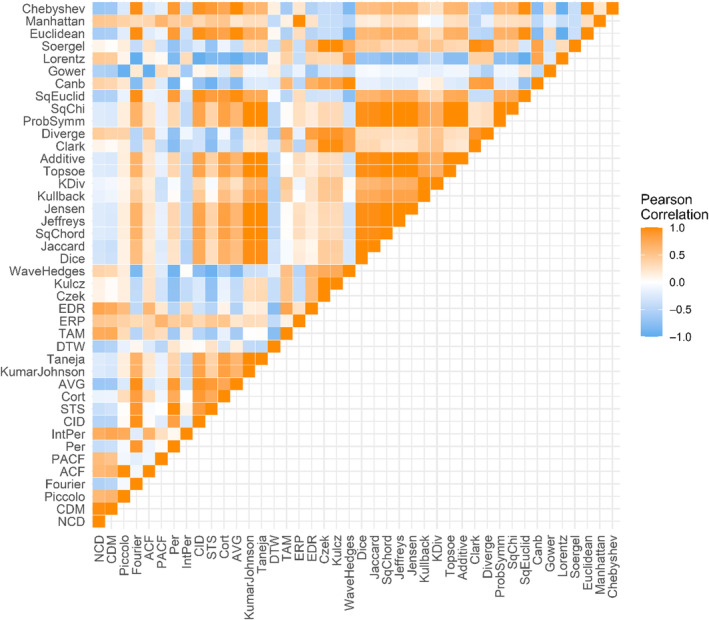
Pairwise Pearson correlation of all tested distance measures, based on the unbinned results of controlled sensitivities testing for translation, amplitude, duration, frequency, white noise, biased noise, and outliers. Distance measures are organized by family on the plot.

Lock‐step shape‐based measures varied in the strength of responses to the sensitivity tests, but none tested as unpredictable and only two (the Chebyshev distance and the Short Time Series, or STS, distance) showed any invariances or insensitivities (Figure 5; also, see Figures 1 and 2 for illustrations of the time series distortions we used to test for sensitivities and invariances). The Welch two‐sample *t*‐test shows that correlations between distance measures within families or groups (mean Pearson correlation = 0.48) are significantly stronger than between unrelated distance measures (mean Pearson correlation = 0.15; *t* = 5.5, df = 82.3, *p* < .001). However, not all related distance measures were closely correlated (see Figure C1), nor were there clear differences between families of distance measures. Elastic, feature‐based, and model‐based distances showed greater variation in responses, with insensitivities, invariances, and unpredictability being common. The two compression‐based distances we tested responded unpredictably to all controlled tests except translation and outliers; They responded unpredictably to *all* uncontrolled tests without exception. See Appendix for more detailed results. Overall, these results imply that choice of measure for a particular application needs to go beyond family.

### 3.3 Time‐based sensitivities and other test results

All distance measures except the Time Alignment Measurement (TAM) distance responded unpredictably to phase sensitivity testing (Figure 6; also, see Figures 1 and 2 for illustrations of the time series distortions we used to test for sensitivities and invariances). TAM was sensitive to phase changes; however, the response curve in uncontrolled testing was not smooth, suggesting some level of unpredictability. The Edit Distance with Real Penalty (ERP) distance was sensitive to uniform time scaling, while all other distances either responded unpredictably or were unable to be tested due to an inability to handle unequal‐length time series. Warping sensitivity was more common, occurring in three elastic distance measures. DTW tested as invariant to warping and was thus the only distance measure we tested with any time‐based invariances. Elastic measures were the only group of distance measures that showed any predictable time‐based sensitivities or time‐based invariances.

Two distance measures in Shannon's entropy family were unable to deal with zeros, while the entire family was unable to deal with negative values. Three other lock‐step shape‐based measures also showed an inability to deal with negative values. Antiparallelism bias showed no obvious group‐based patterns, but negative antiparallelism bias was most common and positive bias was least common.

**FIGURE C2:**
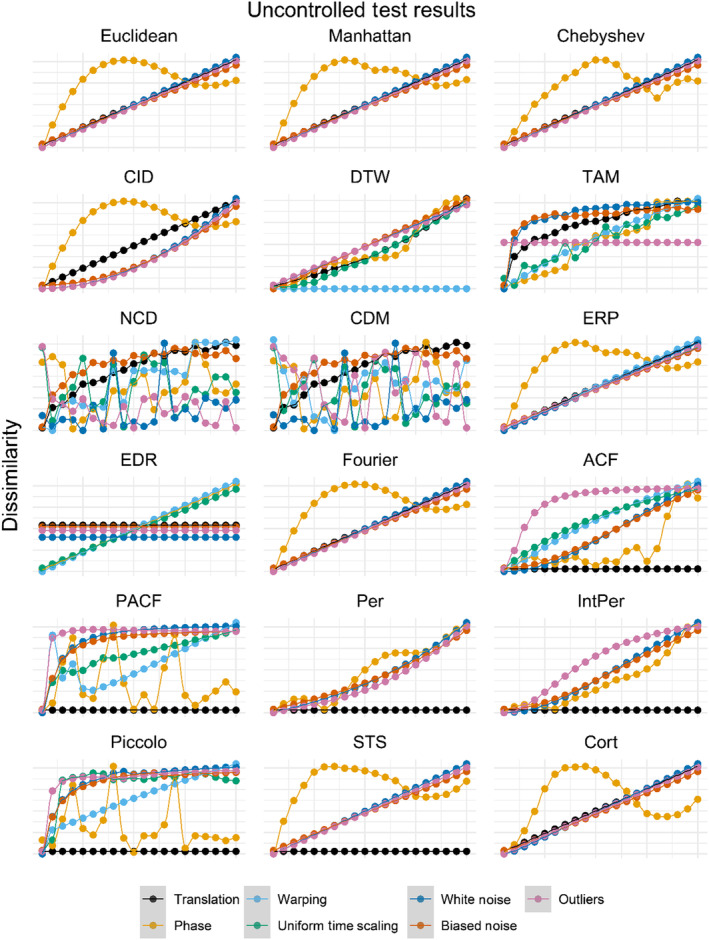
Dissimilarity measurements from 17 distance measures of the TSdist package after applying transformations to a randomly selected time series from the Yoga dataset of the UCR Time Series Archive. The *x*‐axis depicts the transformation value *q* across a range of 1–200 in increments of 10. Dissimilarity values were rescaled using Min–Max scaling to a range of [0,1] to ensure that the shape of each response curve would be visible regardless of the strength of the response.

**FIGURE C3:**
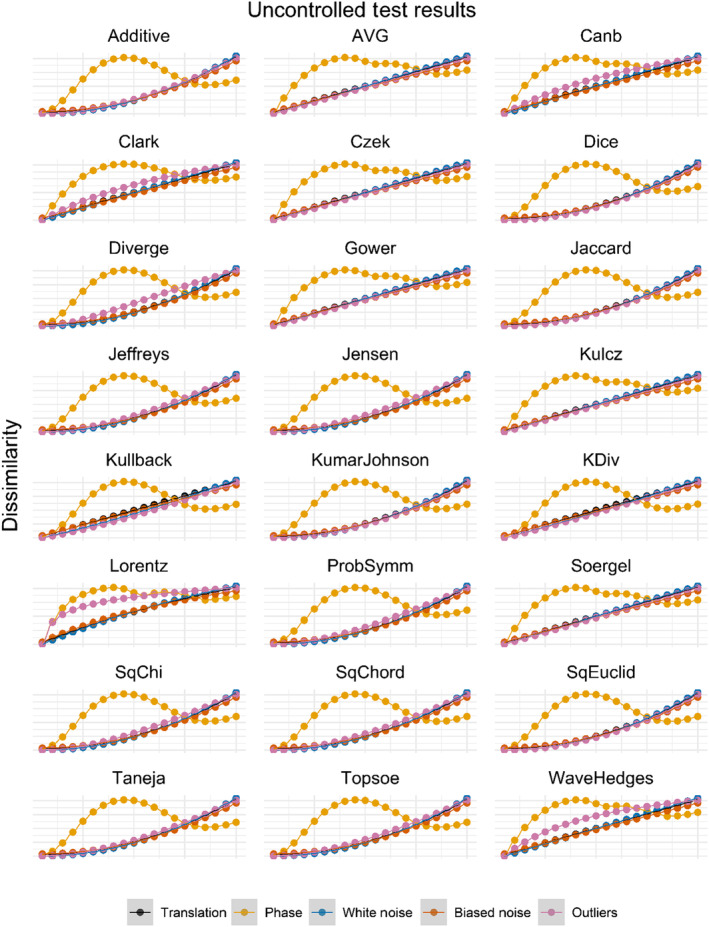
Dissimilarity measurements from 24 distance measures of the philentropy package after applying transformations to a randomly selected time series from the Yoga dataset of the UCR Time Series Archive. The *x*‐axis depicts the transformation value *q* across a range of 1–200 in increments of 10. Dissimilarity values were rescaled using Min‐Max scaling to a range of [0,1] to ensure that the shape of each response curve would be visible regardless of the strength of the response.

**FIGURE C4:**
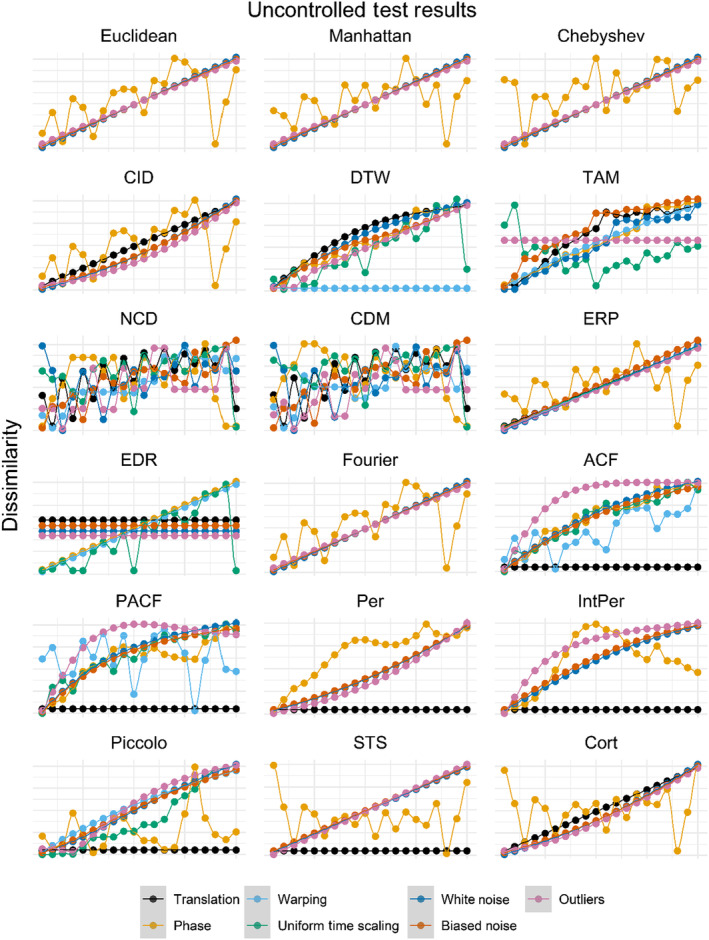
Dissimilarity measurements from 17 distance measures of the TSdist package after applying transformations to a randomly selected time series from the Synthetic Control dataset of the UCR Time‐Series Archive. The *x*‐axis depicts the transformation value *q* across a range of 1–20 in increments of 1. Dissimilarity values were rescaled using Min–Max scaling to a range of [0,1] to ensure that the shape of each response curve would be visible regardless of the strength of the response.

**FIGURE C5:**
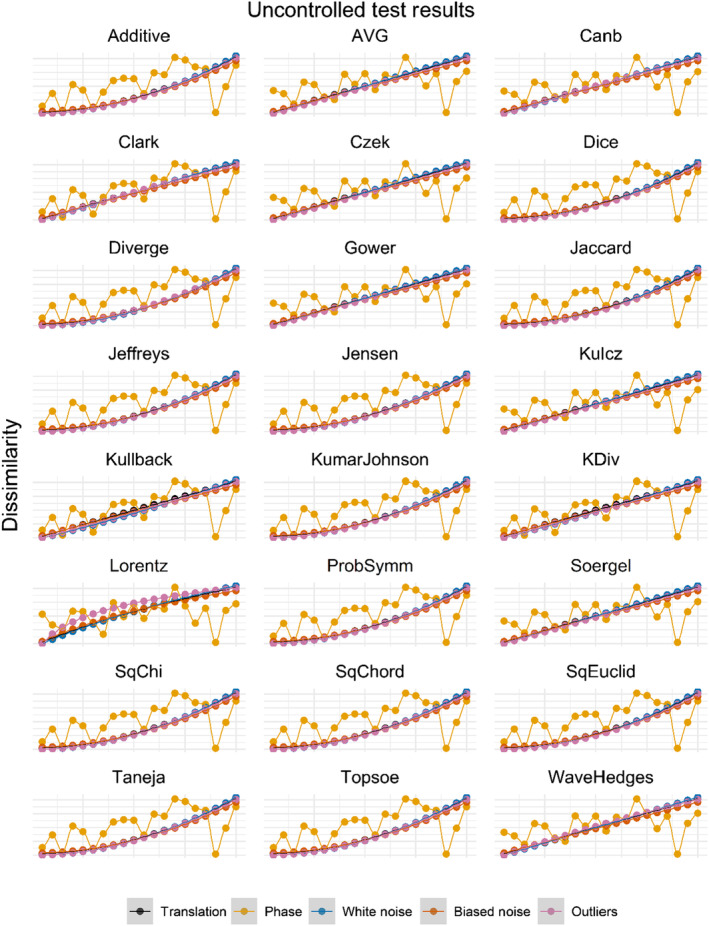
Dissimilarity measurements from 24 distance measures of the philentropy package after applying transformations to a randomly selected time series from the Synthetic Control dataset of the UCR Time Series Archive. The *x*‐axis depicts the transformation value *q* across a range of 1–20 in increments of 1. Dissimilarity values were rescaled using Min‐Max scaling to a range of [0,1] to ensure that the shape of each response curve would be visible regardless of the strength of the response.

Uncontrolled test results were largely consistent with the controlled test results in Figures 5 and 6 (see Appendix, especially Figures C2, C3, C4, C5).

### 3.4 Selection process and applications

The distance measure selection process we describe and demonstrate here was developed using the results from this study in combination with existing literature and is intended to be useful for any dataset and task the user might have in mind. The first step in the selection process should be to determine the task to be performed. Both the dataset and the intended task are important in selecting an appropriate distance measure. For example, in classification, generally the entire shape of the time series is important, while anomaly detection might work best with distance measures that are especially sensitive to outliers. Classifying bird species according to their songs may require flexibility on the time axis (e.g., warping invariance), while clustering fish populations according to changes in biomass over a set time period does not.

**FIGURE 7:**
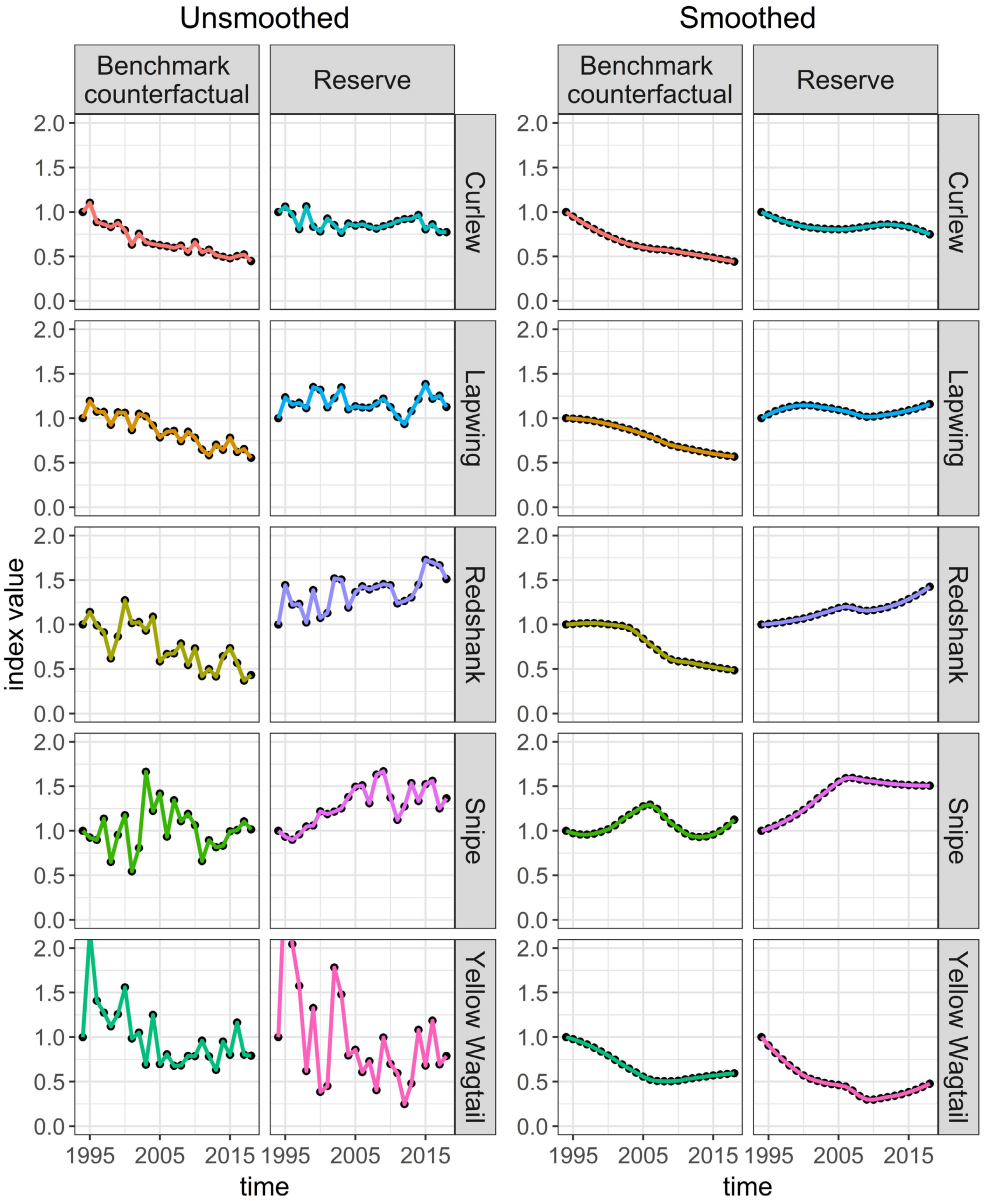
Reserve and counterfactual trends for five wading bird species that breed on RSPB lowland wet grassland reserves in the UK. Left: Unsmoothed trends based on original data presented in Jellesmark et al. (2021). Right: LOESS smoothed trends with a span setting of 0.75.

We demonstrate the process of selecting an appropriate distance measure using a real‐life example dataset from a study that used trends from wading birds inside and outside of reserves to determine the conservation impact of reserves (Figure 7; see detailed description in section 2.8; also Jellesmark et al., 2021). A greater difference between the trend within reserves and the corresponding counterfactual trend outside of reserves means greater conservation impact on a given wading bird species. We chose this example because it is a type of application that many readers will be unfamiliar with in the context of distance measures, and because the results can be compared with other methods.

**FIGURE 8:**
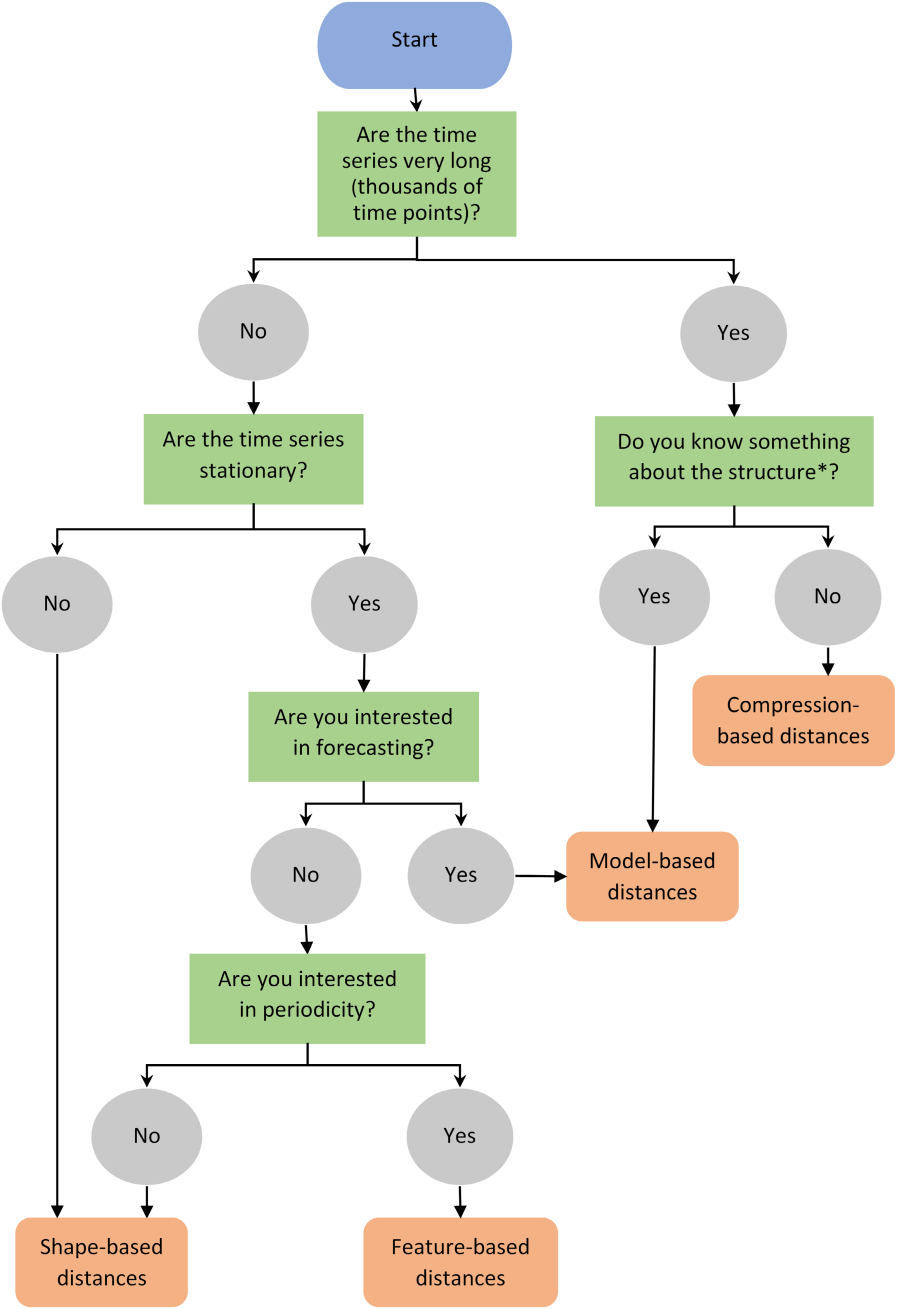
Decision tree to aid in choosing a distance measure category. *Structure refers to trends, repeated patterns, spikes, etc.

**FIGURE 9:**
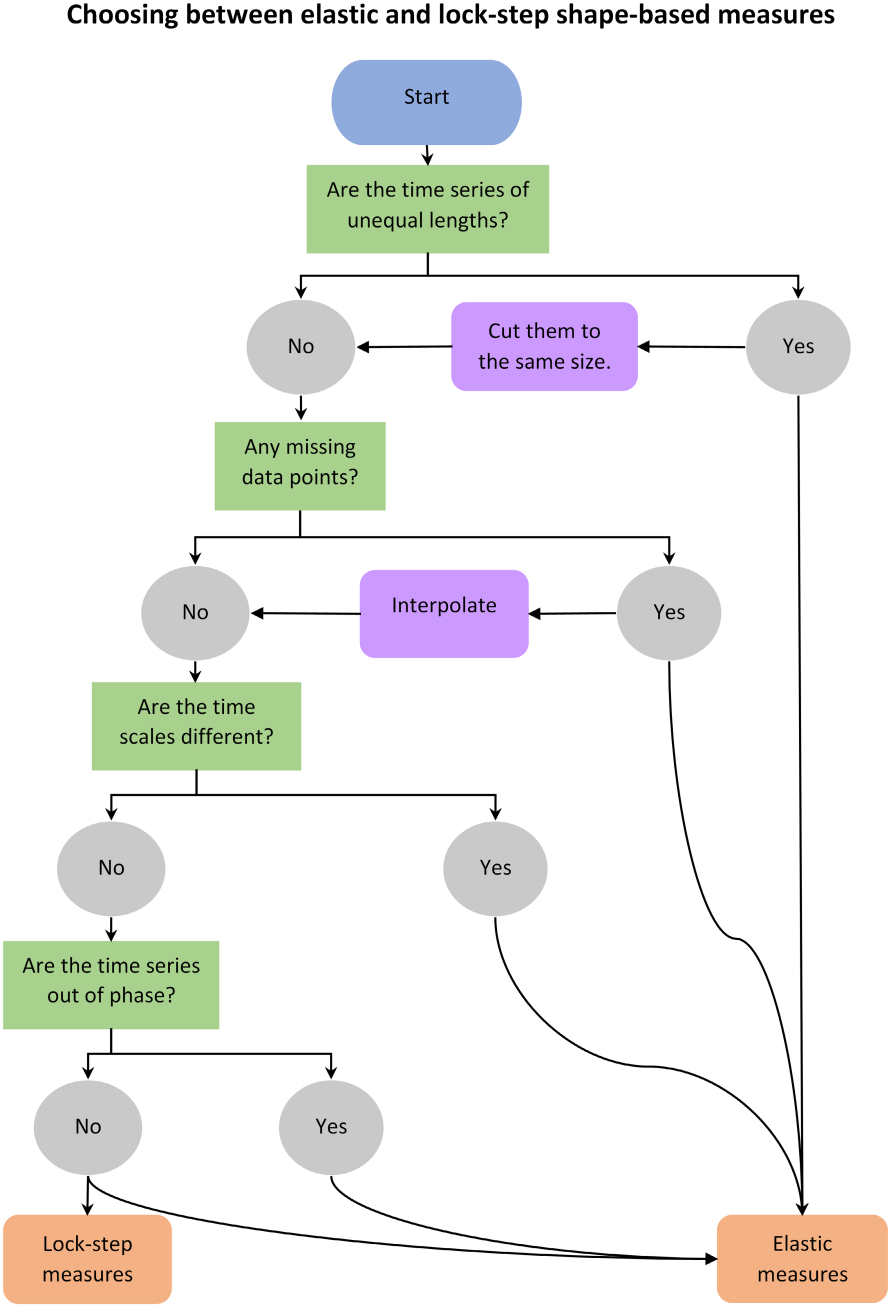
Decision tree to aid in choosing a sub‐category of shape‐based distance measures.

We began by examining our wading bird dataset in context of the decision trees in Figures 8 and 9. The dataset consisted exclusively of short (25 data points), non‐stationary time series. Following Figure 8, we focused on shape‐based distance measures, which compare raw data values. As the time series were of equal length, in phase, using the same time scale, and without any missing data points, both lock‐step and elastic measures would be appropriate (Figure 9).

Next, we worked through Table 1. As our wading bird trends were indexed to a starting value of one (Figure 7), they had the same starting value and the same value scale. There were no negative values because the trends were indexed and based on wetland bird counts; nor were there any zeroes. However, we did notice that some of our time series were noisy (Figure 7), which could obscure the trends. Noise is a common characteristic of population data, largely due to the stochasticity of population dynamics and the environmental variables they depend on (Vasseur & Yodzis, 2004). While this noise is often white (random, uncorrelated), biased “red” noise (positively autocorrelated, tending toward a single direction) is also common, for example, when environmental conditions are above or below average for an extended period (van de Pol et al., 2011; Vasseur & Yodzis, 2004). Biased noise is therefore more likely to represent a legitimate difference in trends. There are multiple ways to deal with noisy time series (Table 1). We first tried the properties‐based solution (Table 1; see below for the pre‐processing solution). Using Figure 5, we filtered out all shape‐based distance measures with a white noise sensitivity category of medium or higher (a sensitivity value of 0.7 or more). Next, we required biased noise to be at least two categories higher in sensitivity than white noise (Figure 5, e.g., if white noise sensitivity was very low, biased noise sensitivity must be at least medium). Our choices here were based on practicality; sensitivity categories are arbitrary (we categorized them for convenience), so we wanted to avoid being too specific while ensuring that any chosen distance measure exhibited a non‐trivial difference in sensitivity between white noise and biased noise.

Finally, we considered the remaining properties in the context of our intended task and desired outcome. We deemed amplitude sensitivity to be important, as we were interested in the overall divergence between population indices within and outside reserves. Duration sensitivity was also important, as we would consider population indices which diverge more steeply or for a longer period to be more different, that is, that conservation measures had a stronger effect on these species. Therefore, both amplitude and duration sensitivity had to be at least low (a sensitivity value of 0.2 or higher; Figure 5). Again, we could have chosen a different (higher) category, but we were more concerned with making sure the distance measures exhibited *some* sensitivity to these properties than the exact degree of sensitivity. We did not filter for antiparallelism bias, as the high stochasticity in some of our time series (Figure 7) would dilute the signal too much for it to matter.

**FIGURE 10:**
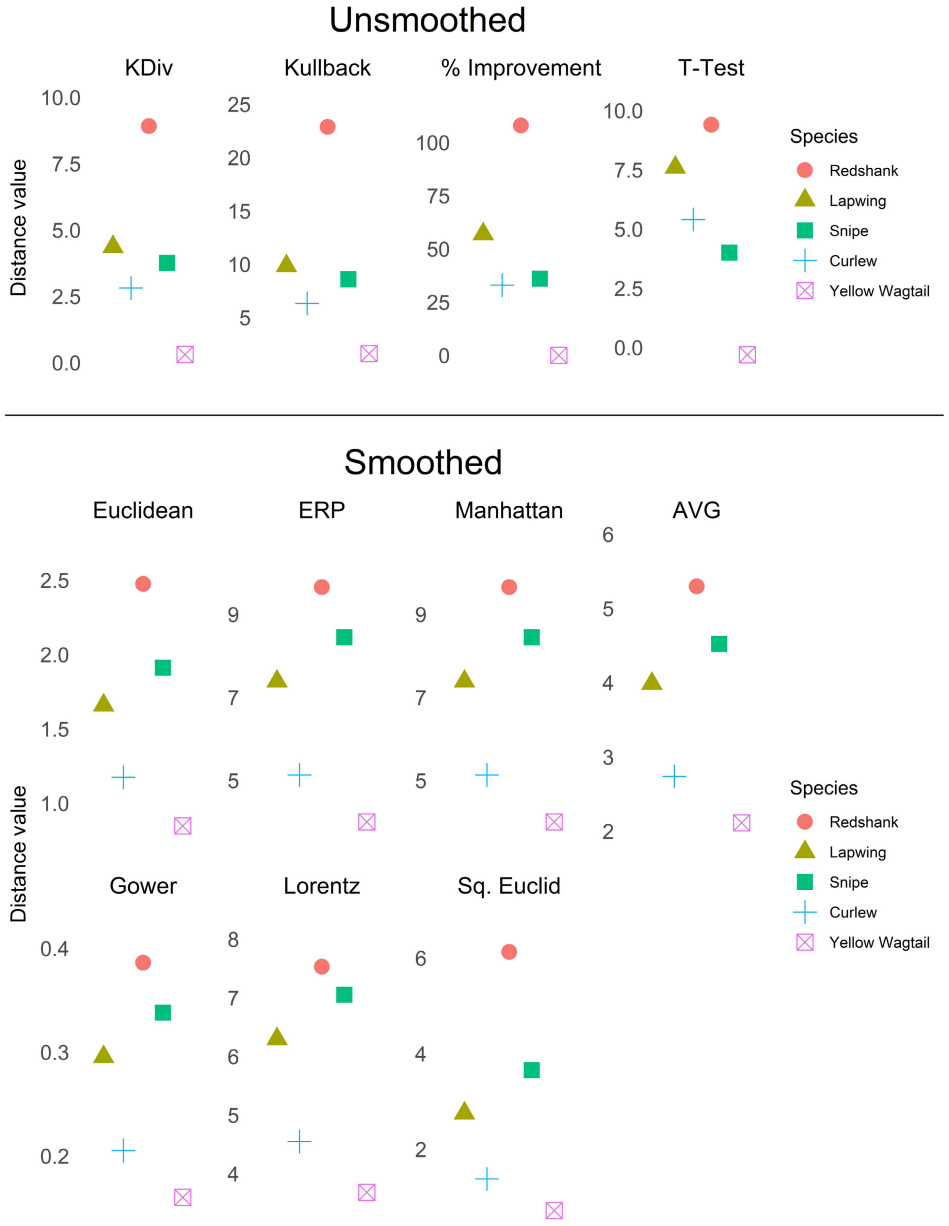
Comparative rankings of conservation impact on five wading bird species. Values on the *y*‐axis represent the distance between unsmoothed (top) or LOESS smoothed (bottom) reserve and counterfactual trends for each species. Results are from the distance measures chosen by our selection process, as well as the percent improvement and *t*‐test methods (top) used by Jellesmark et al. (2021). Percent improvement is the difference (multiplied by 100) between the final year index values of the two trends (within and outside of reserves) for a given bird species, while the t‐test represents the results of a Welch two‐sample *t*‐test between the two trends.

**FIGURE D1:**
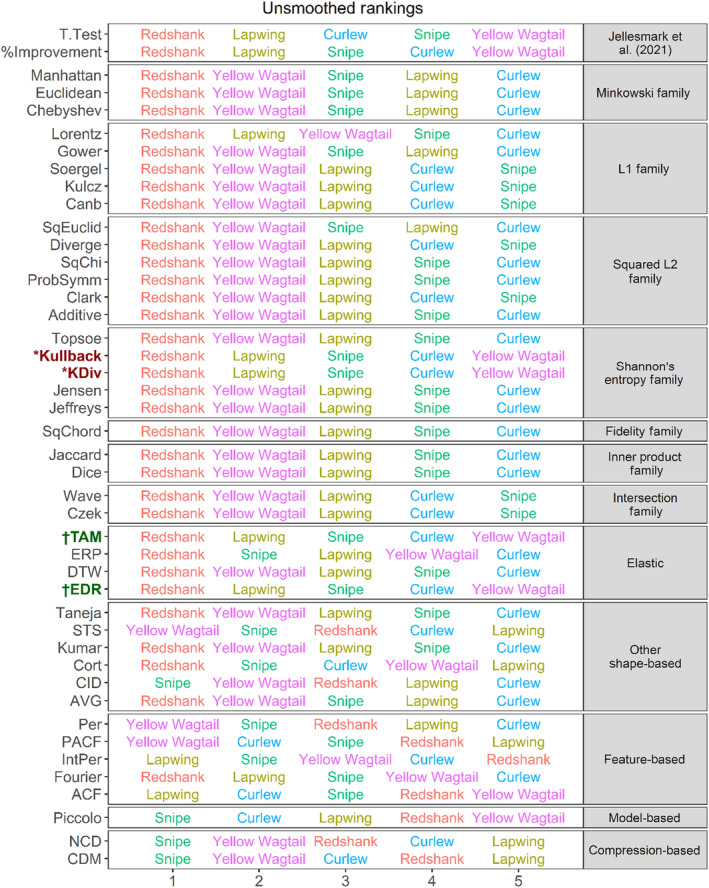
Comparative rankings of conservation impact on unsmoothed trends of five wading bird species according to percent improvement, *t*‐test, and distance measures. Species ranked first had the greatest difference between trends. *Starred distance measures were chosen by our selection process. ^†^Daggered distance measures were not chosen, but returned the same results as the chosen measures.

This selection process left us with two distance measures: the K Divergence (KDiv) and the Kullback–Leibler distance (Kullback), both of which returned the same rankings that Jellesmark et al. (2021) obtained using percent improvement (Figure 10). Only two of the 40 unselected distance measures, the Edit Distance for Real Sequences (EDR) and TAM, returned the same rankings (see Figure D1 in Appendix D).

Another way of dealing with noisy time series is by applying a smoothing algorithm (Table 1). We applied a LOESS smoothing algorithm (span = 0.75) to all time series in the dataset to remove the noise and reveal the trends (Figure 7). We then re‐ran the selection process using the same settings, except that we did not filter for noise sensitivity, and we added a filter for antiparallelism bias. Antiparallelism bias is not very important when dealing with highly stochastic time series because the signals for slope and direction are muddied by noise; however, smoothing introduces strong positive autocorrelation, making the slope and direction signals clear. We selected neutral for antiparallelism bias (Figure 5) because we were more interested in relative differences in the population indices than the direction of change.

**FIGURE D2:**
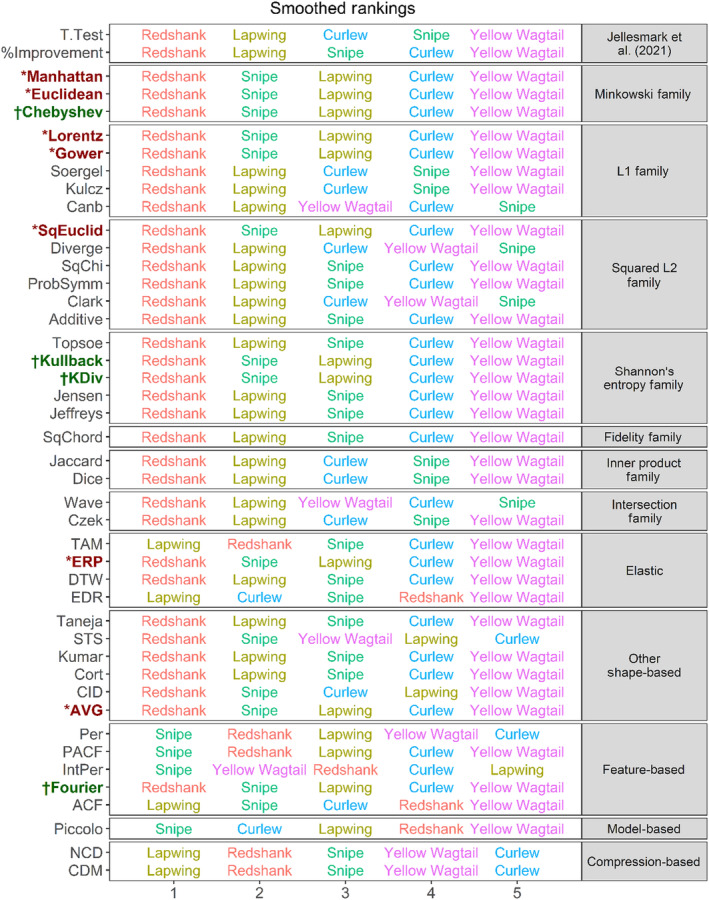
Comparative rankings of conservation impact on smoothed trends of five wading bird species according to percent improvement, *t*‐test, and distance measures. Species ranked first had the greatest difference between trends. *Starred distance measures were chosen by our selection process. ^†^Daggered distance measures were not chosen, but returned the same results as the chosen measures.

We were left with seven distance measures: ERP, the Euclidean distance, the Manhattan distance, the Gower distance, the Lorentzian distance (Lorentz), the Average distance (AVG), and the Squared Euclidean distance (Sq. Euclid). All seven selected distance measures agreed on the following order: Redshank, Snipe, Lapwing, Curlew, Yellow Wagtail (Figure 10). Four of the 35 unselected distance measures returned the same results (see Figure D2 in Appendix D).

## 4 DISCUSSION

The aim of this study was to provide enough information to make informed, objective decisions about which distance measures to use to compare ecological time series data. We tested 42 distance measures for 16 properties and presented an objective method of selecting distance measures for any task based on those properties. We demonstrated the viability of the method on a real‐world dataset by selecting distance measures to rank differences between pairs of wading bird population trends (within and outside of reserves) and showing that the distance measures we selected were fit‐for‐purpose and consistent in their rankings. Importantly, we found that different conclusions would be reached when choosing a measure at random, or based upon different criteria. The method is user‐directed; therefore, success depends on an understanding of the dataset, the task to be performed, and the hoped‐for outcome.

Time series length and stationarity inform what category of distance measures the user should focus on (Figure 8). Shape‐based distances are best for short time series with differences that are easy to visualize, while longer, stationary time series may be better suited to feature‐based, model‐based, or compression‐based distance measures (Esling & Agon, 2012). Most times series for abundances or biomass are relatively short, meaning shape‐based measures are probably most suitable, but other applications may have more data points, although the choice will depend on other aspects of the question (discussed more below).

Most distance measures we tested are lock‐step measures. While we have categorized many of them by family, it is not evident from our testing that there is enough similarity between distance measures within families for this categorization to be of much use. While there are clear differences in sensitivities between lock‐step measures, they share a rigidity in their treatment of time, comparing all point pairs 1‐to‐1, and most lack invariances. This makes them best‐suited to applications where sampling is repetitive (e.g., yearly) and standardized in time, such as long‐term population trends. Elastic measures, such as DTW, have tremendous flexibility due to their ability to match multiple time points to a single time point and are therefore best used when time series have different time structures, such as recordings of animal calls or movements.

The broadest difference in use cases occurs between shape‐based and non‐shape‐based distance measures. Feature‐based and model‐based measures are typically used to compare stationary time series, which are time series whose parameters (e.g., mean and variance) are not time‐dependent, as well as non‐stationary time series that are characterized by repeating patterns rather than stochasticity. Model‐based and feature‐based measures identify particular aspects of these time series; thus, their uses tend to be more specific than shape‐based measures. They are especially useful for prediction, as repeating patterns can be forecast into the future. For example, they might be used to classify or predict time series of environmental parameters (temperature, pollution, etc.), or events or changes that fluctuate or reoccur seasonally or diurnally. Compression‐based measures are designed to be extremely general and can theoretically be applied to any kind of time series. However, in practice we did not find them to be of any use on the time series we used for testing. They were unpredictable and did not demonstrate their purported metric properties. They are better suited to much longer time series (many thousands or even millions of time points), but these are very rare in ecological surveys.

The results of our properties tests showed a variation in strength of sensitivity to different properties in different distance measures, although most distance measures were highly sensitive to outliers. Invariances were uncommon among the distance measures we tested, although several distance measures did demonstrate invariance to translation. Some distance measures, such as EDR and ERP, have tuning parameters that may affect their behavior. In the case of ERP, these parameters can determine whether and how sensitive it is to missing values; in the case of EDR, the threshold setting determines how far apart values must be to be considered different and therefore serves to toggle responses to multiple properties between invariance and sensitivity.

When dealing with time series of unequal‐length or missing data points, distance measures that allow unequal matching (e.g., matching multiple points to one point), such as DTW, or that allow gaps, such as ERP, may be the solution. Alternatively, pre‐processing of data may remove such concerns. For example, missing data points can be filled in by interpolation, or longer time series can be cut to the same length as shorter ones (only attempt such solutions if they make sense for the data).

Elastic measures, such as DTW, EDR, and ERP, are the most versatile distance measures, able to handle many common complications of datasets with little or no pre‐processing. For general tasks, they are often a good option (see our decision tree: Figures 8 and 9). However, for tasks involving large datasets containing thousands of time series, some elastic measures may be impractical due to processing speed. Much of the research into speeding up time series comparisons for large datasets has focused on a select few distance measures, especially the Euclidean distance and DTW. While the Euclidean distance is faster, better known, and still widely used in some fields, an extensive body of research has shown DTW to be more accurate (Dau et al., 2019; Paparrizos et al., 2020; Zhu et al., 2012), and it is considered the de facto standard for accuracy in classification (note that it is still important to consider the properties of DTW in relation to the data, as it does not perform well in every case). Despite this, it is rarely used in ecology (Hegg & Kennedy, 2021). Note, however, that DTW is computationally expensive and therefore can be slow for large datasets (for discussion on ways to speed up DTW, see Appendix E).

For many analyses involving distance measures, researchers may first want to normalize or standardize their data or translate it along the *y*‐axis. This may be an important step if the time series use different scales or have different starting values. For example, when performing classification or clustering tasks, it is common to apply z‐normalization to rescale time series to a mean of zero and standard deviation of one (Rakthanmanon et al., 2013). Min–max normalization to a scale of [0,1] or [−1,1] is also common for datasets that are not normally distributed. Be aware, however, that these transformations may affect the subsequent choice of distance measures, as some cannot handle zeros or negative values and some metrics are non‐metric when there are negative values present (see Figure 4).

Although we ignored the metric properties of distance measures for our real‐world example, they are very important for some tasks. For example, many algorithms for classification and clustering are designed to work only in metric space and may return unexpected results for non‐metric distances, while some classification and clustering problems require a semi‐ or non‐metric to get meaningful results (Weinshall et al., 1998). Another thing to be aware of is that output values (distances) returned by distance measures can be on dramatically different scales. Some, such as the Jaccard distance, are confined to [0,1], while others go to positive infinity [0,∞) (e.g., the Euclidean distance), or even include negative values (any distance that does not satisfy non‐negativity, e.g., the Canberra distance). Depending on the intended application, the output scale could affect analysis, so may be worth considering.

Noise is a common aspect of ecological time series, as environmental and population dynamics are stochastic. There are several potential ways to deal with noisy time series. Some distance measures, such as EDR, have threshold settings; any difference between time series that falls below the threshold will be ignored. If the noise is relatively uniform in amplitude, this may be a simple solution if the distance measure in question meets all other requirements. Other distance measures, such as KDiv, are relatively robust against white noise although they lack a sensitivity setting, and may be more appropriate if the noise is less uniform. A more drastic solution is to apply a smoothing algorithm as a pre‐processing step, though this should be approached with caution. Smoothing will remove noise and outliers but may distort the time series and increase bias in the process. Therefore, it is important to avoid over‐smoothing. Smoothing time series that have sudden and/or drastic value changes may also be problematic, particularly if these changes are an important aspect of differentiation between time series.

Our demonstration using wading bird trends from Jellesmark et al. (2021) served to illustrate both the potential benefits and complications introduced by smoothing. When we filtered by noise sensitivity, we were left with two distance measures; both returned the same results as the percentage difference calculations by Jellesmark et al. (2021). When we ran the method after applying a smoothing algorithm, we were left with a larger choice of seven distance measures. Although the ordering differed slightly from Jellesmark et al. (2021), all seven distance measures agreed with each other. The slight difference in ordering (Snipe vs Lapwing, ambiguous from visual inspection of the trends; Figure 7) is unsurprising given that the smoothing algorithm removed all noise from the trends, while the distance measures we selected using noise filtering, although demonstrating very low sensitivity to white noise, were not invariant to it. Smoothing in this case gave us more distance measures to choose from, but with the added complication of not knowing whether we had improved or distorted our results.

While in both cases (smoothed and unsmoothed trends), there were distance measures that gave the same rankings as Jellesmark et al. (2021) despite not matching our selection criteria (see Figures D1 and D2 in Appendix D), and the distance measures we selected were all in agreement. Had we been less specific when choosing important properties, we would have risked including measures that were not fit for purpose. A single suitable distance measure is better than any number of ill‐suited measures, and as with other statistics it is better to choose the measure up front, based upon justified criteria, rather than risk returning multiple results by choosing multiple measures and then cherry‐picking the result that is most convenient.

## 5 CONCLUSION

Our work should lead to an improved understanding of, and greater scope for, the use of distance measures for comparing time series within the field of ecology. Nonetheless, it is up to the user to think their way through the process. There are many scenarios/questions that would require the use of distance measures to compare time series in ecology, and we discuss only some of them here and cannot cover all potential issues that may arise in the process of metric selection. However, we hope to have opened the door for more ecologists to consider new questions where time series comparison is an important tool. Our framework can easily be adapted to incorporate other properties to select a distance measure that is appropriate for the task in question. There is not always a right choice of distance measure, but there are wrong ones, and our main goal is to help avoid those.

## AUTHOR CONTRIBUTIONS

**Shawn Dove:** Conceptualization (lead); data curation (lead); formal analysis (lead); investigation (lead); methodology (lead); project administration (lead); software (lead); visualization (lead); writing – original draft (lead); writing – review and editing (equal). **Monika Böhm:** Conceptualization (supporting); supervision (supporting); writing – review and editing (equal). **Robin Freeman:** Conceptualization (supporting); supervision (supporting); writing – review and editing (supporting). **Sean Jellesmark:** Data curation (supporting); investigation (supporting); writing – review and editing (supporting). **David J. Murrell:** Conceptualization (supporting); supervision (lead); writing – review and editing (equal).

## CONFLICT OF INTEREST STATEMENT

The authors have no conflict of interest to declare.

## Supporting information


Data S1:

## Data Availability

We used data from multiple sources, as well as simulated data, for this study. R scripts to recreate all simulated data and reproduce all results are available on GitHub at https://github.com/shawndove/Trend_compare. Wading bird indices produced from data provided by the RSPB and UK Breeding Bird Survey, as well as raw data from distance measure properties test results are archived at Dryad at https://doi.org/10.5061/dryad.bzkh189g7. Datasets from the UCR Time Series Classification Archive are available at https://www.cs.ucr.edu/~eamonn/time_series_data_2018/.

## APPENDIX A DISTANCE MEASURES

### Table of all tested distance measures

**TABLE A1:** Distance measures we tested (names are not necessarily given by the original authors, as some authors did not name their distance measures), with abbreviated names, type or family, category, important and/or interesting characteristics, and number of parameters that can or must be set, as well as sources for additional information.

Distance measure	Abbreviated name	Type/family	Category	Characteristics	Parameters	References
Euclidean distance^a^ ^,^ ^b^	Euclidean	Norm distance (*L*_p_ Minkowski family)	Lock‐step shape‐based	Shortest distance between points in Euclidean space	0	Cha (2007)
Manhattan distance^a^ ^,^ ^b^	Manhattan	Norm distance (*L*_p_ Minkowski family)	Lock‐step shape‐based	Shortest distance between points on a grid	0	Cha (2007)
Chebyshev distance^a^ ^,^ ^b^	Chebyshev	Norm distance (*L*_p_ Minkowski family)	Lock‐step shape‐based	Takes maximum distance between point pairs (all other point pairs are ignored)	0	Cha (2007)
Complexity‐invariant distance^a^	CID	Correction factor	Lock‐step shape‐based	Invariant to complexity	1	Batista et al. (2011)
Dynamic time warping distance^a^	DTW	Dog‐man Distance	Elastic shape‐based	Warping path	0–4	Sakoe and Chiba (1978)
Time alignment measurement distance^a^	TAM	Dog‐man Distance	Elastic shape‐based	Warping path Time distortion penalty	0	Folgado et al. (2018)
Normalized compression distance^a^	NCD	Compression distance	Compression‐based	Normalization of differences Quasi‐universality Choice of compression algorithm	1	Cilibrasi and Vitányi (2005)
Compression‐based dissimilarity measure^a^	CDM	Compression distance	Compression‐based	Compatible with symbolic representation Choice of compression algorithm	1	Keogh et al. (2004)
Edit distance with real penalty^a^	ERP	Edit distance	Elastic shape‐based	Gap‐length penalty	1–2	Chen and Ng (2004)
Edit distance on real sequences^a^	EDR	Edit distance	Elastic shape‐based	Threshold parameter Gap‐length penalty	1–2	Chen et al. (2005)
Fourier coefficient‐based distance^a^	Fourier		Feature‐based	Frequency domain	1	Agrawal et al. (1993)
Autocorrelation‐based dissimilarity^a^	ACF		Feature‐based	Compares autocorrelation coefficients	1–2	D'Urso and Maharaj (2009)
Partial autocorrelation‐based dissimilarity^a^	PACF		Feature‐based	Compares partial autocorrelation coefficients	1–2	Montero and Vilar (2014)
Periodogram‐based dissimilarity^a^	Per		Feature‐based	Frequency domain	2	(Caiado et al., 2006)
Integrated periodogram‐based dissimilarity^a^	IntPer		Feature‐based	Frequency domain	1	Casado de Lucas (2010)
Piccolo distance^a^	Piccolo		Model‐based	ARIMA models	0–3	Piccolo (1990)
Short time series distance^a^	STS		Lock‐step shape‐based	Captures temporal information	0–2	Möller‐Levet et al. (2003)
Dissimilarity index combining temporal correlation and raw value behaviour^a^	Cort	Correction factor	Lock‐step shape‐based and feature‐based	Temporal correlation coefficient Adaptive tuning function	2	Chouakria and Nagabhushan (2007)
Gower distance^b^	Gower	*L*_1_ family	Lock‐step shape‐based		0	Cha (2007)
Soergel distance^b^	Soergel	*L*_1_ family	Lock‐step shape‐based		0	Cha (2007)
Kulczynski distance^b^	Kulcz	*L*_*1*_ family	Lock‐step shape‐based		0	Cha (2007)
Canberra distance^b^	Canberra	*L*_1_ family	Lock‐step shape‐based	Normalizes	0	Cha (2007)
Lorentzian distance^b^	Lorentz	*L*_1_ family	Lock‐step shape‐based		1	Cha (2007)
Wave‐Hedges distance^b^	Wave	Intersection family	Lock‐step shape‐based	Normalizes	0	Cha (2007)
Czekanowski distance^b^	Czek	Intersection family	Lock‐step shape‐based	Normalizes	0	Cha (2007)
Jaccard distance^b^	Jaccard	Inner product family	Lock‐step shape‐based	Normalizes	0	Cha (2007)
Dice dissimilarity^b^	Dice	Inner product family	Lock‐step shape‐based	Normalizes	0	Cha (2007)
Squared‐Chord distance^b^	SqChord	Fidelity family	Lock‐step shape‐based		0	Cha (2007)
Squared Euclidean distance^b^	SqEuclid	Squared L_2_ family	Lock‐step shape‐based		0	Cha (2007)
Squared chi‐squared distance^b^	SqChi	Squared L_2_ family	Lock‐step shape‐based		0	Cha (2007)
Probabilistic symmetric chi‐squared distance^b^	ProbSymm	Squared L_2_ family	Lock‐step shape‐based		0	Cha (2007)
Divergence squared distance^b^	Diverge	Squared L_2_ family	Lock‐step shape‐based	Normalizes	0	Cha (2007)
Clark squared distance^b^	Clark	Squared L_2_ family	Lock‐step shape‐based	Normalizes	0	Cha (2007)
Additive symmetric chi‐squared distance^b^	Additive	Squared L_2_ family	Lock‐step shape‐based		0	Cha (2007)
Kullback–Leibler (KL) divergence^b^	Kullback	Shannon's entropy family	Lock‐step shape‐based	Non‐symmetric	1	Cha (2007)
Jeffreys divergence^b^	Jeffreys	Shannon's entropy family	Lock‐step shape‐based	Symmetric form of KL divergence	1	Cha (2007)
K divergence^b^	KDiv	Shannon's entropy family	Lock‐step shape‐based	Non‐symmetric	1	Cha (2007)
Topsoe distance^b^	Topsoe	Shannon's entropy family	Lock‐step shape‐based	Symmetric form of K divergence	1	Cha (2007)
Jensen difference^b^	Jensen	Shannon's entropy family	Lock‐step shape‐based		1	Cha (2007)
Taneja difference^b^	Taneja		Lock‐step shape‐based	Uses both arithmetic and geometric means	1	Cha (2007)
Kumar‐Johnson distance^b^	Kumar		Lock‐step shape‐based	Uses symmetric chi‐squared, arithmetic, and geometric means	0	Cha (2007)
Average (L_1_, L_inf_)^b^	AVG		Lock‐step shape‐based	Average of the Manhattan and Chebyshev distances	0	Cha (2007)

*Note*: Parameters are provided as a range if one or more parameters are optional (e.g., some can be set to “NULL,” while others are only sometimes relevant, depending on the input data). Note that we may list one or more parameters for distance measures that are considered parameter‐free if they require the user to choose a linked component, for example, a compression algorithm.

^a^ Available in the TSdist R package (Mori et al., 2016a, 2016b).

^b^ Available in the philentropy R package (Drost, 2018).

### Descriptions and formulas for selected distance measures

The *Euclidean distance* (Euclidean), also known as the *L* ^2^ distance, is the straight‐line distance between a pair of points. It also forms the basis for some of the more complicated transformation‐based and model‐based metrics presented here. It is defined as:

(A1)
$$d_{\mathrm{Euc}}=\sqrt{\sum_{i=1}^{d}{\left|P_{i}-Q_{i}\right|}^{2}}$$

where *P* and *Q* are (time series) vectors and *d* is the length of the vectors.

The *Manhattan distance* (Manhattan), or L^1^ distance, is the shortest distance between two points on a grid. Because it is not based on Euclidean geometry, there can be multiple paths with the same shortest distance. It is defined as:

(A2)
$$d_{\mathrm{CB}}=\;\sum_{i=1}^{d}\;|\;P_{i}-Q_{i}\;|$$

The *Chebyshev distance* (Chebyshev), or L^∞^ distance, is the greatest of the differences between two points or vectors along any coordinate dimension. For example, if two points had the *x*, *y* coordinates (0,0) and (3,5), the Chebyshev distance would be 5, the difference between the *y* coordinates of the two points, as this is greater than 3, the difference between the *x* coordinates. The Chebyshev distance is defined as:

(A3)
$$d_{\text{Cheb}}=\max_{i}\;|\;P_{i}-Q_{i}\;|$$

The *Complexity‐Invariant Distance* (CID) applies a complexity correction factor to the Euclidean distance to increase the dissimilarity value between time series with different complexities (where complexity is the length of a time series if stretched into a straight line—more and greater peaks and valleys means more complexity). It is defined as:

(A4)
$$d_{\mathrm{CID}}\left(X_{T},Y_{T}\right)=\mathrm{CF}\left(X_{T},Y_{T}\right)\cdot d\left(X_{T},Y_{T}\right)$$

where *d* is the Euclidean distance and CF is a complexity correction factor.

(A5)
$$\mathrm{CF}\left(X_{T},Y_{T}\right)=\frac{\max\left\{\mathrm{CE}\left(X_{T}\right),\mathrm{CE}\left(Y_{T}\right)\right\}}{\min\left\{\mathrm{CE}\left(X_{T}\right),\mathrm{CE}\left(Y_{T}\right)\right\}}$$

where CE is a complexity estimator.

(A6)
$$\mathrm{CE}\left(X_{T}\right)=\sqrt{\sum_{t=1}^{T-1}{\left(X_{t}-X_{t+1}\right)}^{2}}$$

The *Dynamic Time Warping distance* (DTW) computes a warping path between two time series to align them in time. It can be defined as a "dog‐man" distance (a distance that determines the shortest leash length between a person and their dog walking separate paths), but instead of the shortest leash length, it measures the average leash length. This makes it more robust, as it is less sensitive to outliers and short divergences. The DTW distance is defined as:

(A7)
$$d_{\mathrm{DTW}}\left(X_{T},Y_{T}\right)=\min_{r\in M}\left(\sum_{i=1,\ldots ,m}|X_{a_{i}}-Y_{b_{i}}|\right)$$

The *Time Alignment Measurement distance* (TAM) is a derivative of the DTW distance that measures how well two time series align in time. Segments not in phase are penalized, while amplitude differences are not. A dissimilarity value of zero can occur for non‐identical series that are perfectly aligned in time.

The *Normalized Compression Distance* (NCD) is based on the concept of Kolmogorov complexity, which is the minimum information needed to generate a string using an algorithm. The Kolmogorov complexity is a measure of randomness of the string. The smaller the value, the less randomness. The NCD applies the concept to a relationship between objects (time series) when a compression algorithm is applied. The greater the advantage in compression (reduction in randomness) gained by multiplying two time series together, the more closely they are related, and therefore the smaller the dissimilarity between them. NCD is defined as:

(A8)
$$d_{\mathrm{NCD}}\left(X_{T},Y_{T}\right)=\frac{C\left(X_{T}Y_{T}\right)-\min\left\{C\left(X_{T}\right),C\left(Y_{T}\right)\right\}}{\max\left\{C\left(X_{T}\right),C\left(Y_{T}\right)\right\}}$$

where *C* represents the compressed size.

The *Compression‐based Dissimilarity Measure* (CDM) is a simplified version of the NCD, defined as:

(A9)
$$d_{\mathrm{CDM}}\left(X_{T},Y_{T}\right)=\frac{C\left(X_{T}Y_{T}\right)}{C\left(X_{T}\right)C\left(Y_{T}\right)}$$

The *Edit Distance with Real Penalty* (ERP) is an edit distance, meaning it quantifies the number of insert, delete, or replace operations required to turn one string (time series) into another. ERP includes a penalty for gaps between matched substrings based on the gap length.

The *Edit Distance for Real Sequences* (EDR) is an edit distance refined for trajectories. It includes a quantization feature as well as the length‐based gap penalty of ERP.

The *Fourier Coefficient‐based distance* (Fourier) calculates the Euclidean distance between discrete Fourier transforms of a pair of time series. Fourier transforms extract frequency information by decomposing a signal (time series) into its frequency components (sine and cosine functions). While a time series is visualized as a single graph of amplitude vs time, its Fourier transform consists of multiple sinusoidal waves, each with a specific, constant amplitude and frequency. Time information is lost. The Fourier transform works well for stationary time series, as they have periodic repeating signals. However, the loss of time information presents problems for deconstructing non‐stationary series, as they change randomly over time.

The *Autocorrelation‐based dissimilarity* (ACF) calculates the Euclidean distance between estimated autocorrelation functions of time series. An autocorrelation function of a time series describes the correlation between two values of the time series at different times with a specified lag (delay between the two values). In other words, it describes the correlation of a time series with a time‐offset version of itself. It is defined as:

(A10)
$$d_{\mathrm{ACF}}\left(X_{T},Y_{T}\right)=\sqrt{{\left({\hat{\rho }}_{X_{T}}-{\hat{\rho }}_{Y_{T}}\right)}^{T}\Omega \left({\hat{\rho }}_{X_{T}}-{\hat{\rho }}_{Y_{T}}\right)}$$

where Ω is a matrix of weights and *ρ‐hat* refers to estimated autocorrelation vectors.

The *Partial Autocorrelation‐based dissimilarity* (PACF) is identical to the ACF except that it uses the partial autocorrelation functions.

The *Periodogram‐based dissimilarity* (Per) calculates the Euclidean distance between the periodograms of time series. A periodogram is a method of estimating the power spectrum of a time series, which is equivalent to the Fourier transform of the autocorrelation function. It describes how power is distributed over the frequency components of a time series.

The *Piccolo distance* (Piccolo) calculates the Euclidean distance between the AR(∞) operators, or autoregressive expansions, of invertible ARIMA models of time series. ARIMA is a time series forecasting method. ARIMA models work by describing autoregressive (AR) and moving average (MA) parameters. An autoregressive model explains a value in a time series by one or more previous values plus random error. It is generally written as AR(p), where p is the order of the model. An autoregressive expansion, AR(∞), is thus an AR model of infinite order. A moving average model—written as MA(q), where q is the order—explains a value in a time series by one or more past random errors as well as its own random error term. Invertible ARIMA models are those which can be written simply as autoregressive (AR) models. This is a necessary property to be able to forecast the dependent variable and is important for the Piccolo distance, since only the AR aspect is used. ARIMA models can be applied to non‐stationary time series, but they must first be converted to stationary time series by one or more differencing operations (subtracting each value from the one before it to remove stochastic trends).

The *Short Time Series distance* (STS) measures the difference between the slopes of time series defined as piecewise linear functions. It is intended to incorporate temporal information while ignoring absolute values, to overcome a weakness of many other distances, including the Euclidean distance, which ignore the temporal order of points and the length of sampling intervals. The STS distance is defined as:

(A11)
$$d_{\mathrm{STS}}\left(X,Y\right)=\sqrt{\sum_{k=0}^{N-1}\left(\frac{y_{k+1}-y_{k}}{t_{k+1}-t_{k}}-\frac{x_{k+1}-x_{k}}{t_{k+1}^{\prime }-t_{k}^{\prime }}\right)}$$

Equations (A1), (A2), (A3) are copied from Cha (2007), equations (A4), (A5), (A6), (A7), (A8), (A9), (A10) are copied from Montero and Vilar (2014), and equation A11 is copied from Mori et al. (2016a, 2016b).

### Distance measure properties

Here, we have included additional explanation for some properties of distance measures.

*Translation sensitivity*: Translation sensitivity is adapted from translation invariance, *d*(*X* + *q*, *Y*) = *d*(*X*, *Y*), where *q* is any real number. Translation invariance is a shape‐preserving property, meaning that a distance measure with this property would treat two time series with identical shapes as equal, even if the mean values were different. However, we can also define translation *sensitivity*, where the dissimilarity between *X* and *Y* increases relative to the value of *q*, and translation *insensitivity*, where the dissimilarity between *X* and *Y* increases by an amount that is independent of *q*. Translation invariance is a special case of translation insensitivity, where *d*(*X* + *q*, *Y*) is independent of *q*. Translation sensitivity can be measured in relative terms, allowing comparison between distance measures.

The effect of translation invariance can be achieved by a vertical shift transformation. For example, time series *X* can be transformed by adding the same real number *q* to each observation, *f*(*X*) = *X* + *q*, such that time series *X* and *Y* have the same starting value (if they already have the same starting value there is no need for transformation). It is a simple matter to apply this transformation to thousands of time series. Note, however, that translation invariance can be problematic. Consider two populations, with population A having a starting size of 100 and population B a starting size of 10,000. If both populations increase by 10 every year for 10 years, population A would now be 200, which means it doubled to twice its original size, while population B would be 10,100, an increase of only 1%. A distance measure with the property of translation invariance (or any distance measure after applying a vertical shift transformation to equalize the starting values) would treat these trends as equal.

An alternative way to deal with such comparisons would be a scale transformation, *f*(*X*) = *X* × *q*, multiplying each observation of time series *x* by the same real number *q*, such that time series *X* and *Y* have the same starting value. A scale transformation allows for shape deformation while preserving percentage change. If populations A and B both doubled by increasing linearly for 10 years from 100 to 200 and 10,000 to 20,000 respectively, a scale transformation would result in identical trends, although they did not originally have the same shape. Likewise, in the previous example where two populations of different sizes increase by the same amount but different percentages, a scale transformation would result in trends with very different shapes (slopes). Scale invariance, which is defined as *d*(*X* × *q*, *Y*) = *d*(*X*, *Y*), with *q* greater than 0 (Batyrshin et al., 2016), is a rare property of distance measures.

*Amplitude sensitivity*: If a vertical shift transformation, *f*(*t*) = *t* + *q*, is applied to one or more observations *t* of time series *X* to form time series *Y*, *d*(*X*, *Y*) > *d*(*X*, *X*) and *d*(*X*, *Y*) increases with *q* (sensitivity). Amplitude sensitivity is particularly relevant when comparing time series which have been scale transformed or vertical shift transformed to have the same starting value. But some distance measures, especially among edit distances, are insensitive to amplitude. For example, the Edit Distance for Real Sequences (EDR) has a threshold value that can be set. Only differences that exceed the threshold are counted.

*White noise sensitivity*: White noise sensitivity is adapted from white noise invariance (invariance against random noise), *d*(*X* + *f*(*X*), *Y*) ≈ *d*(*X*, *Y*), where *f*(*X*) is a function that adds a small pseudo‐random value from a normal distribution with a mean of zero and standard deviation *q* to each observation of time series *X* (adapted from Lhermitte et al., 2011). Adding a random noise term to one time series from a pair should have an inconsequential effect on the dissimilarity value between them. A distance measure sensitive to white noise will show an increase in dissimilarity values relative to *q*, allowing us to obtain a relative measure of robustness against white noise.

*Biased noise sensitivity*: Biased noise sensitivity is adapted from biased noise invariance (invariance against non‐random noise, i.e., noise in a single direction), *d*(*X* + *f*(*X*), *Y*) ≈ *d*(*X*, *Y*), where *f*(*X*) is a function that adds a small non‐random value *q* to some of the observations (randomly chosen) of time series *X* (adapted from Lhermitte et al., 2011).

*Outlier sensitivity*: Outlier sensitivity is adapted from outlier invariance, *d*(*X* + *f*(*X*), *Y*) ≈ *d*(*X*, *Y*), where *f*(*X*) is a function that adds a large pseudo‐random value *q* to a single randomly chosen observation of time series *X*. Outlier sensitivity is thus defined as the dissimilarity value increasing with *q*, and is a specific case of amplitude sensitivity limited to a single time point.

*Antiparallelism bias*: Mathematically, if *Y* = *f*(*X*), where *f*(*X*) is a function that reflects *X* across the axis of *t*_0_ (for all *t* in *X*, $Y_{t_{i}}=2X_{t_{0}}-X_{t_{i}}$), and *Z* = *g*(*X*), where *g*(*X*) is a function that applies a scale transformation to *X* relative to *t*_0_ such that the absolute difference in summed values between *Z* and *X* is the same as that between *Y* and *X* (for all *t* in *X*, $Z_{t_{i}}=3X_{t_{i}}–2X_{t_{0}}$), then *d*(*X*,*Z*) > *d*(*X*,*Y*) for positively biased distance measures; *d*(*X*,*Z*) < *d*(*X*,*Y*) for negatively biased measures; and *d*(*X*,*Z*) = *d*(*X*,*Y*) for neutral measures.

*Phase sensitivity*: Phase sensitivity is adapted from phase invariance, *d*(*X*_*i* + *p*_, *Y*_*i*_) = *d*(*X*_*i*_, *Y*_*i*_) (adapted from Lhermitte et al., 2011).

*Time scaling sensitivity*: Time scaling sensitivity is adapted from time scaling invariance, *d*(*X*_*pi*_, *Y*_*i*_) = *d*(*X*_*i*_, *Y*_*i*_) (adapted from Esling & Agon, 2012). If one time series is expanded or compressed along its time axis, the dissimilarity value should not change.

*Warping sensitivity*: Time scaling invariance can be defined locally, that is, involving the expansion or compression of one or more sections of a time series, rather than the entire series (Batista et al., 2011). If a function *f*(*S*_*i*_) = *S*_*pi*_ is applied to expand or compress *S*, where *S* is any subset of *X*, *S* ⊆ *X*, to form time series *Y*, then *d*(*X*,*X*) = *d*(*X*,*Y*). Warping sensitivity thus occurs when the dissimilarity value increases with *p*.

*Frequency sensitivity*: If time series *Y* is obtained by applying the same transformation *f*(*t*) to one or more observations *t* of time series *X*, such that *d*(*X*, *Y*) > *d*(*X*, *X*), then the dissimilarity value will depend on the number of observations to which the transformation f(t) is applied.

*Duration sensitivity*: If time series *Y* is obtained by applying the same transformation *f*(*t*) to one or more consecutive observations of time series *X*, such that *d*(*X*, *Y*) > *d*(*X*, *X*), then the dissimilarity value will depend on the number of consecutive observations to which the transformation *f*(*t*) is applied. This property is a special case of frequency sensitivity. Distance measures which are sensitive to duration must be sensitive to frequency, but the converse is not true. Some distance measures, such as Dynamic Time Warping (DTW) or the Short Time Series Distance (STS), may rank time series with more differences as more dissimilar *only if those differences are separated by similarities*. Consider a time series *A*, with five points, *t*_0_, *t*_1_,…, *t*_4_. Some transformation *f*(*t*) is applied only to point *t*_1_ to form time series *B* (*B* thus differs from *A* by a single point), to both *t*_1_ and *t*_2_ to form time series *C*, and to both *t*_1_ and *t*_3_ to form time series *D* (thus *C* and *D* each differ from *A* by the same value at two points, but in *C* those points are consecutive while in *D* they are not). For distance measures which are sensitive to *both* frequency and duration, *d*(*D*, *A*) > *d*(*C*, *A*) > *d*(*B*, *A*), but for distance measures which are sensitive to frequency but *not* duration, *d*(*D*, *A*) > *d*(*B*, *A*), while *d*(*C*, *A*) = *d*(*B*, *A*). This is because a distance that is invariant to duration will treat a difference that occurs over multiple consecutive time points as a single difference.

## APPENDIX B TESTING METHODOLOGY

### Metric testing

The test for reflexivity was conducted by comparing a time series first to itself, and then to a similar time series with a value difference at a single point. For distance measures with threshold settings (e.g., EDR), we set the threshold to zero to ensure they would recognize the difference. Any distance measure that returned a value of zero when comparing the time series against itself, and any non‐zero value when comparing it against a time series with a value difference at a single point, was considered to demonstrate reflexivity.

Symmetry was tested by comparing a pair of different time series, *X* and *Y*, in both forward order, *d*(*X*, *Y*), and reverse order, *d*(*Y*, *X*). If the two values returned were identical, the distance measure was considered to demonstrate symmetry. We ensured that the time series were different enough that no distance measure returned 0 for both forward and reverse order.

The triangle inequality and non‐negativity properties were tested by comparing thousands of short, randomized time series generated by a stochastic exponential model. We found that shorter time series were better at detecting violations, so we set the length to five. We generated 300,000 time series and divided them into 100,000 sets of three. Within each set of three, we considered each time series to represent one corner of a triangle and compared them pairwise, with the resulting distances representing the sides of the triangle. We then subtracted the two shorter sides from the longest side. If the difference was greater than zero for any of the 100,000 sets, then the distance measure was considered to violate the triangle inequality. Additionally, if any of the 300,000 time series comparisons produced a negative value, the distance measure was considered to violate non‐negativity. We set the time series generator such that zeros and negative values were included in some time series, as some distance measures satisfy the triangle inequality and/or non‐negativity only when all input values are positive or non‐negative.

Distance measures were classified as “full” for full metric if they passed all metric tests, “semi” for semi‐metric if they passed all tests except the triangle inequality, or “non” for non‐metric if they failed one or more of the other tests.

Settings for adaptive distance measures (distance measures with settings that can be changed to alter their behavior) were set at defaults given in examples from the documentation of the TSdist R package (Mori et al., 2016a, 2016b). For triangle inequality and non‐negativity tests, we kept the same settings for initial testing. If they passed the tests at those settings, we tested them over a range of settings. If they failed at default settings, there was no need for further testing.

### Controlled testing

We used the Manhattan distance as a basis for devising controlled sensitivity tests for translation, amplitude, duration, frequency, white noise, biased noise, and outliers. The Manhattan distance is the summed absolute difference between each pair of points in a time series. It is a simple‐to‐calculate metric and demonstrates all the sensitivities we tested for. Furthermore, it responds to sensitivity tests in a linear manner. These properties make the Manhattan distance an ideal basis for comparison of other distance measures.

For each sensitivity test, we constructed a series of n time series with linearly increasing differences, *T*_1_, *T*_2_,…, *T*_*n*_, such that the differences in absolute value between point pairs of any consecutive pair of time series summed to 1. Thus, the Manhattan distance between any pair of consecutive time series, *T*_*i*_ and *T*_*i* + 1_, was 1, and between any non‐consecutive pair of time series, *T*_*i*_ and *T*_*i* + j_, is j. For example, the Manhattan distance between *T*_1_ and *T*_2_ would be 1, between *T*_2_ and *T*_3_ would be 1, and between *T*_1_ and *T*_5_ would be 4.

Sensitivity tests were conducted for each distance measure by comparing each time series *T*_*i*_ in the set *T*_1_, *T*_2_,…, *T*_*n*,_ to *T*_1_. Any distance measure returning a dissimilarity value of 0 for *every* pair of time series for a given sensitivity test would be considered as invariant for that property, while a distance measure returning the same *non‐zero* value for *every* time series pair would be considered as insensitive (note that invariance implies insensitivity, but insensitivity is not the same as invariance. Distance measures that demonstrate insensitivity to a property register differences as binary—different or not different—while those demonstrating invariance do not register differences at all).

Sensitivity is calculated as the mean of all distances between consecutive time series,

(B1)
$$s=\frac{\sum_{i=1}^{n-1}d\left(T_{i},T_{i+1}\right)}{n},$$

where *s* is sensitivity, *d*(*T*_*i*_, *T*_*i* + 1_) is the distance between a pair of consecutive time series *T*_*i*_ and *T*_*i* + 1_, and n is the total number of time series being compared.

Given that *s* is an absolute sensitivity value, its interpretation is dependent on the scale of the distance measure. A scale‐independent relative sensitivity is obtained by

(B2)
$$rs_{x}=\frac{s_{x}}{s_{\mu }},$$

where *rs*_*x*_ is the relative sensitivity to property *x*, *s*_*x*_ is the absolute sensitivity to that property, and *s*_μ_ is the mean of absolute sensitivities to all tested properties.

The sensitivity values for all distance measures are separated into five bins and designated as “very low,” “low,” “medium,” “high,” or “very high.” The sensitivity value for the Manhattan distance is 1 for every property and serves as the median value for the bins, which are: less than 0.2, 0.2 to 0.75, 0.75 to 1.25, 1.25 to 2.5, and greater than 2.5, respectively. Note, however, that the equation for sensitivity is derived from the linear slope equation, but the sensitivity for many distance measures is non‐linear. The calculated sensitivity is a linear approximation along the tested range.

Phase sensitivity testing was conducted in a similar way to sensitivity testing, with *T*_*1*_, *T*_*2*_,…, *T*_*n*_ representing a set of time series, with the difference in phase increasing with *i* in *T*_*i*_. However, the Manhattan distance could not be used as a basis for comparison. This is because lock‐step distance measures (those that match every time point 1‐to‐1), including the Manhattan distance, do not respond to time translation in a way that can be interpreted by a function. Distance measures were designated as “inv” (meaning they demonstrated phase invariance) when the dissimilarity between *every* pair of time series was 0, “ins” (insensitive) when *every* pair of time series returned the same *non‐zero* dissimilarity value, “sens” (sensitive) when the dissimilarity value was dependent on *i*, or “unp” (unpredictable) when dissimilarity values differed but did not depend on *i*. For those distances with window size settings (e.g., some distance measures that act stepwise along time series have a setting to control how many time points are considered in each step), we set the window large enough to cover the maximum difference in phase (that between *T*_1_ and *T*_*n*_).

Time scaling sensitivity was tested using a set of time series in which *T*_*i* + 1_ was stretched compared with *T*_*i*_. This involved lengthening the time series *T*_*i*_, keeping the first and last time points the same while altering the values at each time point in between to fit the shape change. Warping was tested by stretching only one horizontal section of a time series, such that a set was formed, with *T*_*i* + 1_ longer than *T*_*i*_. As with phase sensitivity, results for time scaling sensitivity and warping sensitivity were not compared against the Manhattan distance, as the Manhattan distance and other lock‐step distance measures are unable to handle time series with different lengths. Elastic distance measures were tested and designated as either “inv” (invariant) if *all* returned dissimilarities were zero, “ins” (insensitive) if *all* returned dissimilarities were *identical* and *non‐zero*, “sens” (sensitive) if the returned value depended on the degree of time scaling or warping, or “unp” (unpredictable) if returned values differed but did not depend on the degree of time scaling or warping. All lock‐step distance measures were designated as “n/a.”

Antiparallelism bias was tested by comparing pairs of time series that differed by the same relative amount in different directions. Distance measures were designated as having “positive” bias if they gave a greater dissimilarity value to pairs of time series differing in opposite directions than to pairs differing in the same direction, “negative” bias if they gave a greater dissimilarity value to those differing in the same direction, or “neutral” if they assigned each pair of time series the same dissimilarity value.

### Uncontrolled testing

We created a function for each property to be tested, which applies a transformation to one or more time points of a real‐world time series given as input. Each function accepts a value *q*, the purpose of which varies depending on the function (see below for details). For example, the translation function adds a real number *q* to every value *t*_*i*_ of a time series *T*. The transformed time series is returned as output and compared against its unaltered counterpart. We applied the functions to a range of *q* in increments, then graphed the results as response curves (see Figures S5–S8). We did not compare them against a reference or assign sensitivity ratings, as they were intended only as a confirmatory check against the results of controlled testing.

#### Functions

Translation sensitivity: Add *q* to every data point of a time series *T*.

White noise sensitivity: Create a normal distribution with mean *q* and standard deviation 0.3 times *q* (the latter is arbitrary). Randomly select half of the data points of a time series *T* and add randomly selected values from the normal distribution to the selected points. Finally, subtract randomly selected values from the normal distribution from the points that were not selected. This function scales *q* by $\frac{q}{\max\left(q\right)}$ to avoid the noise being too large.

Biased noise sensitivity: Proceed exactly as with white noise sensitivity but skip the final step (the points that were not selected remain untransformed). This function scales *q* by $\frac{q}{0.5\times \max\left(q\right)}$ (the 0.5 is because the function is only applied to half of the time points).

Outlier sensitivity: Add *q* to one randomly selected point of a time series *T* (excluding the first and final points, which can cause unintended behavior in some distance measures).

Phase invariance: Shift the first *q* time points of a time series *T* to the end of the time series.

Warping sensitivity: Randomly select a single value from a time series *T* and extend the time series by repeating the chosen value *q* times.

Uniform time scaling sensitivity: Stretch a time series *T* along the *x*‐axis by a factor of *q*. The *y*‐axis values of the first and final points remain unchanged, but the final point is shifted along the *x*‐axis and all points in between are recomputed. For this function, *q* is scaled: $\frac{q}{\max\left(q\right)}+1$. Thus, time series *T* will be stretched to a maximum of twice its original length.

## APPENDIX C TEST RESULTS

### Metric test results

In some cases, results depended on input values or settings. Eight of the lock‐step shape‐based distance measures passed the triangle inequality test and/or non‐negativity test when inputs were constrained to non‐negative real numbers, but failed when negative numbers were included. EDR behaved as a metric when the threshold setting, ε, was set near zero, but failed the triangle inequality test when ε was set at five. The Normalized Compression Distance (NCD) and the Compression‐based Dissimilarity Measure (CDM) both failed our reflexivity and symmetry tests and thus qualified as non‐metrics, although NCD is stated by its authors to be a metric (Cilibrasi & Vitányi, 2005). However, this is qualified with respect to the compression algorithm paired with it, with none quite reaching the definition the metric behavior depends on. NCD should approach closer to true metric behavior the longer the time series (Cilibrasi & Vitányi, 2005). We tested it here with very short time series, and therefore it would not be expected to behave as a metric. Additional testing (not included) showed NCD came closer to passing the reflexivity and symmetry tests, although as the time series reached a length of 1 million, it was still failing. Beyond that length, running the tests was too slow to be practical. CDM, on the other hand, is not considered to be a metric (Keogh et al., 2004), nor did it approach closer to metric behavior when tested with longer time series.

### Controlled test results

#### Sensitivity tests

Results for EDR depended on the value of the threshold setting, ε. We reported the results with ε at 0.1. However, when ε was set high, EDR was invariant to all seven of these properties. When ε was set within the range of the input values, results were less predictable.

The two compression‐based distances we tested, the Normalized Compression Distance (NCD) and the Compression‐based Dissimilarity Measure (CDM), showed insensitivity to translation and outliers. However, our uncontrolled test results did not confirm this. It is not clear why this difference occurred, but keep in mind that compression‐based distances may behave differently for short time series than for long ones (e.g., they do not behave as metrics when comparing short time series).

#### Time‐based sensitivities and other tests

Again, results for EDR depended on the value of ε. We reported the results with ε at 0.1, but when ε was set high, EDR was invariant to phase, and sensitive to both warping and time scaling. When ε was set within the range of the input values, it responded unpredictably to phase shift, but remained sensitive to both warping and time scaling.

The results for the Autocorrelation‐based dissimilarity (ACF) and the Partial Autocorrelation‐based dissimilarity (PACF) were “n/a” for both warping and time scaling, suggesting that these distance measures are unable to deal with unequal‐length time series. However, this is not the case. The problem is that these measures require an equal number of autocorrelation coefficients, which the short time series we used for controlled testing did not satisfy. However, ACF and PACF did provide results for warping and time scaling in uncontrolled testing (Figures C2 and C3).

### Uncontrolled test results

Figures C2, C3, C4, C5 show the results of uncontrolled testing of distance measure properties using two real‐world time series (Figure 3) from the UCR Time Series Classification Archive (Dau et al., 2019), an archive of 128 time series datasets intended for testing of classification algorithms. All dissimilarity values in the test results have been rescaled to a range of [0,1] using Min–Max scaling. This was done to facilitate placing response curves for different types of transformations on the same plot while still allowing the shape of each response curve to be seen regardless of the strength of the response. For controlled testing, time series were carefully constructed to allow comparison of response strength across different properties. That is a far more difficult problem when working with real‐world time series, so we opted instead to exchange strength information for better shape resolution.

For those distance measures that are sensitive to a tested property, the dissimilarity value shows a response curve as the size of the transformation value *q* increases. The sensitivity response curve may be linear or not but should be described by a function. Invariances show as horizontal lines at a dissimilarity value of zero, while insensitivities show as horizontal lines at some non‐zero value. The response curves for some properties differ in shape between time series, especially for elastic distance measures and those designed for stationary time series. Despite this, results are largely consistent with the controlled testing results shown in Figures 5 and 6.

There are a few exceptions, however. Both compression‐based distances we tested, NCD and CDM, registered as insensitive to translation and outliers in controlled testing, while showing unpredictability in uncontrolled testing. Two feature‐based distances, ACF and PACF, showed unpredictability for warping sensitivity and uniform time scaling sensitivity in uncontrolled testing but failed to give results in controlled testing. This was because these distance measures require the time series being compared with have an equal number of autocorrelation coefficients, a requirement which was met when extending the real‐world time series, but not when extending the short time series that we created for controlled testing. Finally, the Time Alignment Measurement distance, TAM, showed unpredictability to outliers in controlled testing, but was insensitive in uncontrolled testing. The raw dissimilarity values from the controlled testing showed a sudden increase from a dissimilarity value of 0 to 0.33 as the value of *q* increased from 2 to 3. Given that we used the same starting value of *q* (1) and the same increment size (also 1) for both controlled and uncontrolled testing, the threshold is presumably not determined simply by the value of the outlier, *q*, but by a more complex calculation.

## APPENDIX D EXAMPLE DATASET RESULTS

### D.1

When comparing unsmoothed time series, the two selected distance measures gave identical results to percent improvement from Jellesmark et al. (2021) (Figure D1). Among the 40 unselected distance measures, only two, EDR and TAM, gave the same rankings as the selected distance measures and percent improvement (Figure D1). Another 30 agreed with Jellesmark et al. (2021) in ranking Redshank first, but beyond that the results differed strongly, with 28 ranking Yellow Wagtail second and 23 ranking Curlew last (Figure D1). None of the distance measures returned the same results as the *t*‐test.

In the smoothed time series comparison, all seven of the selected distance measures agreed with each other but differed slightly from the percent improvement results of Jellesmark et al. (2021) by placing Snipe ahead of Lapwing (Figure D2). Of the 35 unselected distance measure, four gave the same rankings as the selected distance measures, while 11 agreed with percent improvement and five agreed with the *t*‐test (Figure D2).

## APPENDIX E SPEEDING UP DTW

### E.1

For matching problems, such as content queries and classification, the slowness of DTW can be avoided by indexing, which severely reduces the number of time series that need to be compared with find the best match. For the Euclidean distance, indexing is relatively straightforward to accomplish. However, as DTW does not satisfy the triangle inequality (Figure 4), it presents more of a challenge. Keogh and Ratanamahatana (2005) solved this problem using a tight “lower‐bounding” measure, which is included in the TSdist package (Mori et al., 2016a, 2016b) as LBKeoghDistance. For an explanation of lower bounding and the indexing process with respect to DTW, refer to Keogh and Ratanamahatana (2005). The lower‐bounding technique does not apply to clustering, where some real‐world problems can take weeks or even months (Zhu et al., 2012). However, Zhu et al. (2012) solved this problem for clustering by creating an interactive “anytime algorithm”, which uses a fast approximation of DTW to give a best available answer that improves over time as exact DTW calculations are performed, and can be paused or terminated at any time.
