# Supplementary material for: A user‐friendly guide to using distance measures to compare time series in ecology
Source: Ecol Evol. 2023 Oct 5;13(10):e10520. doi: 10.1002/ece3.10520 (PMC10551742; doi:10.1002/ece3.10520)
Supplement: Supplementary file 1 — Data S1: [file ECE3-13-e10520-s001.pdf]

Contents

1. Plots of controlled test results for all distance measures 2
2. Tables of controlled test results for all distance measures 44
3. Plots of wading bird rankings for all distance measures 86

1. Plots of controlled test results for all distance measures

This section contains plots of controlled testing results for all 42 distance measures we tested. Each figure includes all time-based and values-based properties for which that distance measure gave results. Distance measures are presented in alphabetical order.

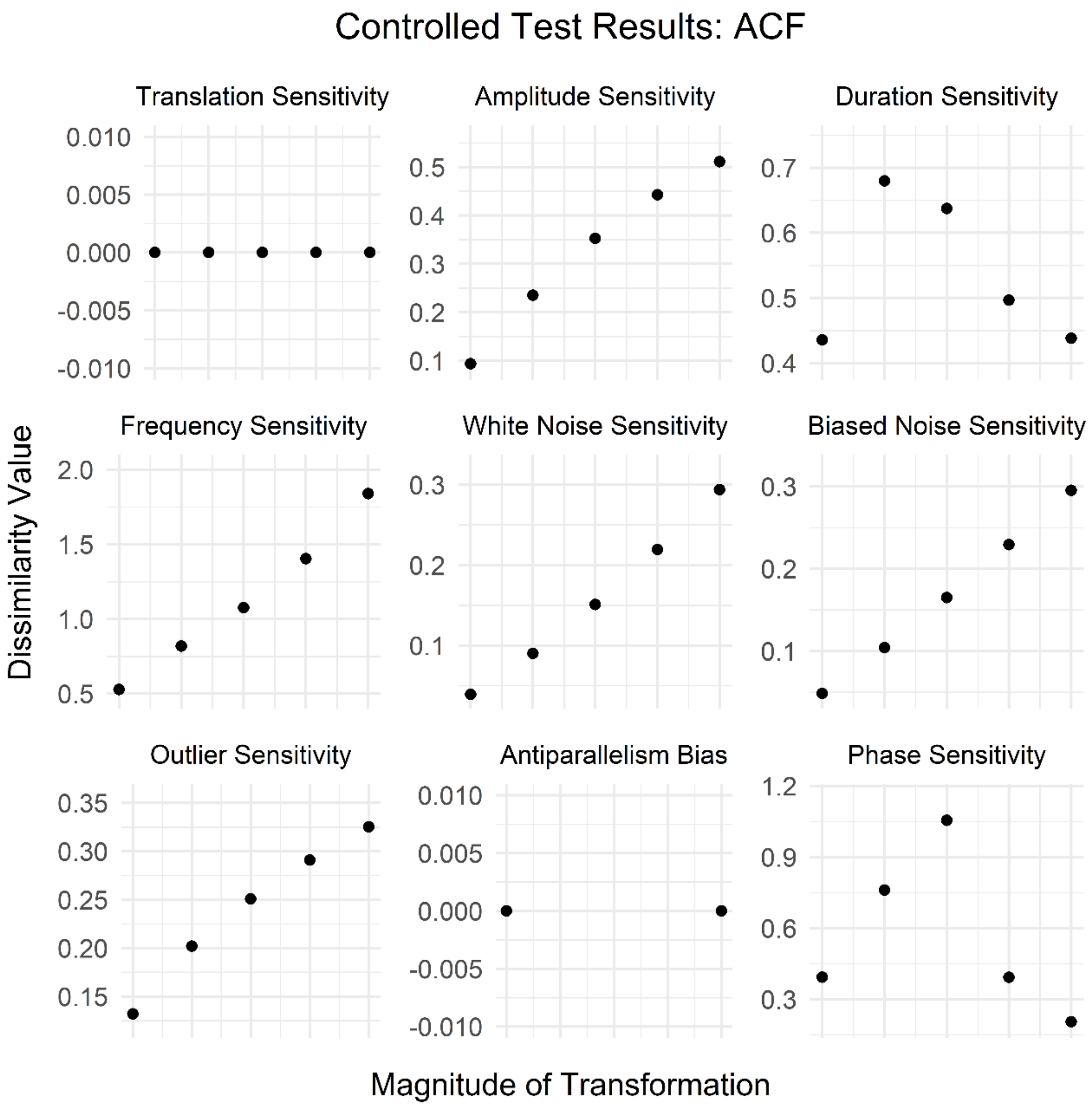

Fig. S1. Controlled testing results for the Autocorrelation-Based Dissimilarity. Sensitivities were tested by comparing time series with linearly increasing differences in summed y-axis values (or phase), against a reference time series. Antiparallelism bias was tested by comparing pairs of time series that differed by the same relative amount in different directions. The bias is neutral if the two values are identical, negative if the value on the left is higher, and positive if the value on the right is higher. Uniform time scaling sensitivity and warping sensitivity could not be tested for this distance measure, as it cannot measure unequal-length time series.

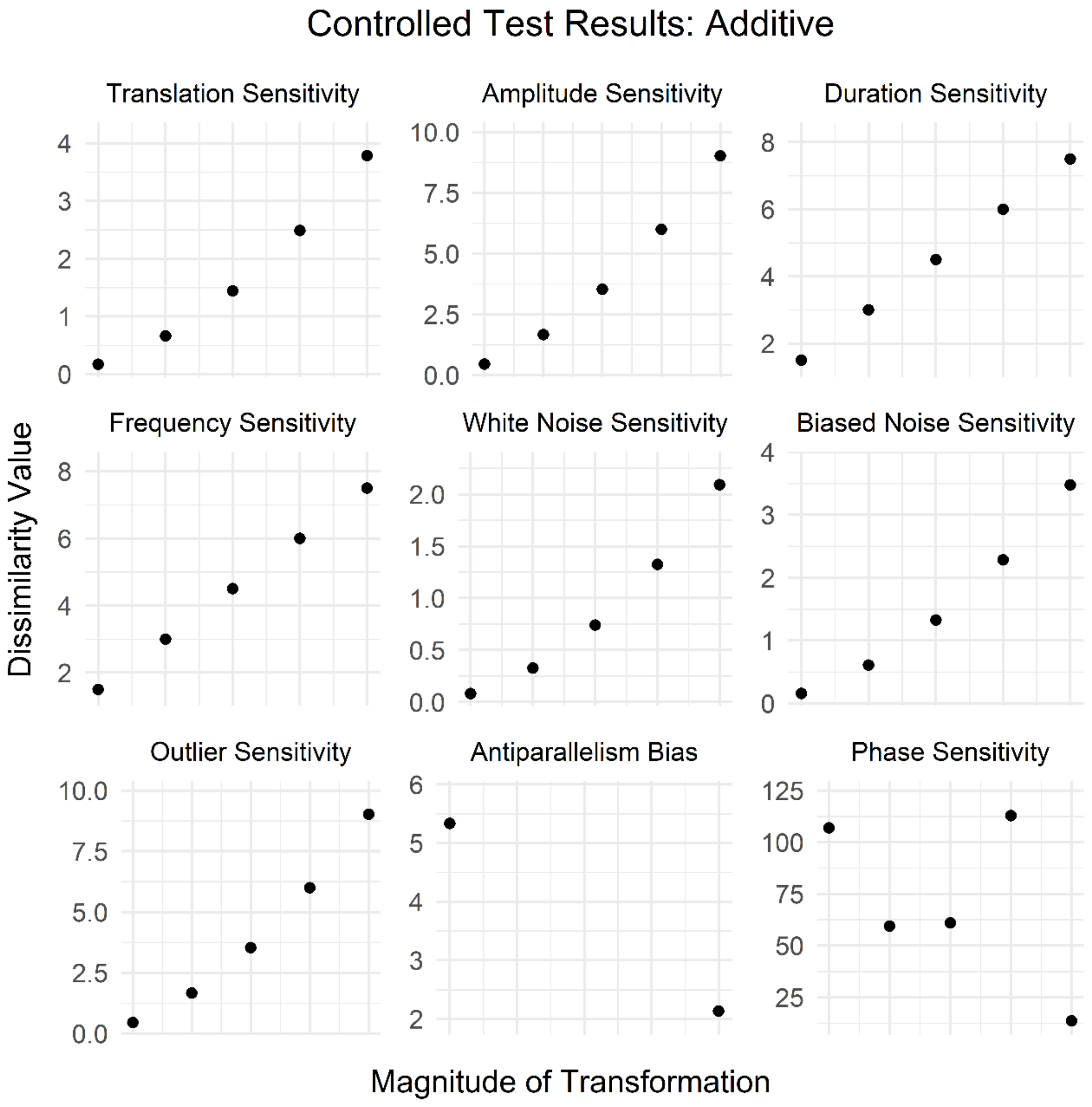

Fig. S2. Controlled testing results for the Additive Symmetric Chi-Squared Distance. Sensitivities were tested by comparing time series with linearly increasing differences in summed y-axis values (or phase), against a reference time series. Antiparallelism bias was tested by comparing pairs of time series that differed by the same relative amount in different directions. The bias is neutral if the two values are identical, negative if the value on the left is higher, and positive if the value on the right is higher. Uniform time scaling sensitivity and warping sensitivity could not be tested for this distance measure, as it cannot measure unequal-length time series.

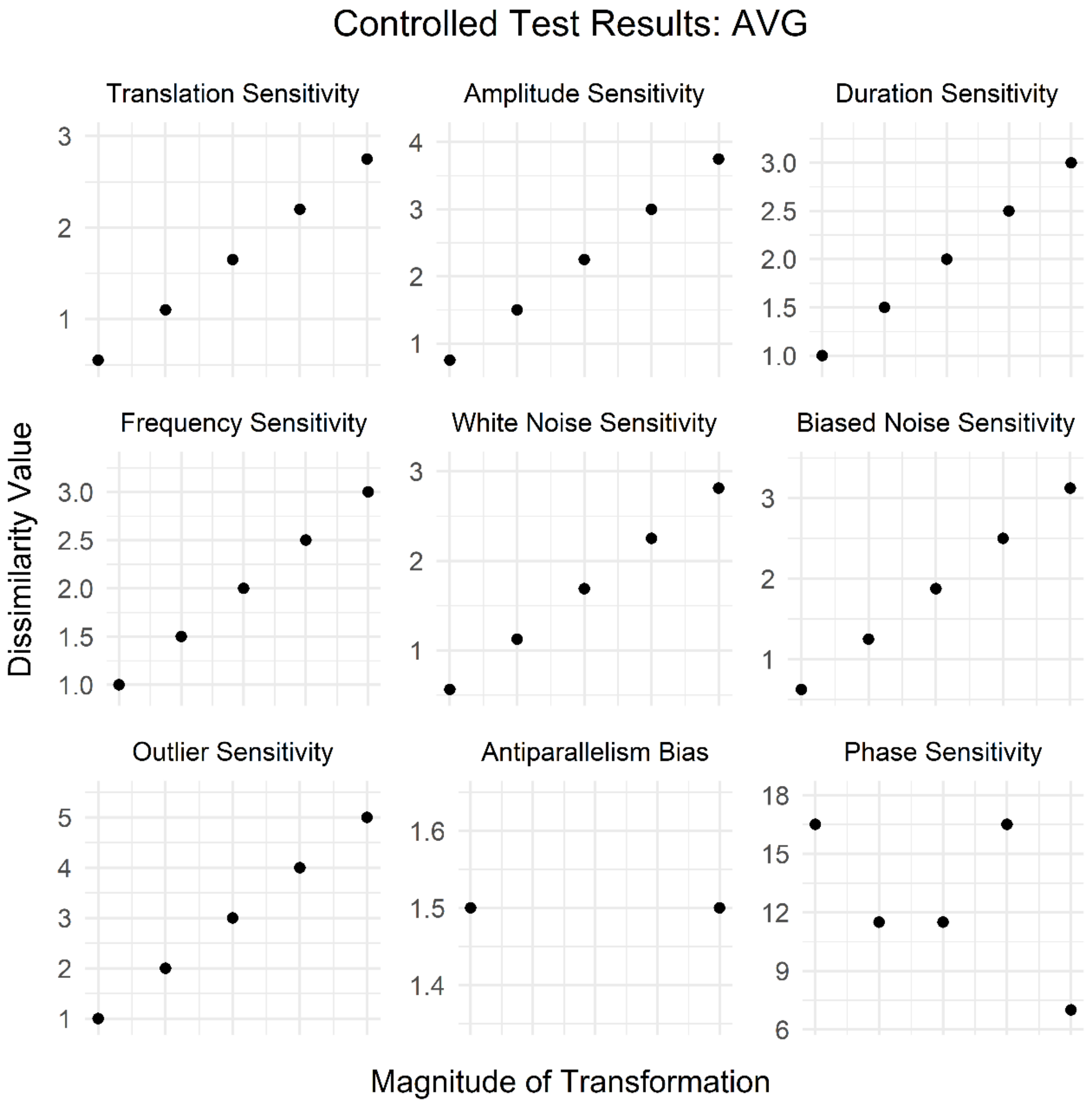

Fig. S3. Controlled testing results for the Average Distance. Sensitivities were tested by comparing time series with linearly increasing differences in summed y-axis values (or phase), against a reference time series. Antiparallelism bias was tested by comparing pairs of time series that differed by the same relative amount in different directions. The bias is neutral if the two values are identical, negative if the value on the left is higher, and positive if the value on the right is higher. Uniform time scaling sensitivity and warping sensitivity could not be tested for this distance measure, as it cannot measure unequal-length time series.

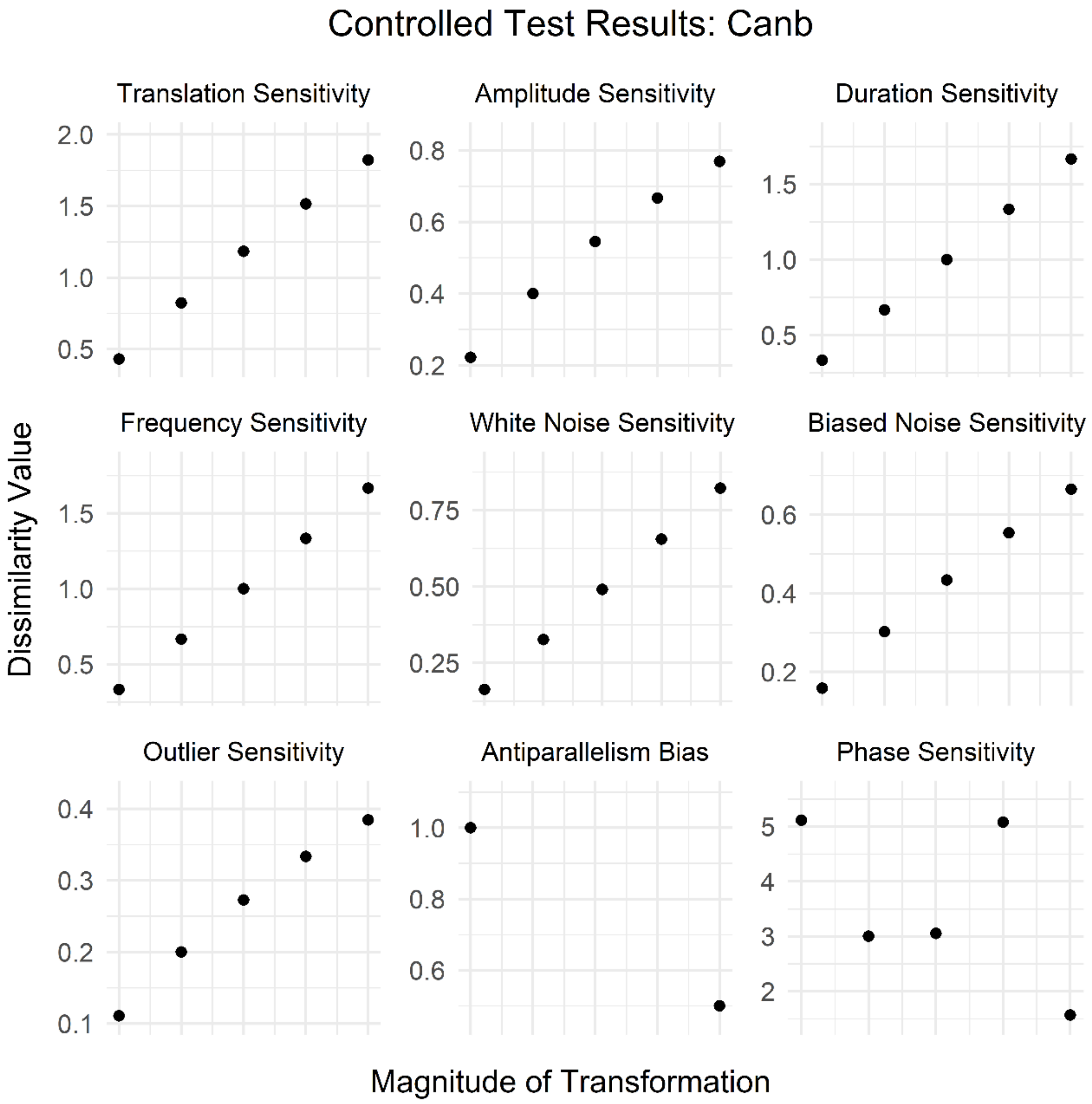

Fig. S4. Controlled testing results for the Canberra Distance. Sensitivities were tested by comparing time series with linearly increasing differences in summed y-axis values (or phase), against a reference time series. Antiparallelism bias was tested by comparing pairs of time series that differed by the same relative amount in different directions. The bias is neutral if the two values are identical, negative if the value on the left is higher, and positive if the value on the right is higher. Uniform time scaling sensitivity and warping sensitivity could not be tested for this distance measure, as it cannot measure unequal-length time series.

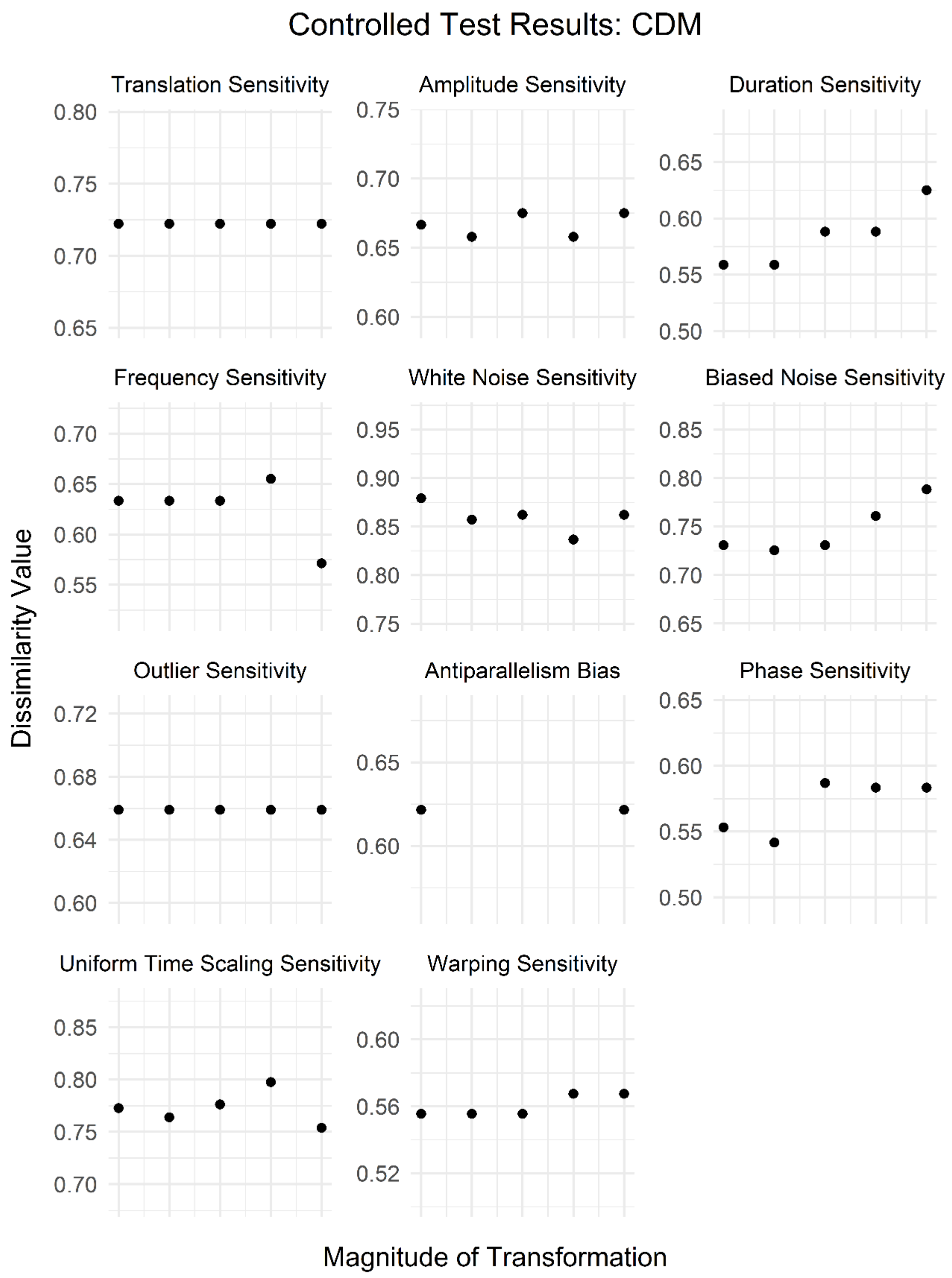

Fig. S5. Controlled testing results for the Compression-Based Dissimilarity Measure. Sensitivities were tested by comparing time series with linearly increasing differences in summed y-axis values (or phase), against a reference time series. Antiparallelism bias was tested by comparing pairs of time series that differed by the same relative amount in different directions. The bias is neutral if the two values are identical, negative if the value on the left is higher, and positive if the value on the right is higher. Uniform time scaling sensitivity and warping sensitivity were tested by stretching time series, or parts of time series, respectively, by different amounts.

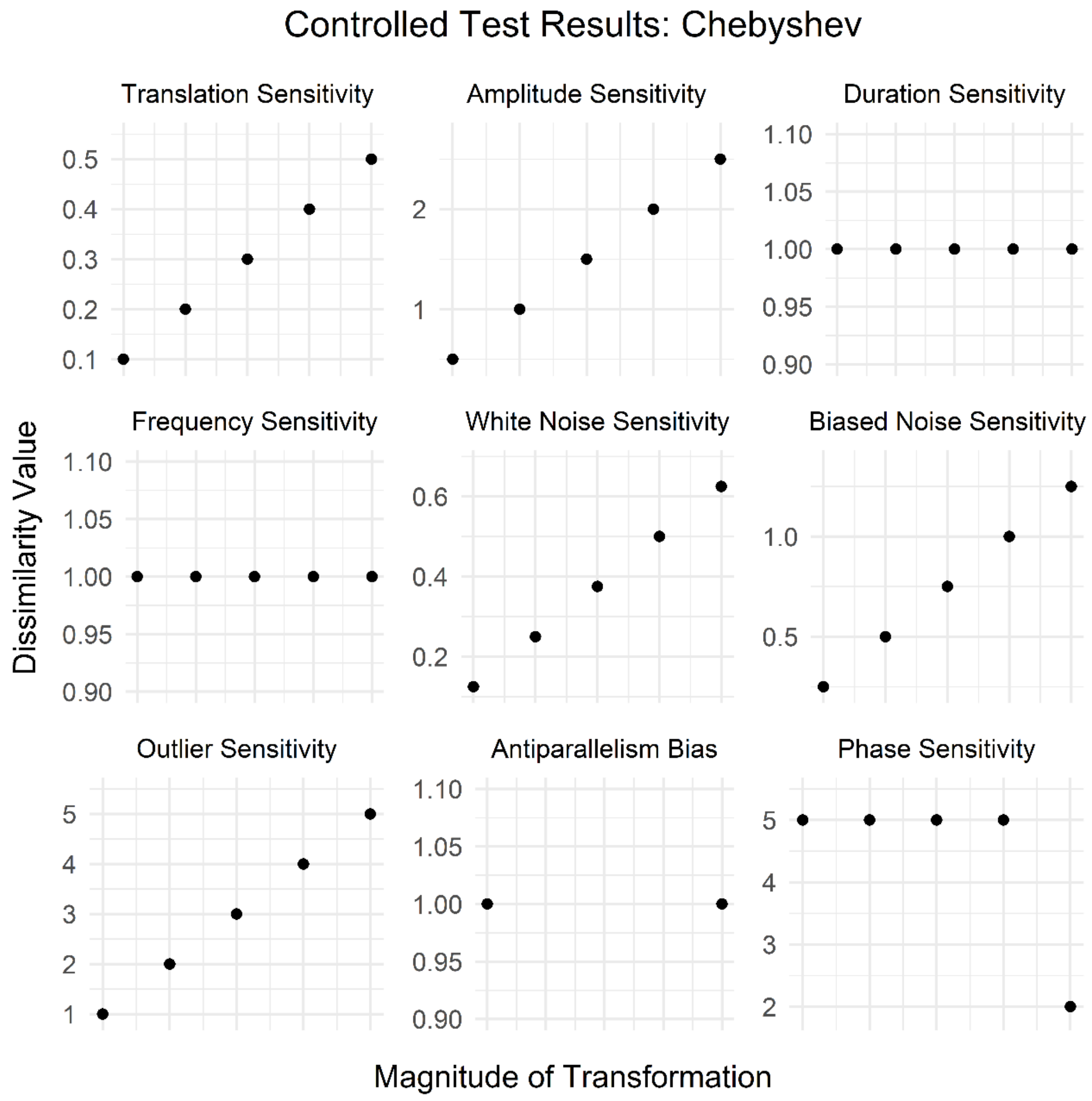

Fig. S6. Controlled testing results for the Chebyshev Distance. Sensitivities were tested by comparing time series with linearly increasing differences in summed y-axis values (or phase), against a reference time series. Antiparallelism bias was tested by comparing pairs of time series that differed by the same relative amount in different directions. The bias is neutral if the two values are identical, negative if the value on the left is higher, and positive if the value on the right is higher. Uniform time scaling sensitivity and warping sensitivity could not be tested for this distance measure, as it cannot measure unequal-length time series.

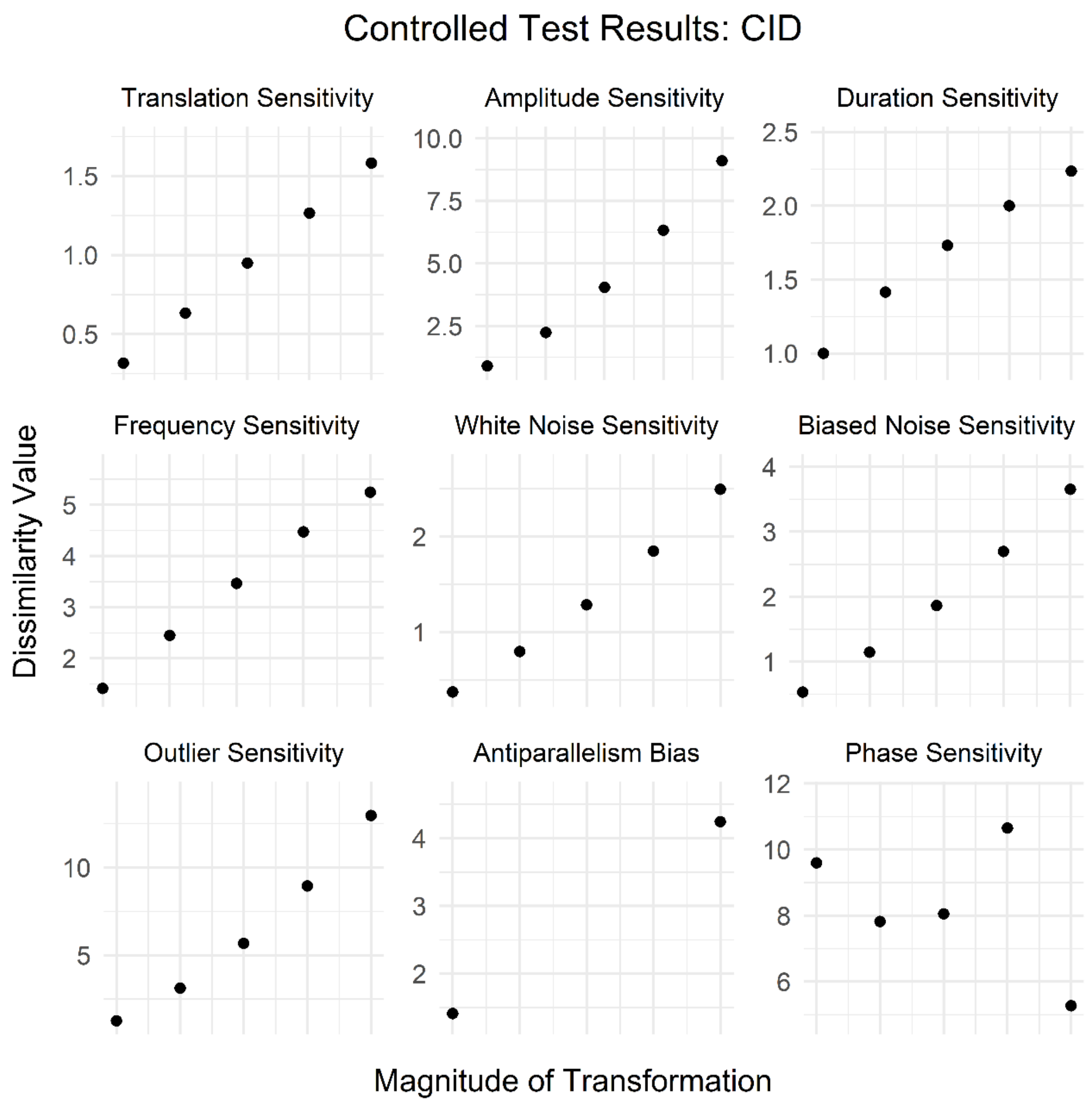

Fig. S7. Controlled testing results for the Complexity-Invariant Distance. Sensitivities were tested by comparing time series with linearly increasing differences in summed y-axis values (or phase), against a reference time series. Antiparallelism bias was tested by comparing pairs of time series that differed by the same relative amount in different directions. The bias is neutral if the two values are identical, negative if the value on the left is higher, and positive if the value on the right is higher. Uniform time scaling sensitivity and warping sensitivity could not be tested for this distance measure, as it cannot measure unequal-length time series.

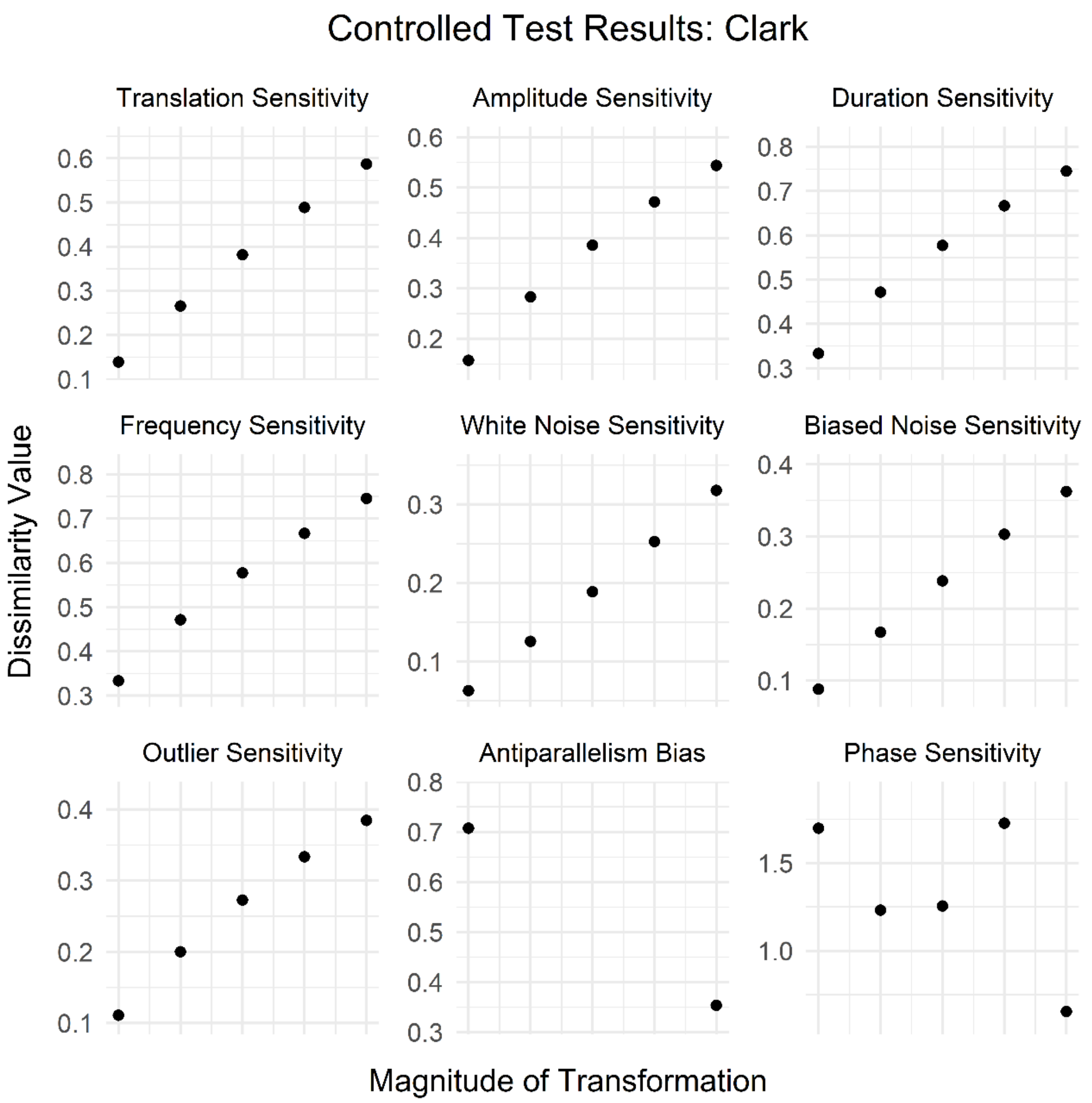

Fig. S8. Controlled testing results for the Clark Squared Distance. Sensitivities were tested by comparing time series with linearly increasing differences in summed y-axis values (or phase), against a reference time series. Antiparallelism bias was tested by comparing pairs of time series that differed by the same relative amount in different directions. The bias is neutral if the two values are identical, negative if the value on the left is higher, and positive if the value on the right is higher. Uniform time scaling sensitivity and warping sensitivity could not be tested for this distance measure, as it cannot measure unequal-length time series.

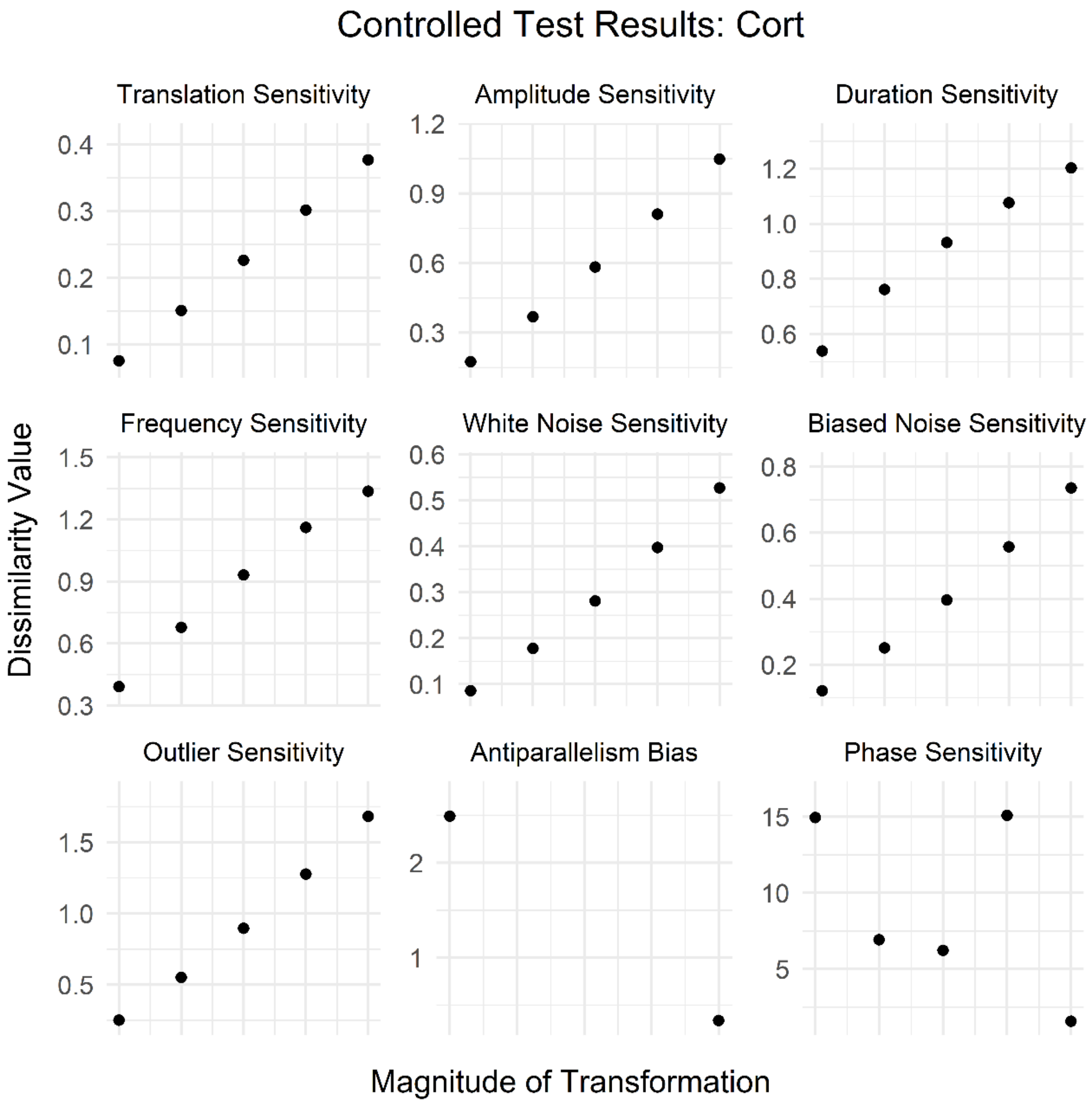

Fig. S9. Controlled testing results for the Dissimilarity Index Combining Temporal Correlation and Raw Value Behaviour. Sensitivities were tested by comparing time series with linearly increasing differences in summed y-axis values (or phase), against a reference time series. Antiparallelism bias was tested by comparing pairs of time series that differed by the same relative amount in different directions. The bias is neutral if the two values are identical, negative if the value on the left is higher, and positive if the value on the right is higher. Uniform time scaling sensitivity and warping sensitivity could not be tested for this distance measure, as it cannot measure unequal-length time series.

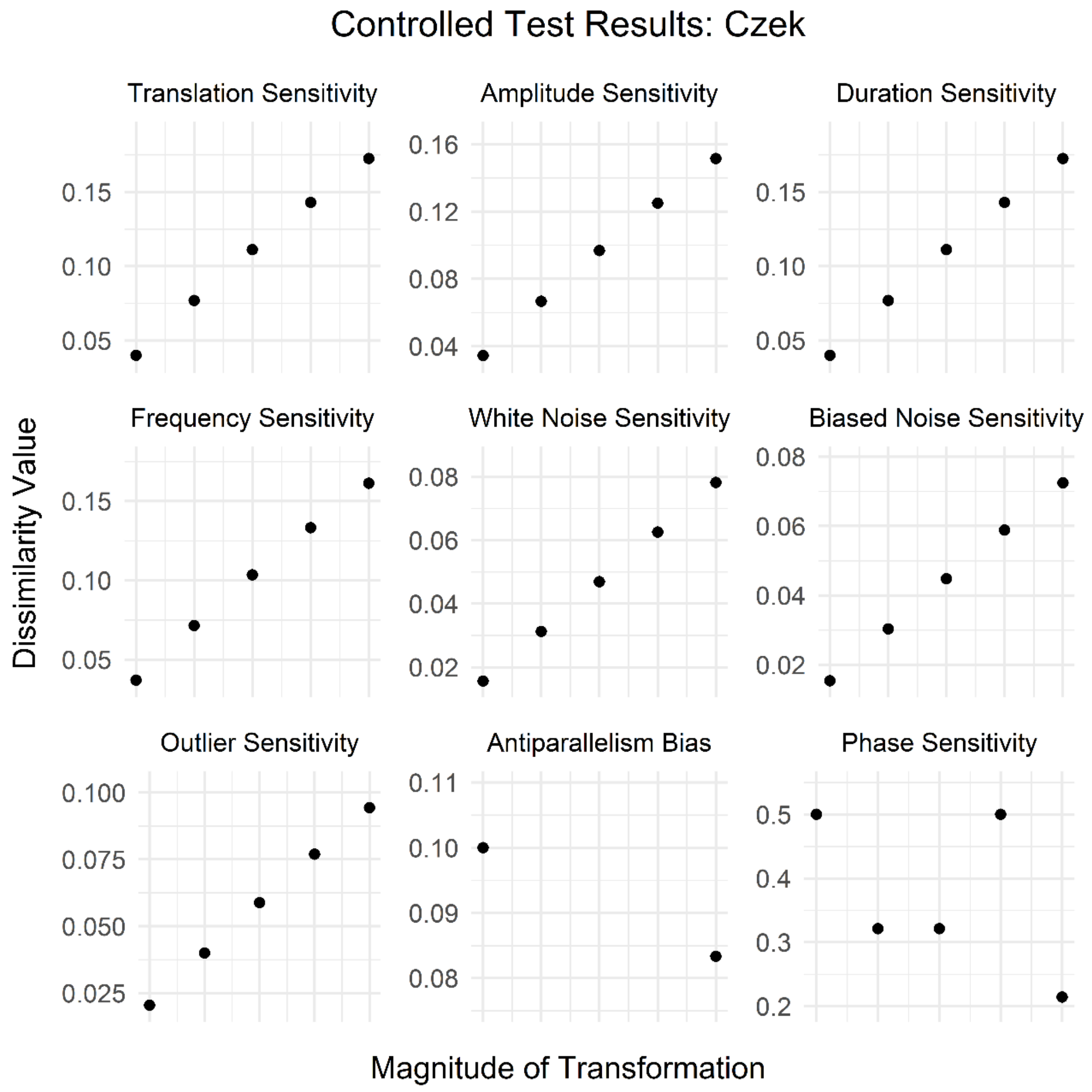

Fig. S10. Controlled testing results for the Czekanowski Distance. Sensitivities were tested by comparing time series with linearly increasing differences in summed y-axis values (or phase), against a reference time series. Antiparallelism bias was tested by comparing pairs of time series that differed by the same relative amount in different directions. The bias is neutral if the two values are identical, negative if the value on the left is higher, and positive if the value on the right is higher. Uniform time scaling sensitivity and warping sensitivity could not be tested for this distance measure, as it cannot measure unequal-length time series.

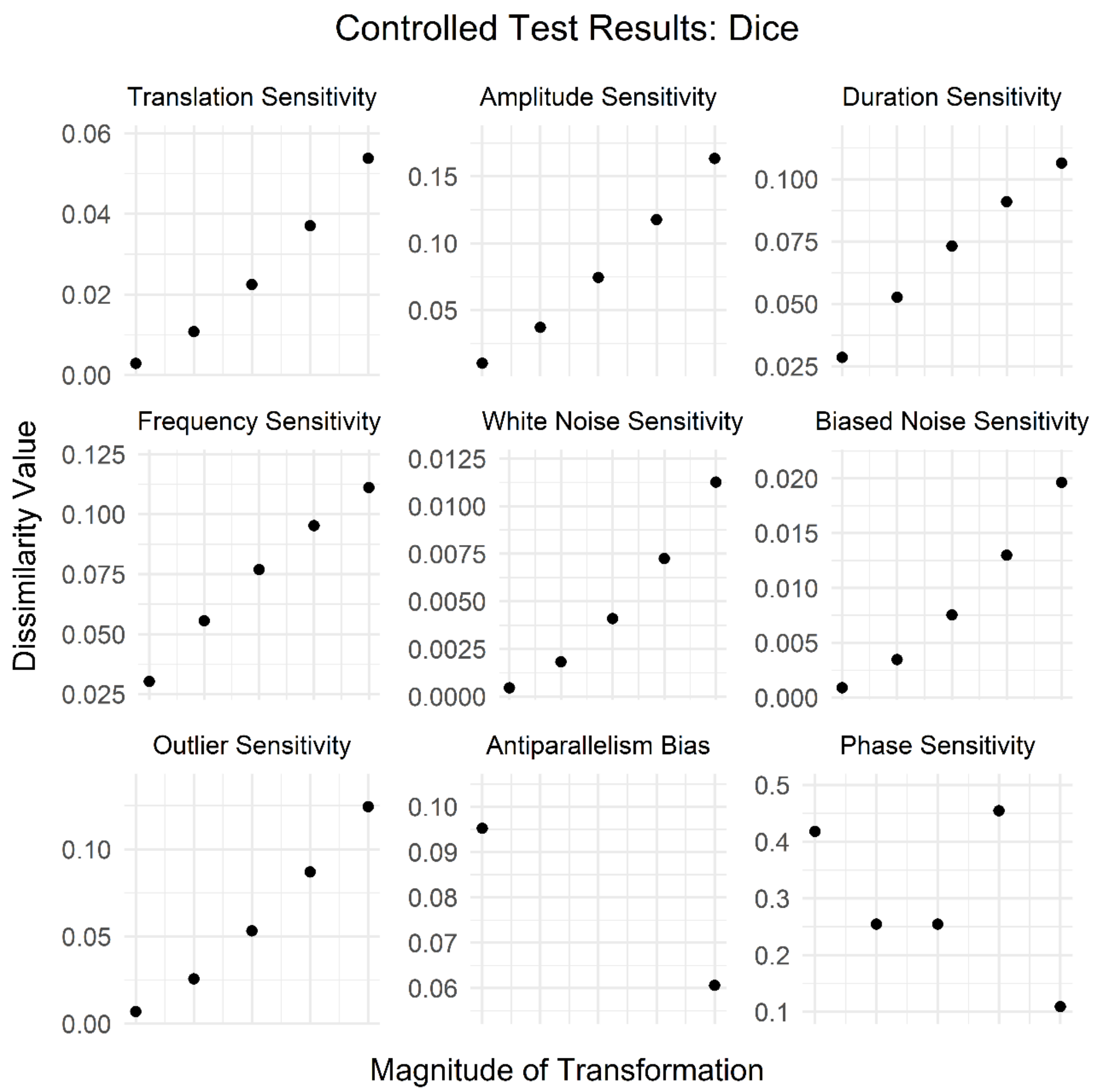

Fig. S11. Controlled testing results for the Dice Dissimilarity. Sensitivities were tested by comparing time series with linearly increasing differences in summed y-axis values (or phase), against a reference time series. Antiparallelism bias was tested by comparing pairs of time series that differed by the same relative amount in different directions. The bias is neutral if the two values are identical, negative if the value on the left is higher, and positive if the value on the right is higher. Uniform time scaling sensitivity and warping sensitivity could not be tested for this distance measure, as it cannot measure unequal-length time series.

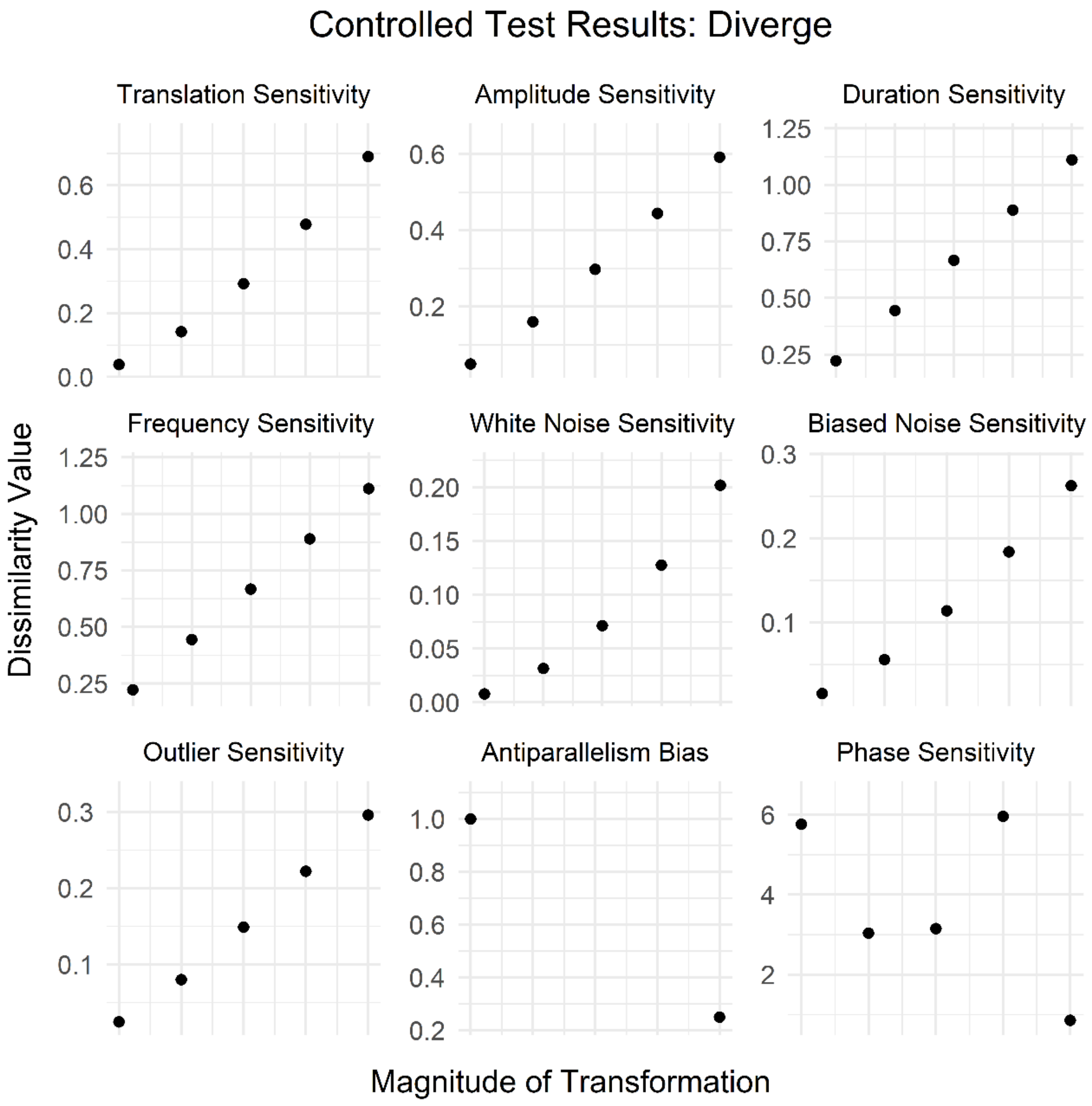

Fig. S12. Controlled testing results for the Divergence Squared distance. Sensitivities were tested by comparing time series with linearly increasing differences in summed y-axis values (or phase), against a reference time series. Antiparallelism bias was tested by comparing pairs of time series that differed by the same relative amount in different directions. The bias is neutral if the two values are identical, negative if the value on the left is higher, and positive if the value on the right is higher. Uniform time scaling sensitivity and warping sensitivity could not be tested for this distance measure, as it cannot measure unequal-length time series.

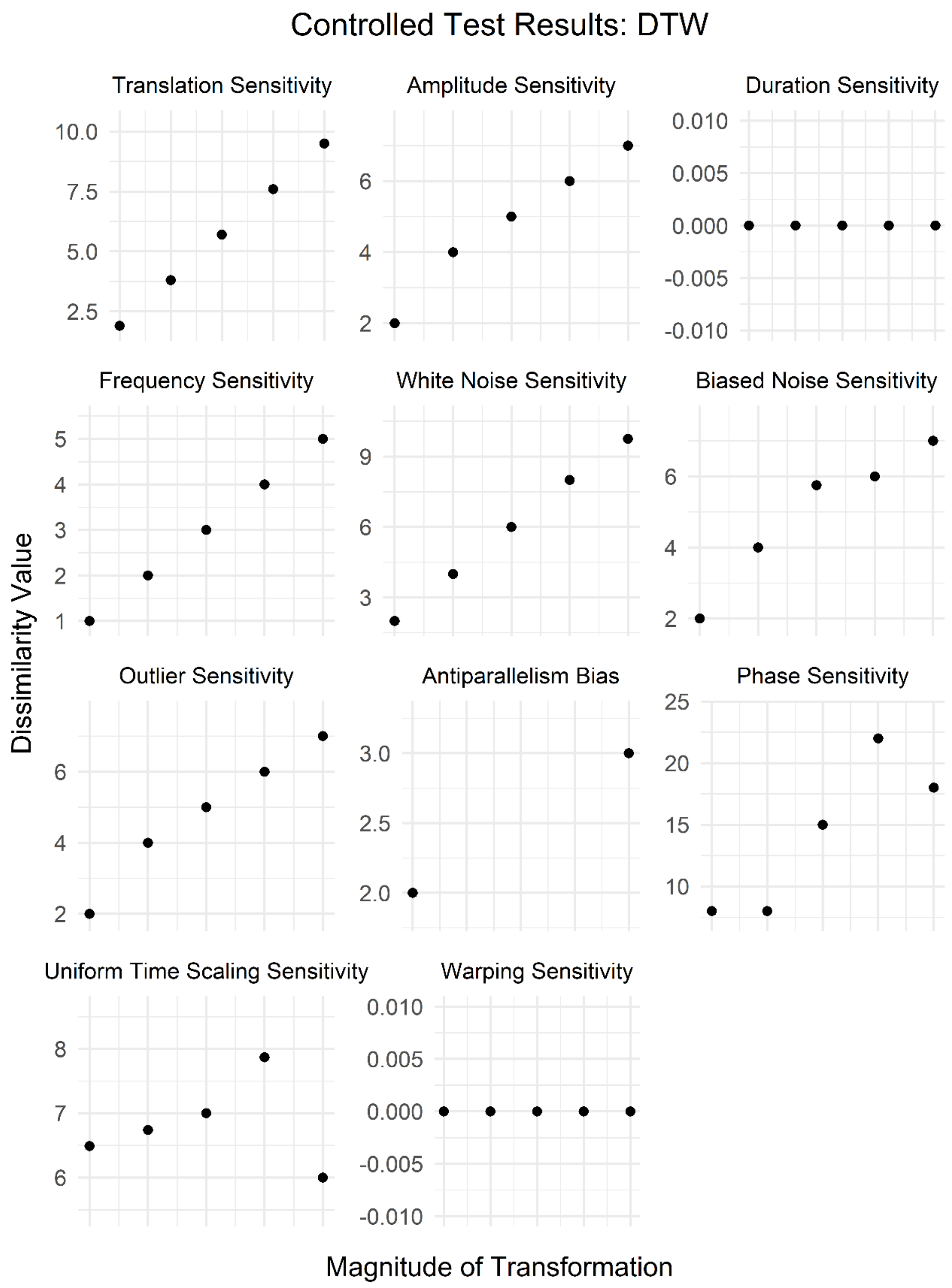

Fig. S13. Controlled testing results for the Dynamic Time Warping Distance. Sensitivities were tested by comparing time series with linearly increasing differences in summed y-axis values (or phase), against a reference time series. Antiparallelism bias was tested by comparing pairs of time series that differed by the same relative amount in different directions. The bias is neutral if the two values are identical, negative if the value on the left is higher, and positive if the value on the right is higher. Uniform time scaling sensitivity and warping sensitivity were tested by stretching time series, or parts of time series, respectively, by different amounts.

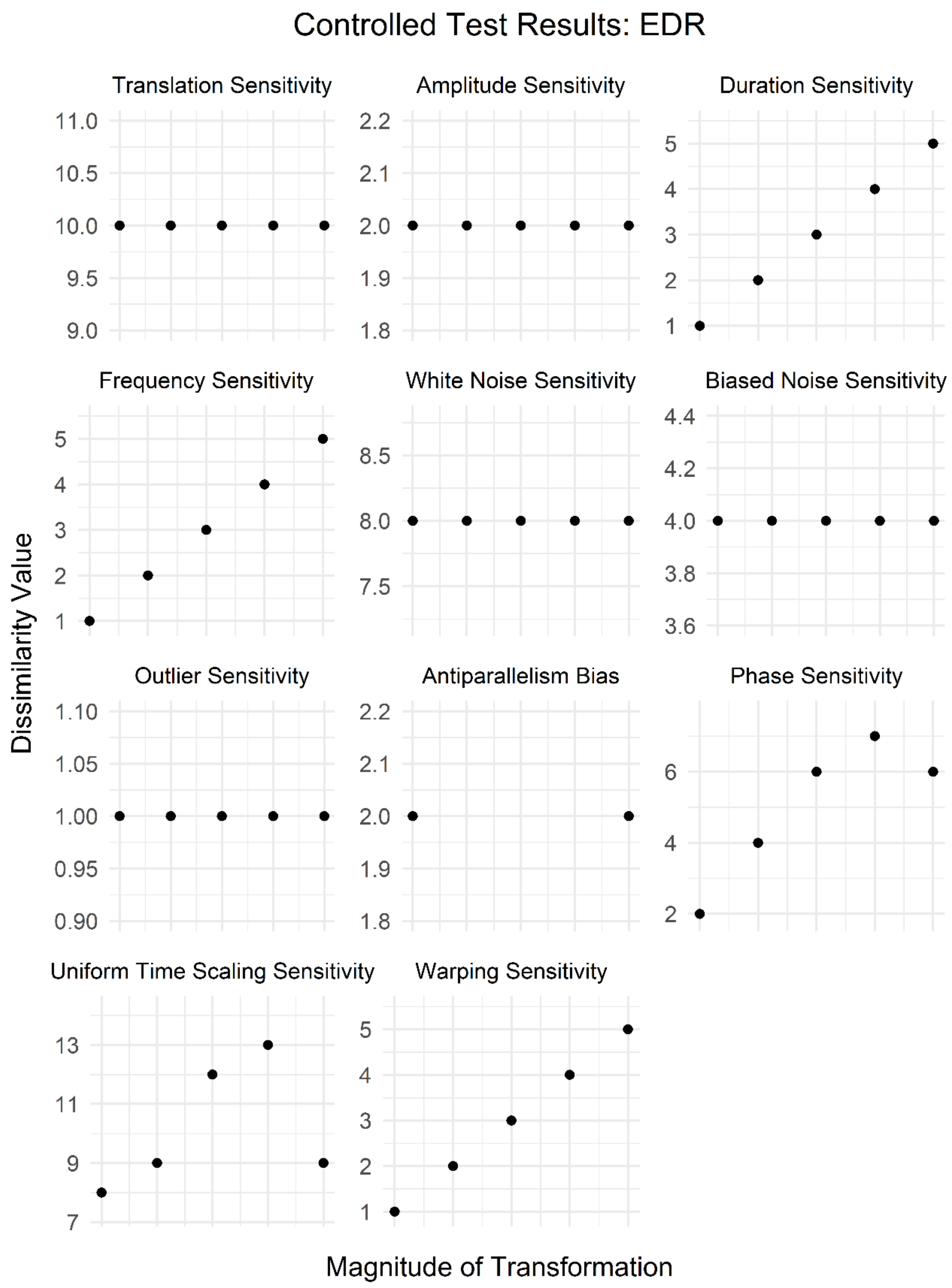

Fig. S14. Controlled testing results for the Edit Distance on Real Sequences. Sensitivities were tested by comparing time series with linearly increasing differences in summed y-axis values (or phase), against a reference time series. Antiparallelism bias was tested by comparing pairs of time series that differed by the same relative amount in different directions. The bias is neutral if the two values are identical, negative if the value on the left is higher, and positive if the value on the right is higher. Uniform time scaling sensitivity and warping sensitivity were tested by stretching time series, or parts of time series, respectively, by different amounts.

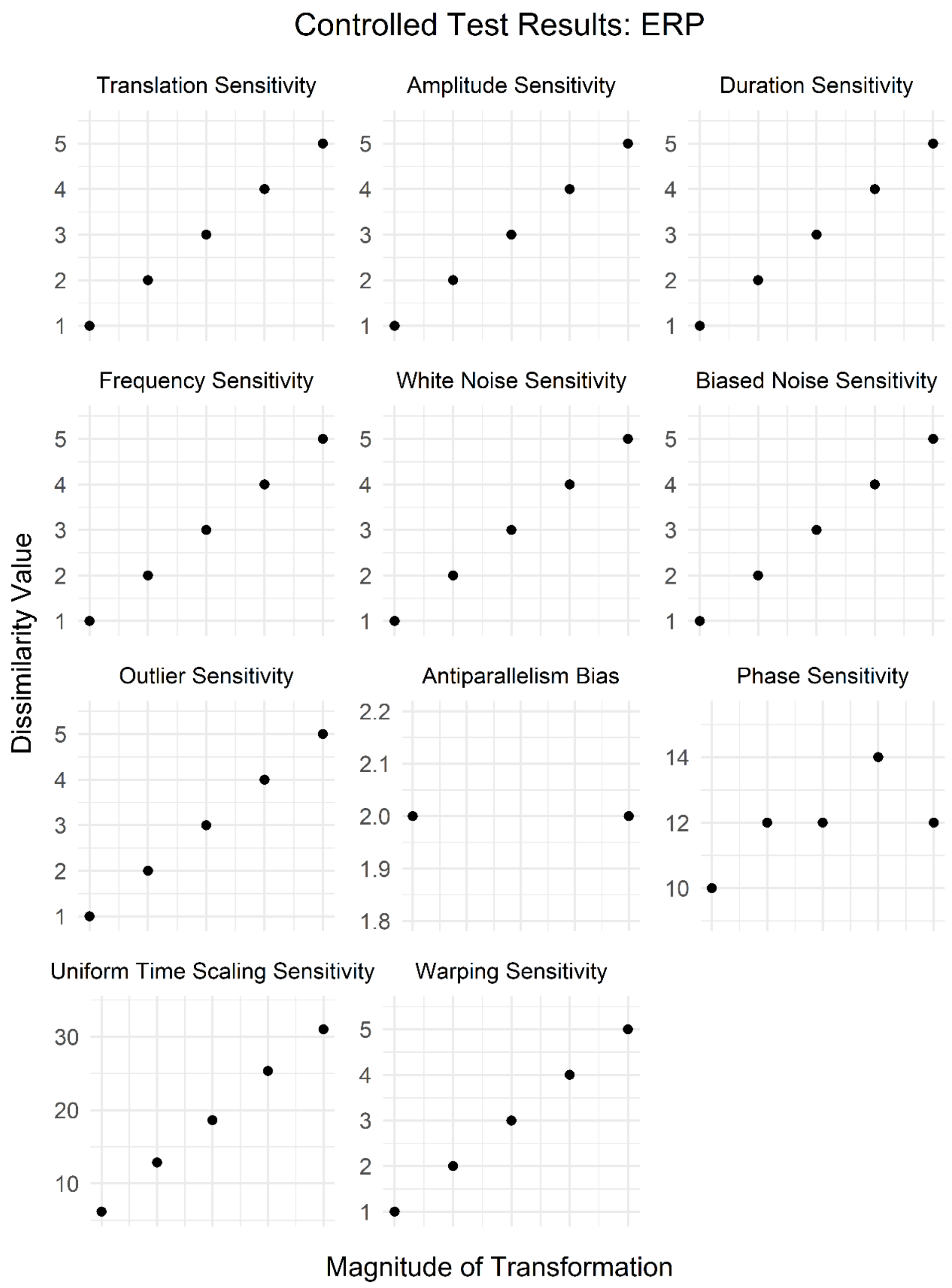

Fig. S15. Controlled testing results for the Edit Distance with Real Penalty. Sensitivities were tested by comparing time series with linearly increasing differences in summed y-axis values (or phase), against a reference time series. Antiparallelism bias was tested by comparing pairs of time series that differed by the same relative amount in different directions. The bias is neutral if the two values are identical, negative if the value on the left is higher, and positive if the value on the right is higher. Uniform time scaling sensitivity and warping sensitivity were tested by stretching time series, or parts of time series, respectively, by different amounts.

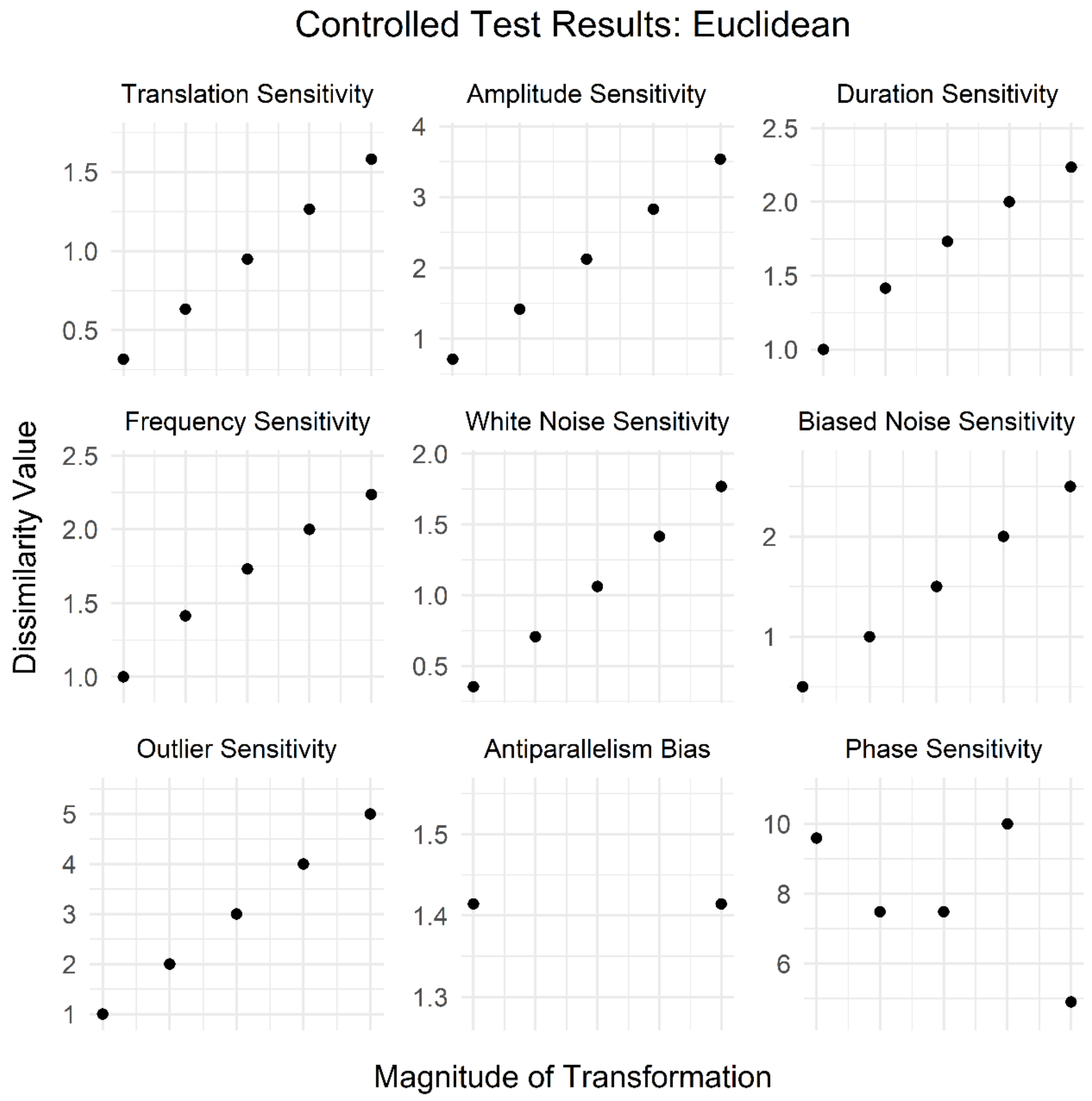

Fig. S16. Controlled testing results for the Euclidean Distance. Sensitivities were tested by comparing time series with linearly increasing differences in summed y-axis values (or phase), against a reference time series. Antiparallelism bias was tested by comparing pairs of time series that differed by the same relative amount in different directions. The bias is neutral if the two values are identical, negative if the value on the left is higher, and positive if the value on the right is higher. Uniform time scaling sensitivity and warping sensitivity could not be tested for this distance measure, as it cannot measure unequal-length time series.

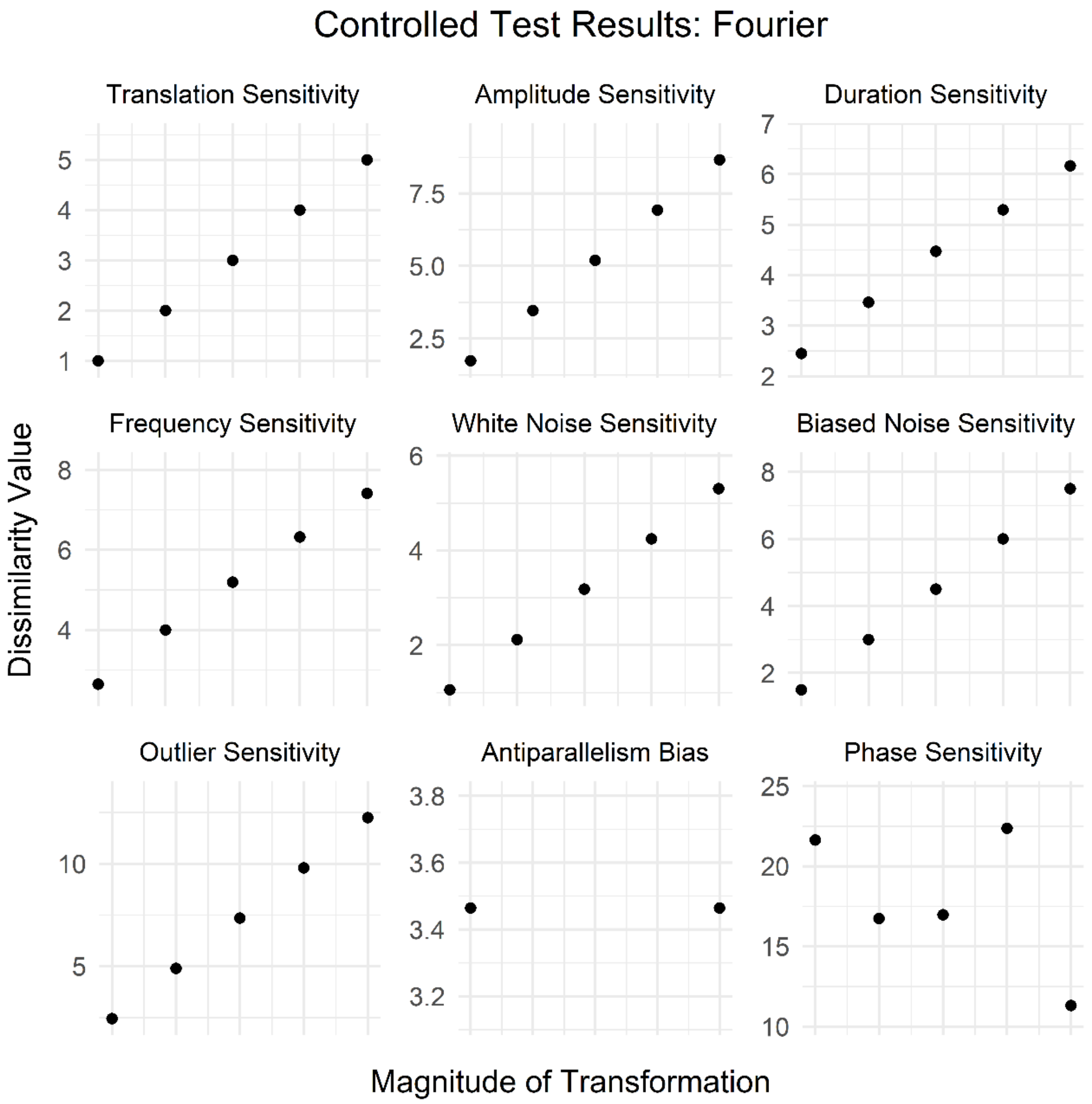

Fig. S17. Controlled testing results for the Fourier Coefficient-Based Distance. Sensitivities were tested by comparing time series with linearly increasing differences in summed y-axis values (or phase), against a reference time series. Antiparallelism bias was tested by comparing pairs of time series that differed by the same relative amount in different directions. The bias is neutral if the two values are identical, negative if the value on the left is higher, and positive if the value on the right is higher. Uniform time scaling sensitivity and warping sensitivity could not be tested for this distance measure, as it cannot measure unequal-length time series.

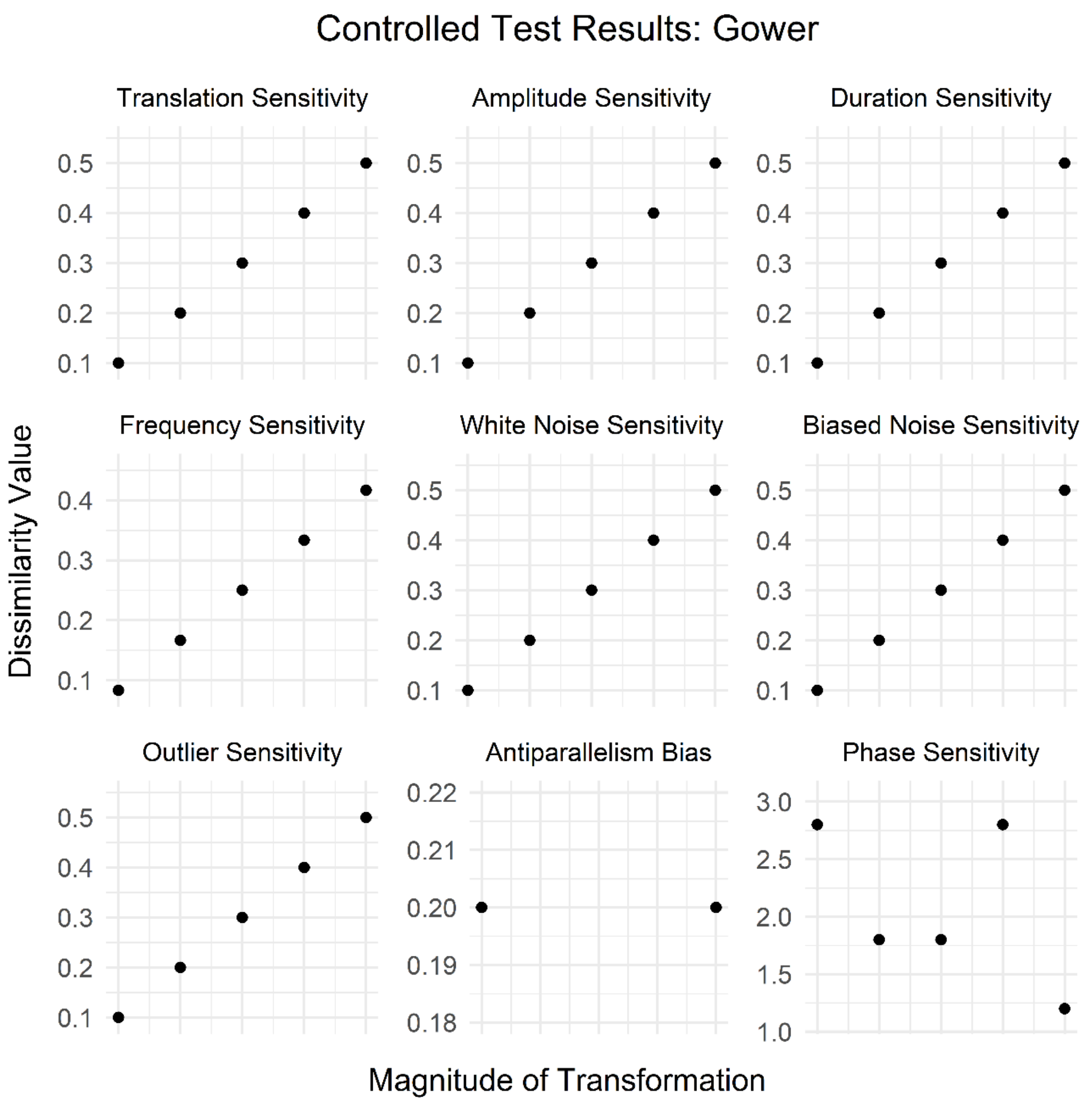

Fig. S18. Controlled testing results for the Gower Distance. Sensitivities were tested by comparing time series with linearly increasing differences in summed y-axis values (or phase), against a reference time series. Antiparallelism bias was tested by comparing pairs of time series that differed by the same relative amount in different directions. The bias is neutral if the two values are identical, negative if the value on the left is higher, and positive if the value on the right is higher. Uniform time scaling sensitivity and warping sensitivity could not be tested for this distance measure, as it cannot measure unequal-length time series.

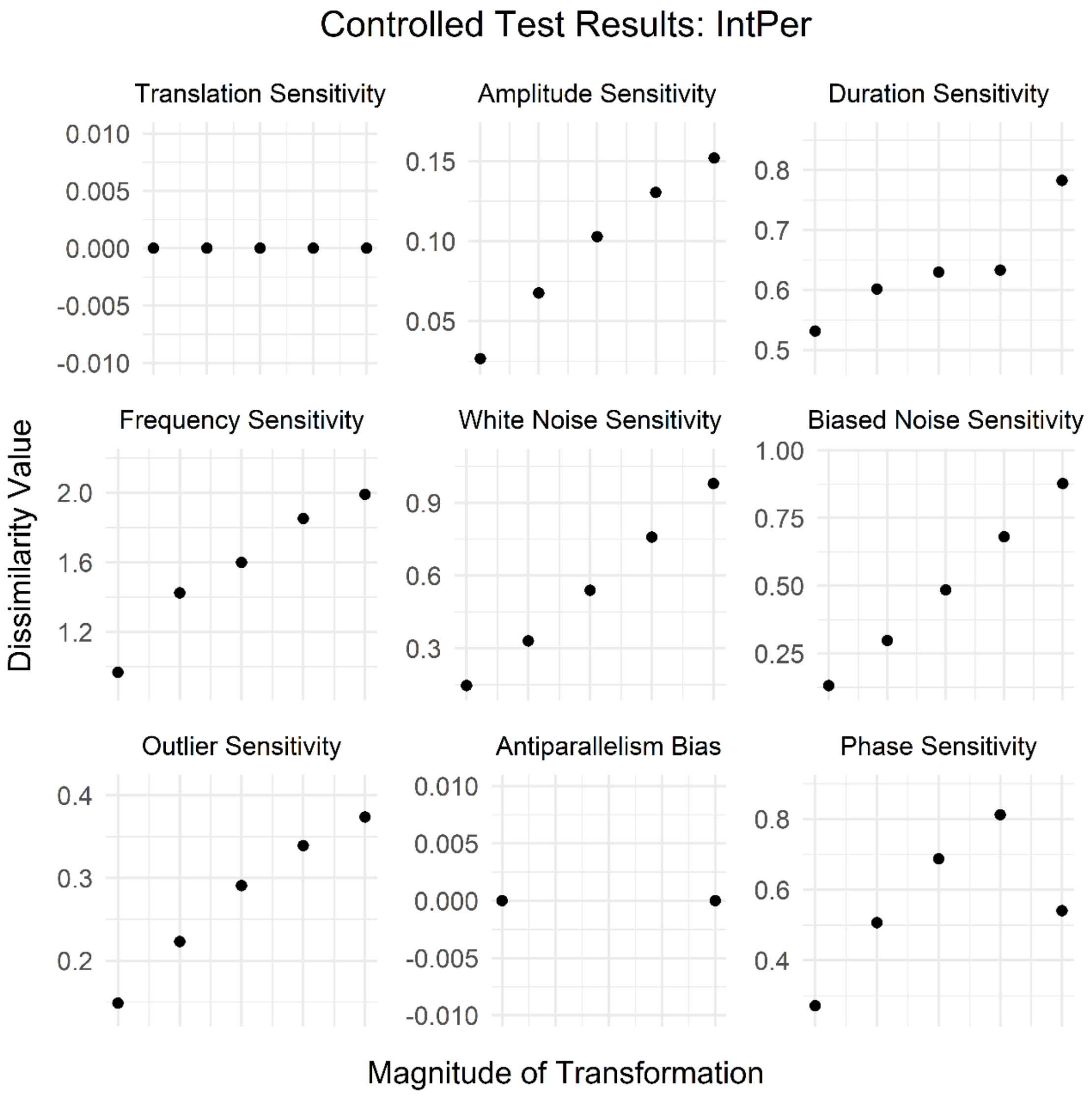

Fig. S19. Controlled testing results for the Integrated Periodogram Based Dissimilarity. Sensitivities were tested by comparing time series with linearly increasing differences in summed y-axis values (or phase), against a reference time series. Antiparallelism bias was tested by comparing pairs of time series that differed by the same relative amount in different directions. The bias is neutral if the two values are identical, negative if the value on the left is higher, and positive if the value on the right is higher. Uniform time scaling sensitivity and warping sensitivity could not be tested for this distance measure, as it cannot measure unequal-length time series.

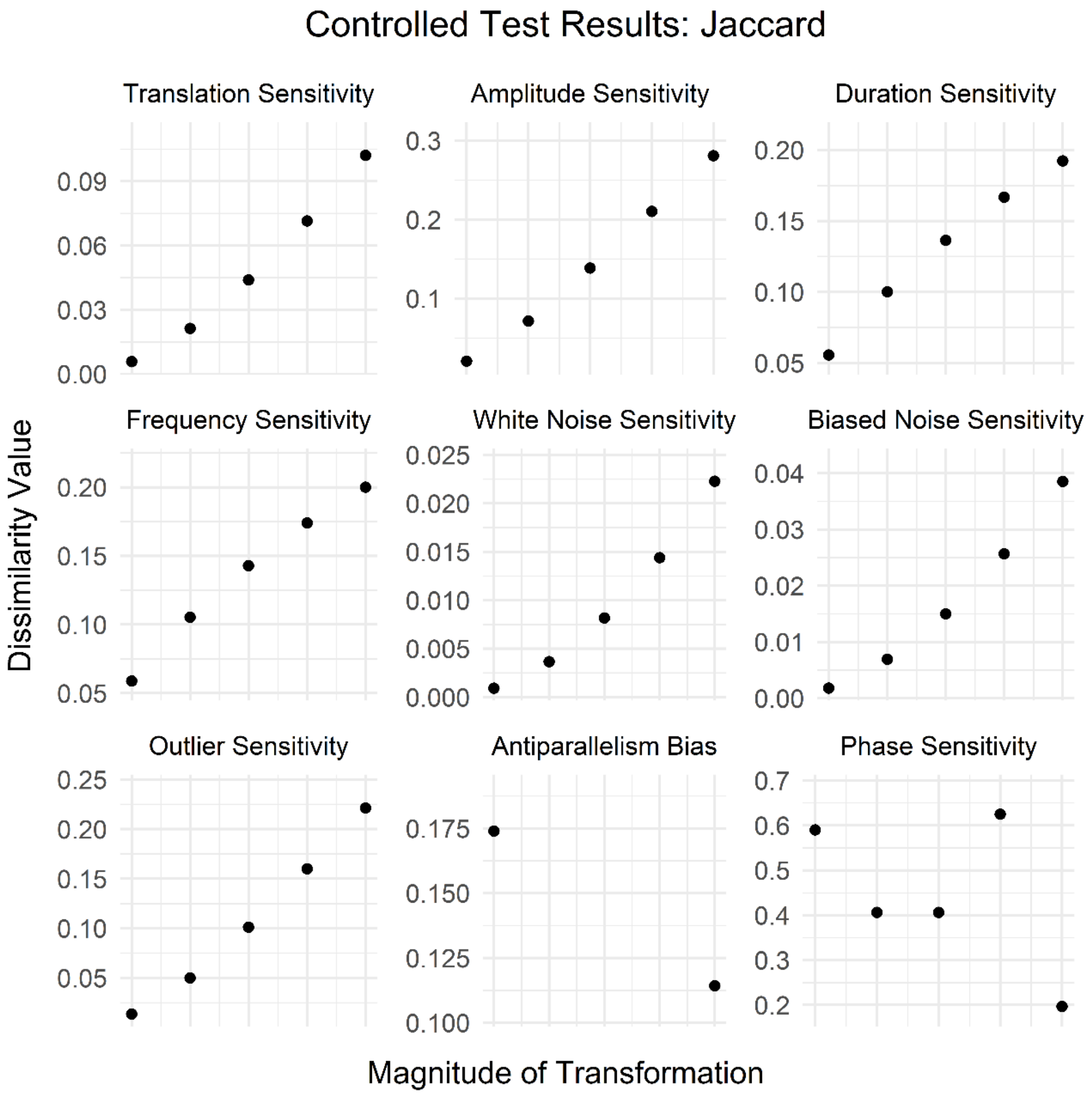

Fig. S20. Controlled testing results for the Jaccard Distance. Sensitivities were tested by comparing time series with linearly increasing differences in summed y-axis values (or phase), against a reference time series. Antiparallelism bias was tested by comparing pairs of time series that differed by the same relative amount in different directions. The bias is neutral if the two values are identical, negative if the value on the left is higher, and positive if the value on the right is higher. Uniform time scaling sensitivity and warping sensitivity could not be tested for this distance measure, as it cannot measure unequal-length time series.

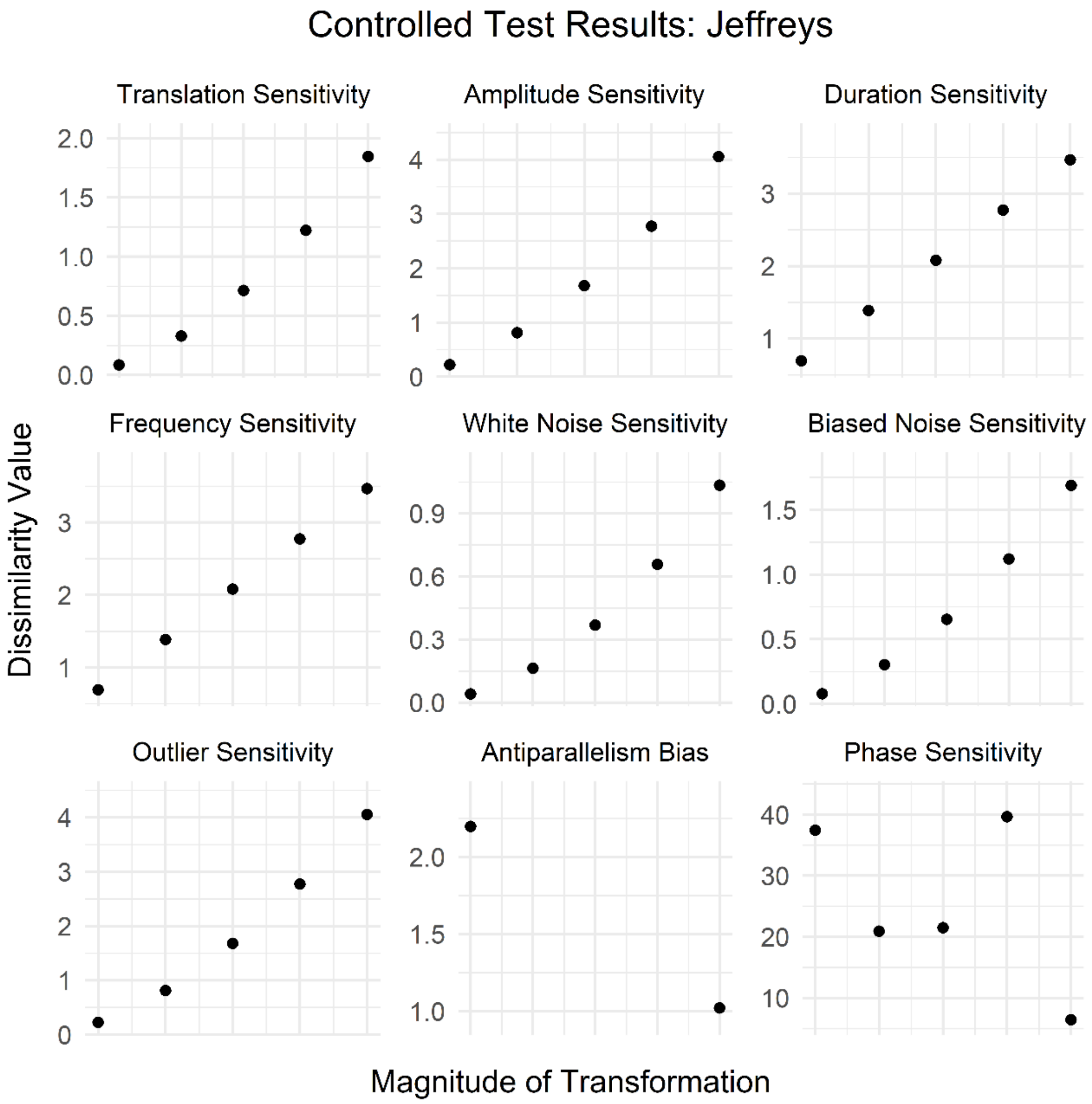

Fig. S21. Controlled testing results for the Jeffreys Divergence. Sensitivities were tested by comparing time series with linearly increasing differences in summed y-axis values (or phase), against a reference time series. Antiparallelism bias was tested by comparing pairs of time series that differed by the same relative amount in different directions. The bias is neutral if the two values are identical, negative if the value on the left is higher, and positive if the value on the right is higher. Uniform time scaling sensitivity and warping sensitivity could not be tested for this distance measure, as it cannot measure unequal-length time series.

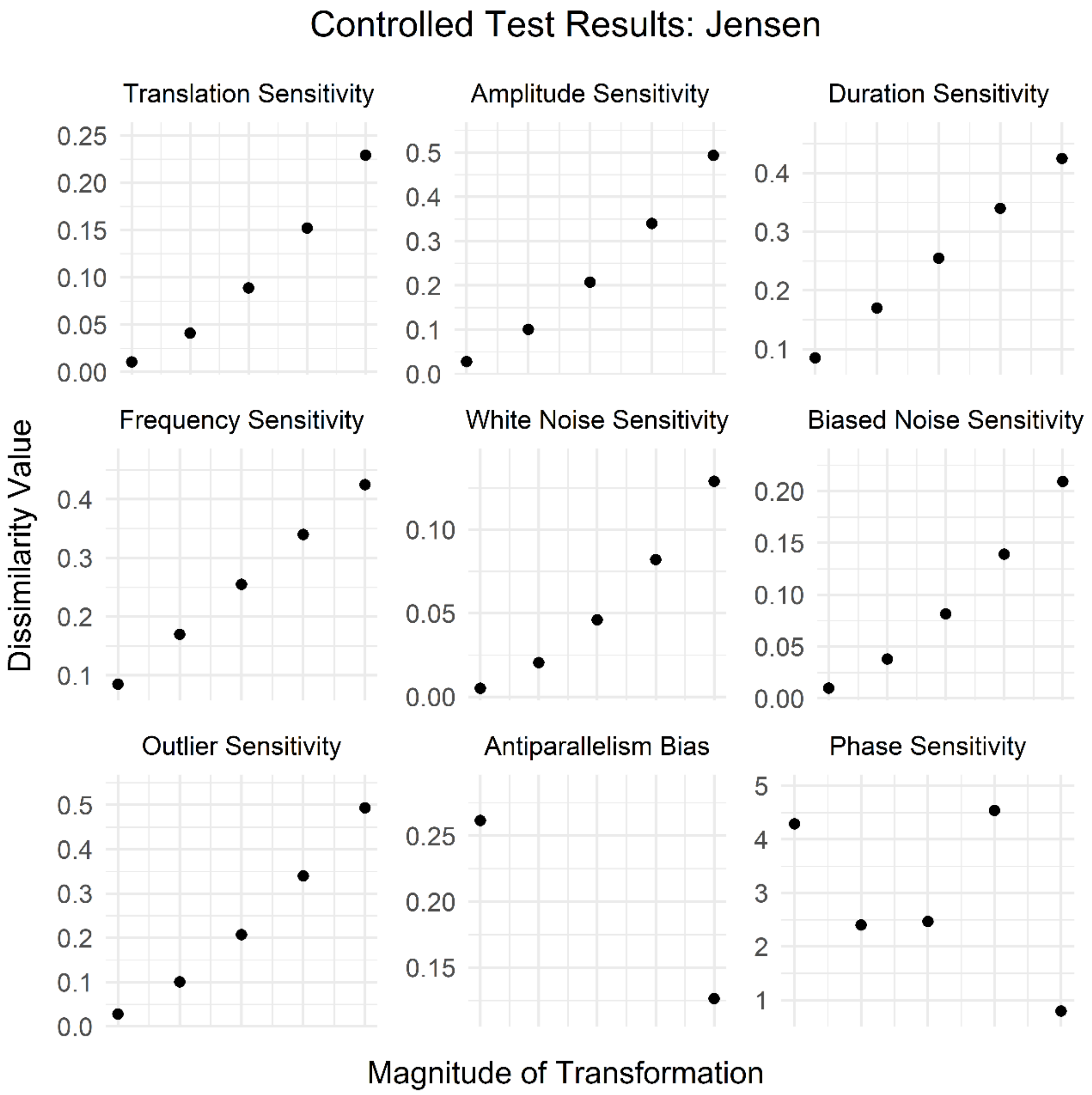

Fig. S22. Controlled testing results for the Jensen Difference. Sensitivities were tested by comparing time series with linearly increasing differences in summed y-axis values (or phase), against a reference time series. Antiparallelism bias was tested by comparing pairs of time series that differed by the same relative amount in different directions. The bias is neutral if the two values are identical, negative if the value on the left is higher, and positive if the value on the right is higher. Uniform time scaling sensitivity and warping sensitivity could not be tested for this distance measure, as it cannot measure unequal-length time series.

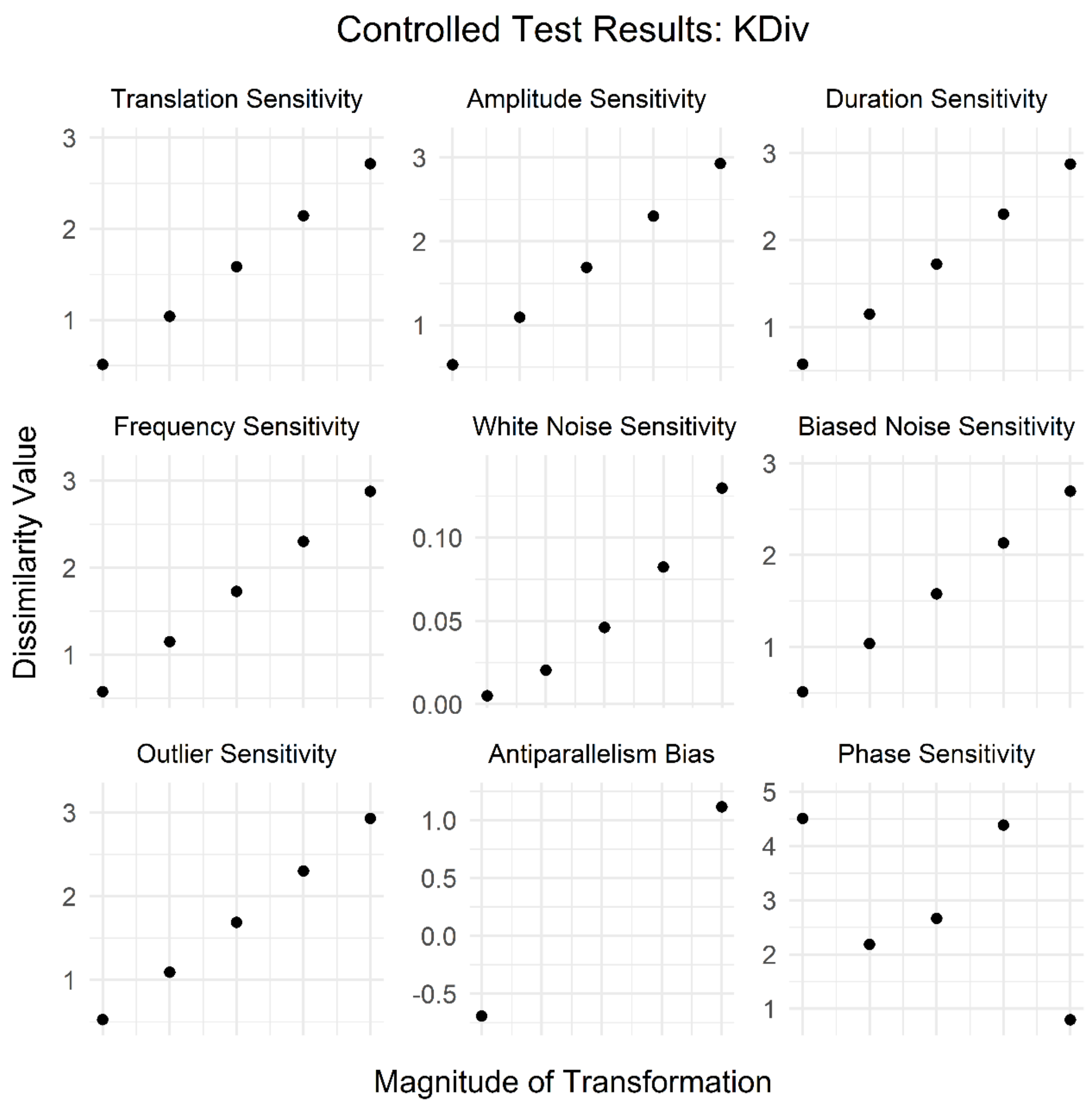

Fig. S23. Controlled testing results for the K Divergence. Sensitivities were tested by comparing time series with linearly increasing differences in summed y-axis values (or phase), against a reference time series. Antiparallelism bias was tested by comparing pairs of time series that differed by the same relative amount in different directions. The bias is neutral if the two values are identical, negative if the value on the left is higher, and positive if the value on the right is higher. Uniform time scaling sensitivity and warping sensitivity could not be tested for this distance measure, as it cannot measure unequal-length time series.

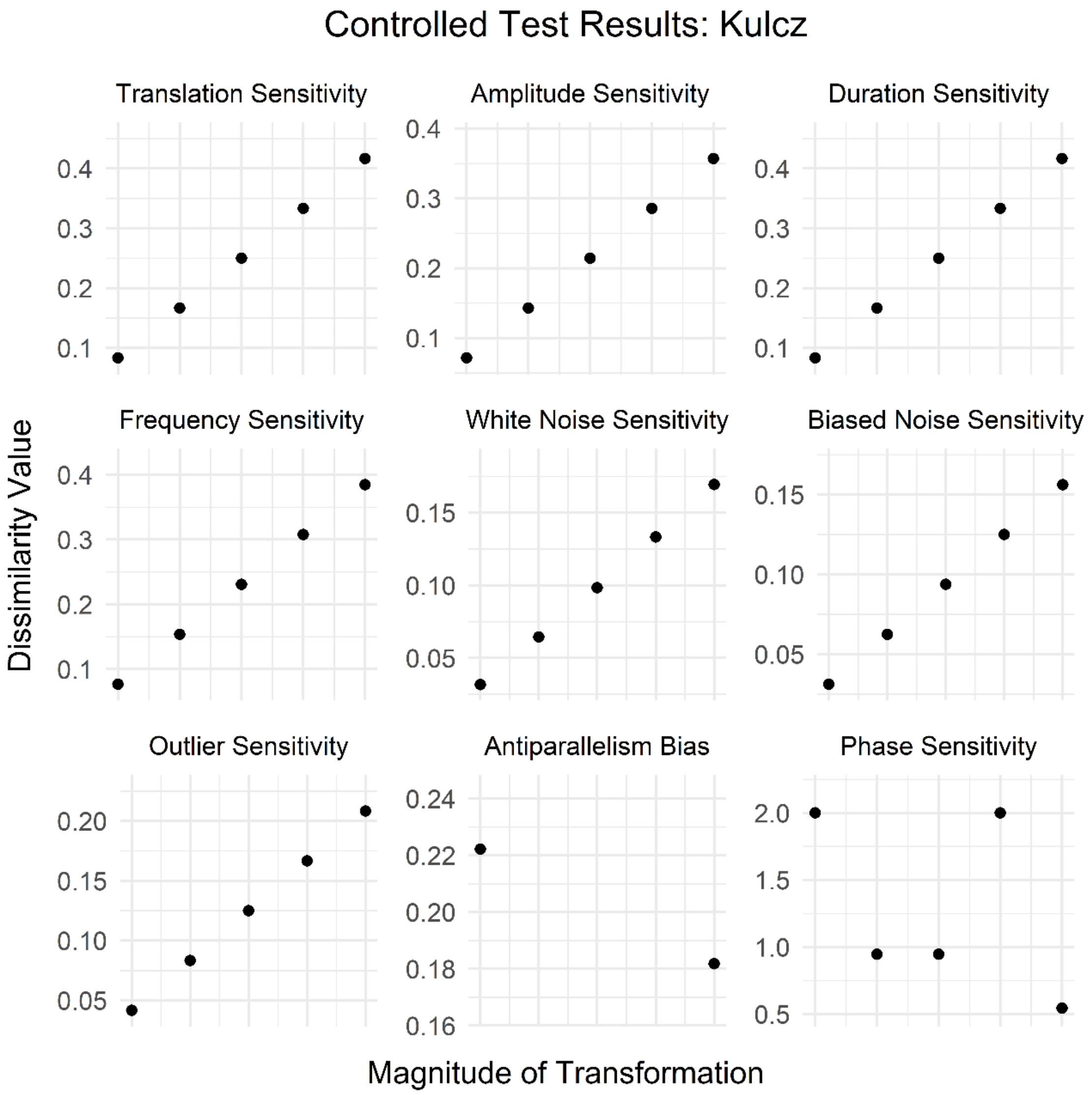

Fig. S24. Controlled testing results for the Kulczynski Distance. Sensitivities were tested by comparing time series with linearly increasing differences in summed y-axis values (or phase), against a reference time series. Antiparallelism bias was tested by comparing pairs of time series that differed by the same relative amount in different directions. The bias is neutral if the two values are identical, negative if the value on the left is higher, and positive if the value on the right is higher. Uniform time scaling sensitivity and warping sensitivity could not be tested for this distance measure, as it cannot measure unequal-length time series.

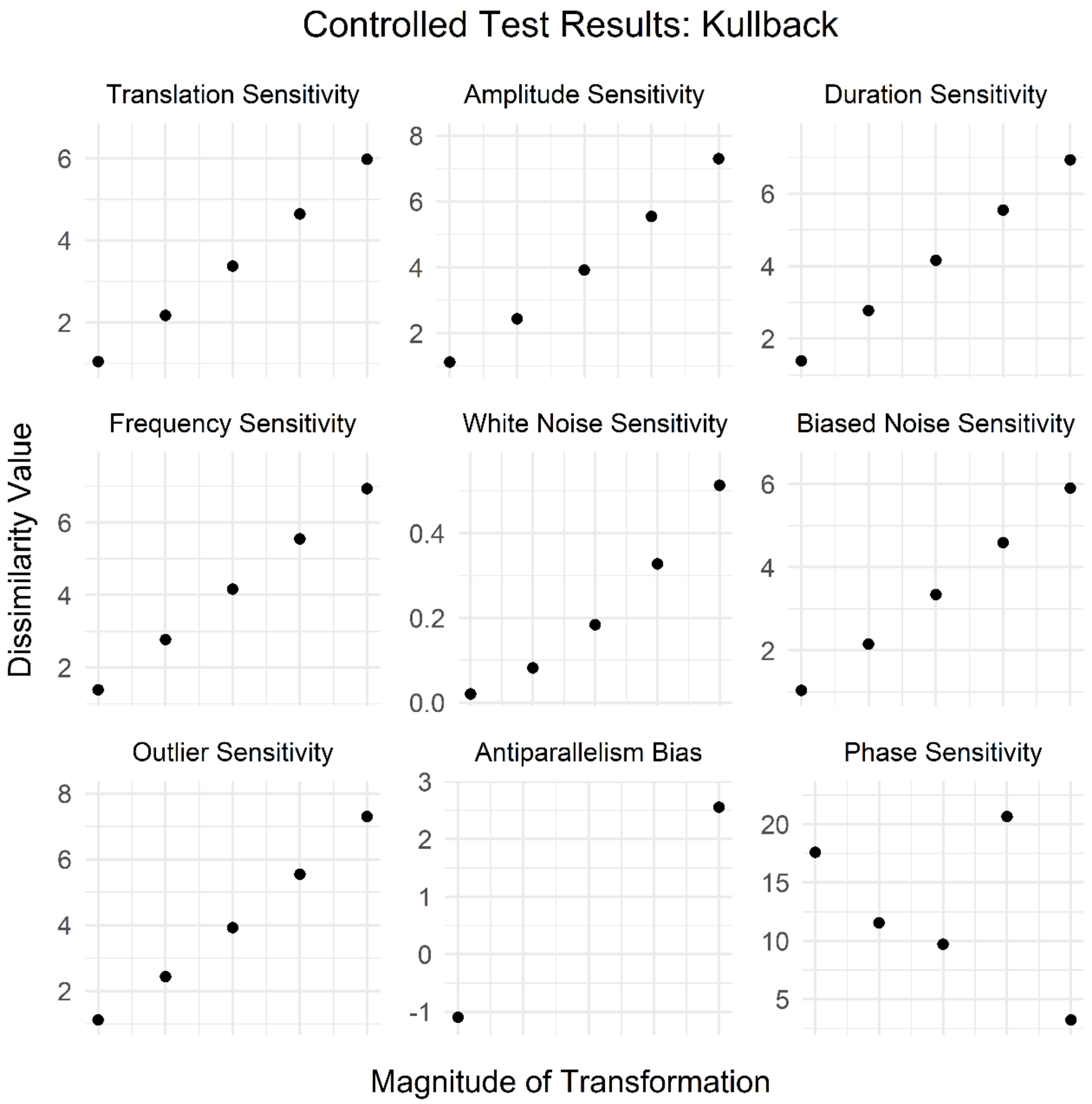

Fig. S25. Controlled testing results for the Kullback-Leibler Divergence. Sensitivities were tested by comparing time series with linearly increasing differences in summed y-axis values (or phase), against a reference time series. Antiparallelism bias was tested by comparing pairs of time series that differed by the same relative amount in different directions. The bias is neutral if the two values are identical, negative if the value on the left is higher, and positive if the value on the right is higher. Uniform time scaling sensitivity and warping sensitivity could not be tested for this distance measure, as it cannot measure unequal-length time series.

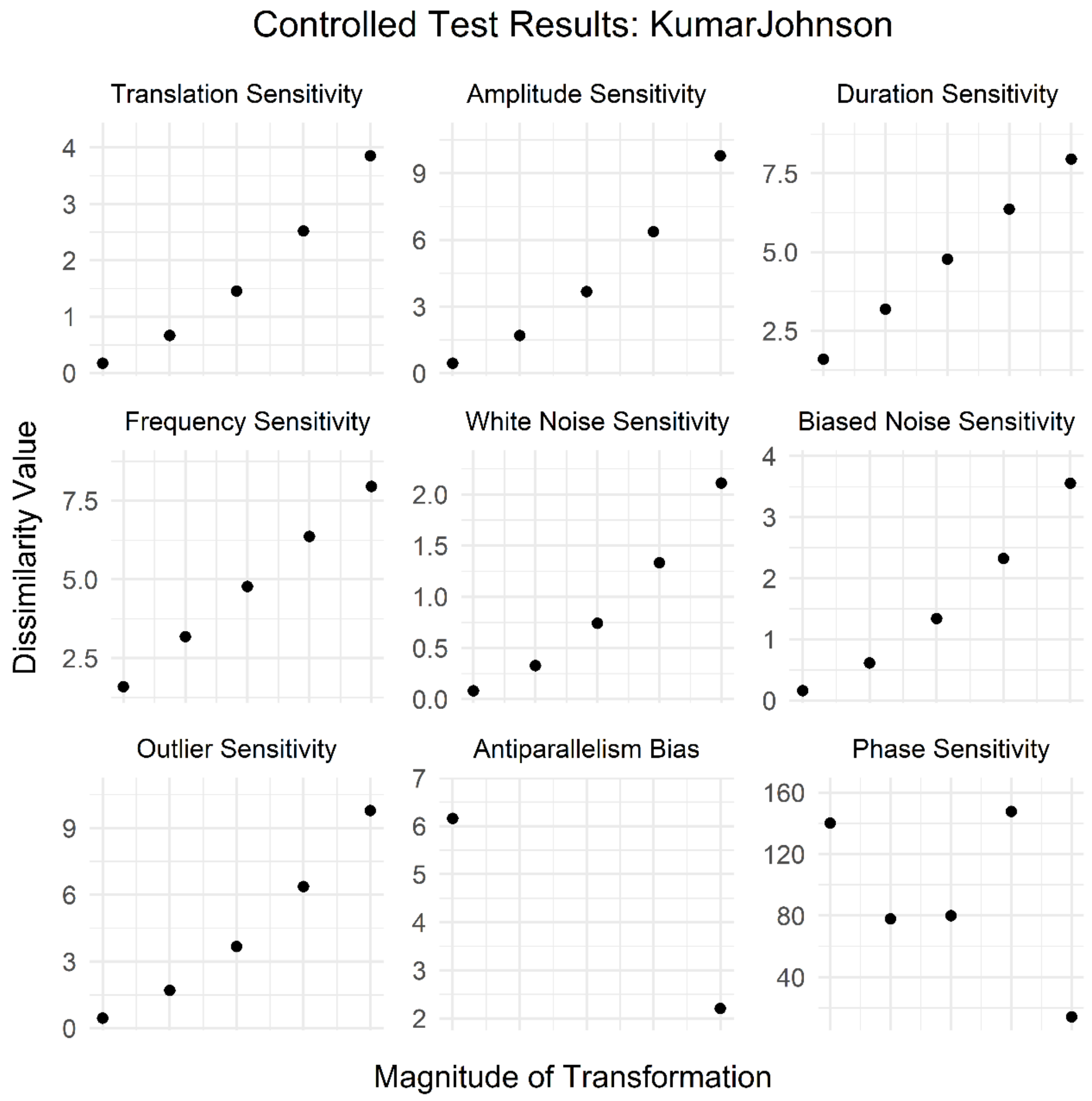

Fig. S26. Controlled testing results for the Kumar-Johnson Distance. Sensitivities were tested by comparing time series with linearly increasing differences in summed y-axis values (or phase), against a reference time series. Antiparallelism bias was tested by comparing pairs of time series that differed by the same relative amount in different directions. The bias is neutral if the two values are identical, negative if the value on the left is higher, and positive if the value on the right is higher. Uniform time scaling sensitivity and warping sensitivity could not be tested for this distance measure, as it cannot measure unequal-length time series.

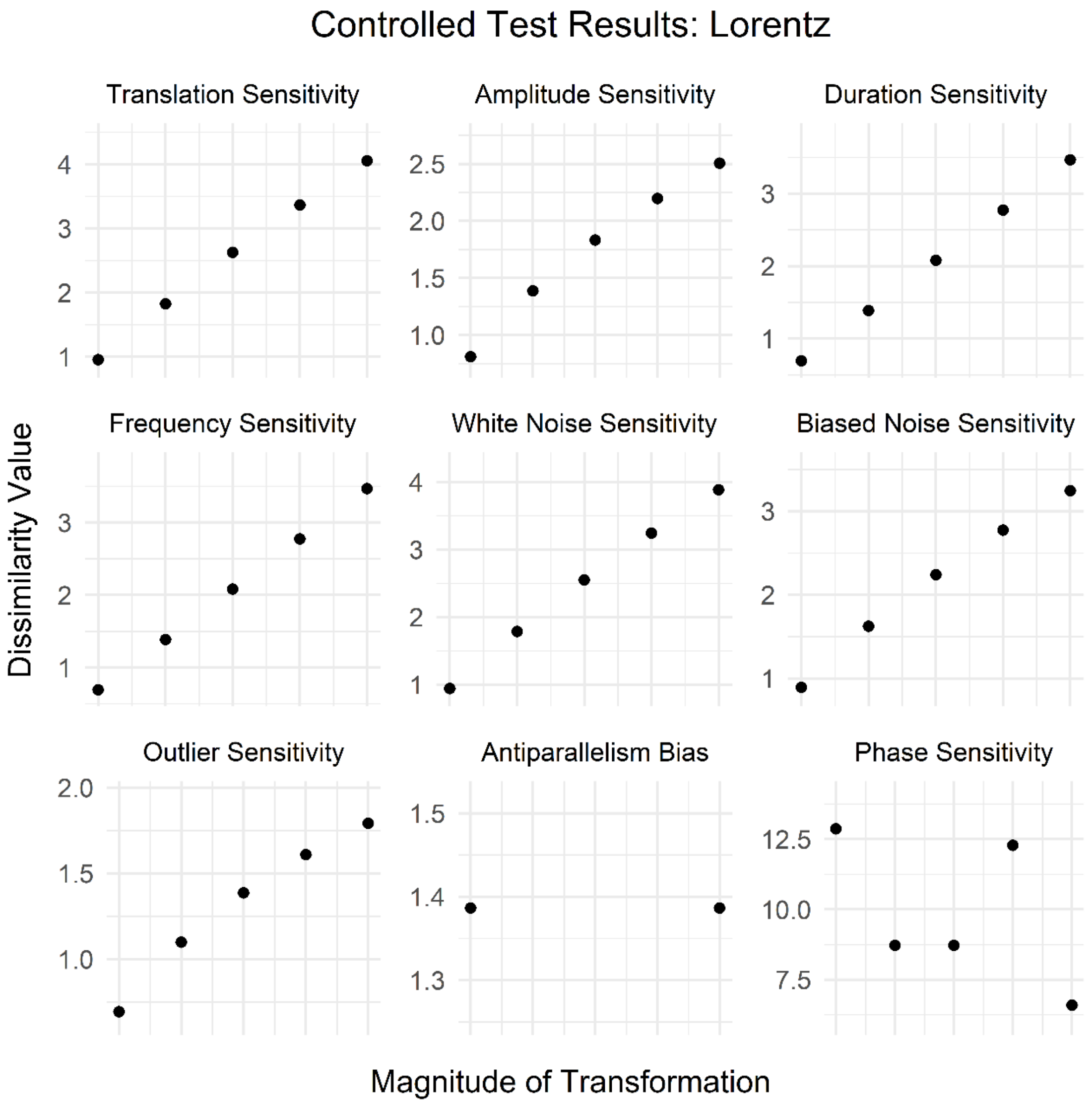

Fig. S27. Controlled testing results for the Lorentzian Distance. Sensitivities were tested by comparing time series with linearly increasing differences in summed y-axis values (or phase), against a reference time series. Antiparallelism bias was tested by comparing pairs of time series that differed by the same relative amount in different directions. The bias is neutral if the two values are identical, negative if the value on the left is higher, and positive if the value on the right is higher. Uniform time scaling sensitivity and warping sensitivity could not be tested for this distance measure, as it cannot measure unequal-length time series.

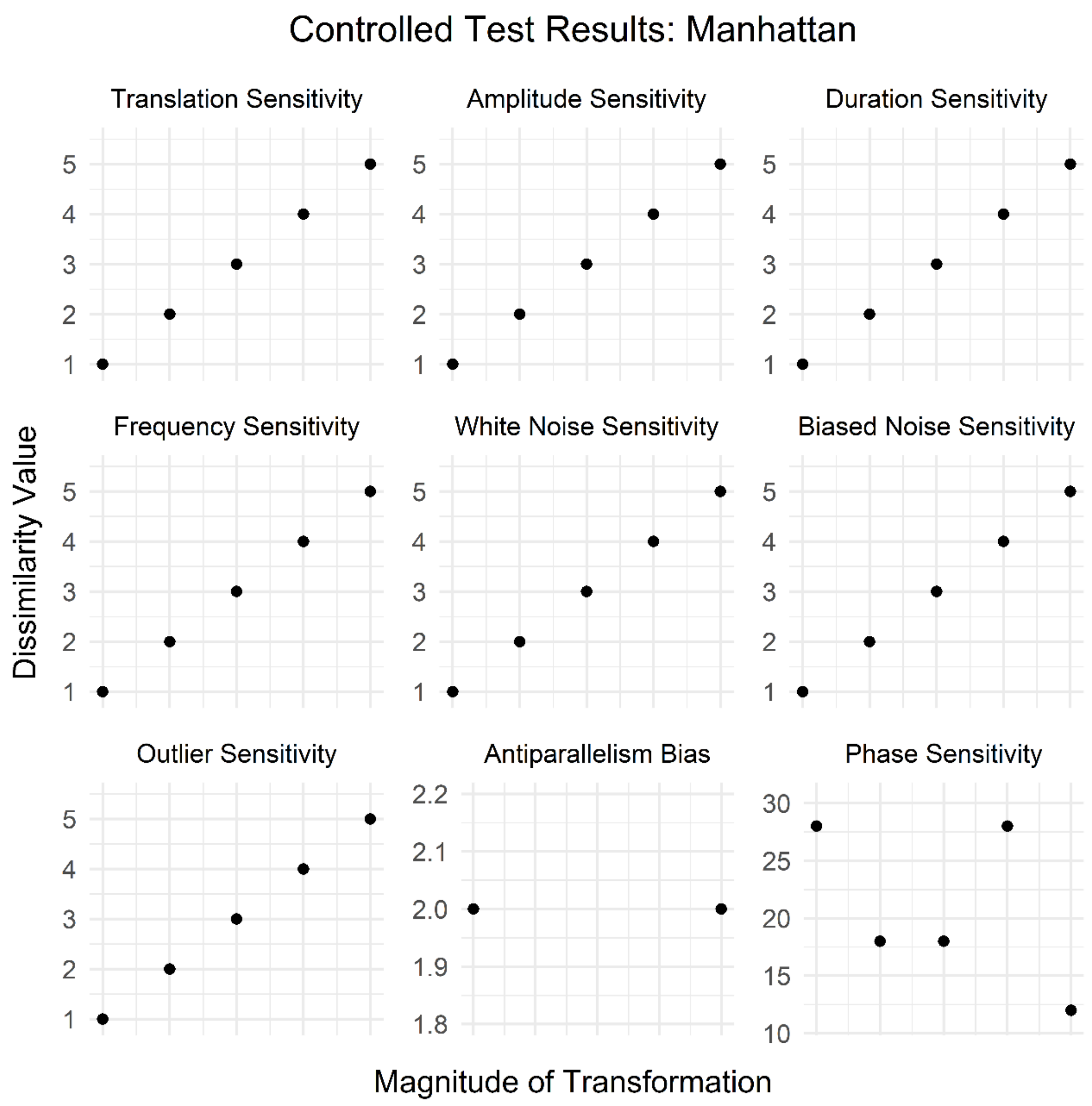

Fig. S28. Controlled testing results for the Manhattan Distance. Sensitivities were tested by comparing time series with linearly increasing differences in summed y-axis values (or phase), against a reference time series. Antiparallelism bias was tested by comparing pairs of time series that differed by the same relative amount in different directions. The bias is neutral if the two values are identical, negative if the value on the left is higher, and positive if the value on the right is higher. Uniform time scaling sensitivity and warping sensitivity could not be tested for this distance measure, as it cannot measure unequal-length time series.

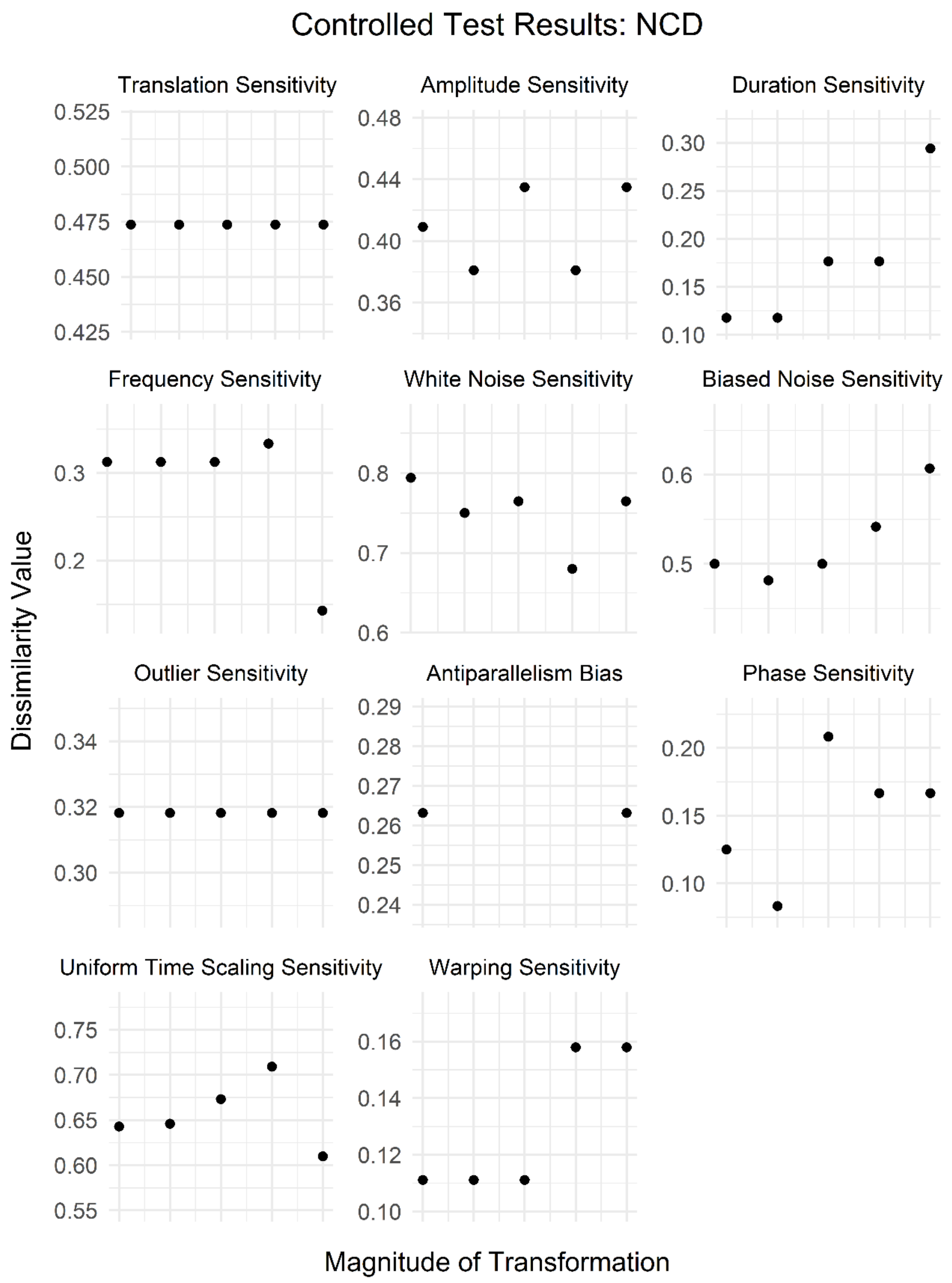

Fig. S29. Controlled testing results for the Normalized Compression Distance. Sensitivities were tested by comparing time series with linearly increasing differences in summed y-axis values (or phase), against a reference time series. Antiparallelism bias was tested by comparing pairs of time series that differed by the same relative amount in different directions. The bias is neutral if the two values are identical, negative if the value on the left is higher, and positive if the value on the right is higher. Uniform time scaling sensitivity and warping sensitivity were tested by stretching time series, or parts of time series, respectively, by different amounts.

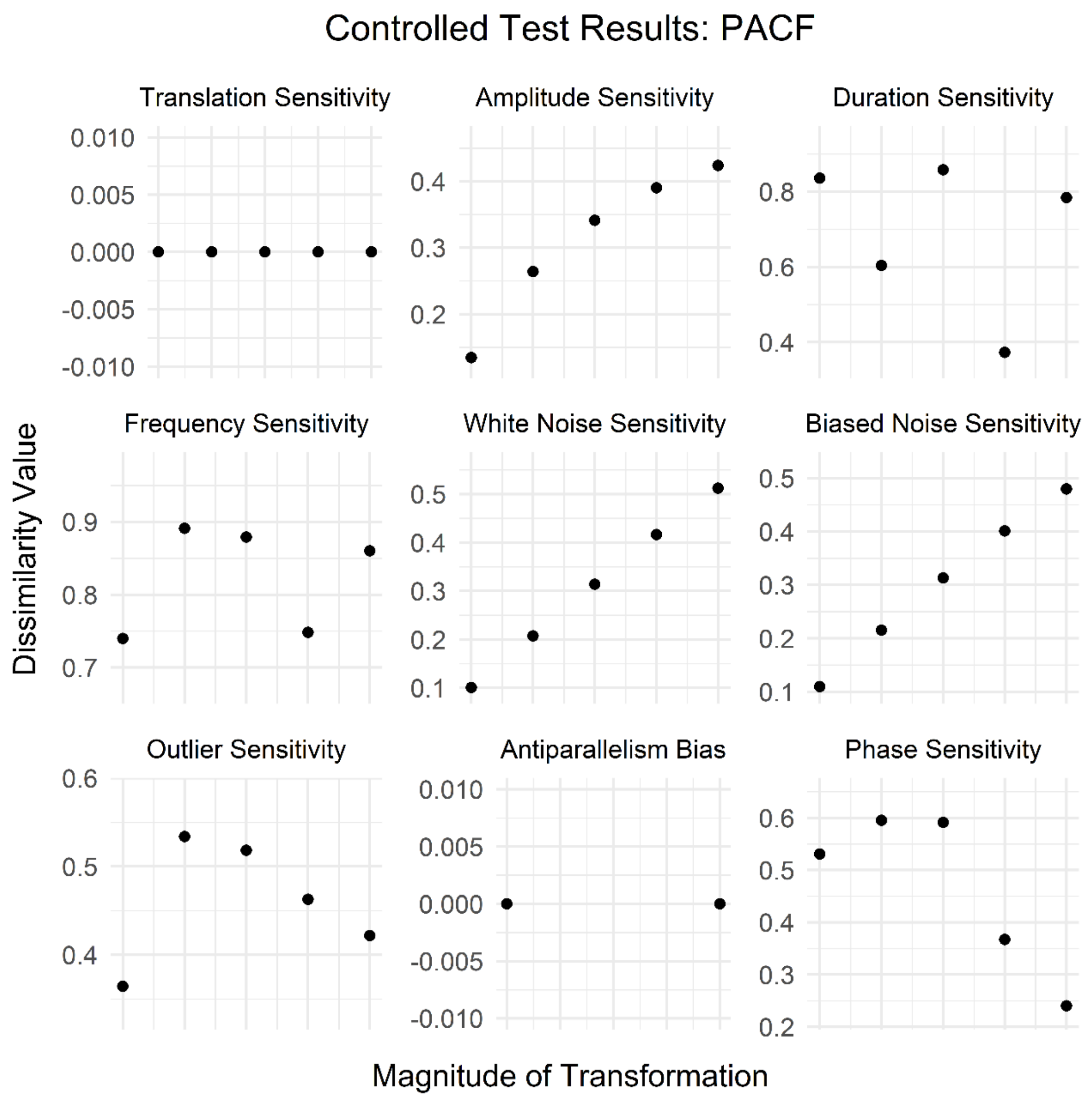

Fig. S30. Controlled testing results for the Partial Autocorrelation-Based Dissimilarity. Sensitivities were tested by comparing time series with linearly increasing differences in summed y-axis values (or phase), against a reference time series. Antiparallelism bias was tested by comparing pairs of time series that differed by the same relative amount in different directions. The bias is neutral if the two values are identical, negative if the value on the left is higher, and positive if the value on the right is higher. Uniform time scaling sensitivity and warping sensitivity could not be tested for this distance measure, as it cannot measure unequal-length time series.

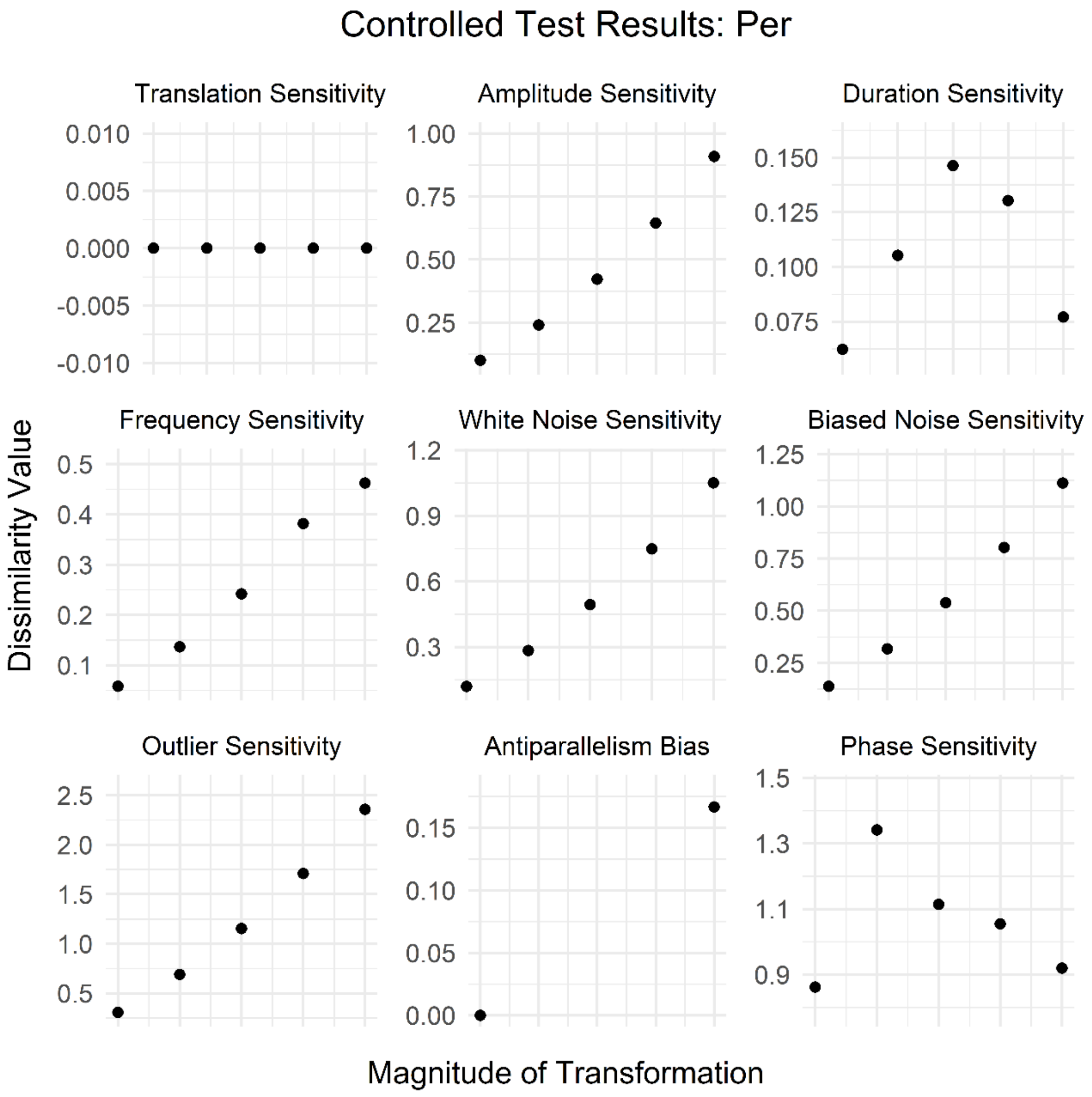

Fig. S31. Controlled testing results for the Periodogram-Based Dissimilarity. Sensitivities were tested by comparing time series with linearly increasing differences in summed y-axis values (or phase), against a reference time series. Antiparallelism bias was tested by comparing pairs of time series that differed by the same relative amount in different directions. The bias is neutral if the two values are identical, negative if the value on the left is higher, and positive if the value on the right is higher. Uniform time scaling sensitivity and warping sensitivity could not be tested for this distance measure, as it cannot measure unequal-length time series.

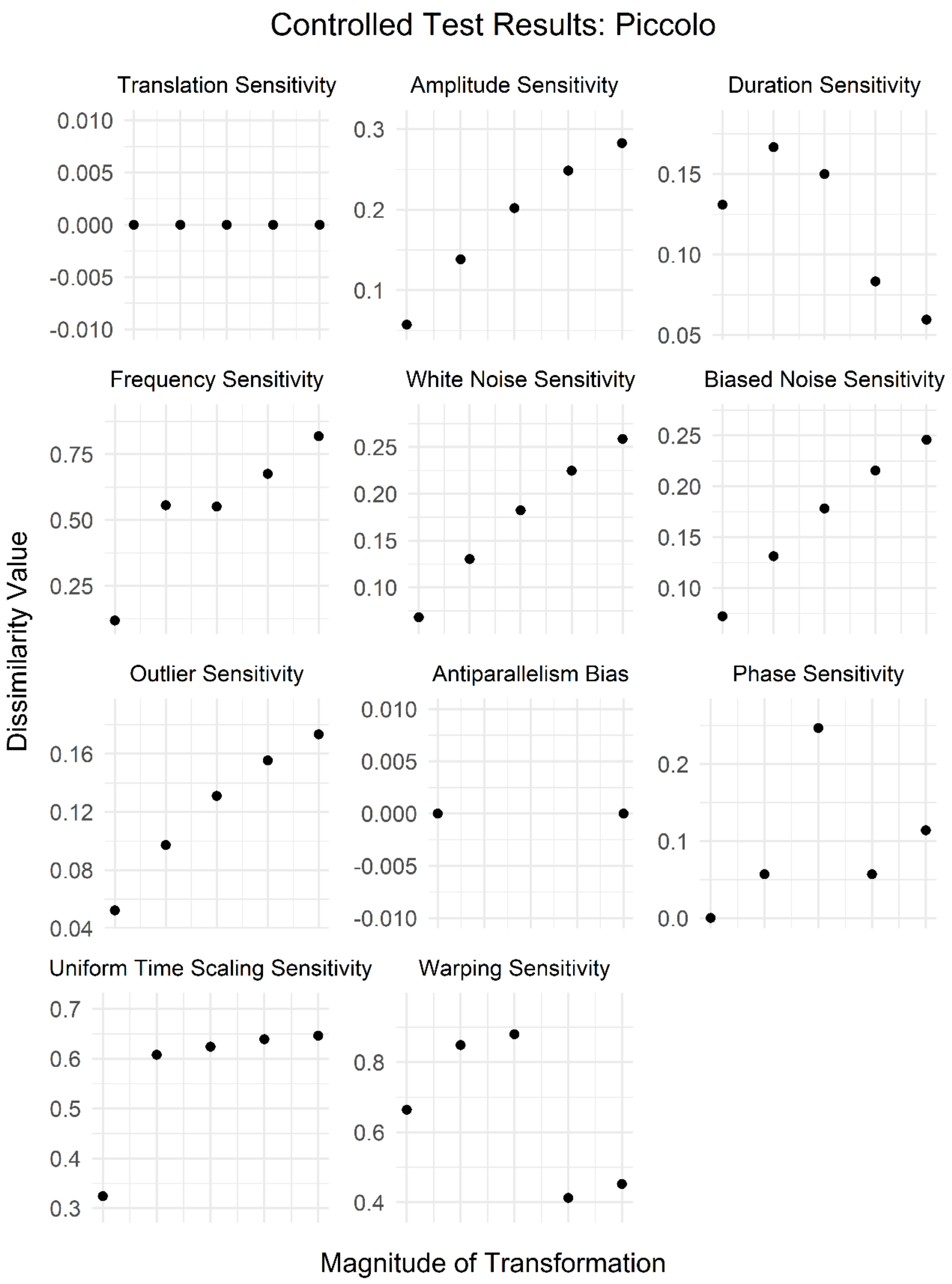

Fig. S32. Controlled testing results for the Piccolo Distance. Sensitivities were tested by comparing time series with linearly increasing differences in summed y-axis values (or phase), against a reference time series. Antiparallelism bias was tested by comparing pairs of time series that differed by the same relative amount in different directions. The bias is neutral if the two values are identical, negative if the value on the left is higher, and positive if the value on the right is higher. Uniform time scaling sensitivity and warping sensitivity were tested by stretching time series, or parts of time series, respectively, by different amounts.

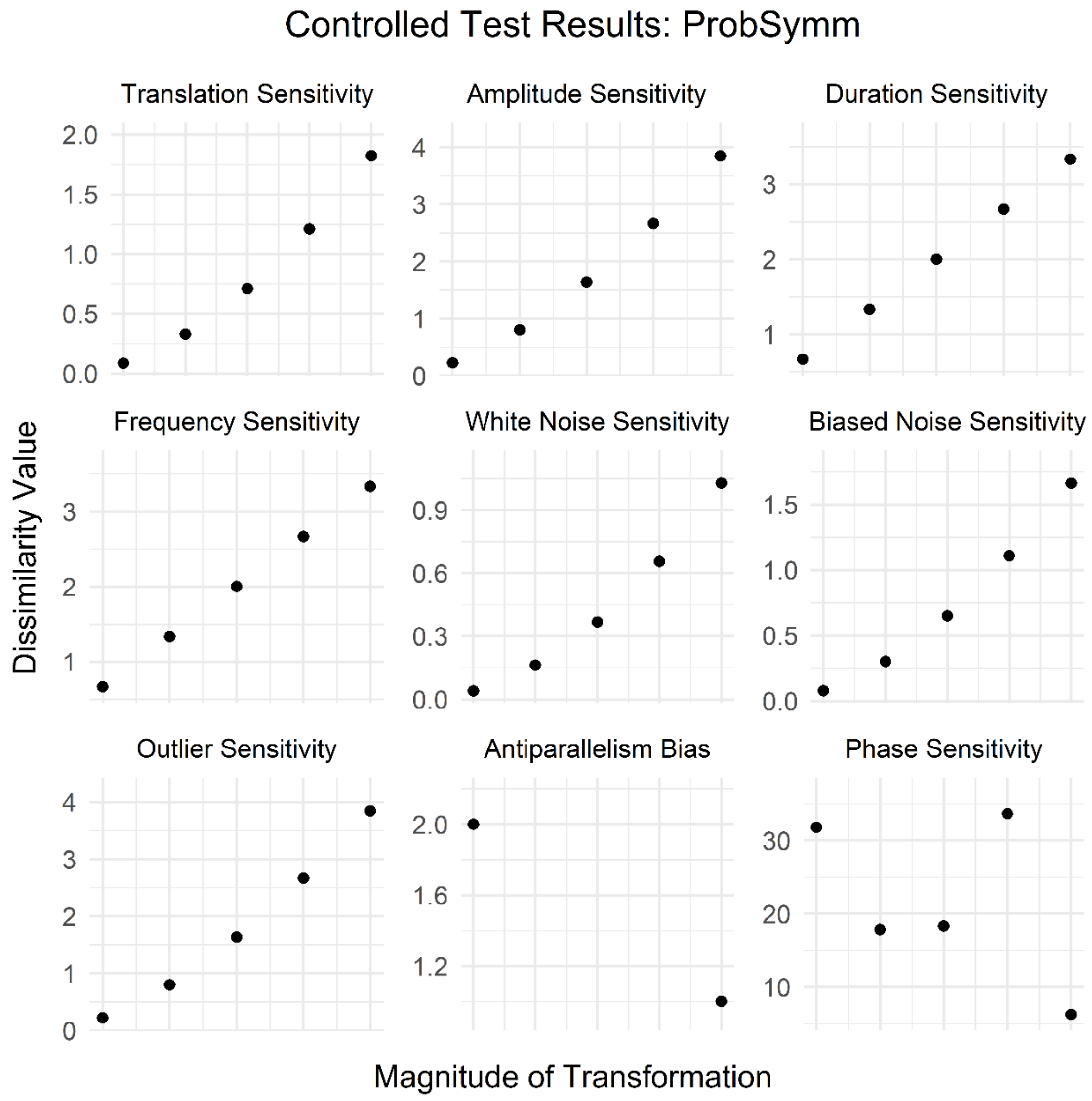

Fig. S33. Controlled testing results for the Probabilistic Symmetric Chi-Squared Distance. Sensitivities were tested by comparing time series with linearly increasing differences in summed y-axis values (or phase), against a reference time series. Antiparallelism bias was tested by comparing pairs of time series that differed by the same relative amount in different directions. The bias is neutral if the two values are identical, negative if the value on the left is higher, and positive if the value on the right is higher. Uniform time scaling sensitivity and warping sensitivity could not be tested for this distance measure, as it cannot measure unequal-length time series.

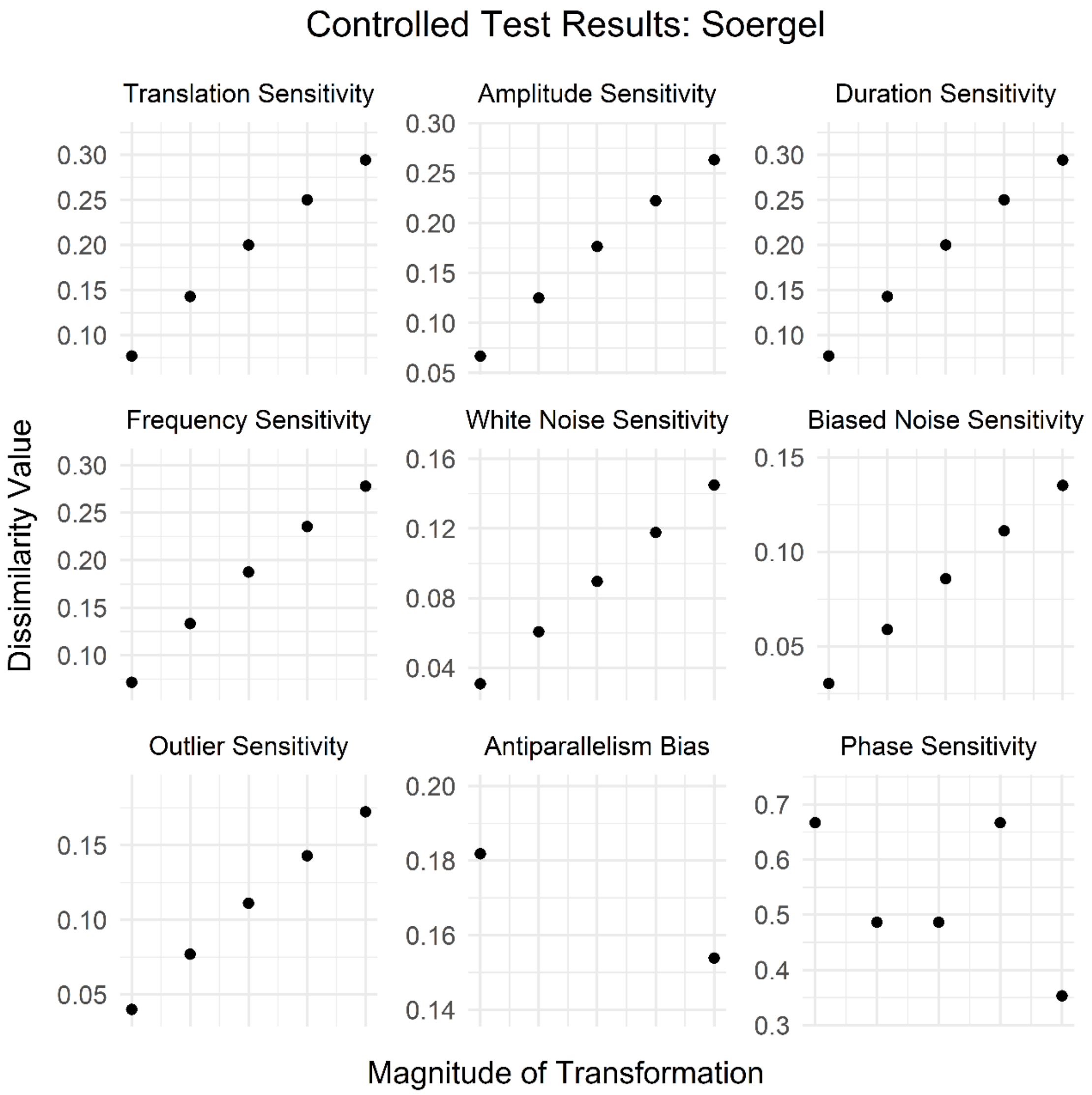

Fig. S34. Controlled testing results for the Soergel Distance. Sensitivities were tested by comparing time series with linearly increasing differences in summed y-axis values (or phase), against a reference time series. Antiparallelism bias was tested by comparing pairs of time series that differed by the same relative amount in different directions. The bias is neutral if the two values are identical, negative if the value on the left is higher, and positive if the value on the right is higher. Uniform time scaling sensitivity and warping sensitivity could not be tested for this distance measure, as it cannot measure unequal-length time series.

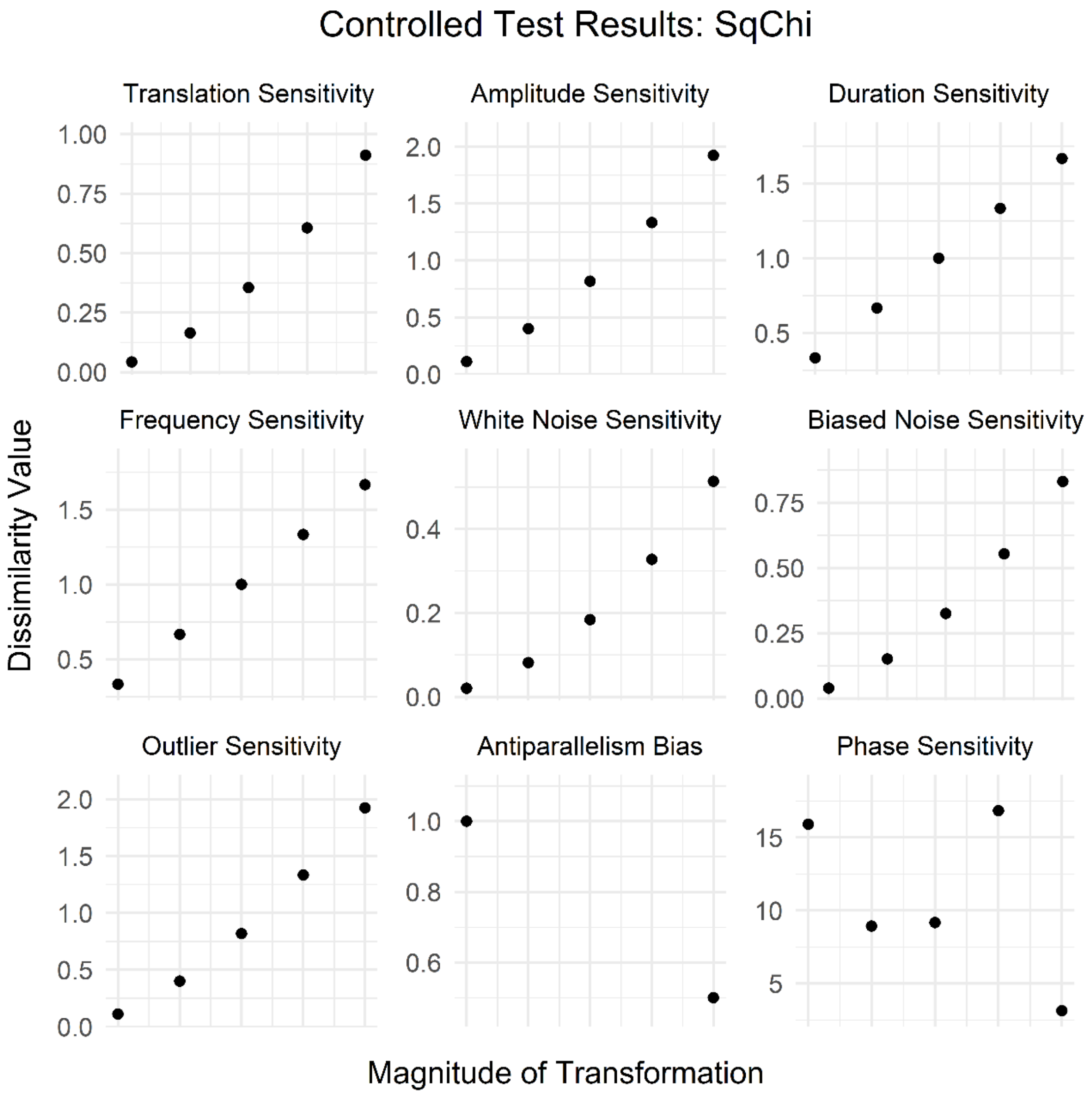

Fig. S35. Controlled testing results for the Squared Chi-Squared Distance. Sensitivities were tested by comparing time series with linearly increasing differences in summed y-axis values (or phase), against a reference time series. Antiparallelism bias was tested by comparing pairs of time series that differed by the same relative amount in different directions. The bias is neutral if the two values are identical, negative if the value on the left is higher, and positive if the value on the right is higher. Uniform time scaling sensitivity and warping sensitivity could not be tested for this distance measure, as it cannot measure unequal-length time series.

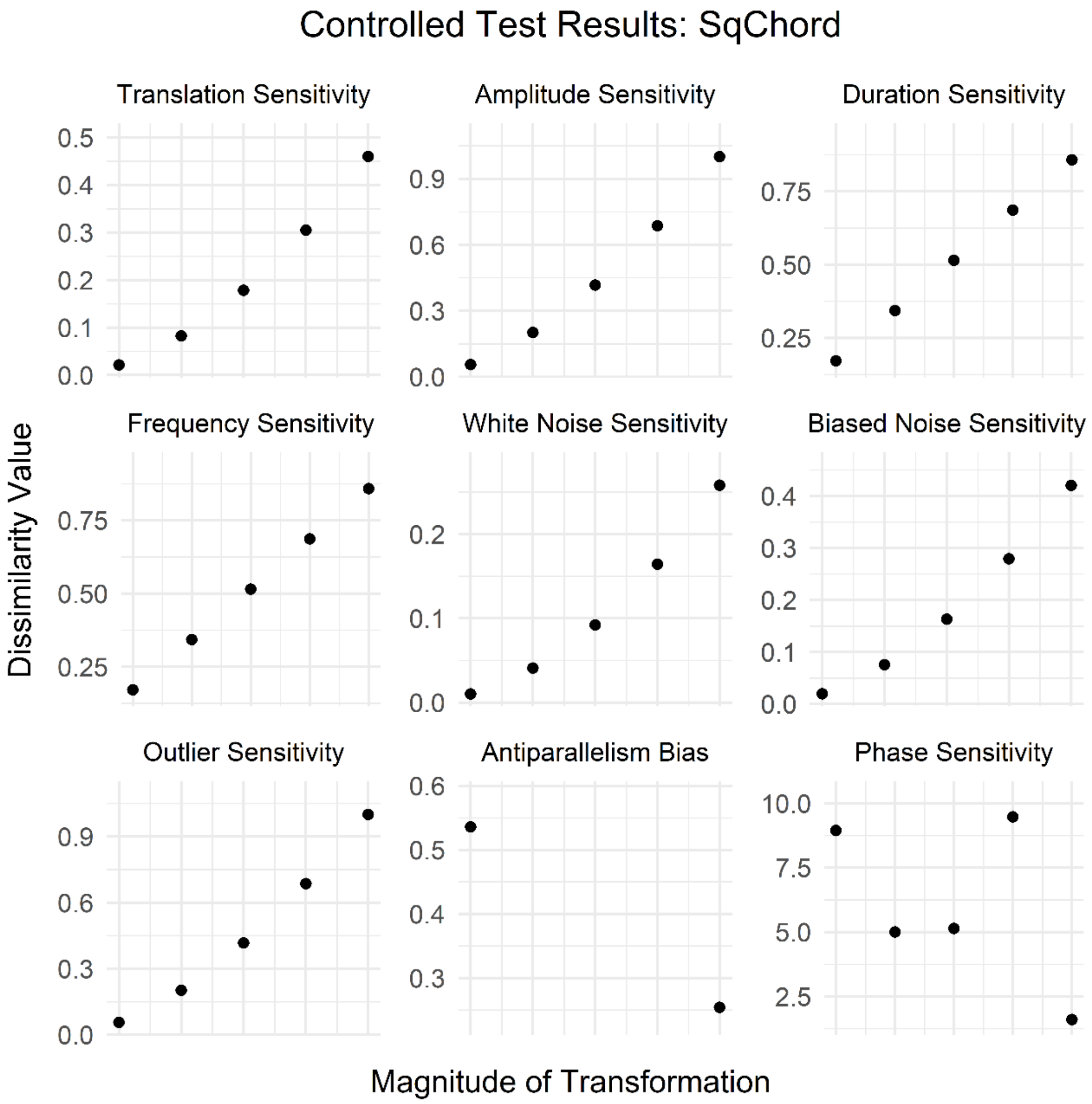

Fig. S36. Controlled testing results for the Squared-Chord Distance. Sensitivities were tested by comparing time series with linearly increasing differences in summed y-axis values (or phase), against a reference time series. Antiparallelism bias was tested by comparing pairs of time series that differed by the same relative amount in different directions. The bias is neutral if the two values are identical, negative if the value on the left is higher, and positive if the value on the right is higher. Uniform time scaling sensitivity and warping sensitivity could not be tested for this distance measure, as it cannot measure unequal-length time series.

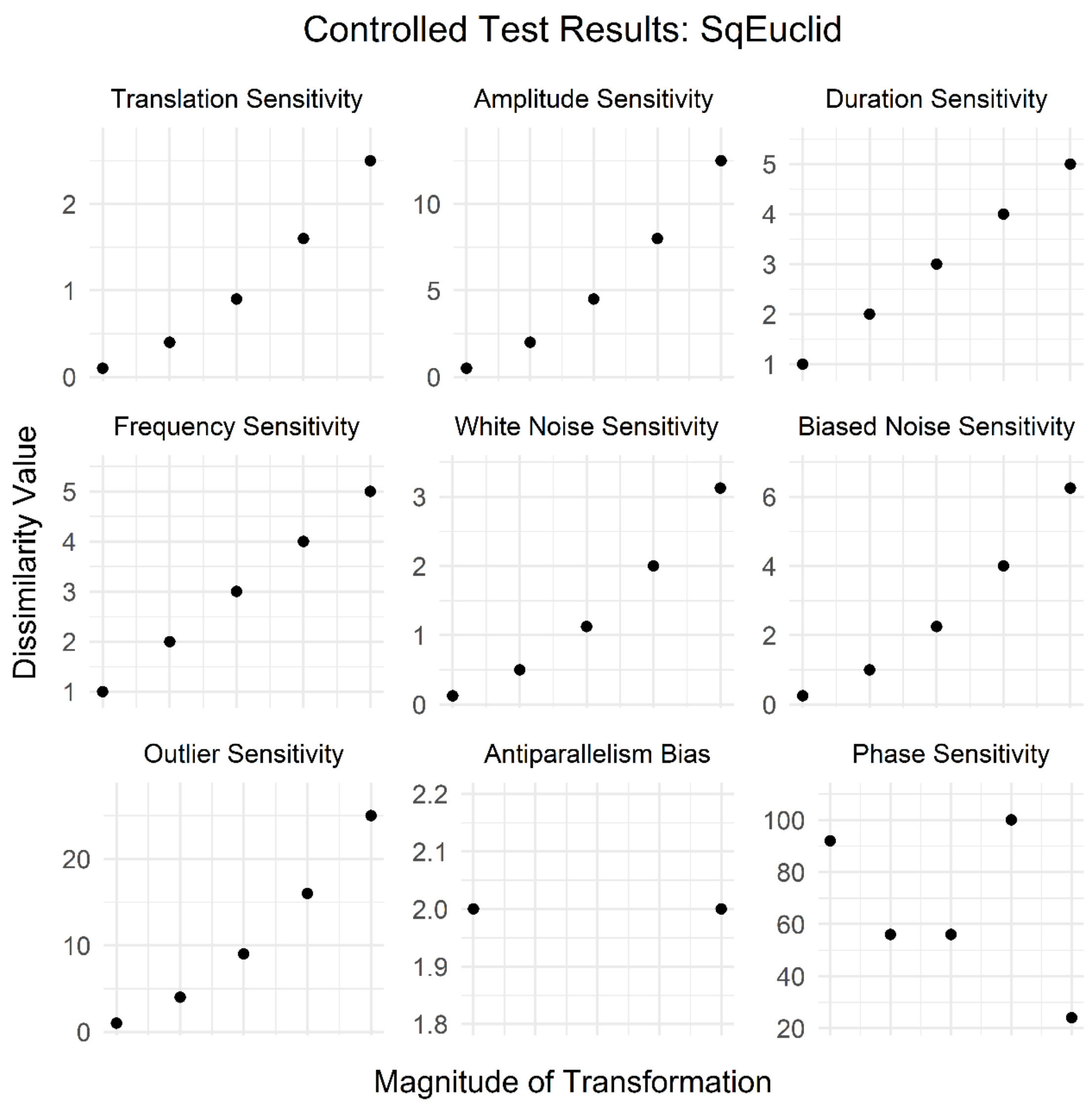

Fig. S37. Controlled testing results for the Squared Euclidean Distance. Sensitivities were tested by comparing time series with linearly increasing differences in summed y-axis values (or phase), against a reference time series. Antiparallelism bias was tested by comparing pairs of time series that differed by the same relative amount in different directions. The bias is neutral if the two values are identical, negative if the value on the left is higher, and positive if the value on the right is higher. Uniform time scaling sensitivity and warping sensitivity could not be tested for this distance measure, as it cannot measure unequal-length time series.

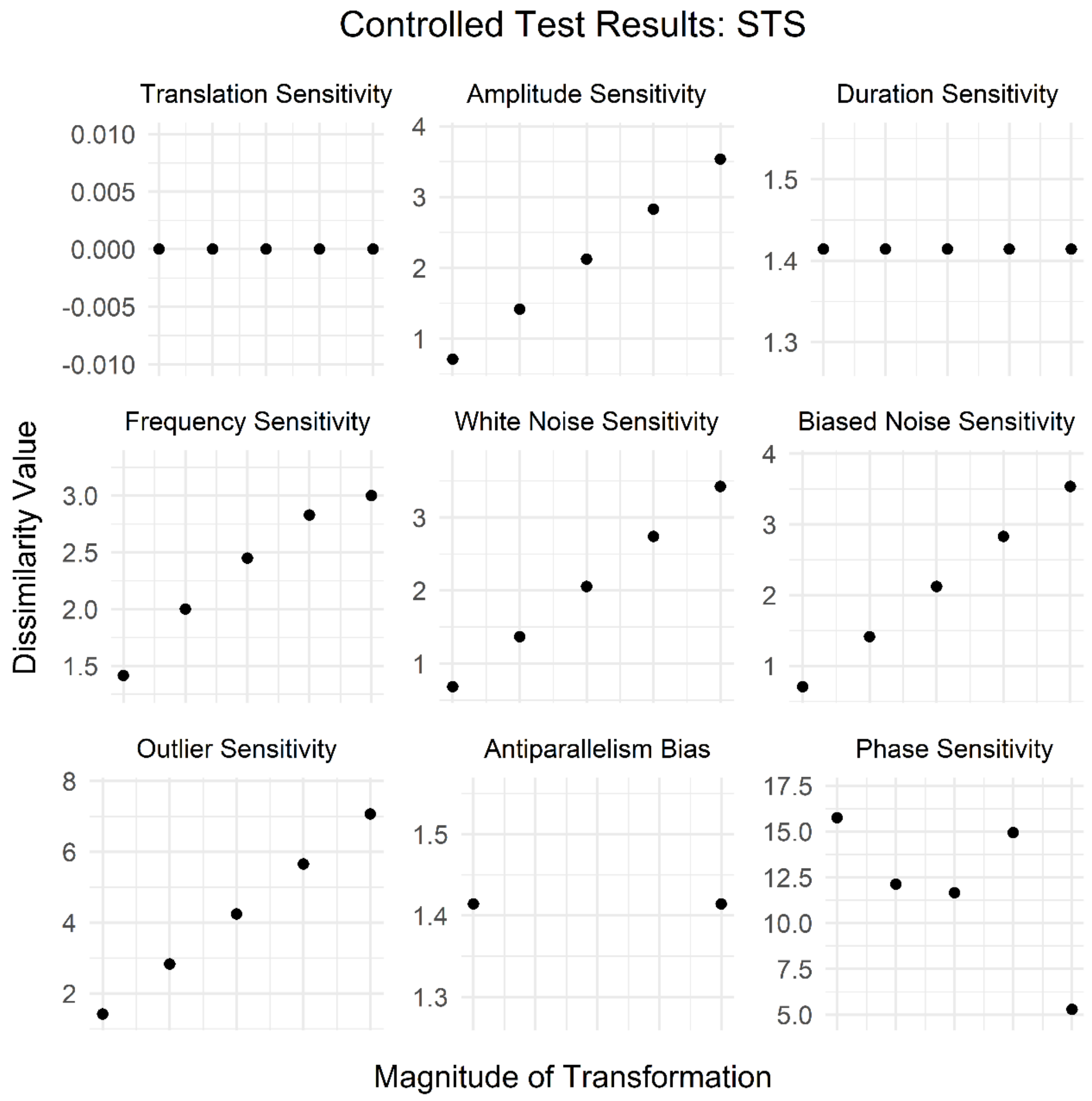

Fig. S38. Controlled testing results for the Short Time Series Distance. Sensitivities were tested by comparing time series with linearly increasing differences in summed y-axis values (or phase), against a reference time series. Antiparallelism bias was tested by comparing pairs of time series that differed by the same relative amount in different directions. The bias is neutral if the two values are identical, negative if the value on the left is higher, and positive if the value on the right is higher. Uniform time scaling sensitivity and warping sensitivity could not be tested for this distance measure, as it cannot measure unequal-length time series.

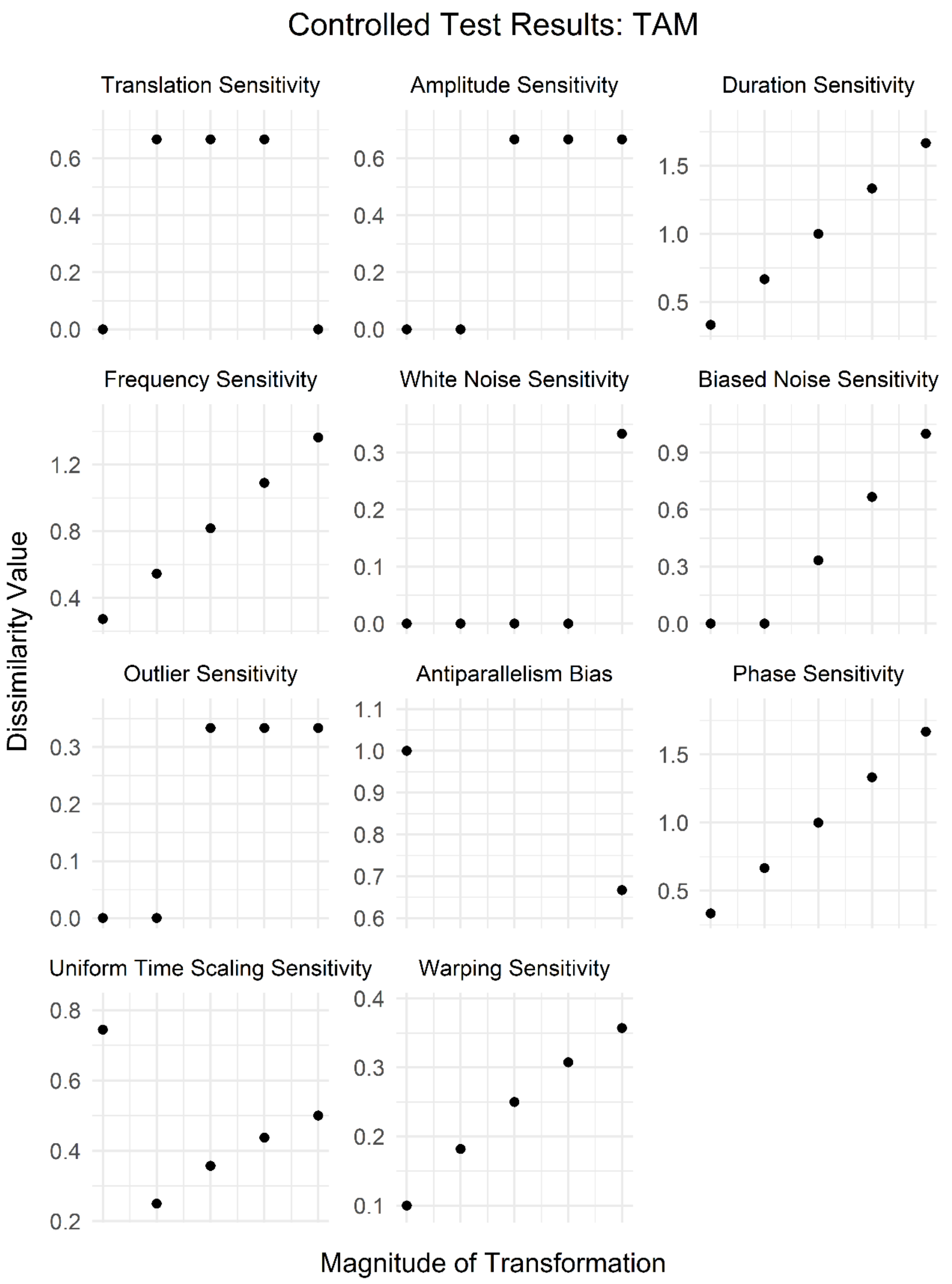

Fig. S39. Controlled testing results for the Time Alignment Measurement Distance. Sensitivities were tested by comparing time series with linearly increasing differences in summed y-axis values (or phase), against a reference time series. Antiparallelism bias was tested by comparing pairs of time series that differed by the same relative amount in different directions. The bias is neutral if the two values are identical, negative if the value on the left is higher, and positive if the value on the right is higher. Uniform time scaling sensitivity and warping sensitivity were tested by stretching time series, or parts of time series, respectively, by different amounts.

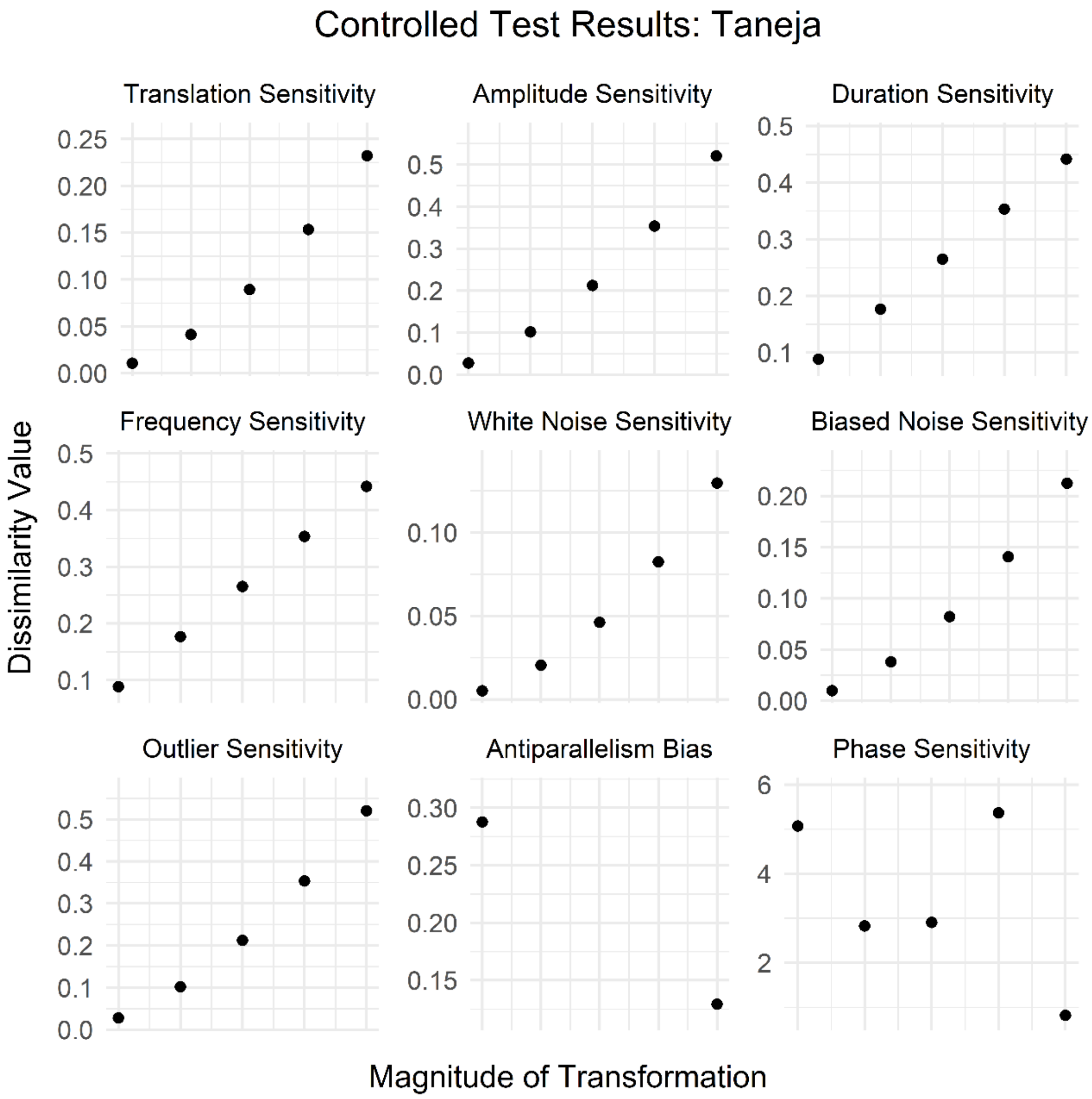

Fig. S40. Controlled testing results for the Taneja Difference. Sensitivities were tested by comparing time series with linearly increasing differences in summed y-axis values (or phase), against a reference time series. Antiparallelism bias was tested by comparing pairs of time series that differed by the same relative amount in different directions. The bias is neutral if the two values are identical, negative if the value on the left is higher, and positive if the value on the right is higher. Uniform time scaling sensitivity and warping sensitivity could not be tested for this distance measure, as it cannot measure unequal-length time series.

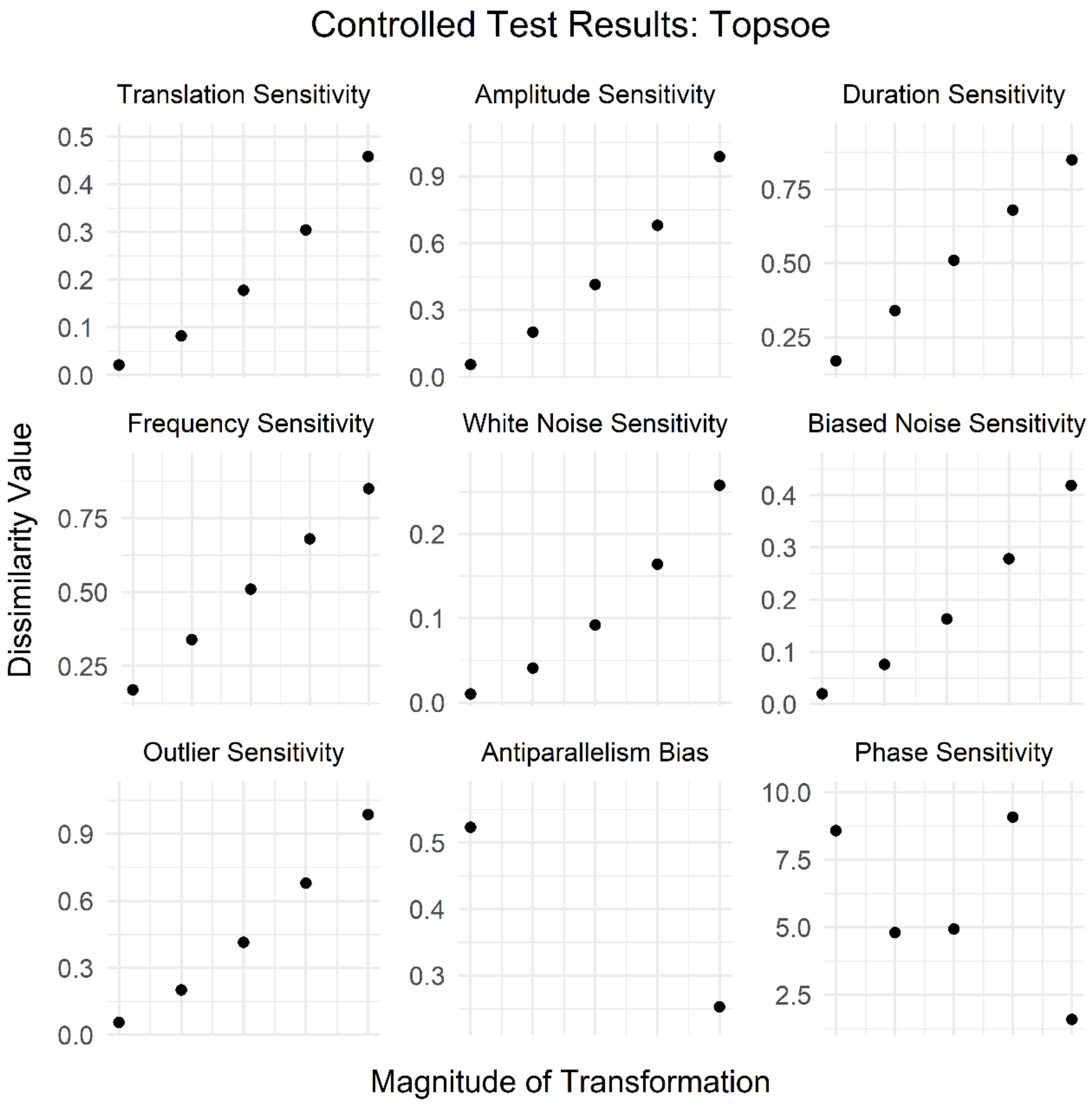

Fig. S41. Controlled testing results for the Topsoe Distance. Sensitivities were tested by comparing time series with linearly increasing differences in summed y-axis values (or phase), against a reference time series. Antiparallelism bias was tested by comparing pairs of time series that differed by the same relative amount in different directions. The bias is neutral if the two values are identical, negative if the value on the left is higher, and positive if the value on the right is higher. Uniform time scaling sensitivity and warping sensitivity could not be tested for this distance measure, as it cannot measure unequal-length time series.

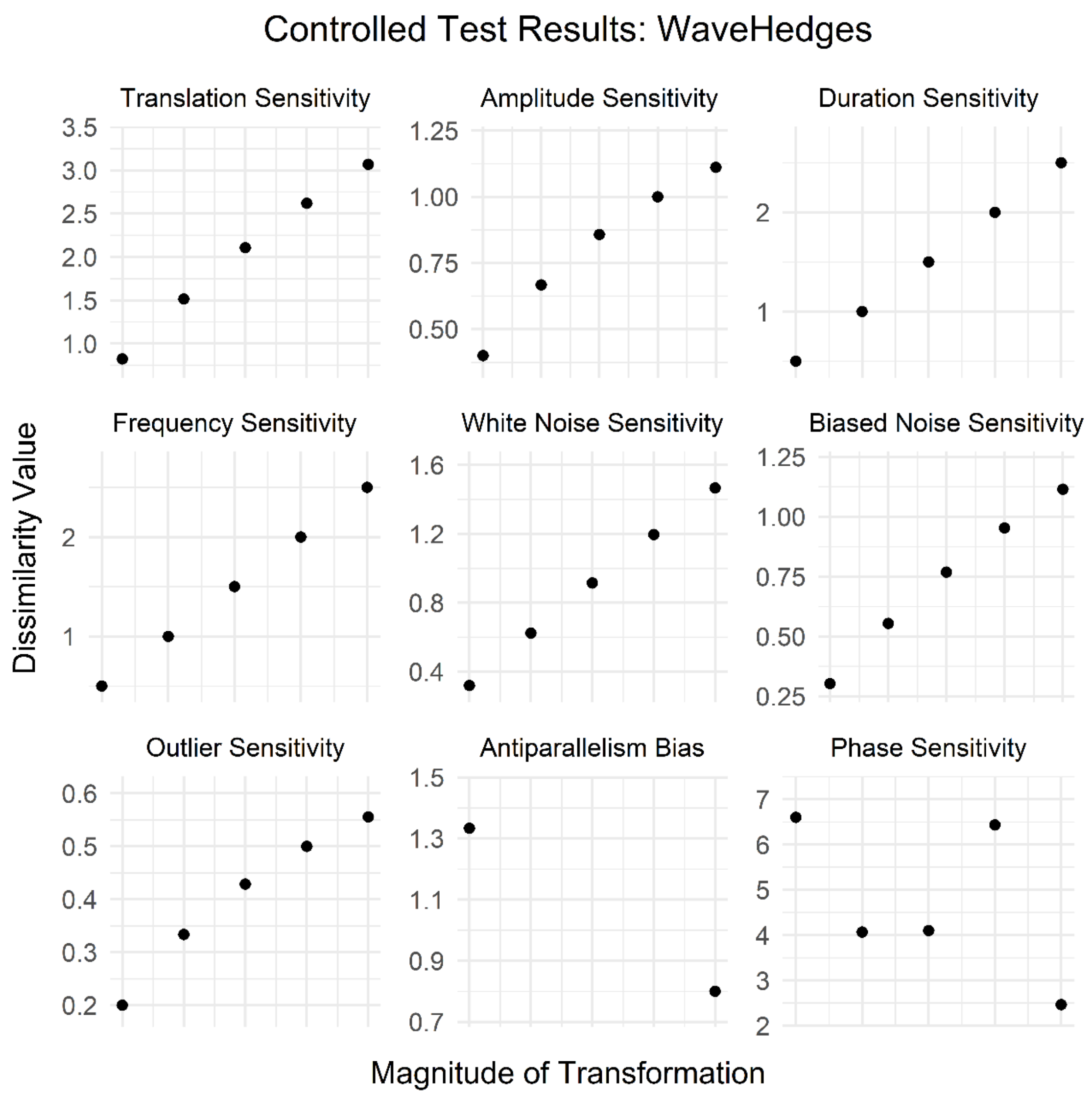

Fig. S42. Controlled testing results for the Wave-Hedges Distance. Sensitivities were tested by comparing time series with linearly increasing differences in summed y-axis values (or phase), against a reference time series. Antiparallelism bias was tested by comparing pairs of time series that differed by the same relative amount in different directions. The bias is neutral if the two values are identical, negative if the value on the left is higher, and positive if the value on the right is higher. Uniform time scaling sensitivity and warping sensitivity could not be tested for this distance measure, as it cannot measure unequal-length time series.

2. Tables of controlled test results for all distance measures

This section contains tables of controlled testing results for all 42 distance measures we tested. Each figure includes all time-based and values-based properties for which that distance measure gave results. Distance measures are presented in alphabetical order.

Table S1. Raw controlled testing results for the Autocorrelation-based Dissimilarity. For details on how testing was performed and how relative sensitivity ranges were calculated, see section S4. Uniform time scaling sensitivity and warping sensitivity could not be tested for this distance measure, as it cannot measure unequal-length time series.

Controlled Test Results: ACF

Test	Res.1	Res.2	Res.3	Res.4	Res.5	Res.6	Res.7
Reflexivity	0.0000						
Symmetry	0.9611	0.9611					
Translation Sensitivity	0.0000	0.0000	0.0000	0.0000	0.0000		
Amplitude Sensitivity	0.0941	0.2352	0.3529	0.4428	0.5114		
Duration Sensitivity	0.4355	0.6796	0.6374	0.4965	0.4381		
Frequency Sensitivity	0.5277	0.8189	1.0761	1.4041	1.8403		
White Noise Sensitivity	0.0393	0.0902	0.1510	0.2196	0.2939		
Biased Noise Sensitivity	0.0485	0.1042	0.1651	0.2294	0.2953		
Outlier Sensitivity	0.1320	0.2021	0.2508	0.2910	0.3254		
Antiparallelism Bias	0.0000	0.0000					
Phase Sensitivity	0.3938	0.7610	1.0553	0.3934	0.2057		
Uniform Time Scaling Sensitivity							
Warping Sensitivity							
Non-positive Value Handling	0.6900	0.6900	0.8552				
Non-negativity	1.0000						
Triangle Inequality	1.0000						
Relative Sensitivity Ranges	0.0000	1.0946	0.6401	3.4429	0.6678	0.6473	0.5073

Table S2. Raw controlled testing results for the Additive Symmetric Chi-Squared Distance. For details on how testing was performed and how relative sensitivity ranges were calculated, see section S4. Uniform time scaling sensitivity and warping sensitivity could not be tested for this distance measure, as it cannot measure unequal-length time series.

Controlled Test Results: Additive

Test	Res.1	Res.2	Res.3	Res.4	Res.5	Res.6	Res.7
Reflexivity	0.00						
Symmetry	42.74	42.74					
Translation Sensitivity	0.17	0.66	1.44	2.49	3.78		
Amplitude Sensitivity	0.45	1.67	3.54	6.00	9.03		
Duration Sensitivity	1.50	3.00	4.50	6.00	7.50		
Frequency Sensitivity	1.50	3.00	4.50	6.00	7.50		
White Noise Sensitivity	0.08	0.33	0.74	1.32	2.09		
Biased Noise Sensitivity	0.16	0.61	1.33	2.29	3.48		
Outlier Sensitivity	0.45	1.67	3.54	6.00	9.03		
Antiparallelism Bias	5.33	2.13					
Phase Sensitivity	106.90	59.40	61.00	112.90	13.60		
Uniform Time Scaling Sensitivity							
Warping Sensitivity							
Non-positive Value Handling	3,999,998.00	0.00	-4.50				
Non-negativity	0.00						
Triangle Inequality	0.00						
Relative Sensitivity Ranges	0.66	1.58	1.10	1.10	0.37	0.61	1.58

Table S3. Raw controlled testing results for the Average Distance. For details on how testing was performed and how relative sensitivity ranges were calculated, see section S4. Uniform time scaling sensitivity and warping sensitivity could not be tested for this distance measure, as it cannot measure unequal-length time series.

Controlled Test Results: AVG

Test	Res.1	Res.2	Res.3	Res.4	Res.5	Res.6	Res.7
Reflexivity	0.000						
Symmetry	7.900	7.900					
Translation Sensitivity	0.550	1.100	1.650	2.200	2.750		
Amplitude Sensitivity	0.750	1.500	2.250	3.000	3.750		
Duration Sensitivity	1.000	1.500	2.000	2.500	3.000		
Frequency Sensitivity	1.000	1.500	2.000	2.500	3.000		
White Noise Sensitivity	0.562	1.125	1.688	2.250	2.812		
Biased Noise Sensitivity	0.625	1.250	1.875	2.500	3.125		
Outlier Sensitivity	1.000	2.000	3.000	4.000	5.000		
Antiparallelism Bias	1.500	1.500					
Phase Sensitivity	16.500	11.500	11.500	16.500	7.000		
Uniform Time Scaling Sensitivity							
Warping Sensitivity							
Non-positive Value Handling	2.000	2.000	3.000				
Non-negativity	1.000						
Triangle Inequality	1.000						
Relative Sensitivity Ranges	0.858	1.170	0.780	0.780	0.877	0.975	1.560

Table S4. Raw controlled testing results for the Canberra Distance. For details on how testing was performed and how relative sensitivity ranges were calculated, see section S4. Uniform time scaling sensitivity and warping sensitivity could not be tested for this distance measure, as it cannot measure unequal-length time series.

Controlled Test Results: Canb

Test	Res.1	Res.2	Res.3	Res.4	Res.5	Res.6	Res.7
Reflexivity	0.0000						
Symmetry	4.6030	4.6030					
Translation Sensitivity	0.4297	0.8225	1.1830	1.5152	1.8222		
Amplitude Sensitivity	0.2222	0.4000	0.5455	0.6667	0.7692		
Duration Sensitivity	0.3333	0.6667	1.0000	1.3333	1.6667		
Frequency Sensitivity	0.3333	0.6667	1.0000	1.3333	1.6667		
White Noise Sensitivity	0.1633	0.3265	0.4903	0.6553	0.8222		
Biased Noise Sensitivity	0.1585	0.3022	0.4334	0.5538	0.6649		
Outlier Sensitivity	0.1111	0.2000	0.2727	0.3333	0.3846		
Antiparallelism Bias	1.0000	0.5000					
Phase Sensitivity	5.1143	3.0032	3.0528	5.0810	1.5667		
Uniform Time Scaling Sensitivity							
Warping Sensitivity							
Non-positive Value Handling	1.0000	1.0000	3.0000				
Non-negativity	0.0000						
Triangle Inequality	0.0000						
Relative Sensitivity Ranges	1.6125	0.6334	1.5440	1.5440	0.7630	0.5864	0.3167

Table S5. Raw controlled testing results for the Compression-Based Dissimilarity Measure. For details on how testing was performed and how relative sensitivity ranges were calculated, see section S4. Uniform time scaling sensitivity and warping sensitivity could not be tested for this distance measure, as it cannot measure unequal-length time series.

Controlled Test Results: CDM

Test	Res.1	Res.2	Res.3	Res.4	Res.5	Res.6	Res.7
Reflexivity	0.5588						
Symmetry	0.7073	0.6829					
Translation Sensitivity	0.7222	0.7222	0.7222	0.7222	0.7222		
Amplitude Sensitivity	0.6667	0.6579	0.6750	0.6579	0.6750		
Duration Sensitivity	0.5588	0.5588	0.5882	0.5882	0.6250		
Frequency Sensitivity	0.6333	0.6333	0.6333	0.6552	0.5714		
White Noise Sensitivity	0.8793	0.8571	0.8621	0.8367	0.8621		
Biased Noise Sensitivity	0.7308	0.7255	0.7308	0.7609	0.7885		
Outlier Sensitivity	0.6591	0.6591	0.6591	0.6591	0.6591		
Antiparallelism Bias	0.6216	0.6216					
Phase Sensitivity	0.5532	0.5417	0.5870	0.5833	0.5833		
Uniform Time Scaling Sensitivity	0.7727	0.7639	0.7763	0.7975	0.7538		
Warping Sensitivity	0.5556	0.5556	0.5556	0.5676	0.5676		
Non-positive Value Handling	0.6923	0.6571	0.6571				
Non-negativity	1.0000						
Triangle Inequality	1.0000						
Relative Sensitivity Ranges	0.0000	0.3138	1.2139	1.5362	0.7810	1.1551	0.0000

Table S6. Raw controlled testing results for the Complexity-Invariant Distance. For details on how testing was performed and how relative sensitivity ranges were calculated, see section S4. Uniform time scaling sensitivity and warping sensitivity could not be tested for this distance measure, as it cannot measure unequal-length time series.

Controlled Test Results: CID

Test	Res.1	Res.2	Res.3	Res.4	Res.5	Res.6	Res.7
Reflexivity	0.000						
Symmetry	18.512	18.512					
Translation Sensitivity	0.316	0.632	0.949	1.265	1.581		
Amplitude Sensitivity	0.901	2.236	4.039	6.325	9.100		
Duration Sensitivity	1.000	1.414	1.732	2.000	2.236		
Frequency Sensitivity	1.414	2.449	3.464	4.472	5.244		
White Noise Sensitivity	0.373	0.796	1.284	1.846	2.492		
Biased Noise Sensitivity	0.533	1.148	1.865	2.697	3.654		
Outlier Sensitivity	1.254	3.117	5.669	8.944	12.956		
Antiparallelism Bias	1.414	4.243					
Phase Sensitivity	9.592	7.820	8.052	10.650	5.272		
Uniform Time Scaling Sensitivity							
Warping Sensitivity							
Non-positive Value Handling	3.464	3.464	7.036				
Non-negativity	1.000						
Triangle Inequality	0.000						
Relative Sensitivity Ranges	0.281	1.824	0.275	0.852	0.471	0.694	2.603

Table S7. Raw controlled testing results for the Clark Squared Distance. For details on how testing was performed and how relative sensitivity ranges were calculated, see section S4. Uniform time scaling sensitivity and warping sensitivity could not be tested for this distance measure, as it cannot measure unequal-length time series.

Controlled Test Results: Clark

Test	Res.1	Res.2	Res.3	Res.4	Res.5	Res.6	Res.7
Reflexivity	0.0000						
Symmetry	1.5951	1.5951					
Translation Sensitivity	0.1390	0.2658	0.3819	0.4886	0.5871		
Amplitude Sensitivity	0.1571	0.2828	0.3857	0.4714	0.5439		
Duration Sensitivity	0.3333	0.4714	0.5774	0.6667	0.7454		
Frequency Sensitivity	0.3333	0.4714	0.5774	0.6667	0.7454		
White Noise Sensitivity	0.0629	0.1256	0.1887	0.2525	0.3176		
Biased Noise Sensitivity	0.0881	0.1670	0.2383	0.3030	0.3622		
Outlier Sensitivity	0.1111	0.2000	0.2727	0.3333	0.3846		
Antiparallelism Bias	0.7071	0.3536					
Phase Sensitivity	1.6974	1.2317	1.2546	1.7263	0.6536		
Uniform Time Scaling Sensitivity							
Warping Sensitivity							
Non-positive Value Handling	1.0000	1.0000	3.0000				
Non-negativity	1.0000						
Triangle Inequality	0.0000						
Relative Sensitivity Ranges	1.2743	1.1001	1.1718	1.1718	0.7244	0.7797	0.7779

Table S8. Raw controlled testing results for the Dissimilarity Index Combining Temporal Correlation and Raw Value Behaviour. For details on how testing was performed and how relative sensitivity ranges were calculated, see section S4. Uniform time scaling sensitivity and warping sensitivity could not be tested for this distance measure, as it cannot measure unequal-length time series.

Controlled Test Results: Cort

Test	Res.1	Res.2	Res.3	Res.4	Res.5	Res.6	Res.7
Reflexivity	0.000						
Symmetry	2.566	2.566					
Translation Sensitivity	0.075	0.151	0.226	0.302	0.377		
Amplitude Sensitivity	0.174	0.369	0.582	0.810	1.048		
Duration Sensitivity	0.538	0.761	0.932	1.076	1.203		
Frequency Sensitivity	0.391	0.678	0.932	1.161	1.336		
White Noise Sensitivity	0.086	0.178	0.281	0.398	0.527		
Biased Noise Sensitivity	0.121	0.251	0.396	0.558	0.736		
Outlier Sensitivity	0.252	0.551	0.897	1.277	1.681		
Antiparallelism Bias	2.491	0.337					
Phase Sensitivity	14.955	6.919	6.215	15.084	1.561		
Uniform Time Scaling Sensitivity							
Warping Sensitivity							
Non-positive Value Handling	0.959	0.959	1.793				
Non-negativity	1.000						
Triangle Inequality	0.000						
Relative Sensitivity Ranges	0.400	1.160	0.883	1.255	0.587	0.816	1.898

Table S9. Raw controlled testing results for the Czekanowski Distance. For details on how testing was performed and how relative sensitivity ranges were calculated, see section S4. Uniform time scaling sensitivity and warping sensitivity could not be tested for this distance measure, as it cannot measure unequal-length time series.

Controlled Test Results: Czek

Test	Res.1	Res.2	Res.3	Res.4	Res.5	Res.6	Res.7
Reflexivity	0.0000						
Symmetry	0.4103	0.4103					
Translation Sensitivity	0.0400	0.0769	0.1111	0.1429	0.1724		
Amplitude Sensitivity	0.0345	0.0667	0.0968	0.1250	0.1515		
Duration Sensitivity	0.0400	0.0769	0.1111	0.1429	0.1724		
Frequency Sensitivity	0.0370	0.0714	0.1034	0.1333	0.1613		
White Noise Sensitivity	0.0156	0.0312	0.0469	0.0625	0.0781		
Biased Noise Sensitivity	0.0154	0.0303	0.0448	0.0588	0.0725		
Outlier Sensitivity	0.0204	0.0400	0.0588	0.0769	0.0943		
Antiparallelism Bias	0.1000	0.0833					
Phase Sensitivity	0.5000	0.3214	0.3214	0.5000	0.2143		
Uniform Time Scaling Sensitivity							
Warping Sensitivity							
Non-positive Value Handling	0.0769	0.0769	0.1200				
Non-negativity	0.0000						
Triangle Inequality	0.0000						
Relative Sensitivity Ranges	1.3248	1.1710	1.3248	1.2432	0.6253	0.5711	0.7397

Table S10. Raw controlled testing results for the Dice Dissimilarity. For details on how testing was performed and how relative sensitivity ranges were calculated, see section S4. Uniform time scaling sensitivity and warping sensitivity could not be tested for this distance measure, as it cannot measure unequal-length time series.

Controlled Test Results: Dice

Test	Res.1	Res.2	Res.3	Res.4	Res.5	Res.6	Res.7
Reflexivity	0.0000						
Symmetry	0.3077	0.3077					
Translation Sensitivity	0.0029	0.0108	0.0224	0.0370	0.0538		
Amplitude Sensitivity	0.0103	0.0370	0.0744	0.1176	0.1634		
Duration Sensitivity	0.0286	0.0526	0.0732	0.0909	0.1064		
Frequency Sensitivity	0.0303	0.0556	0.0769	0.0952	0.1111		
White Noise Sensitivity	0.0005	0.0018	0.0041	0.0072	0.0113		
Biased Noise Sensitivity	0.0009	0.0035	0.0075	0.0130	0.0196		
Outlier Sensitivity	0.0069	0.0256	0.0533	0.0870	0.1244		
Antiparallelism Bias	0.0952	0.0606					
Phase Sensitivity	0.4182	0.2545	0.2545	0.4545	0.1091		
Uniform Time Scaling Sensitivity							
Warping Sensitivity							
Non-positive Value Handling	0.0909	0.0909	0.2000				
Non-negativity	1.0000						
Triangle Inequality	0.0000						
Relative Sensitivity Ranges	0.6987	2.1029	1.0688	1.1100	0.1483	0.2575	1.6138

Table S11. Raw controlled testing results for the Divergence Squared Distance. For details on how testing was performed and how relative sensitivity ranges were calculated, see section S4. Uniform time scaling sensitivity and warping sensitivity could not be tested for this distance measure, as it cannot measure unequal-length time series.

Controlled Test Results: Diverge

Test	Res.1	Res.2	Res.3	Res.4	Res.5	Res.6	Res.7
Reflexivity	0.000						
Symmetry	5.089	5.089					
Translation Sensitivity	0.039	0.141	0.292	0.478	0.689		
Amplitude Sensitivity	0.049	0.160	0.298	0.444	0.592		
Duration Sensitivity	0.222	0.444	0.667	0.889	1.111		
Frequency Sensitivity	0.222	0.444	0.667	0.889	1.111		
White Noise Sensitivity	0.008	0.032	0.071	0.128	0.202		
Biased Noise Sensitivity	0.016	0.056	0.114	0.184	0.262		
Outlier Sensitivity	0.025	0.080	0.149	0.222	0.296		
Antiparallelism Bias	1.000	0.250					
Phase Sensitivity	5.763	3.034	3.148	5.960	0.854		
Uniform Time Scaling Sensitivity							
Warping Sensitivity							
Non-positive Value Handling	2.000	2.000	18.000				
Non-negativity	1.000						
Triangle Inequality	0.000						
Relative Sensitivity Ranges	1.237	1.031	1.690	1.690	0.368	0.469	0.515

Table S12. Raw controlled testing results for the Dynamic Time Warping Distance. For details on how testing was performed and how relative sensitivity ranges were calculated, see section S4.

Controlled Test Results: DTW

Test	Res.1	Res.2	Res.3	Res.4	Res.5	Res.6	Res.7
Reflexivity	0.000						
Symmetry	13.800	13.800					
Translation Sensitivity	1.900	3.800	5.700	7.600	9.500		
Amplitude Sensitivity	2.000	4.000	5.000	6.000	7.000		
Duration Sensitivity	0.000	0.000	0.000	0.000	0.000		
Frequency Sensitivity	1.000	2.000	3.000	4.000	5.000		
White Noise Sensitivity	2.000	4.000	6.000	8.000	9.750		
Biased Noise Sensitivity	2.000	4.000	5.750	6.000	7.000		
Outlier Sensitivity	2.000	4.000	5.000	6.000	7.000		
Antiparallelism Bias	2.000	3.000					
Phase Sensitivity	8.000	8.000	15.000	22.000	18.000		
Uniform Time Scaling Sensitivity	6.490	6.740	7.000	7.870	6.000		
Warping Sensitivity	0.000	0.000	0.000	0.000	0.000		
Non-positive Value Handling	3.000	3.000	4.000				
Non-negativity	1.000						
Triangle Inequality	0.000						
Relative Sensitivity Ranges	1.328	0.873	0.000	0.699	1.354	0.873	0.873

Table S13. Raw controlled testing results for the Edit Distance on Real Sequences. For details on how testing was performed and how relative sensitivity ranges were calculated, see section S4.

Controlled Test Results: EDR

Test	Res.1	Res.2	Res.3	Res.4	Res.5	Res.6	Res.7
Reflexivity	0.000						
Symmetry	10.000	10.000					
Translation Sensitivity	10.000	10.000	10.000	10.000	10.000		
Amplitude Sensitivity	2.000	2.000	2.000	2.000	2.000		
Duration Sensitivity	1.000	2.000	3.000	4.000	5.000		
Frequency Sensitivity	1.000	2.000	3.000	4.000	5.000		
White Noise Sensitivity	8.000	8.000	8.000	8.000	8.000		
Biased Noise Sensitivity	4.000	4.000	4.000	4.000	4.000		
Outlier Sensitivity	1.000	1.000	1.000	1.000	1.000		
Antiparallelism Bias	2.000	2.000					
Phase Sensitivity	2.000	4.000	6.000	7.000	6.000		
Uniform Time Scaling Sensitivity	8.000	9.000	12.000	13.000	9.000		
Warping Sensitivity	1.000	2.000	3.000	4.000	5.000		
Non-positive Value Handling	1.000	1.000	1.000				
Non-negativity	1.000						
Triangle Inequality	0.000						
Relative Sensitivity Ranges	0.000	0.000	1.000	1.000	0.000	0.000	0.000

Table S14. Raw controlled testing results for the Edit Distance with Real Penalty. For details on how testing was performed and how relative sensitivity ranges were calculated, see section S4.

Controlled Test Results: ERP

Test	Res.1	Res.2	Res.3	Res.4	Res.5	Res.6	Res.7
Reflexivity	0.000						
Symmetry	11.200	11.200					
Translation Sensitivity	1.000	2.000	3.000	4.000	5.000		
Amplitude Sensitivity	1.000	2.000	3.000	4.000	5.000		
Duration Sensitivity	1.000	2.000	3.000	4.000	5.000		
Frequency Sensitivity	1.000	2.000	3.000	4.000	5.000		
White Noise Sensitivity	1.000	2.000	3.000	4.000	5.000		
Biased Noise Sensitivity	1.000	2.000	3.000	4.000	5.000		
Outlier Sensitivity	1.000	2.000	3.000	4.000	5.000		
Antiparallelism Bias	2.000	2.000					
Phase Sensitivity	10.000	12.000	12.000	14.000	12.000		
Uniform Time Scaling Sensitivity	6.170	12.860	18.625	25.340	31.000		
Warping Sensitivity	1.000	2.000	3.000	4.000	5.000		
Non-positive Value Handling	2.000	2.000	3.000				
Non-negativity	1.000						
Triangle Inequality	1.000						
Relative Sensitivity Ranges	1.000	1.000	1.000	1.000	1.000	1.000	1.000

Table S15. Raw controlled testing results for the Euclidean Distance. For details on how testing was performed and how relative sensitivity ranges were calculated, see section S4. Uniform time scaling sensitivity and warping sensitivity could not be tested for this distance measure, as it cannot measure unequal-length time series.

Controlled Test Results: Euclidean

Test	Res.1	Res.2	Res.3	Res.4	Res.5	Res.6	Res.7
Reflexivity	0.000						
Symmetry	4.541	4.541					
Translation Sensitivity	0.316	0.632	0.949	1.265	1.581		
Amplitude Sensitivity	0.707	1.414	2.121	2.828	3.536		
Duration Sensitivity	1.000	1.414	1.732	2.000	2.236		
Frequency Sensitivity	1.000	1.414	1.732	2.000	2.236		
White Noise Sensitivity	0.354	0.707	1.061	1.414	1.768		
Biased Noise Sensitivity	0.500	1.000	1.500	2.000	2.500		
Outlier Sensitivity	1.000	2.000	3.000	4.000	5.000		
Antiparallelism Bias	1.414	1.414					
Phase Sensitivity	9.592	7.483	7.483	10.000	4.899		
Uniform Time Scaling Sensitivity							
Warping Sensitivity							
Non-positive Value Handling	2.000	2.000	3.000				
Non-negativity	1.000						
Triangle Inequality	1.000						
Relative Sensitivity Ranges	0.633	1.416	0.619	0.619	0.708	1.001	2.003

Table S16. Raw controlled testing results for the Fourier Coefficient-Based Distance. For details on how testing was performed and how relative sensitivity ranges were calculated, see section S4. Uniform time scaling sensitivity and warping sensitivity could not be tested for this distance measure, as it cannot measure unequal-length time series.

Controlled Test Results: Fourier

Test	Res.1	Res.2	Res.3	Res.4	Res.5	Res.6	Res.7
Reflexivity	0.000						
Symmetry	11.381	11.381					
Translation Sensitivity	1.000	2.000	3.000	4.000	5.000		
Amplitude Sensitivity	1.732	3.464	5.196	6.928	8.660		
Duration Sensitivity	2.449	3.464	4.472	5.292	6.164		
Frequency Sensitivity	2.646	4.000	5.196	6.325	7.416		
White Noise Sensitivity	1.061	2.121	3.182	4.243	5.303		
Biased Noise Sensitivity	1.500	3.000	4.500	6.000	7.500		
Outlier Sensitivity	2.449	4.899	7.348	9.798	12.247		
Antiparallelism Bias	3.464	3.464					
Phase Sensitivity	21.633	16.733	16.971	22.361	11.314		
Uniform Time Scaling Sensitivity							
Warping Sensitivity							
Non-positive Value Handling	4.899	4.899	7.348				
Non-negativity	1.000						
Triangle Inequality	1.000						
Relative Sensitivity Ranges	0.710	1.229	0.659	0.846	0.753	1.065	1.738

Table S17. Raw controlled testing results for the Gower Distance. For details on how testing was performed and how relative sensitivity ranges were calculated, see section S4. Uniform time scaling sensitivity and warping sensitivity could not be tested for this distance measure, as it cannot measure unequal-length time series.

Controlled Test Results: Gower

Test	Res.1	Res.2	Res.3	Res.4	Res.5	Res.6	Res.7
Reflexivity	0.0000						
Symmetry	1.2800	1.2800					
Translation Sensitivity	0.1000	0.2000	0.3000	0.4000	0.5000		
Amplitude Sensitivity	0.1000	0.2000	0.3000	0.4000	0.5000		
Duration Sensitivity	0.1000	0.2000	0.3000	0.4000	0.5000		
Frequency Sensitivity	0.0833	0.1667	0.2500	0.3333	0.4167		
White Noise Sensitivity	0.1000	0.2000	0.3000	0.4000	0.5000		
Biased Noise Sensitivity	0.1000	0.2000	0.3000	0.4000	0.5000		
Outlier Sensitivity	0.1000	0.2000	0.3000	0.4000	0.5000		
Antiparallelism Bias	0.2000	0.2000					
Phase Sensitivity	2.8000	1.8000	1.8000	2.8000	1.2000		
Uniform Time Scaling Sensitivity							
Warping Sensitivity							
Non-positive Value Handling	0.2000	0.2000	0.3000				
Non-negativity	1.0000						
Triangle Inequality	1.0000						
Relative Sensitivity Ranges	1.0244	1.0244	1.0244	0.8537	1.0244	1.0244	1.0244

Table S18. Raw controlled testing results for the Chebyshev Distance. For details on how testing was performed and how relative sensitivity ranges were calculated, see section S4. Uniform time scaling sensitivity and warping sensitivity could not be tested for this distance measure, as it cannot measure unequal-length time series.

Controlled Test Results: Chebyshev

Test	Res.1	Res.2	Res.3	Res.4	Res.5	Res.6	Res.7
Reflexivity	0.0000						
Symmetry	3.0000	3.0000					
Translation Sensitivity	0.1000	0.2000	0.3000	0.4000	0.5000		
Amplitude Sensitivity	0.5000	1.0000	1.5000	2.0000	2.5000		
Duration Sensitivity	1.0000	1.0000	1.0000	1.0000	1.0000		
Frequency Sensitivity	1.0000	1.0000	1.0000	1.0000	1.0000		
White Noise Sensitivity	0.1250	0.2500	0.3750	0.5000	0.6250		
Biased Noise Sensitivity	0.2500	0.5000	0.7500	1.0000	1.2500		
Outlier Sensitivity	1.0000	2.0000	3.0000	4.0000	5.0000		
Antiparallelism Bias	1.0000	1.0000					
Phase Sensitivity	5.0000	5.0000	5.0000	5.0000	2.0000		
Uniform Time Scaling Sensitivity							
Warping Sensitivity							
Non-positive Value Handling	2.0000	2.0000	3.0000				
Non-negativity	1.0000						
Triangle Inequality	1.0000						
Relative Sensitivity Ranges	0.2532	1.2658	0.0000	0.0000	0.3165	0.6329	2.5316

Table S19. Raw controlled testing results for the Integrated Periodogram-Based Dissimilarity. For details on how testing was performed and how relative sensitivity ranges were calculated, see section S4. Uniform time scaling sensitivity and warping sensitivity could not be tested for this distance measure, as it cannot measure unequal-length time series.

Controlled Test Results: IntPer

Test	Res.1	Res.2	Res.3	Res.4	Res.5	Res.6	Res.7
Reflexivity	0.0000						
Symmetry	0.8095	0.8095					
Translation Sensitivity	0.0000	0.0000	0.0000	0.0000	0.0000		
Amplitude Sensitivity	0.0266	0.0676	0.1029	0.1305	0.1520		
Duration Sensitivity	0.5316	0.6016	0.6298	0.6331	0.7826		
Frequency Sensitivity	0.9671	1.4235	1.5994	1.8517	1.9900		
White Noise Sensitivity	0.1465	0.3306	0.5389	0.7586	0.9791		
Biased Noise Sensitivity	0.1315	0.2976	0.4847	0.6810	0.8772		
Outlier Sensitivity	0.1486	0.2231	0.2909	0.3391	0.3739		
Antiparallelism Bias	0.0000	0.0000					
Phase Sensitivity	0.2718	0.5069	0.6876	0.8120	0.5408		
Uniform Time Scaling Sensitivity							
Warping Sensitivity							
Non-positive Value Handling	1.1307	1.1307	1.2898				
Non-negativity	1.0000						
Triangle Inequality	1.0000						
Relative Sensitivity Ranges	0.0000	0.2742	0.5485	2.2354	1.8197	1.6298	0.4924

Table S20. Raw controlled testing results for the Jaccard Distance. For details on how testing was performed and how relative sensitivity ranges were calculated, see section S4. Uniform time scaling sensitivity and warping sensitivity could not be tested for this distance measure, as it cannot measure unequal-length time series.

Controlled Test Results: Jaccard

Test	Res.1	Res.2	Res.3	Res.4	Res.5	Res.6	Res.7
Reflexivity	0.0000						
Symmetry	0.4706	0.4706					
Translation Sensitivity	0.0058	0.0213	0.0439	0.0714	0.1020		
Amplitude Sensitivity	0.0204	0.0714	0.1385	0.2105	0.2809		
Duration Sensitivity	0.0556	0.1000	0.1364	0.1667	0.1923		
Frequency Sensitivity	0.0588	0.1053	0.1429	0.1739	0.2000		
White Noise Sensitivity	0.0009	0.0036	0.0082	0.0144	0.0223		
Biased Noise Sensitivity	0.0018	0.0069	0.0150	0.0256	0.0385		
Outlier Sensitivity	0.0137	0.0500	0.1011	0.1600	0.2212		
Antiparallelism Bias	0.1739	0.1143					
Phase Sensitivity	0.5897	0.4058	0.4058	0.6250	0.1967		
Uniform Time Scaling Sensitivity							
Warping Sensitivity							
Non-positive Value Handling	0.1667	0.1667	0.3333				
Non-negativity	1.0000						
Triangle Inequality	0.0000						
Relative Sensitivity Ranges	0.7484	2.0254	1.0633	1.0977	0.1660	0.2856	1.6137

Table S21. Raw controlled testing results for the Jeffreys Divergence. For details on how testing was performed and how relative sensitivity ranges were calculated, see section S4. Uniform time scaling sensitivity and warping sensitivity could not be tested for this distance measure, as it cannot measure unequal-length time series.

Controlled Test Results: Jeffreys

Test	Res.1	Res.2	Res.3	Res.4	Res.5	Res.6	Res.7
Reflexivity	0.000						
Symmetry	14.372	14.372					
Translation Sensitivity	0.086	0.330	0.714	1.223	1.845		
Amplitude Sensitivity	0.223	0.811	1.679	2.773	4.055		
Duration Sensitivity	0.693	1.386	2.079	2.773	3.466		
Frequency Sensitivity	0.693	1.386	2.079	2.773	3.466		
White Noise Sensitivity	0.041	0.163	0.368	0.658	1.034		
Biased Noise Sensitivity	0.079	0.303	0.654	1.119	1.687		
Outlier Sensitivity	0.223	0.811	1.679	2.773	4.055		
Antiparallelism Bias	2.197	1.022					
Phase Sensitivity	37.437	20.901	21.476	39.634	6.438		
Uniform Time Scaling Sensitivity							
Warping Sensitivity							
Non-positive Value Handling	29.017	23.026					
Non-negativity	1.000						
Triangle Inequality	0.000						
Relative Sensitivity Ranges	0.701	1.527	1.105	1.105	0.396	0.641	1.527

Table S22. Raw controlled testing results for the Jaccard Difference. For details on how testing was performed and how relative sensitivity ranges were calculated, see section S4. Uniform time scaling sensitivity and warping sensitivity could not be tested for this distance measure, as it cannot measure unequal-length time series.

Controlled Test Results: Jensen

Test	Res.1	Res.2	Res.3	Res.4	Res.5	Res.6	Res.7
Reflexivity	0.0000						
Symmetry	1.6459	1.6459					
Translation Sensitivity	0.0107	0.0412	0.0890	0.1522	0.2292		
Amplitude Sensitivity	0.0278	0.1007	0.2072	0.3398	0.4934		
Duration Sensitivity	0.0849	0.1699	0.2548	0.3398	0.4247		
Frequency Sensitivity	0.0849	0.1699	0.2548	0.3398	0.4247		
White Noise Sensitivity	0.0051	0.0204	0.0460	0.0821	0.1288		
Biased Noise Sensitivity	0.0099	0.0378	0.0815	0.1392	0.2093		
Outlier Sensitivity	0.0278	0.1007	0.2072	0.3398	0.4934		
Antiparallelism Bias	0.2616	0.1263					
Phase Sensitivity	4.2886	2.4001	2.4658	4.5413	0.7938		
Uniform Time Scaling Sensitivity							
Warping Sensitivity							
Non-positive Value Handling	0.6931	0.6931					
Non-negativity	1.0000						
Triangle Inequality	0.0000						
Relative Sensitivity Ranges	0.7105	1.5142	1.1052	1.1052	0.4024	0.6485	1.5142

Table S23. Raw controlled testing results for the Kulczynski Distance. For details on how testing was performed and how relative sensitivity ranges were calculated, see section S4. Uniform time scaling sensitivity and warping sensitivity could not be tested for this distance measure, as it cannot measure unequal-length time series.

Controlled Test Results: Kulcz

Test	Res.1	Res.2	Res.3	Res.4	Res.5	Res.6	Res.7
Reflexivity	0.0000						
Symmetry	1.3913	1.3913					
Translation Sensitivity	0.0833	0.1667	0.2500	0.3333	0.4167		
Amplitude Sensitivity	0.0714	0.1429	0.2143	0.2857	0.3571		
Duration Sensitivity	0.0833	0.1667	0.2500	0.3333	0.4167		
Frequency Sensitivity	0.0769	0.1538	0.2308	0.3077	0.3846		
White Noise Sensitivity	0.0317	0.0645	0.0984	0.1333	0.1695		
Biased Noise Sensitivity	0.0312	0.0625	0.0938	0.1250	0.1562		
Outlier Sensitivity	0.0417	0.0833	0.1250	0.1667	0.2083		
Antiparallelism Bias	0.2222	0.1818					
Phase Sensitivity	2.0000	0.9474	0.9474	2.0000	0.5455		
Uniform Time Scaling Sensitivity							
Warping Sensitivity							
Non-positive Value Handling	0.1667	0.1667	0.2727				
Non-negativity	0.0000						
Triangle Inequality	0.0000						
Relative Sensitivity Ranges	1.3811	1.1838	1.3811	1.2749	0.5707	0.5179	0.6905

Table S24. Raw controlled testing results for the Kullback-Leibler Divergence. For details on how testing was performed and how relative sensitivity ranges were calculated, see section S4. Uniform time scaling sensitivity and warping sensitivity could not be tested for this distance measure, as it cannot measure unequal-length time series.

Controlled Test Results: Kullback

Test	Res.1	Res.2	Res.3	Res.4	Res.5	Res.6	Res.7
Reflexivity	0.000						
Symmetry	14.449	-0.077					
Translation Sensitivity	1.044	2.170	3.371	4.644	5.981		
Amplitude Sensitivity	1.116	2.433	3.917	5.545	7.298		
Duration Sensitivity	1.386	2.773	4.159	5.545	6.931		
Frequency Sensitivity	1.386	2.773	4.159	5.545	6.931		
White Noise Sensitivity	0.020	0.082	0.184	0.327	0.513		
Biased Noise Sensitivity	1.040	2.156	3.342	4.591	5.900		
Outlier Sensitivity	1.116	2.433	3.917	5.545	7.298		
Antiparallelism Bias	-1.099	2.554					
Phase Sensitivity	17.594	11.549	9.716	20.654	3.219		
Uniform Time Scaling Sensitivity							
Warping Sensitivity							
Non-positive Value Handling	-0.000	0.000					
Non-negativity	0.000						
Triangle Inequality	0.000						
Relative Sensitivity Ranges	1.024	1.283	1.150	1.150	0.102	1.008	1.283

Table S25. Raw controlled testing results for the Kumar-Johnson Distance. For details on how testing was performed and how relative sensitivity ranges were calculated, see section S4. Uniform time scaling sensitivity and warping sensitivity could not be tested for this distance measure, as it cannot measure unequal-length time series.

Controlled Test Results: KumarJohnson

Test	Res.1	Res.2	Res.3	Res.4	Res.5	Res.6	Res.7
Reflexivity	0.00						
Symmetry	60.02	60.02					
Translation Sensitivity	0.17	0.67	1.45	2.52	3.85		
Amplitude Sensitivity	0.45	1.70	3.68	6.36	9.78		
Duration Sensitivity	1.59	3.18	4.77	6.36	7.95		
Frequency Sensitivity	1.59	3.18	4.77	6.36	7.95		
White Noise Sensitivity	0.08	0.33	0.74	1.33	2.11		
Biased Noise Sensitivity	0.16	0.61	1.34	2.32	3.55		
Outlier Sensitivity	0.45	1.70	3.68	6.36	9.78		
Antiparallelism Bias	6.16	2.20					
Phase Sensitivity	140.11	77.92	79.91	147.54	14.17		
Uniform Time Scaling Sensitivity							
Warping Sensitivity							
Non-positive Value Handling	2,828,427,124.74	1,600,000.00					
Non-negativity	1.00						
Triangle Inequality	0.00						
Relative Sensitivity Ranges	0.64	1.61	1.10	1.10	0.35	0.59	1.61

Table S26. Raw controlled testing results for the K Divergence. For details on how testing was performed and how relative sensitivity ranges were calculated, see section S4. Uniform time scaling sensitivity and warping sensitivity could not be tested for this distance measure, as it cannot measure unequal-length time series.

Controlled Test Results: KDiv

Test	Res.1	Res.2	Res.3	Res.4	Res.5	Res.6	Res.7
Reflexivity	0.0000						
Symmetry	5.1984	-1.9065					
Translation Sensitivity	0.5106	1.0400	1.5853	2.1441	2.7147		
Amplitude Sensitivity	0.5268	1.0939	1.6881	2.3015	2.9288		
Duration Sensitivity	0.5754	1.1507	1.7261	2.3015	2.8768		
Frequency Sensitivity	0.5754	1.1507	1.7261	2.3015	2.8768		
White Noise Sensitivity	0.0051	0.0204	0.0461	0.0824	0.1298		
Biased Noise Sensitivity	0.5097	1.0367	1.5780	2.1314	2.6954		
Outlier Sensitivity	0.5268	1.0939	1.6881	2.3015	2.9288		
Antiparallelism Bias	-0.6931	1.1157					
Phase Sensitivity	4.5097	2.1852	2.6634	4.3847	0.7938		
Uniform Time Scaling Sensitivity							
Warping Sensitivity							
Non-positive Value Handling	-0.0000						
Non-negativity	0.0000						
Triangle Inequality	0.0000						
Relative Sensitivity Ranges	1.1083	1.2078	1.1572	1.1572	0.0627	1.0990	1.2078

Table S27. Raw controlled testing results for the Lorentzian Distance. For details on how testing was performed and how relative sensitivity ranges were calculated, see section S4. Uniform time scaling sensitivity and warping sensitivity could not be tested for this distance measure, as it cannot measure unequal-length time series.

Controlled Test Results: Lorentz

Test	Res.1	Res.2	Res.3	Res.4	Res.5	Res.6	Res.7
Reflexivity	0.000						
Symmetry	7.927	7.927					
Translation Sensitivity	0.953	1.823	2.624	3.365	4.055		
Amplitude Sensitivity	0.811	1.386	1.833	2.197	2.506		
Duration Sensitivity	0.693	1.386	2.079	2.773	3.466		
Frequency Sensitivity	0.693	1.386	2.079	2.773	3.466		
White Noise Sensitivity	0.942	1.785	2.548	3.244	3.884		
Biased Noise Sensitivity	0.893	1.622	2.238	2.773	3.244		
Outlier Sensitivity	0.693	1.099	1.386	1.609	1.792		
Antiparallelism Bias	1.386	1.386					
Phase Sensitivity	12.871	8.723	8.723	12.283	6.592		
Uniform Time Scaling Sensitivity							
Warping Sensitivity							
Non-positive Value Handling	1.099	1.099	1.386				
Non-negativity	1.000						
Triangle Inequality	1.000						
Relative Sensitivity Ranges	1.297	0.709	1.160	1.160	1.231	0.984	0.460

Table S28. Raw controlled testing results for the Manhattan Distance. For details on how testing was performed and how relative sensitivity ranges were calculated, see section S4. Uniform time scaling sensitivity and warping sensitivity could not be tested for this distance measure, as it cannot measure unequal-length time series.

Controlled Test Results: Manhattan

Test	Res.1	Res.2	Res.3	Res.4	Res.5	Res.6	Res.7
Reflexivity	0.000						
Symmetry	12.800	12.800					
Translation Sensitivity	1.000	2.000	3.000	4.000	5.000		
Amplitude Sensitivity	1.000	2.000	3.000	4.000	5.000		
Duration Sensitivity	1.000	2.000	3.000	4.000	5.000		
Frequency Sensitivity	1.000	2.000	3.000	4.000	5.000		
White Noise Sensitivity	1.000	2.000	3.000	4.000	5.000		
Biased Noise Sensitivity	1.000	2.000	3.000	4.000	5.000		
Outlier Sensitivity	1.000	2.000	3.000	4.000	5.000		
Antiparallelism Bias	2.000	2.000					
Phase Sensitivity	28.000	18.000	18.000	28.000	12.000		
Uniform Time Scaling Sensitivity							
Warping Sensitivity							
Non-positive Value Handling	2.000	2.000	3.000				
Non-negativity	1.000						
Triangle Inequality	1.000						
Relative Sensitivity Ranges	1.000	1.000	1.000	1.000	1.000	1.000	1.000

Table S29. Raw controlled testing results for the Normalized Compression Distance. For details on how testing was performed and how relative sensitivity ranges were calculated, see section S4.

Controlled Test Results: NCD

Test	Res.1	Res.2	Res.3	Res.4	Res.5	Res.6	Res.7
Reflexivity	0.1176						
Symmetry	0.5000	0.4583					
Translation Sensitivity	0.4737	0.4737	0.4737	0.4737	0.4737		
Amplitude Sensitivity	0.4091	0.3810	0.4348	0.3810	0.4348		
Duration Sensitivity	0.1176	0.1176	0.1765	0.1765	0.2941		
Frequency Sensitivity	0.3125	0.3125	0.3125	0.3333	0.1429		
White Noise Sensitivity	0.7941	0.7500	0.7647	0.6800	0.7647		
Biased Noise Sensitivity	0.5000	0.4815	0.5000	0.5417	0.6071		
Outlier Sensitivity	0.3182	0.3182	0.3182	0.3182	0.3182		
Antiparallelism Bias	0.2632	0.2632					
Phase Sensitivity	0.1250	0.0833	0.2083	0.1667	0.1667		
Uniform Time Scaling Sensitivity	0.6429	0.6458	0.6731	0.7091	0.6098		
Warping Sensitivity	0.1111	0.1111	0.1111	0.1579	0.1579		
Non-positive Value Handling	0.4545	0.3333	0.3333				
Non-negativity	1.0000						
Triangle Inequality	1.0000						
Relative Sensitivity Ranges	0.0000	0.4075	1.3358	1.4418	0.8638	0.9512	0.0000

Table S30. Raw controlled testing results for the Partial Autocorrelation-Based Dissimilarity. For details on how testing was performed and how relative sensitivity ranges were calculated, see section S4. Uniform time scaling sensitivity and warping sensitivity could not be tested for this distance measure, as it cannot measure unequal-length time series.

Controlled Test Results: PACF

Test	Res.1	Res.2	Res.3	Res.4	Res.5	Res.6	Res.7
Reflexivity	0.0000						
Symmetry	0.6472	0.6472					
Translation Sensitivity	0.0000	0.0000	0.0000	0.0000	0.0000		
Amplitude Sensitivity	0.1346	0.2640	0.3414	0.3904	0.4238		
Duration Sensitivity	0.8358	0.6037	0.8581	0.3727	0.7841		
Frequency Sensitivity	0.7398	0.8911	0.8791	0.7482	0.8603		
White Noise Sensitivity	0.1006	0.2073	0.3138	0.4162	0.5122		
Biased Noise Sensitivity	0.1096	0.2155	0.3131	0.4011	0.4796		
Outlier Sensitivity	0.3645	0.5340	0.5184	0.4628	0.4217		
Antiparallelism Bias	0.0000	0.0000					
Phase Sensitivity	0.5308	0.5950	0.5913	0.3674	0.2403		
Uniform Time Scaling Sensitivity							
Warping Sensitivity							
Non-positive Value Handling	0.8325	0.8325	0.9658				
Non-negativity	1.0000						
Triangle Inequality	1.0000						
Relative Sensitivity Ranges	0.0000	1.0786	1.8102	0.5642	1.5350	1.3798	0.6321

Table S31. Raw controlled testing results for the Periodogram-Based Dissimilarity. For details on how testing was performed and how relative sensitivity ranges were calculated, see section S4. Uniform time scaling sensitivity and warping sensitivity could not be tested for this distance measure, as it cannot measure unequal-length time series.

Controlled Test Results: Per

Test	Res.1	Res.2	Res.3	Res.4	Res.5	Res.6	Res.7
Reflexivity	0.0000						
Symmetry	0.8701	0.8701					
Translation Sensitivity	0.0000	0.0000	0.0000	0.0000	0.0000		
Amplitude Sensitivity	0.1000	0.2404	0.4218	0.6445	0.9086		
Duration Sensitivity	0.0623	0.1053	0.1463	0.1303	0.0771		
Frequency Sensitivity	0.0582	0.1368	0.2421	0.3819	0.4624		
White Noise Sensitivity	0.1191	0.2836	0.4936	0.7494	1.0511		
Biased Noise Sensitivity	0.1389	0.3176	0.5385	0.8029	1.1115		
Outlier Sensitivity	0.3066	0.6883	1.1539	1.7090	2.3572		
Antiparallelism Bias	0.0000	0.1666					
Phase Sensitivity	0.8618	1.3408	1.1143	1.0548	0.9195		
Uniform Time Scaling Sensitivity							
Warping Sensitivity							
Non-positive Value Handling	0.1963	0.1963	0.4163				
Non-negativity	1.0000						
Triangle Inequality	1.0000						
Relative Sensitivity Ranges	0.0000	1.0778	0.1120	0.5386	1.2421	1.2963	2.7332

Table S32. Raw controlled testing results for the Piccolo Distance. For details on how testing was performed and how relative sensitivity ranges were calculated, see section S4.

Controlled Test Results: Piccolo

Test	Res.1	Res.2	Res.3	Res.4	Res.5	Res.6	Res.7
Reflexivity	0.0000						
Symmetry	0.5655	0.5655					
Translation Sensitivity	0.0000	0.0000	0.0000	0.0000	0.0000		
Amplitude Sensitivity	0.0571	0.1382	0.2019	0.2484	0.2827		
Duration Sensitivity	0.1310	0.1667	0.1500	0.0833	0.0595		
Frequency Sensitivity	0.1182	0.5564	0.5511	0.6753	0.8182		
White Noise Sensitivity	0.0680	0.1302	0.1824	0.2246	0.2586		
Biased Noise Sensitivity	0.0721	0.1312	0.1782	0.2155	0.2459		
Outlier Sensitivity	0.0523	0.0973	0.1310	0.1555	0.1734		
Antiparallelism Bias	0.0000	0.0000					
Phase Sensitivity	0.0000	0.0570	0.2468	0.0570	0.1139		
Uniform Time Scaling Sensitivity	0.3241	0.6077	0.6239	0.6389	0.6461		
Warping Sensitivity	0.6642	0.8488	0.8799	0.4125	0.4522		
Non-positive Value Handling	0.9883	0.9883	1.1271				
Non-negativity	1.0000						
Triangle Inequality	1.0000						
Relative Sensitivity Ranges	0.0000	1.0399	0.4940	3.2275	0.8785	0.8014	0.5587

Table S33. Raw controlled testing results for the Probabilistic Symmetric Chi-Squared Distance. For details on how testing was performed and how relative sensitivity ranges were calculated, see section S4. Uniform time scaling sensitivity and warping sensitivity could not be tested for this distance measure, as it cannot measure unequal-length time series.

Controlled Test Results: ProbSymm

Test	Res.1	Res.2	Res.3	Res.4	Res.5	Res.6	Res.7
Reflexivity	0.000						
Symmetry	12.279	12.279					
Translation Sensitivity	0.086	0.329	0.710	1.212	1.822		
Amplitude Sensitivity	0.222	0.800	1.636	2.667	3.846		
Duration Sensitivity	0.667	1.333	2.000	2.667	3.333		
Frequency Sensitivity	0.667	1.333	2.000	2.667	3.333		
White Noise Sensitivity	0.041	0.163	0.368	0.655	1.028		
Biased Noise Sensitivity	0.079	0.302	0.650	1.108	1.662		
Outlier Sensitivity	0.222	0.800	1.636	2.667	3.846		
Antiparallelism Bias	2.000	1.000					
Phase Sensitivity	31.810	17.854	18.334	33.676	6.267		
Uniform Time Scaling Sensitivity							
Warping Sensitivity							
Non-positive Value Handling	4.000	4.000	18.000				
Non-negativity	0.000						
Triangle Inequality	0.000						
Relative Sensitivity Ranges	0.720	1.502	1.105	1.105	0.409	0.656	1.502

Table S34. Raw controlled testing results for the Soergel Distance. For details on how testing was performed and how relative sensitivity ranges were calculated, see section S4. Uniform time scaling sensitivity and warping sensitivity could not be tested for this distance measure, as it cannot measure unequal-length time series.

Controlled Test Results: Soergel

Test	Res.1	Res.2	Res.3	Res.4	Res.5	Res.6	Res.7
Reflexivity	0.0000						
Symmetry	0.5818	0.5818					
Translation Sensitivity	0.0769	0.1429	0.2000	0.2500	0.2941		
Amplitude Sensitivity	0.0667	0.1250	0.1765	0.2222	0.2632		
Duration Sensitivity	0.0769	0.1429	0.2000	0.2500	0.2941		
Frequency Sensitivity	0.0714	0.1333	0.1875	0.2353	0.2778		
White Noise Sensitivity	0.0308	0.0606	0.0896	0.1176	0.1449		
Biased Noise Sensitivity	0.0303	0.0588	0.0857	0.1111	0.1351		
Outlier Sensitivity	0.0400	0.0769	0.1111	0.1429	0.1724		
Antiparallelism Bias	0.1818	0.1538					
Phase Sensitivity	0.6667	0.4865	0.4865	0.6667	0.3529		
Uniform Time Scaling Sensitivity							
Warping Sensitivity							
Non-positive Value Handling	0.1429	0.1429	0.2143				
Non-negativity	1.0000						
Triangle Inequality	1.0000						
Relative Sensitivity Ranges	1.2791	1.1572	1.2791	1.2152	0.6723	0.6174	0.7798

Table S35. Raw controlled testing results for the Squared Chi-Squared Distance. For details on how testing was performed and how relative sensitivity ranges were calculated, see section S4. Uniform time scaling sensitivity and warping sensitivity could not be tested for this distance measure, as it cannot measure unequal-length time series.

Controlled Test Results: SqChi

Test	Res.1	Res.2	Res.3	Res.4	Res.5	Res.6	Res.7
Reflexivity	0.000						
Symmetry	6.139	6.139					
Translation Sensitivity	0.043	0.165	0.355	0.606	0.911		
Amplitude Sensitivity	0.111	0.400	0.818	1.333	1.923		
Duration Sensitivity	0.333	0.667	1.000	1.333	1.667		
Frequency Sensitivity	0.333	0.667	1.000	1.333	1.667		
White Noise Sensitivity	0.020	0.082	0.184	0.328	0.514		
Biased Noise Sensitivity	0.040	0.151	0.325	0.554	0.831		
Outlier Sensitivity	0.111	0.400	0.818	1.333	1.923		
Antiparallelism Bias	1.000	0.500					
Phase Sensitivity	15.905	8.927	9.167	16.838	3.133		
Uniform Time Scaling Sensitivity							
Warping Sensitivity							
Non-positive Value Handling	2.000	2.000	9.000				
Non-negativity	0.000						
Triangle Inequality	0.000						
Relative Sensitivity Ranges	0.720	1.502	1.105	1.105	0.409	0.656	1.502

Table S36. Raw controlled testing results for the Squared-Chord Distance. For details on how testing was performed and how relative sensitivity ranges were calculated, see section S4. Uniform time scaling sensitivity and warping sensitivity could not be tested for this distance measure, as it cannot measure unequal-length time series.

Controlled Test Results: SqChord

Test	Res.1	Res.2	Res.3	Res.4	Res.5	Res.6	Res.7
Reflexivity	0.0000						
Symmetry	3.4299	3.4299					
Translation Sensitivity	0.0215	0.0824	0.1781	0.3050	0.4598		
Amplitude Sensitivity	0.0557	0.2020	0.4170	0.6863	1.0000		
Duration Sensitivity	0.1716	0.3431	0.5147	0.6863	0.8579		
Frequency Sensitivity	0.1716	0.3431	0.5147	0.6863	0.8579		
White Noise Sensitivity	0.0102	0.0408	0.0921	0.1643	0.2580		
Biased Noise Sensitivity	0.0198	0.0757	0.1633	0.2791	0.4201		
Outlier Sensitivity	0.0557	0.2020	0.4170	0.6863	1.0000		
Antiparallelism Bias	0.5359	0.2540					
Phase Sensitivity	8.9453	4.9998	5.1372	9.4719	1.5984		
Uniform Time Scaling Sensitivity							
Warping Sensitivity							
Non-positive Value Handling	1.9972	2.0000					
Non-negativity	1.0000						
Triangle Inequality	0.0000						
Relative Sensitivity Ranges	0.7057	1.5204	1.1050	1.1050	0.3990	0.6446	1.5204

Table S37. Raw controlled testing results for the Squared Euclidean Distance. For details on how testing was performed and how relative sensitivity ranges were calculated, see section S4. Uniform time scaling sensitivity and warping sensitivity could not be tested for this distance measure, as it cannot measure unequal-length time series.

Controlled Test Results: SqEuclid

Test	Res.1	Res.2	Res.3	Res.4	Res.5	Res.6	Res.7
Reflexivity	0.00						
Symmetry	20.62	20.62					
Translation Sensitivity	0.10	0.40	0.90	1.60	2.50		
Amplitude Sensitivity	0.50	2.00	4.50	8.00	12.50		
Duration Sensitivity	1.00	2.00	3.00	4.00	5.00		
Frequency Sensitivity	1.00	2.00	3.00	4.00	5.00		
White Noise Sensitivity	0.12	0.50	1.12	2.00	3.12		
Biased Noise Sensitivity	0.25	1.00	2.25	4.00	6.25		
Outlier Sensitivity	1.00	4.00	9.00	16.00	25.00		
Antiparallelism Bias	2.00	2.00					
Phase Sensitivity	92.00	56.00	56.00	100.00	24.00		
Uniform Time Scaling Sensitivity							
Warping Sensitivity							
Non-positive Value Handling	4.00	4.00	9.00				
Non-negativity	1.00						
Triangle Inequality	0.00						
Relative Sensitivity Ranges	0.30	1.52	0.51	0.51	0.38	0.76	3.03

Table S38. Raw controlled testing results for the Short Time Series Distance. For details on how testing was performed and how relative sensitivity ranges were calculated, see section S4. Uniform time scaling sensitivity and warping sensitivity could not be tested for this distance measure, as it cannot measure unequal-length time series.

Controlled Test Results: STS

Test	Res.1	Res.2	Res.3	Res.4	Res.5	Res.6	Res.7
Reflexivity	0.000						
Symmetry	5.257	5.257					
Translation Sensitivity	0.000	0.000	0.000	0.000	0.000		
Amplitude Sensitivity	0.707	1.414	2.121	2.828	3.536		
Duration Sensitivity	1.414	1.414	1.414	1.414	1.414		
Frequency Sensitivity	1.414	2.000	2.449	2.828	3.000		
White Noise Sensitivity	0.685	1.369	2.054	2.739	3.423		
Biased Noise Sensitivity	0.707	1.414	2.121	2.828	3.536		
Outlier Sensitivity	1.414	2.828	4.243	5.657	7.071		
Antiparallelism Bias	1.414	1.414					
Phase Sensitivity	15.748	12.124	11.662	14.933	5.292		
Uniform Time Scaling Sensitivity							
Warping Sensitivity							
Non-positive Value Handling	2.828	2.828	4.243				
Non-negativity	1.000						
Triangle Inequality	1.000						
Relative Sensitivity Ranges	0.000	1.085	0.000	0.608	1.051	1.085	2.170

Table S39. Raw controlled testing results for the Time Alignment Measurement Distance. For details on how testing was performed and how relative sensitivity ranges were calculated, see section S4.

Controlled Test Results: TAM

Test	Res.1	Res.2	Res.3	Res.4	Res.5	Res.6	Res.7
Reflexivity	0.0000						
Symmetry	2.0000	2.0000					
Translation Sensitivity	0.0000	0.6667	0.6667	0.6667	0.0000		
Amplitude Sensitivity	0.0000	0.0000	0.6667	0.6667	0.6667		
Duration Sensitivity	0.3333	0.6667	1.0000	1.3333	1.6667		
Frequency Sensitivity	0.2727	0.5455	0.8182	1.0909	1.3636		
White Noise Sensitivity	0.0000	0.0000	0.0000	0.0000	0.3333		
Biased Noise Sensitivity	0.0000	0.0000	0.3333	0.6667	1.0000		
Outlier Sensitivity	0.0000	0.0000	0.3333	0.3333	0.3333		
Antiparallelism Bias	1.0000	0.6667					
Phase Sensitivity	0.3333	0.6667	1.0000	1.3333	1.6667		
Uniform Time Scaling Sensitivity	0.7444	0.2500	0.3571	0.4375	0.5000		
Warping Sensitivity	0.1000	0.1818	0.2500	0.3077	0.3571		
Non-positive Value Handling	0.3333	0.3333	0.3333				
Non-negativity	1.0000						
Triangle Inequality	0.0000						
Relative Sensitivity Ranges	0.8603	0.8603	1.7207	1.4078	0.4302	1.2905	0.4302

Table S40. Raw controlled testing results for the Taneja Difference. For details on how testing was performed and how relative sensitivity ranges were calculated, see section S4. Uniform time scaling sensitivity and warping sensitivity could not be tested for this distance measure, as it cannot measure unequal-length time series.

Controlled Test Results: Taneja

Test	Res.1	Res.2	Res.3	Res.4	Res.5	Res.6	Res.7
Reflexivity	0.000						
Symmetry	1.947	1.947					
Translation Sensitivity	0.011	0.041	0.089	0.153	0.232		
Amplitude Sensitivity	0.028	0.102	0.213	0.353	0.520		
Duration Sensitivity	0.088	0.177	0.265	0.353	0.442		
Frequency Sensitivity	0.088	0.177	0.265	0.353	0.442		
White Noise Sensitivity	0.005	0.020	0.046	0.082	0.130		
Biased Noise Sensitivity	0.010	0.038	0.082	0.141	0.212		
Outlier Sensitivity	0.028	0.102	0.213	0.353	0.520		
Antiparallelism Bias	0.288	0.129					
Phase Sensitivity	5.071	2.825	2.903	5.367	0.816		
Uniform Time Scaling Sensitivity							
Warping Sensitivity							
Non-positive Value Handling	6.561	12.206					
Non-negativity	1.000						
Triangle Inequality	0.000						
Relative Sensitivity Ranges	0.692	1.539	1.104	1.104	0.389	0.633	1.539

Table S41. Raw controlled testing results for the Topsoe Distance. For details on how testing was performed and how relative sensitivity ranges were calculated, see section S4. Uniform time scaling sensitivity and warping sensitivity could not be tested for this distance measure, as it cannot measure unequal-length time series.

Controlled Test Results: Topsoe

Test	Res.1	Res.2	Res.3	Res.4	Res.5	Res.6	Res.7
Reflexivity	0.0000						
Symmetry	3.2919	3.2919					
Translation Sensitivity	0.0215	0.0824	0.1779	0.3043	0.4584		
Amplitude Sensitivity	0.0557	0.2014	0.4143	0.6796	0.9868		
Duration Sensitivity	0.1699	0.3398	0.5097	0.6796	0.8495		
Frequency Sensitivity	0.1699	0.3398	0.5097	0.6796	0.8495		
White Noise Sensitivity	0.0102	0.0408	0.0920	0.1641	0.2577		
Biased Noise Sensitivity	0.0198	0.0757	0.1631	0.2784	0.4186		
Outlier Sensitivity	0.0557	0.2014	0.4143	0.6796	0.9868		
Antiparallelism Bias	0.5232	0.2527					
Phase Sensitivity	8.5772	4.8003	4.9316	9.0826	1.5876		
Uniform Time Scaling Sensitivity							
Warping Sensitivity							
Non-positive Value Handling	1.3863						
Non-negativity	1.0000						
Triangle Inequality	0.0000						
Relative Sensitivity Ranges	0.7105	1.5142	1.1052	1.1052	0.4024	0.6485	1.5142

Table S42. Raw controlled testing results for the Wave-Hedges Distance. For details on how testing was performed and how relative sensitivity ranges were calculated, see section S4. Uniform time scaling sensitivity and warping sensitivity could not be tested for this distance measure, as it cannot measure unequal-length time series.

Controlled Test Results: WaveHedges

Test	Res.1	Res.2	Res.3	Res.4	Res.5	Res.6	Res.7
Reflexivity	0.0000						
Symmetry	6.0500	6.0500					
Translation Sensitivity	0.8225	1.5152	2.1070	2.6190	3.0667		
Amplitude Sensitivity	0.4000	0.6667	0.8571	1.0000	1.1111		
Duration Sensitivity	0.5000	1.0000	1.5000	2.0000	2.5000		
Frequency Sensitivity	0.5000	1.0000	1.5000	2.0000	2.5000		
White Noise Sensitivity	0.3189	0.6231	0.9147	1.1955	1.4670		
Biased Noise Sensitivity	0.3022	0.5538	0.7677	0.9524	1.1141		
Outlier Sensitivity	0.2000	0.3333	0.4286	0.5000	0.5556		
Antiparallelism Bias	1.3333	0.8000					
Phase Sensitivity	6.6000	4.0667	4.1000	6.4333	2.4667		
Uniform Time Scaling Sensitivity							
Warping Sensitivity							
Non-positive Value Handling	1.0000	1.0000	1.5000				
Non-negativity	0.0000						
Triangle Inequality	0.0000						
Relative Sensitivity Ranges	1.6945	0.5369	1.5101	1.5101	0.8669	0.6130	0.2685

3. Plots of wading bird rankings for all distance measures

This section contains plots of wading bird dissimilarity results for all 42 distance measures we tested. Each figure shows dissimilarity results for both smoothed and unsmoothed indices of all five wading birds. Distance measures are presented in alphabetical order.

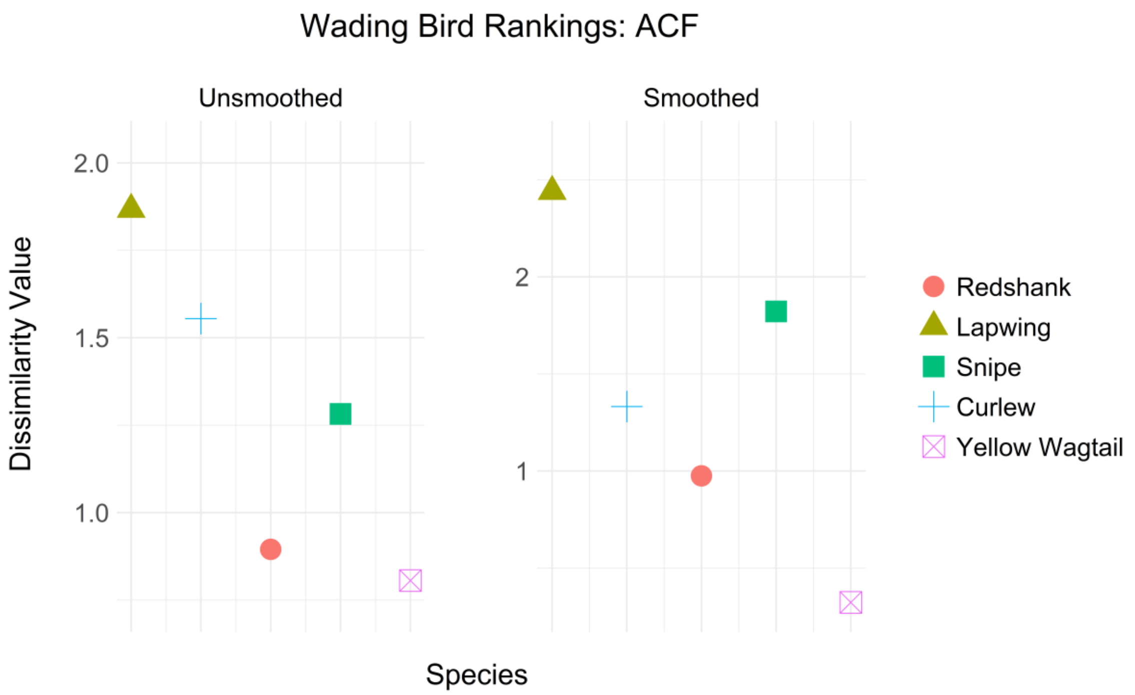

Fig. S43. Dissimilarity values for trend comparisons of five wading bird species using the Autocorrelation-Based Dissimilarity. Trends within reserves were compared with counterfactual trends from outside of reserves. Values on the left were from comparisons of the unsmoothed trends, while values on the right were calculated after applying LOESS smoothing with a span setting of 0.75. The dataset is from a study of conservation impact of wet grassland reserves on breeding birds in the UK (Jellesmark et al., 2021).

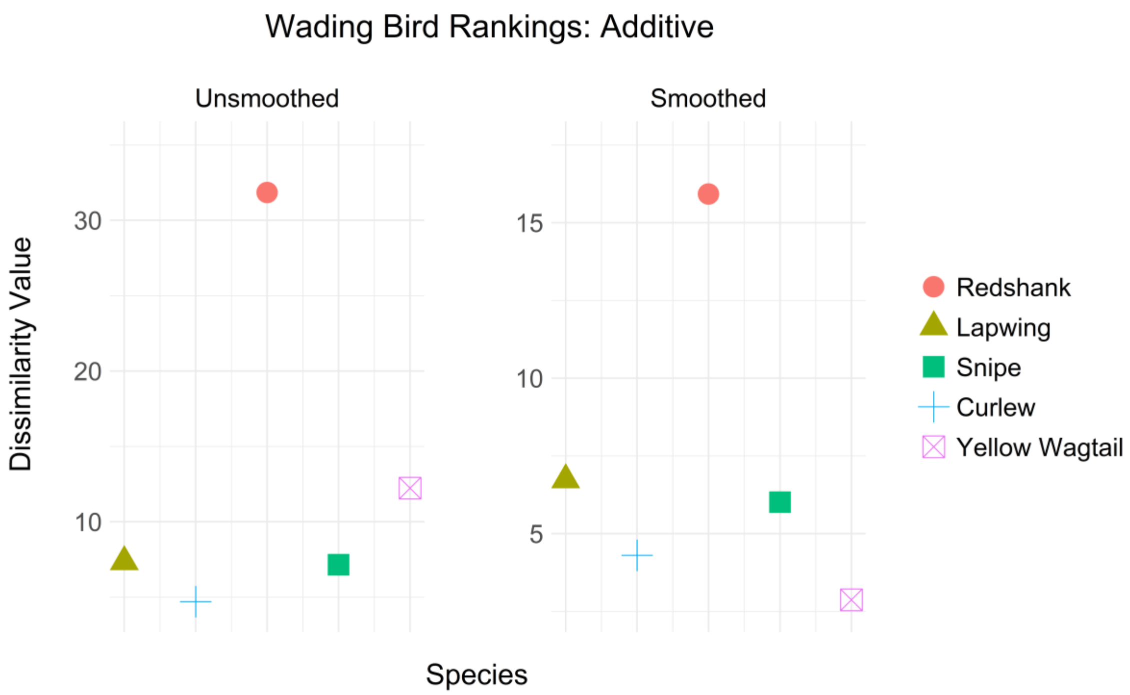

Fig. S44. Dissimilarity values for trend comparisons of five wading bird species using the Additive Symmetric Chi-Squared Distance. Trends within reserves were compared with counterfactual trends from outside of reserves. Values on the left were from comparisons of the unsmoothed trends, while values on the right were calculated after applying LOESS smoothing with a span setting of 0.75. The dataset is from a study of conservation impact of wet grassland reserves on breeding birds in the UK (Jellesmark et al., 2021).

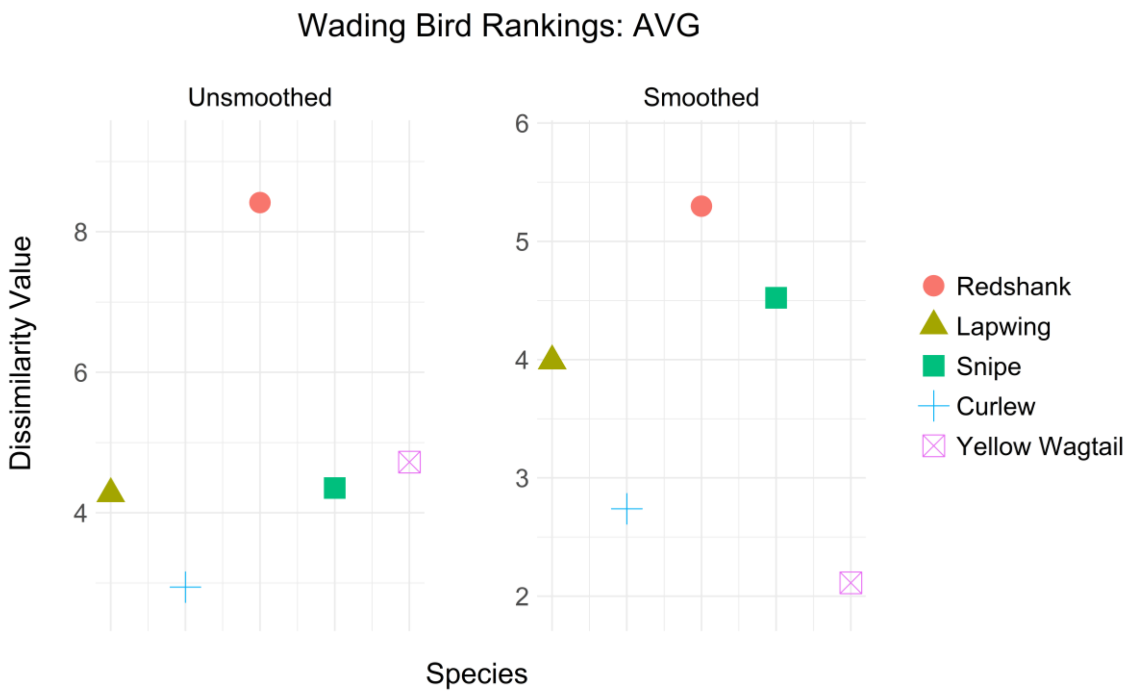

Fig. S45. Dissimilarity values for trend comparisons of five wading bird species using the Average Distance. Trends within reserves were compared with counterfactual trends from outside of reserves. Values on the left were from comparisons of the unsmoothed trends, while values on the right were calculated after applying LOESS smoothing with a span setting of 0.75. The dataset is from a study of conservation impact of wet grassland reserves on breeding birds in the UK (Jellesmark et al., 2021).

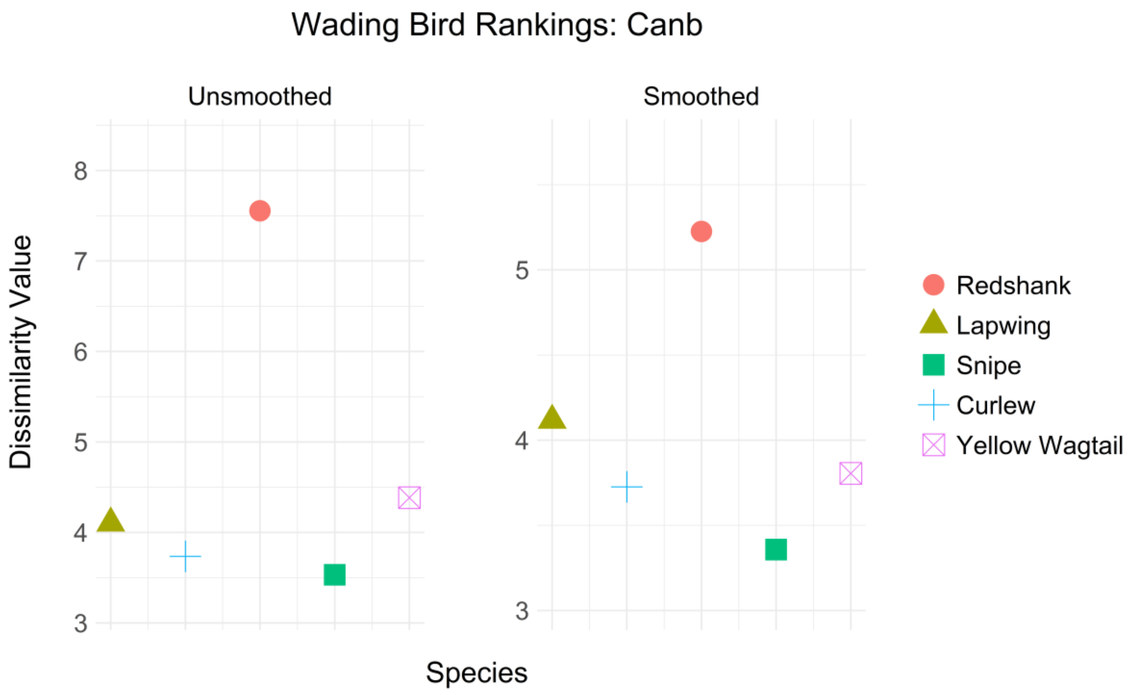

Fig. S46. Dissimilarity values for trend comparisons of five wading bird species using the Canberra Distance. Trends within reserves were compared with counterfactual trends from outside of reserves. Values on the left were from comparisons of the unsmoothed trends, while values on the right were calculated after applying LOESS smoothing with a span setting of 0.75. The dataset is from a study of conservation impact of wet grassland reserves on breeding birds in the UK (Jellesmark et al., 2021).

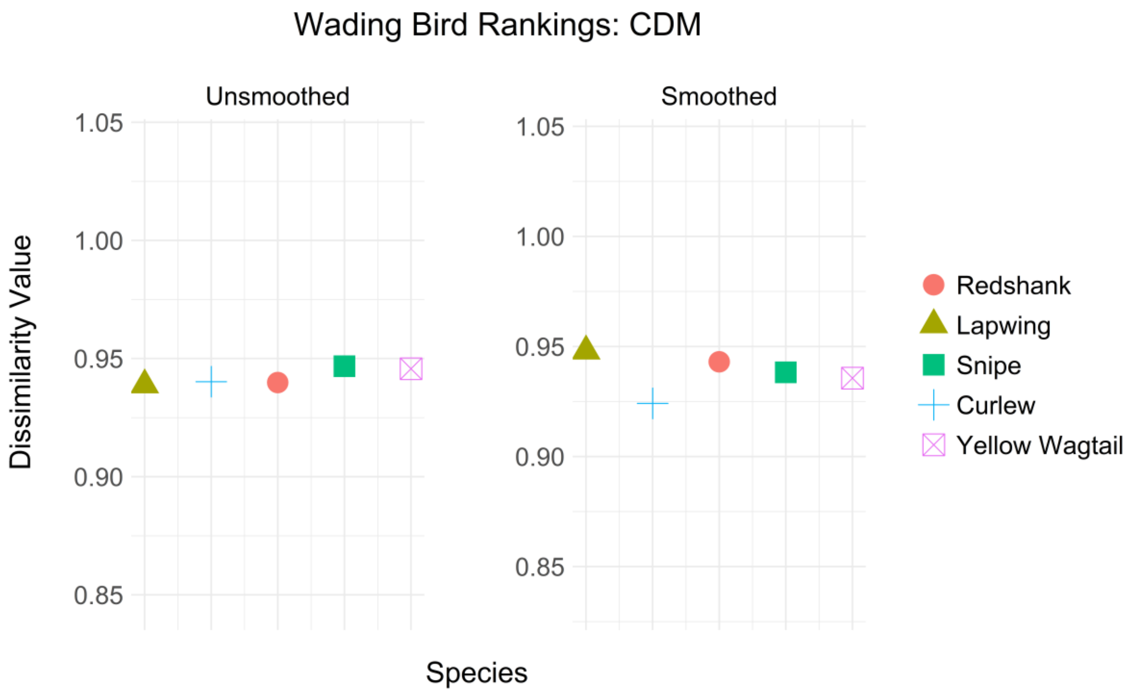

Fig. S47. Dissimilarity values for trend comparisons of five wading bird species using the Compression-Based Dissimilarity Measure. Trends within reserves were compared with counterfactual trends from outside of reserves. Values on the left were from comparisons of the unsmoothed trends, while values on the right were calculated after applying LOESS smoothing with a span setting of 0.75. The dataset is from a study of conservation impact of wet grassland reserves on breeding birds in the UK (Jellesmark et al., 2021).

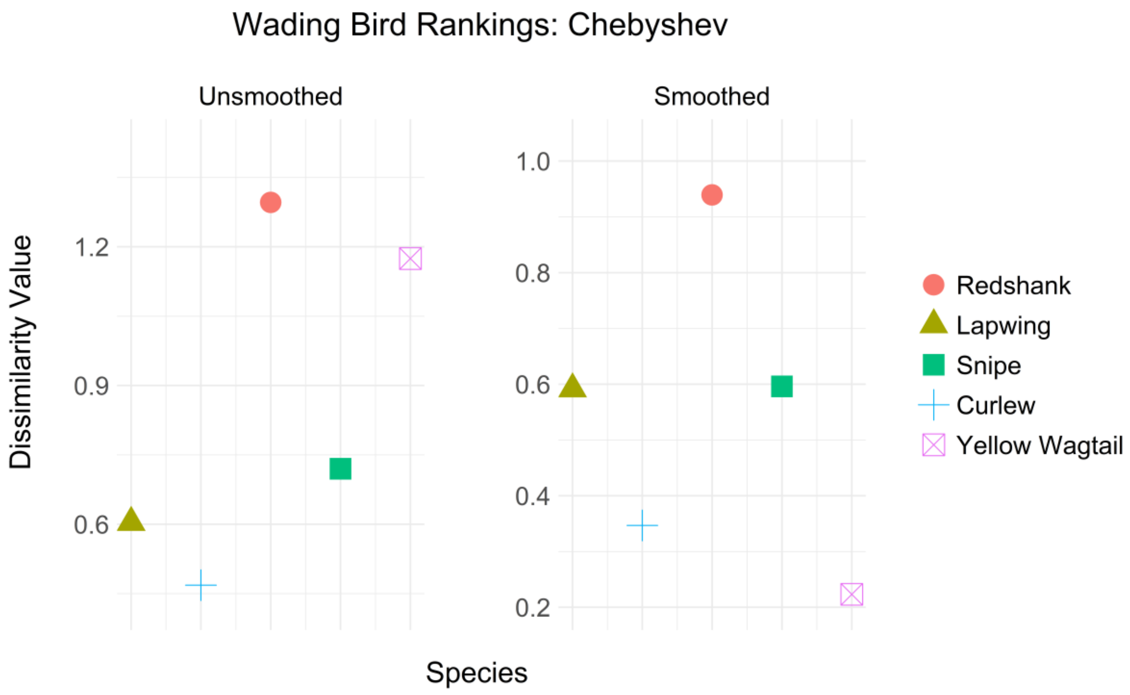

Fig. S48. Dissimilarity values for trend comparisons of five wading bird species using the Chebyshev Distance. Trends within reserves were compared with counterfactual trends from outside of reserves. Values on the left were from comparisons of the unsmoothed trends, while values on the right were calculated after applying LOESS smoothing with a span setting of 0.75. The dataset is from a study of conservation impact of wet grassland reserves on breeding birds in the UK (Jellesmark et al., 2021).

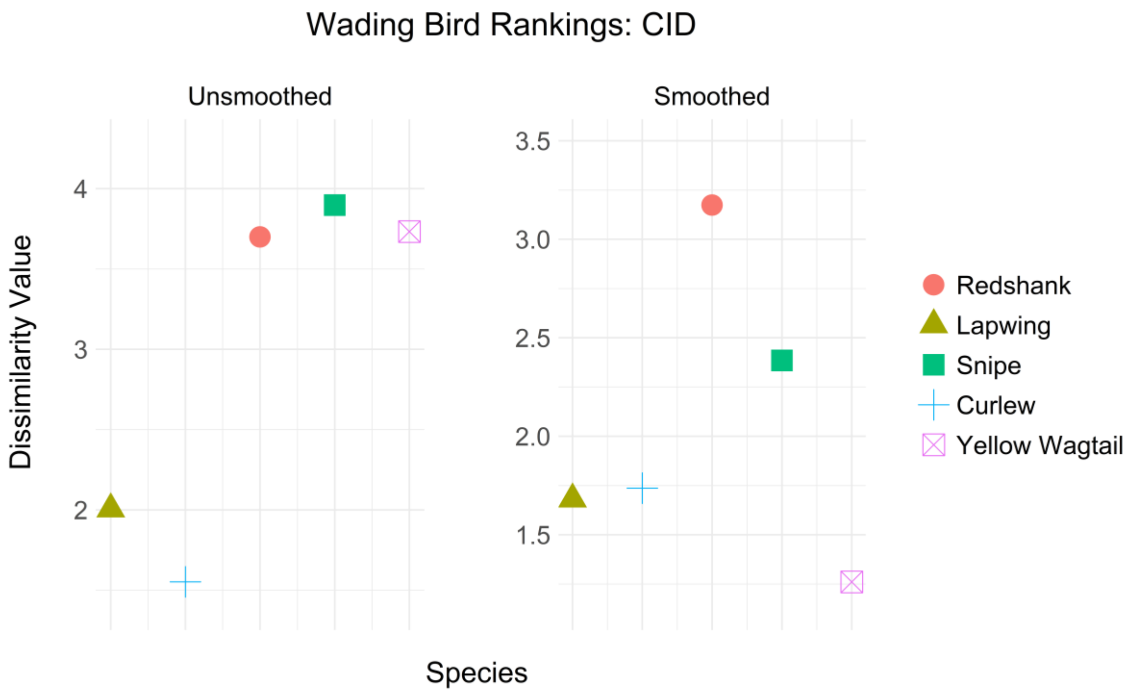

Fig. S49. Dissimilarity values for trend comparisons of five wading bird species using the Complexity-Invariant Distance. Trends within reserves were compared with counterfactual trends from outside of reserves. Values on the left were from comparisons of the unsmoothed trends, while values on the right were calculated after applying LOESS smoothing with a span setting of 0.75. The dataset is from a study of conservation impact of wet grassland reserves on breeding birds in the UK (Jellesmark et al., 2021).

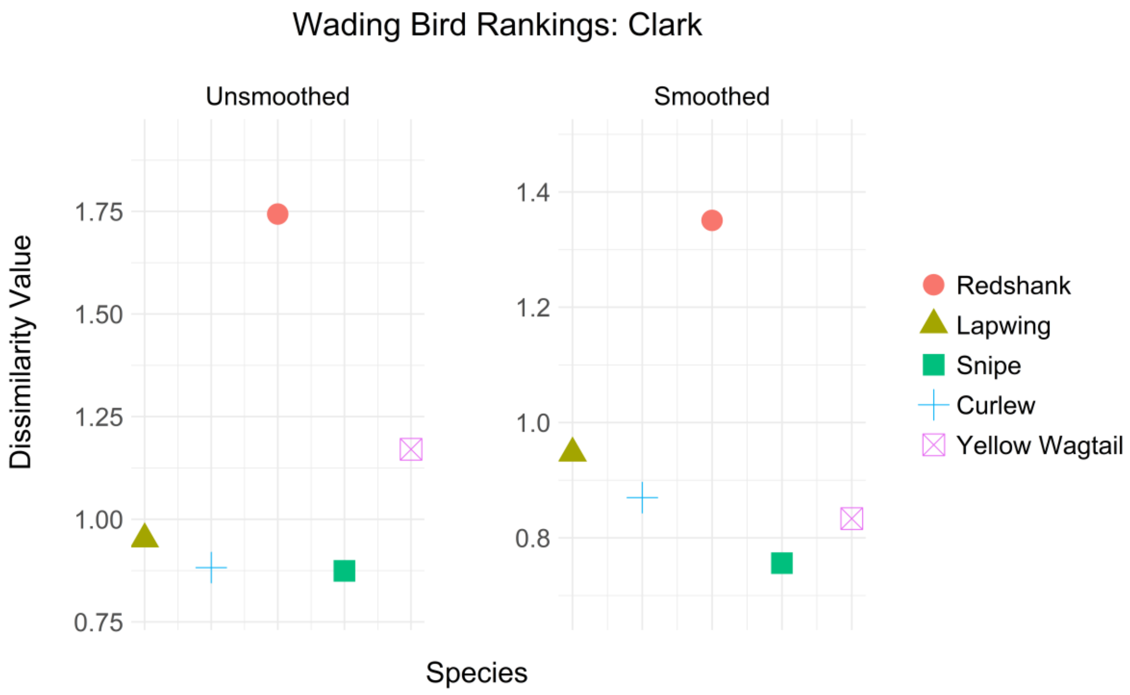

Fig. S50. Dissimilarity values for trend comparisons of five wading bird species using the Clark Squared Distance. Trends within reserves were compared with counterfactual trends from outside of reserves. Values on the left were from comparisons of the unsmoothed trends, while values on the right were calculated after applying LOESS smoothing with a span setting of 0.75. The dataset is from a study of conservation impact of wet grassland reserves on breeding birds in the UK (Jellesmark et al., 2021).

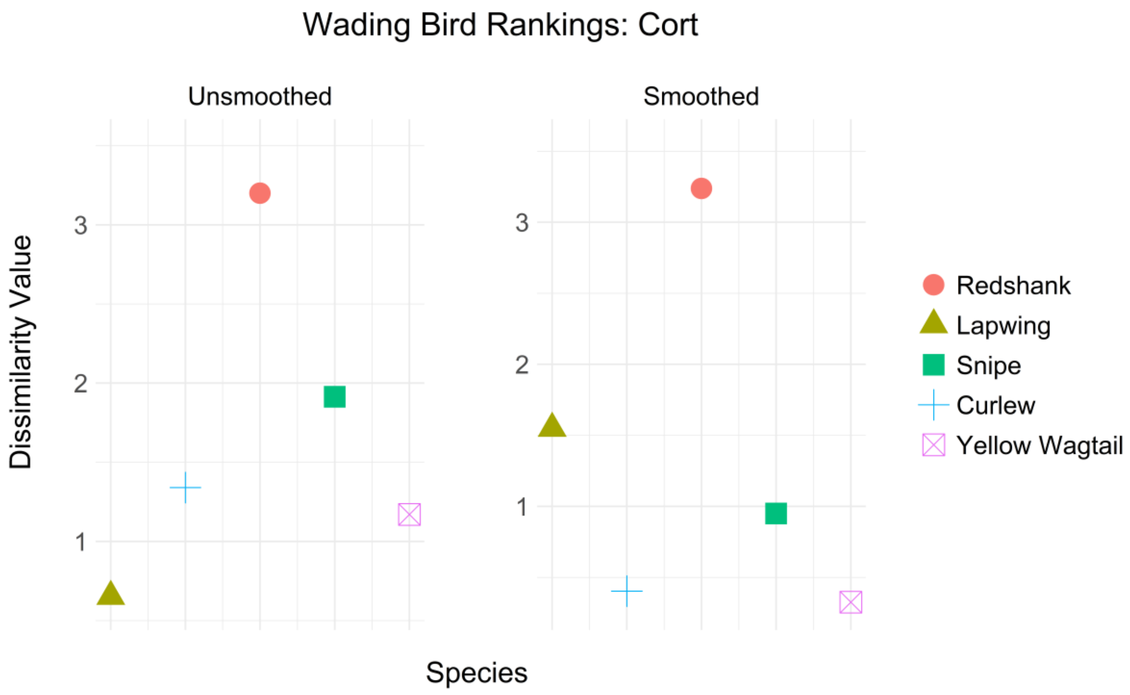

Fig. S51. Dissimilarity values for trend comparisons of five wading bird species using the Dissimilarity Index Combining Temporal Correlation and Raw Value Behaviour. Trends within reserves were compared with counterfactual trends from outside of reserves. Values on the left were from comparisons of the unsmoothed trends, while values on the right were calculated after applying LOESS smoothing with a span setting of 0.75. The dataset is from a study of conservation impact of wet grassland reserves on breeding birds in the UK (Jellesmark et al., 2021).

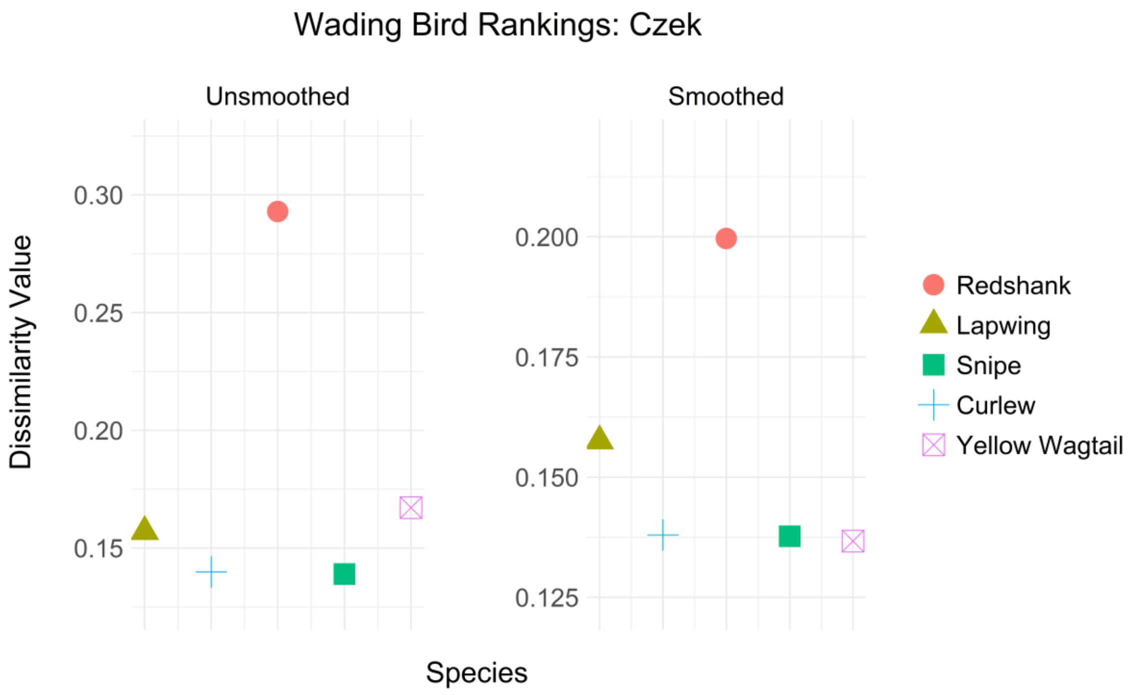

Fig. S52. Dissimilarity values for trend comparisons of five wading bird species using the Czekanowski Distance. Trends within reserves were compared with counterfactual trends from outside of reserves. Values on the left were from comparisons of the unsmoothed trends, while values on the right were calculated after applying LOESS smoothing with a span setting of 0.75. The dataset is from a study of conservation impact of wet grassland reserves on breeding birds in the UK (Jellesmark et al., 2021).

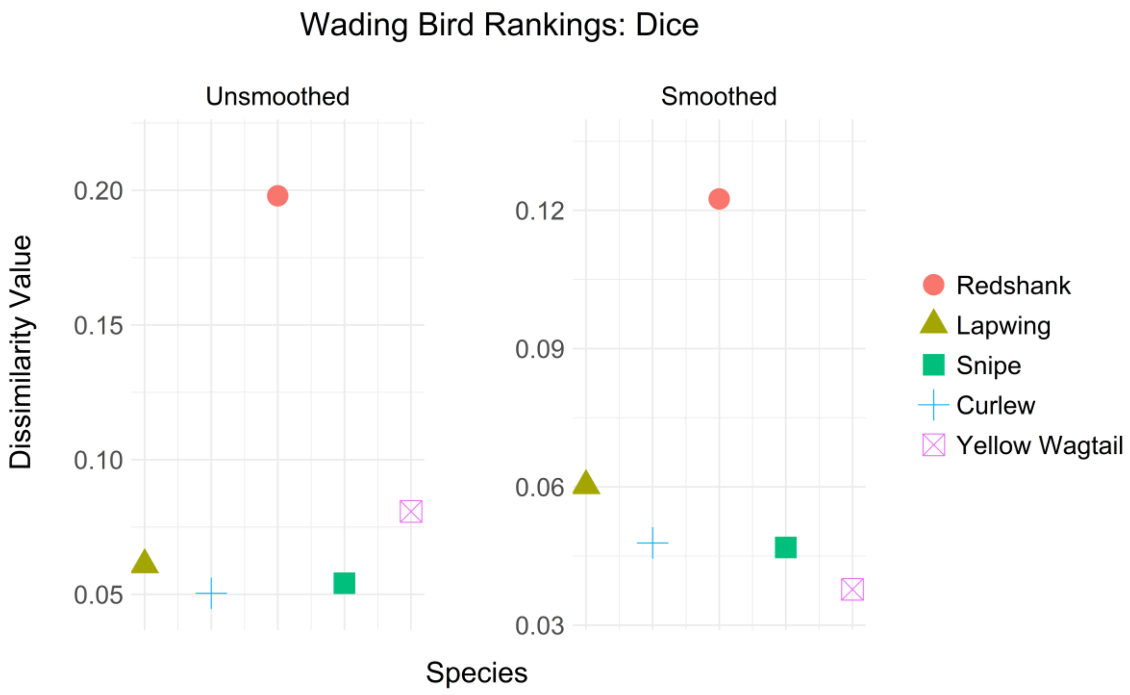

Fig. S53. Dissimilarity values for trend comparisons of five wading bird species using the Dice Dissimilarity. Trends within reserves were compared with counterfactual trends from outside of reserves. Values on the left were from comparisons of the unsmoothed trends, while values on the right were calculated after applying LOESS smoothing with a span setting of 0.75. The dataset is from a study of conservation impact of wet grassland reserves on breeding birds in the UK (Jellesmark et al., 2021).

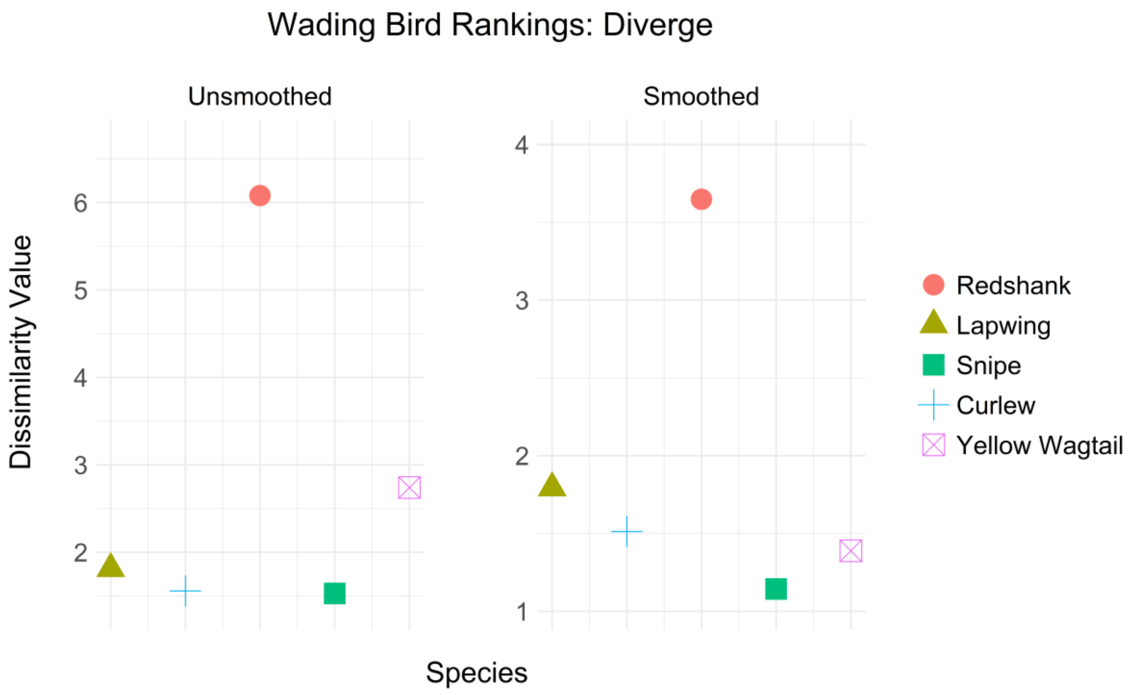

Fig. S54. Dissimilarity values for trend comparisons of five wading bird species using the Divergence Squared Distance. Trends within reserves were compared with counterfactual trends from outside of reserves. Values on the left were from comparisons of the unsmoothed trends, while values on the right were calculated after applying LOESS smoothing with a span setting of 0.75. The dataset is from a study of conservation impact of wet grassland reserves on breeding birds in the UK (Jellesmark et al., 2021).

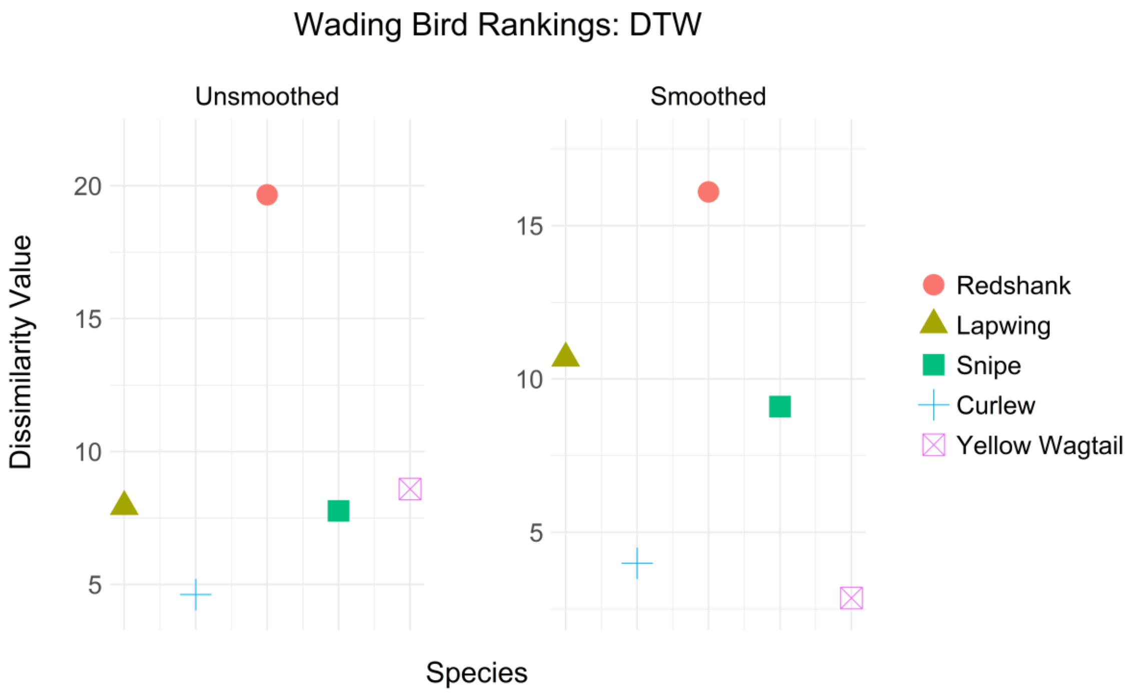

Fig. S55. Dissimilarity values for trend comparisons of five wading bird species using the Dynamic Time Warping Distance. Trends within reserves were compared with counterfactual trends from outside of reserves. Values on the left were from comparisons of the unsmoothed trends, while values on the right were calculated after applying LOESS smoothing with a span setting of 0.75. The dataset is from a study of conservation impact of wet grassland reserves on breeding birds in the UK (Jellesmark et al., 2021).

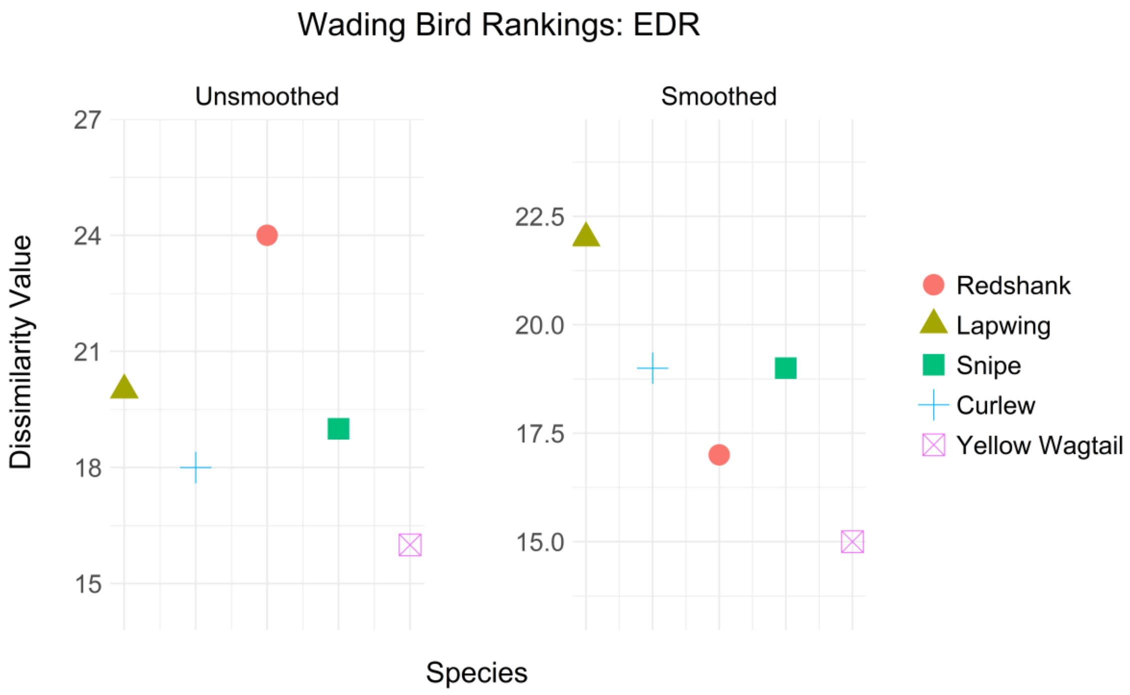

Fig. S56. Dissimilarity values for trend comparisons of five wading bird species using the Edit Distance on Real Sequences. Trends within reserves were compared with counterfactual trends from outside of reserves. Values on the left were from comparisons of the unsmoothed trends, while values on the right were calculated after applying LOESS smoothing with a span setting of 0.75. The dataset is from a study of conservation impact of wet grassland reserves on breeding birds in the UK (Jellesmark et al., 2021).

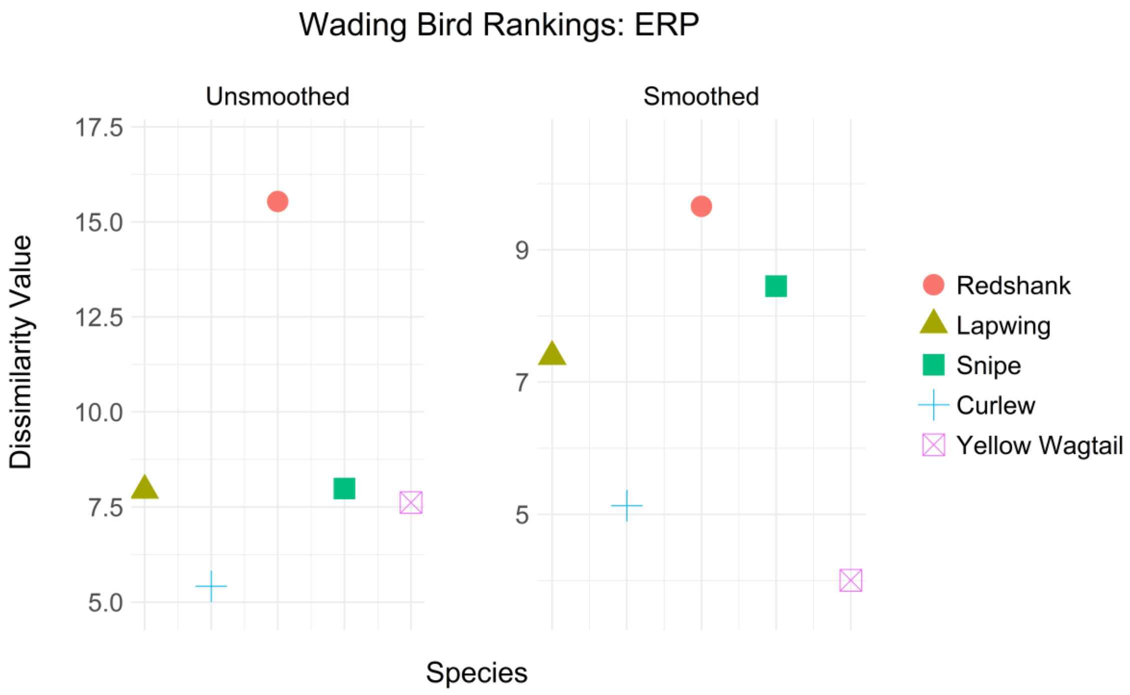

Fig. S57. Dissimilarity values for trend comparisons of five wading bird species using the Edit Distance with Real Penalty. Trends within reserves were compared with counterfactual trends from outside of reserves. Values on the left were from comparisons of the unsmoothed trends, while values on the right were calculated after applying LOESS smoothing with a span setting of 0.75. The dataset is from a study of conservation impact of wet grassland reserves on breeding birds in the UK (Jellesmark et al., 2021).

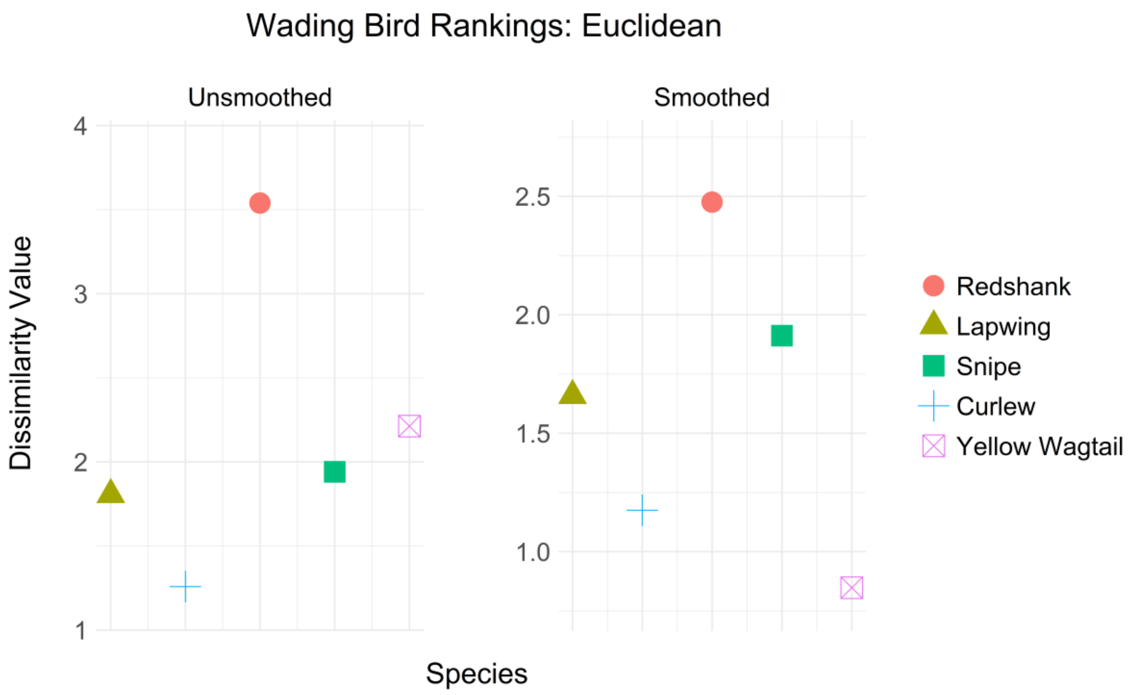

Fig. S58. Dissimilarity values for trend comparisons of five wading bird species using the Euclidean Distance. Trends within reserves were compared with counterfactual trends from outside of reserves. Values on the left were from comparisons of the unsmoothed trends, while values on the right were calculated after applying LOESS smoothing with a span setting of 0.75. The dataset is from a study of conservation impact of wet grassland reserves on breeding birds in the UK (Jellesmark et al., 2021).

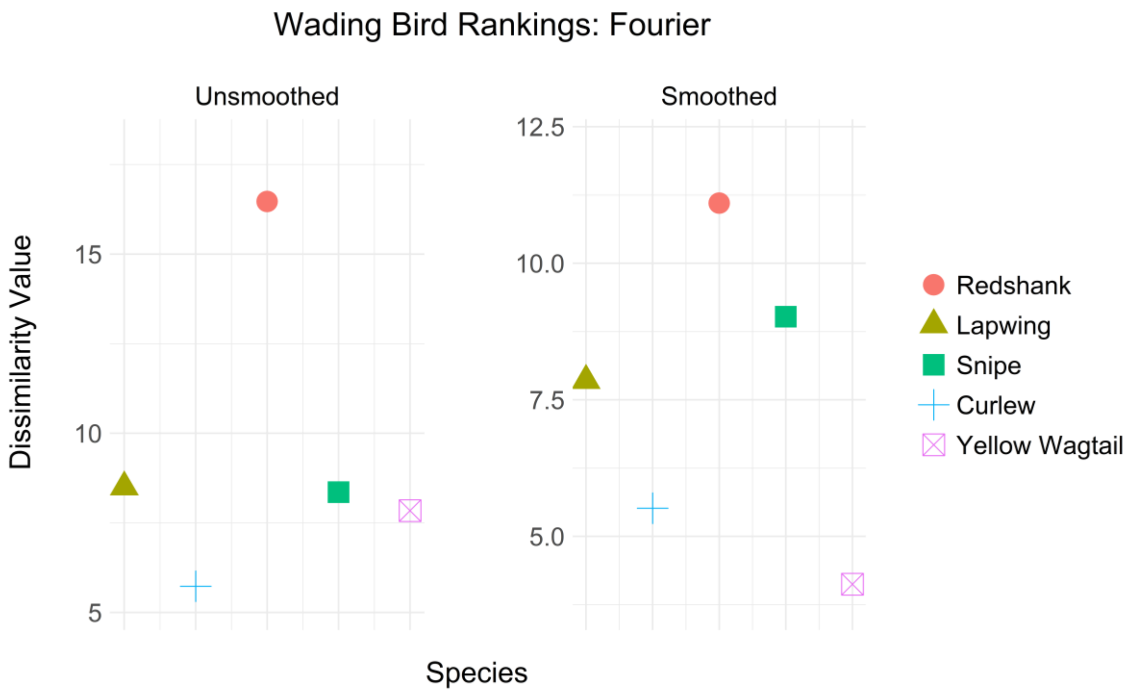

Fig. S59. Dissimilarity values for trend comparisons of five wading bird species using the Fourier Coefficient-Based Distance. Trends within reserves were compared with counterfactual trends from outside of reserves. Values on the left were from comparisons of the unsmoothed trends, while values on the right were calculated after applying LOESS smoothing with a span setting of 0.75. The dataset is from a study of conservation impact of wet grassland reserves on breeding birds in the UK (Jellesmark et al., 2021).

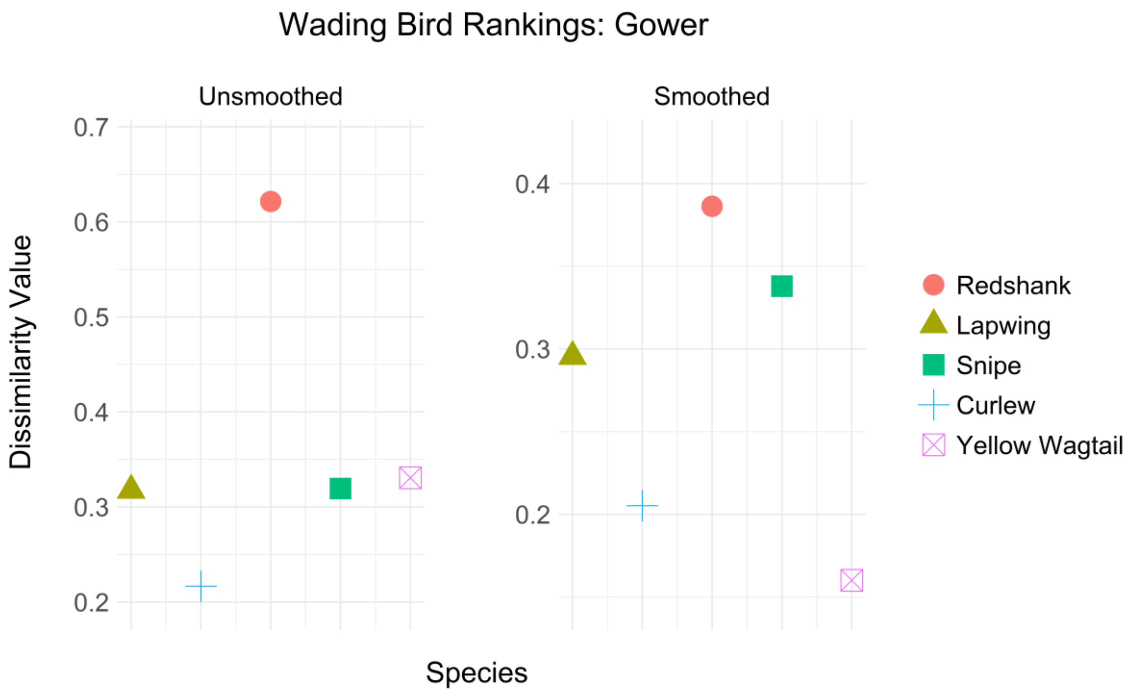

Fig. S60. Dissimilarity values for trend comparisons of five wading bird species using the Gower Distance. Trends within reserves were compared with counterfactual trends from outside of reserves. Values on the left were from comparisons of the unsmoothed trends, while values on the right were calculated after applying LOESS smoothing with a span setting of 0.75. The dataset is from a study of conservation impact of wet grassland reserves on breeding birds in the UK (Jellesmark et al., 2021).

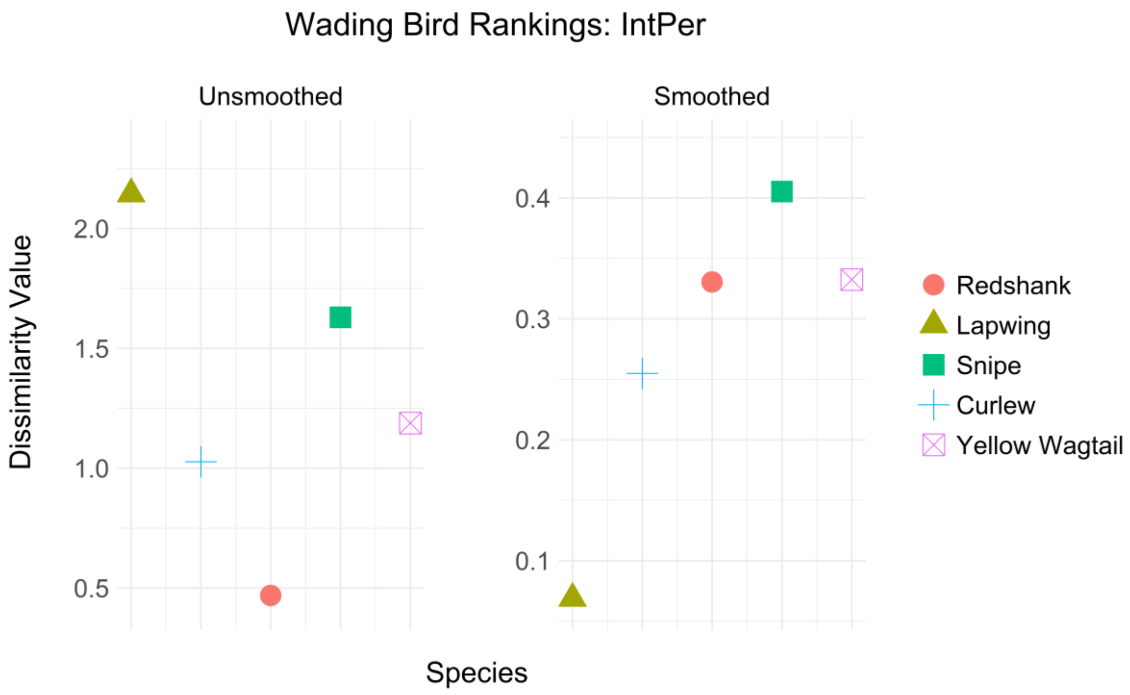

Fig. S61. Dissimilarity values for trend comparisons of five wading bird species using the Integrated Periodogram-Based Dissimilarity. Trends within reserves were compared with counterfactual trends from outside of reserves. Values on the left were from comparisons of the unsmoothed trends, while values on the right were calculated after applying LOESS smoothing with a span setting of 0.75. The dataset is from a study of conservation impact of wet grassland reserves on breeding birds in the UK (Jellesmark et al., 2021).

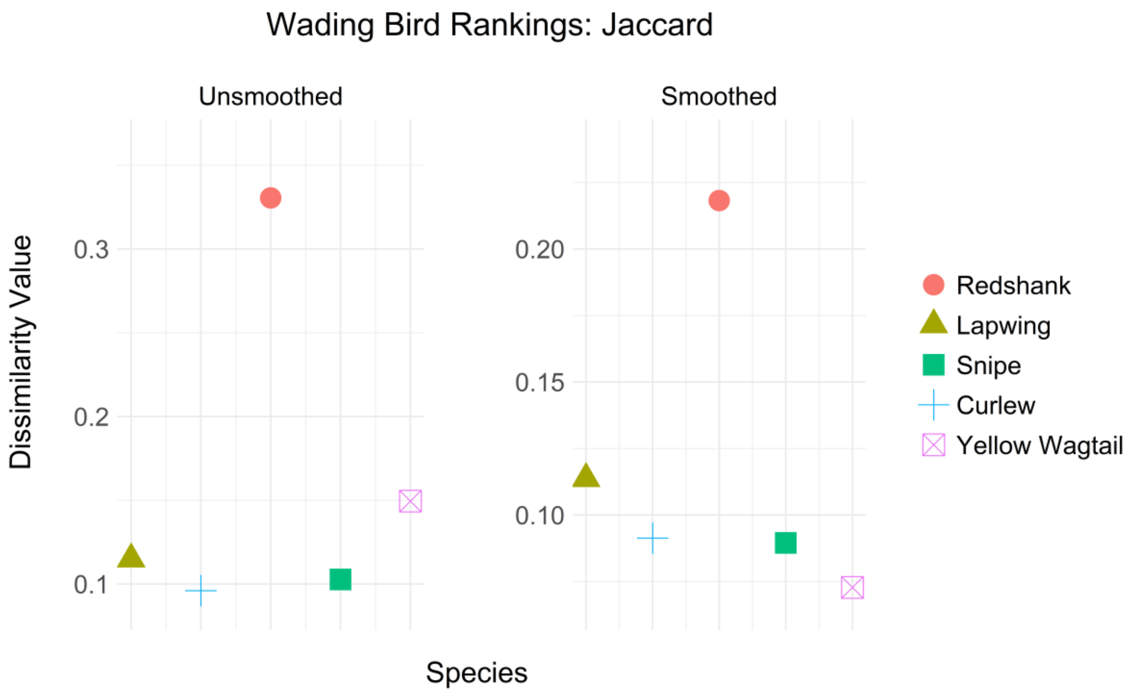

Fig. S62. Dissimilarity values for trend comparisons of five wading bird species using the Jaccard Distance. Trends within reserves were compared with counterfactual trends from outside of reserves. Values on the left were from comparisons of the unsmoothed trends, while values on the right were calculated after applying LOESS smoothing with a span setting of 0.75. The dataset is from a study of conservation impact of wet grassland reserves on breeding birds in the UK (Jellesmark et al., 2021).

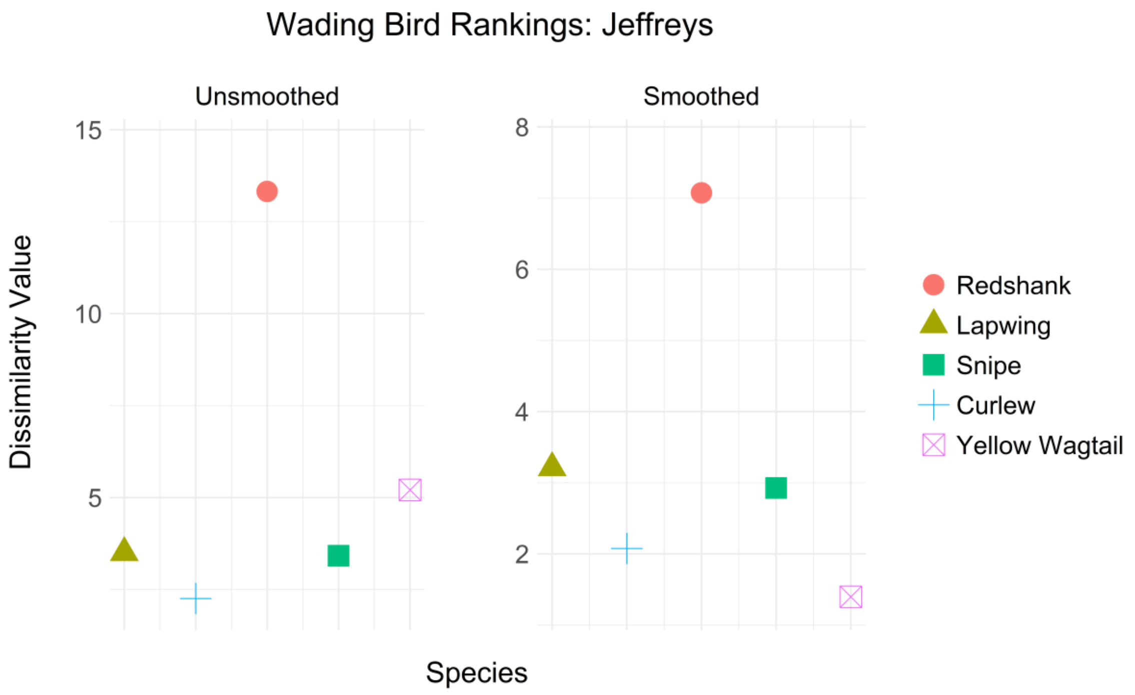

Fig. S63. Dissimilarity values for trend comparisons of five wading bird species using the Jeffreys Divergence. Trends within reserves were compared with counterfactual trends from outside of reserves. Values on the left were from comparisons of the unsmoothed trends, while values on the right were calculated after applying LOESS smoothing with a span setting of 0.75. The dataset is from a study of conservation impact of wet grassland reserves on breeding birds in the UK (Jellesmark et al., 2021).

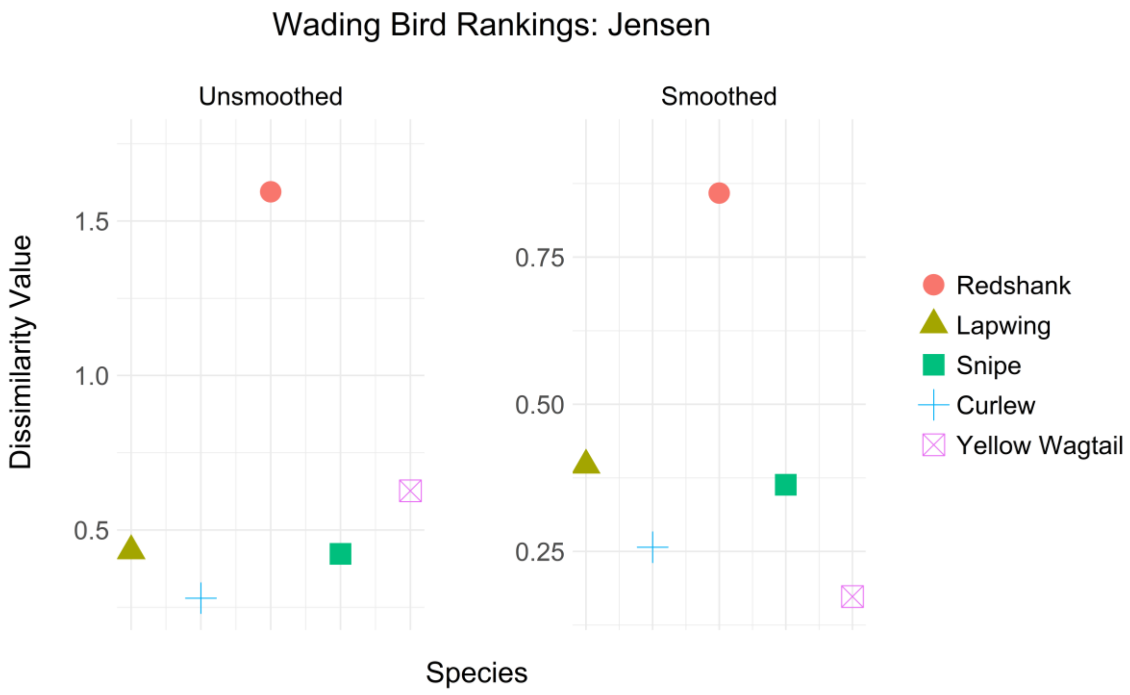

Fig. S64. Dissimilarity values for trend comparisons of five wading bird species using the Jensen Difference. Trends within reserves were compared with counterfactual trends from outside of reserves. Values on the left were from comparisons of the unsmoothed trends, while values on the right were calculated after applying LOESS smoothing with a span setting of 0.75. The dataset is from a study of conservation impact of wet grassland reserves on breeding birds in the UK (Jellesmark et al., 2021).

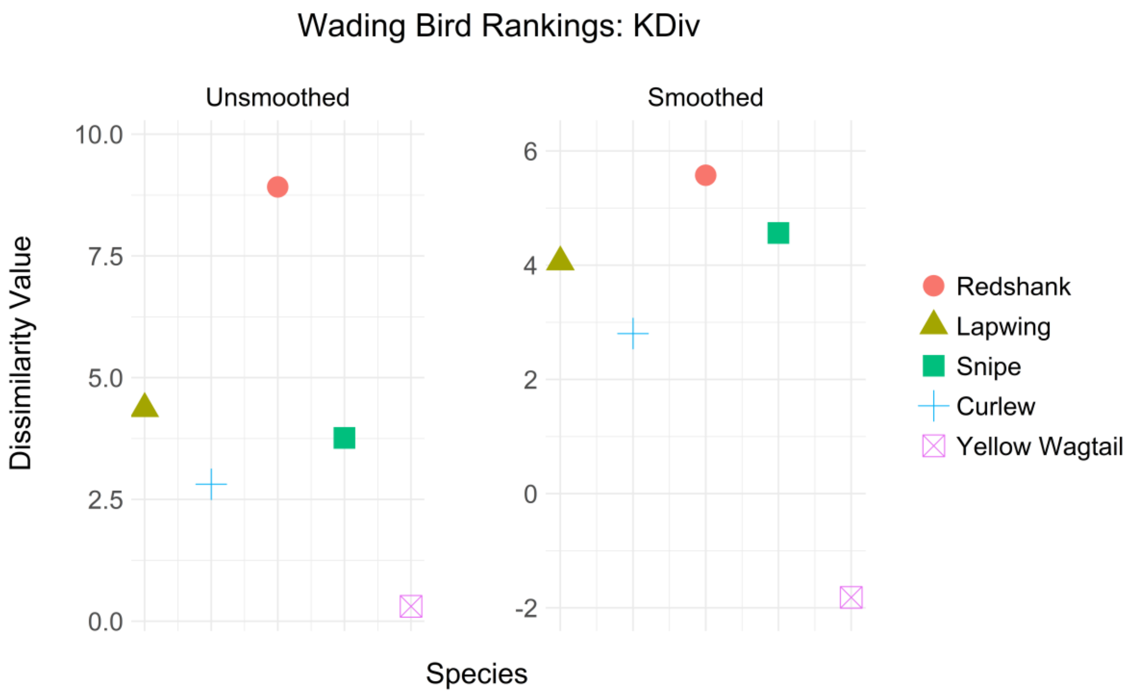

Fig. S65. Dissimilarity values for trend comparisons of five wading bird species using the K Divergence. Trends within reserves were compared with counterfactual trends from outside of reserves. Values on the left were from comparisons of the unsmoothed trends, while values on the right were calculated after applying LOESS smoothing with a span setting of 0.75. The dataset is from a study of conservation impact of wet grassland reserves on breeding birds in the UK (Jellesmark et al., 2021).

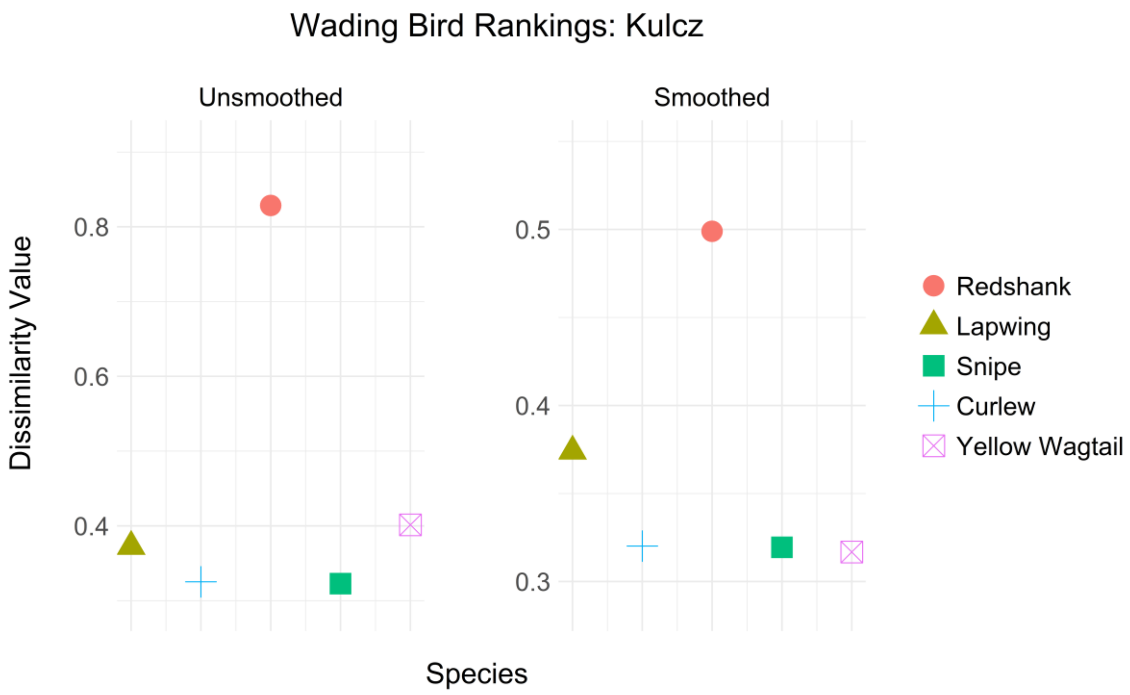

Fig. S66. Dissimilarity values for trend comparisons of five wading bird species using the Kulczynski Distance. Trends within reserves were compared with counterfactual trends from outside of reserves. Values on the left were from comparisons of the unsmoothed trends, while values on the right were calculated after applying LOESS smoothing with a span setting of 0.75. The dataset is from a study of conservation impact of wet grassland reserves on breeding birds in the UK (Jellesmark et al., 2021).

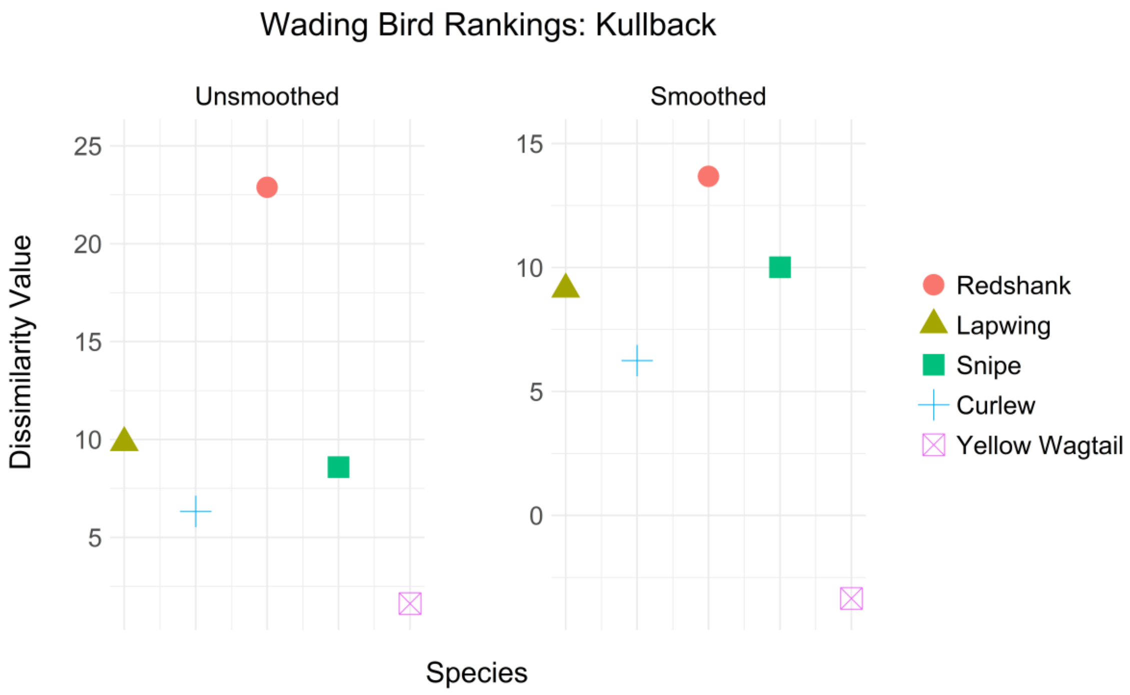

Fig. S67. Dissimilarity values for trend comparisons of five wading bird species using the Kullback-Leibler Divergence. Trends within reserves were compared with counterfactual trends from outside of reserves. Values on the left were from comparisons of the unsmoothed trends, while values on the right were calculated after applying LOESS smoothing with a span setting of 0.75. The dataset is from a study of conservation impact of wet grassland reserves on breeding birds in the UK (Jellesmark et al., 2021).

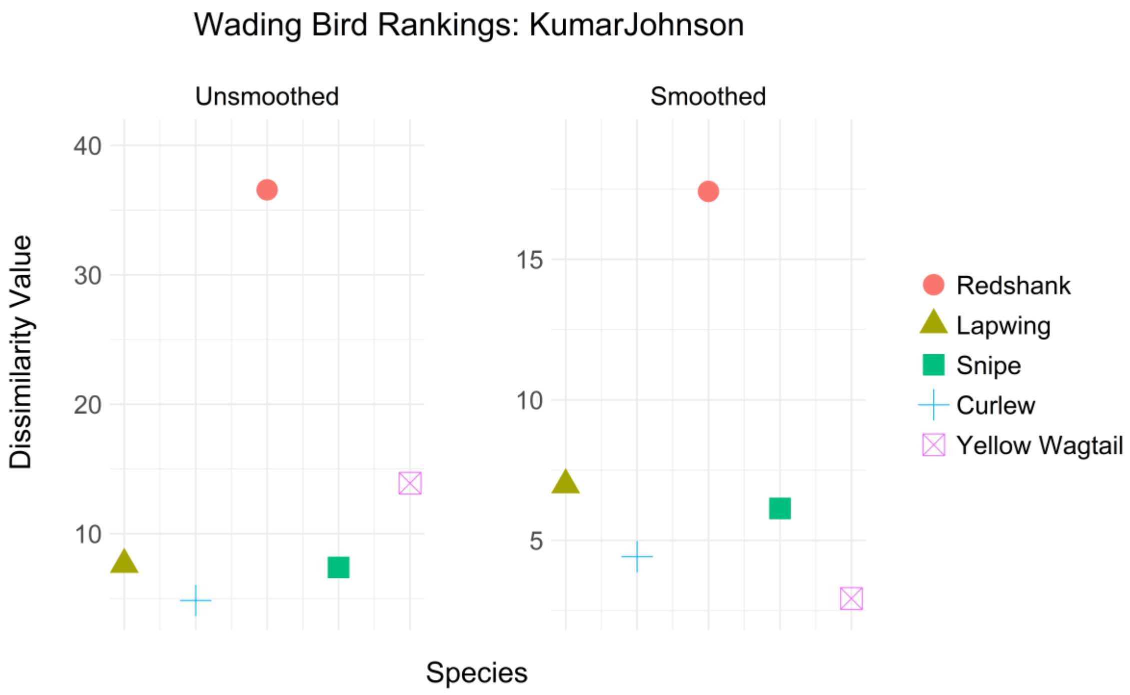

Fig. S68. Dissimilarity values for trend comparisons of five wading bird species using the Kumar-Johnson Distance. Trends within reserves were compared with counterfactual trends from outside of reserves. Values on the left were from comparisons of the unsmoothed trends, while values on the right were calculated after applying LOESS smoothing with a span setting of 0.75. The dataset is from a study of conservation impact of wet grassland reserves on breeding birds in the UK (Jellesmark et al., 2021).

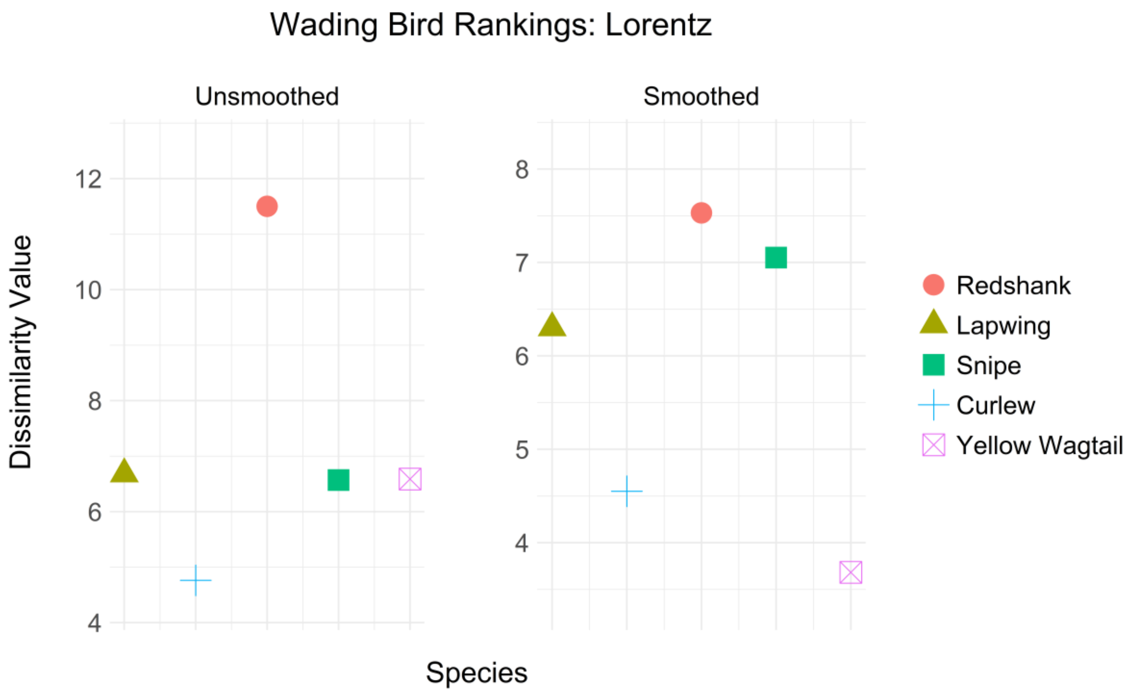

Fig. S69. Dissimilarity values for trend comparisons of five wading bird species using the Lorentzian Distance. Trends within reserves were compared with counterfactual trends from outside of reserves. Values on the left were from comparisons of the unsmoothed trends, while values on the right were calculated after applying LOESS smoothing with a span setting of 0.75. The dataset is from a study of conservation impact of wet grassland reserves on breeding birds in the UK (Jellesmark et al., 2021).

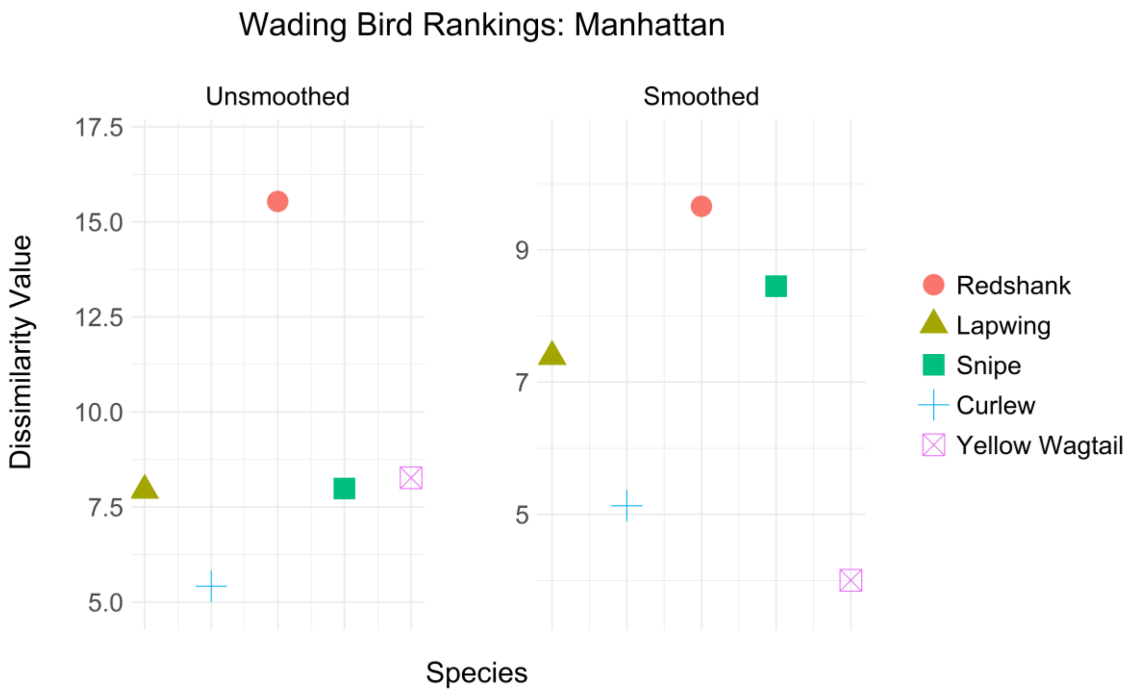

Fig. S70. Dissimilarity values for trend comparisons of five wading bird species using the Manhattan Distance. Trends within reserves were compared with counterfactual trends from outside of reserves. Values on the left were from comparisons of the unsmoothed trends, while values on the right were calculated after applying LOESS smoothing with a span setting of 0.75. The dataset is from a study of conservation impact of wet grassland reserves on breeding birds in the UK (Jellesmark et al., 2021).

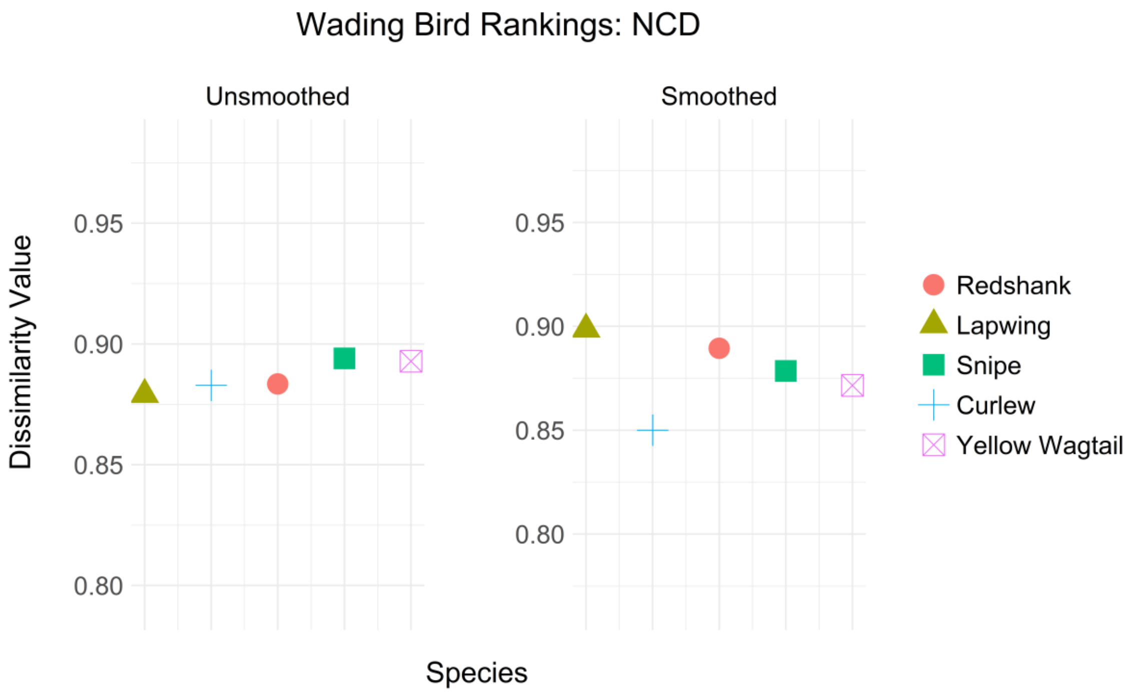

Fig. S71. Dissimilarity values for trend comparisons of five wading bird species using the Normalized Compression Distance. Trends within reserves were compared with counterfactual trends from outside of reserves. Values on the left were from comparisons of the unsmoothed trends, while values on the right were calculated after applying LOESS smoothing with a span setting of 0.75. The dataset is from a study of conservation impact of wet grassland reserves on breeding birds in the UK (Jellesmark et al., 2021).

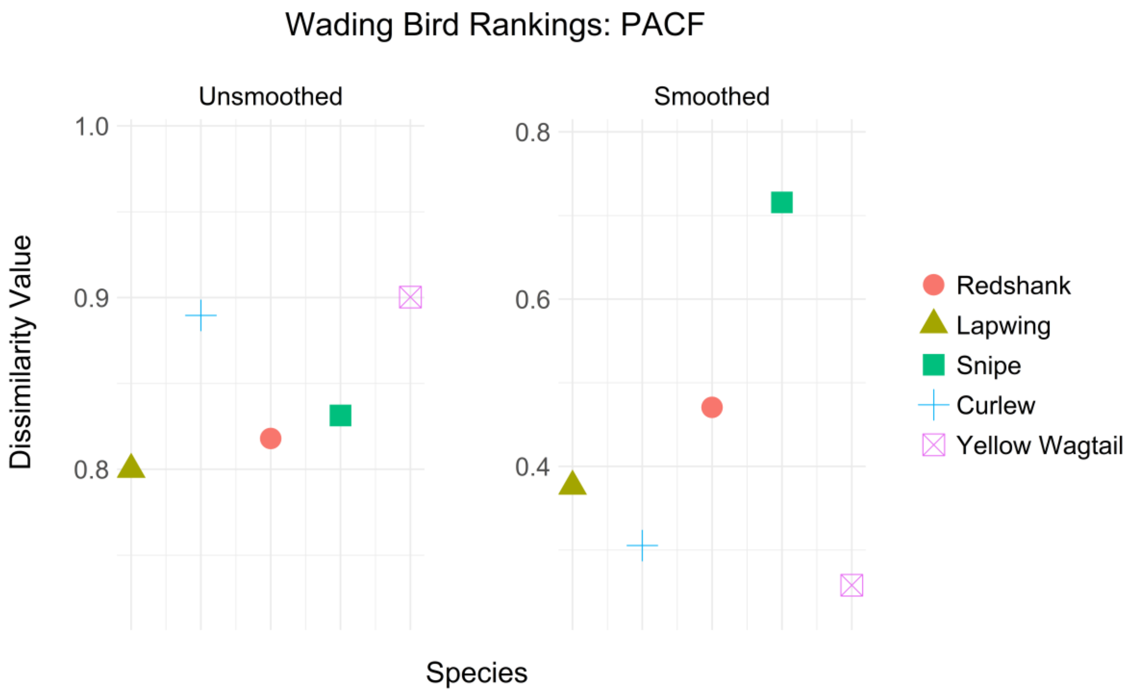

Fig. S72. Dissimilarity values for trend comparisons of five wading bird species using the Partial Autocorrelation-Based Dissimilarity. Trends within reserves were compared with counterfactual trends from outside of reserves. Values on the left were from comparisons of the unsmoothed trends, while values on the right were calculated after applying LOESS smoothing with a span setting of 0.75. The dataset is from a study of conservation impact of wet grassland reserves on breeding birds in the UK (Jellesmark et al., 2021).

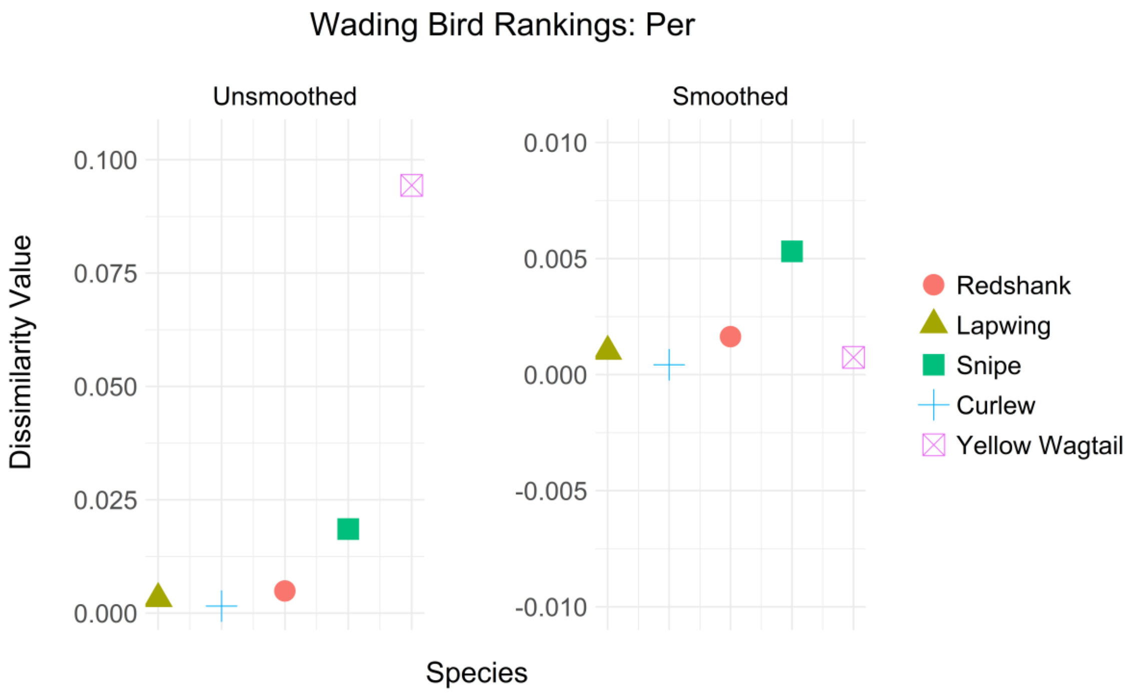

Fig. S73. Dissimilarity values for trend comparisons of five wading bird species using the Periodogram-Based Dissimilarity. Trends within reserves were compared with counterfactual trends from outside of reserves. Values on the left were from comparisons of the unsmoothed trends, while values on the right were calculated after applying LOESS smoothing with a span setting of 0.75. The dataset is from a study of conservation impact of wet grassland reserves on breeding birds in the UK (Jellesmark et al., 2021).

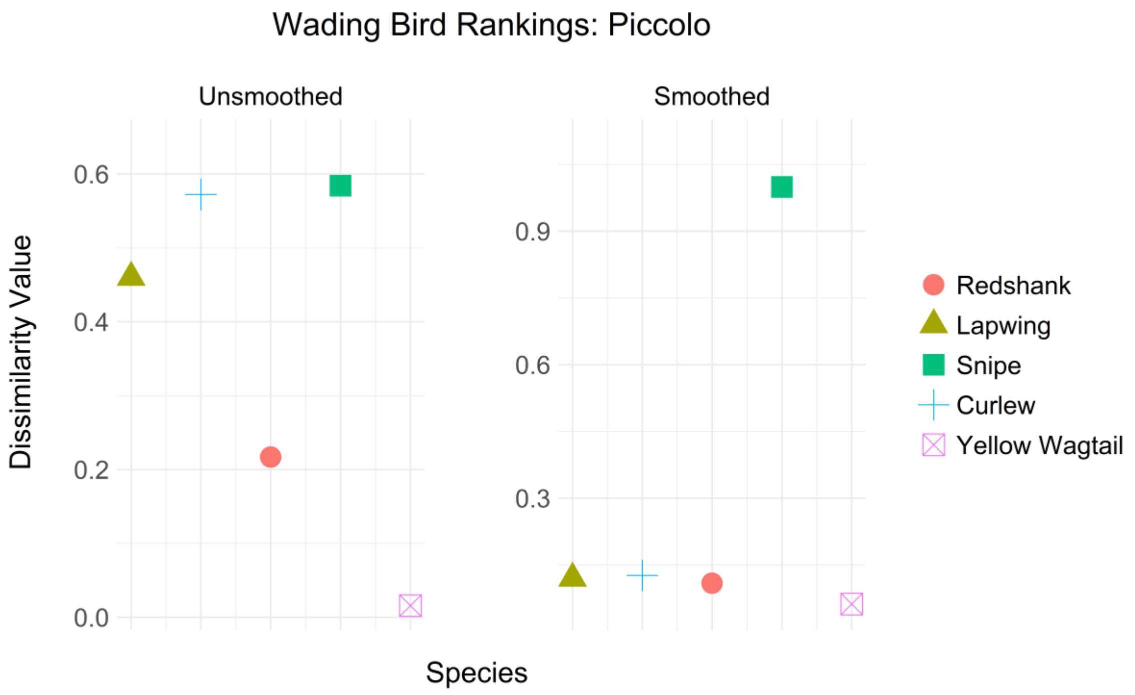

Fig. S74. Dissimilarity values for trend comparisons of five wading bird species using the Piccolo Distance. Trends within reserves were compared with counterfactual trends from outside of reserves. Values on the left were from comparisons of the unsmoothed trends, while values on the right were calculated after applying LOESS smoothing with a span setting of 0.75. The dataset is from a study of conservation impact of wet grassland reserves on breeding birds in the UK (Jellesmark et al., 2021).

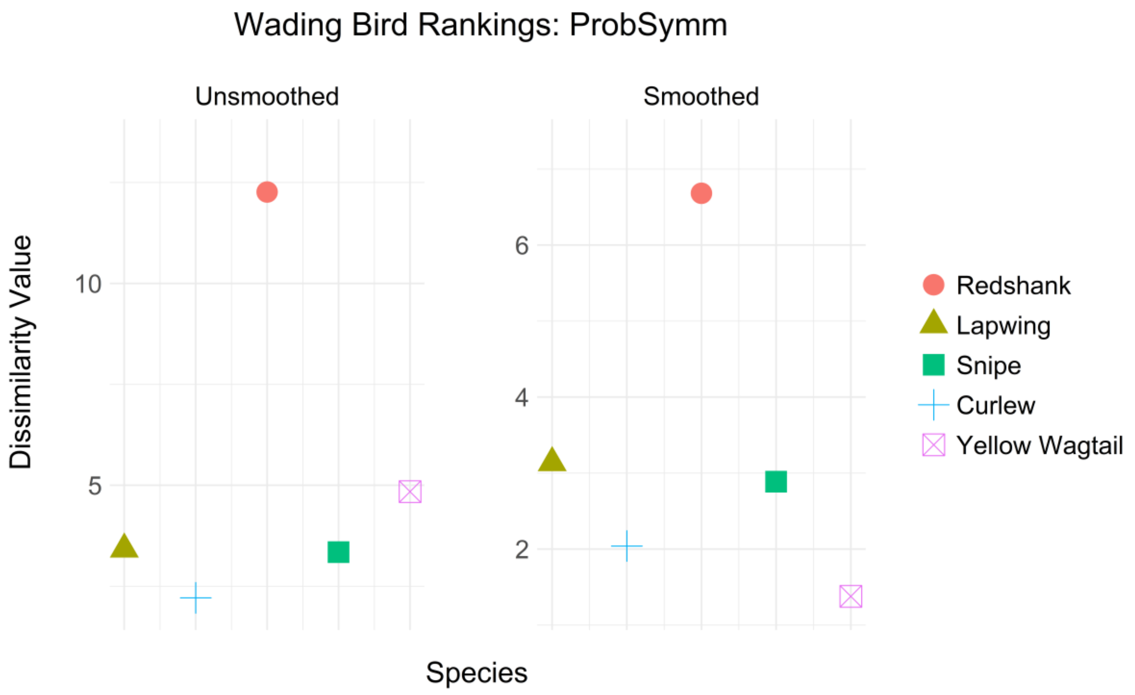

Fig. S75. Dissimilarity values for trend comparisons of five wading bird species using the Probabilistic Symmetric Chi-Squared Distance. Trends within reserves were compared with counterfactual trends from outside of reserves. Values on the left were from comparisons of the unsmoothed trends, while values on the right were calculated after applying LOESS smoothing with a span setting of 0.75. The dataset is from a study of conservation impact of wet grassland reserves on breeding birds in the UK (Jellesmark et al., 2021).

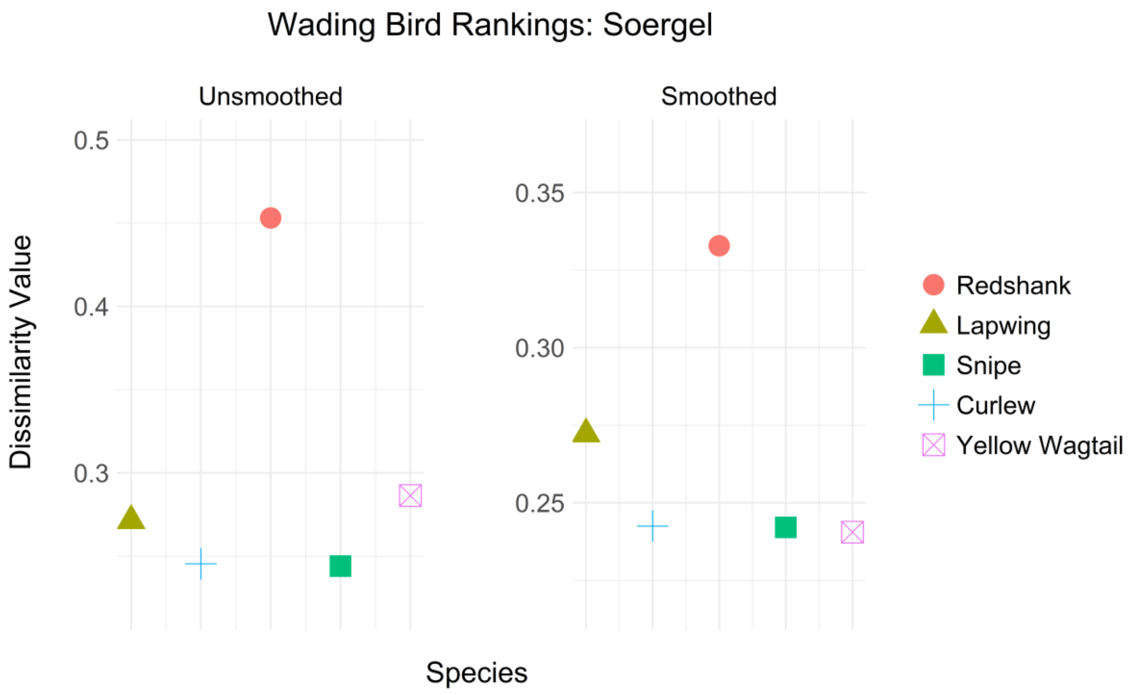

Fig. S76. Dissimilarity values for trend comparisons of five wading bird species using the Soergel Distance. Trends within reserves were compared with counterfactual trends from outside of reserves. Values on the left were from comparisons of the unsmoothed trends, while values on the right were calculated after applying LOESS smoothing with a span setting of 0.75. The dataset is from a study of conservation impact of wet grassland reserves on breeding birds in the UK (Jellesmark et al., 2021).

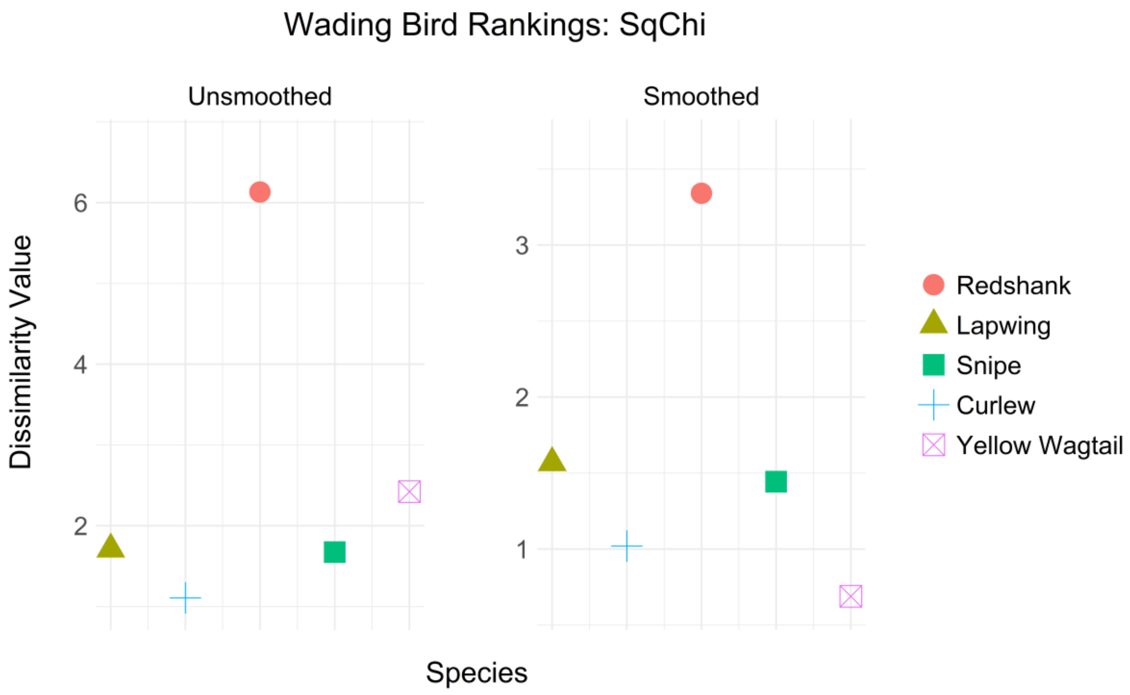

Fig. S77. Dissimilarity values for trend comparisons of five wading bird species using the Squared Chi-Squared Distance. Trends within reserves were compared with counterfactual trends from outside of reserves. Values on the left were from comparisons of the unsmoothed trends, while values on the right were calculated after applying LOESS smoothing with a span setting of 0.75. The dataset is from a study of conservation impact of wet grassland reserves on breeding birds in the UK (Jellesmark et al., 2021).

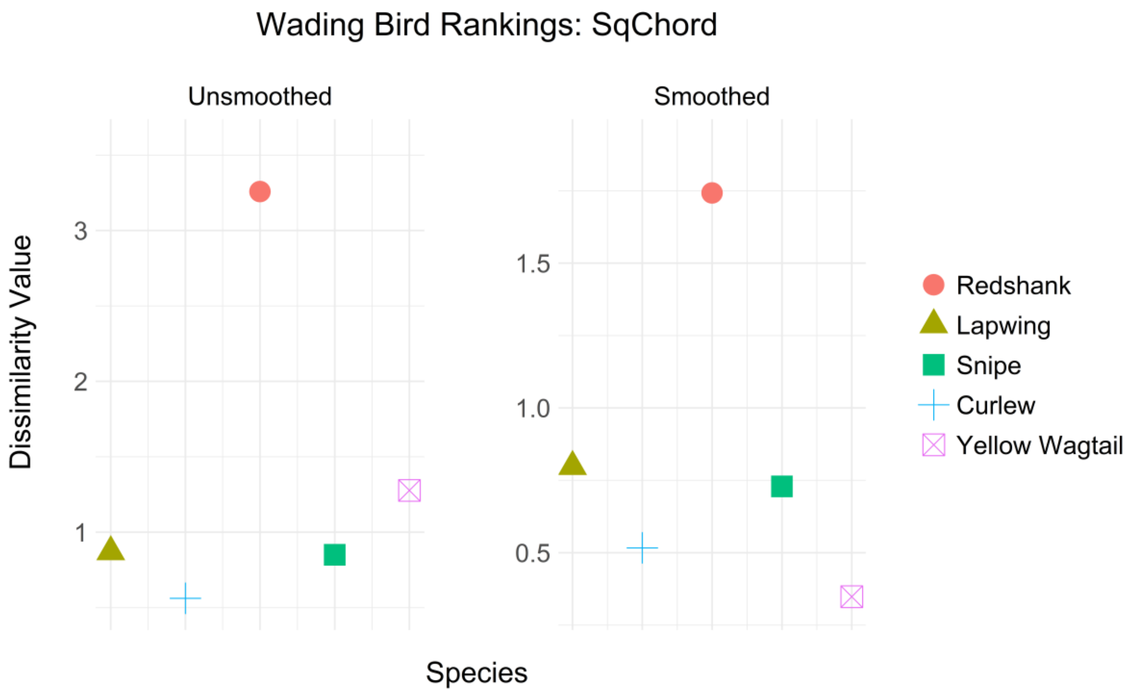

Fig. S78. Dissimilarity values for trend comparisons of five wading bird species using the Squared-Chord Distance. Trends within reserves were compared with counterfactual trends from outside of reserves. Values on the left were from comparisons of the unsmoothed trends, while values on the right were calculated after applying LOESS smoothing with a span setting of 0.75. The dataset is from a study of conservation impact of wet grassland reserves on breeding birds in the UK (Jellesmark et al., 2021).

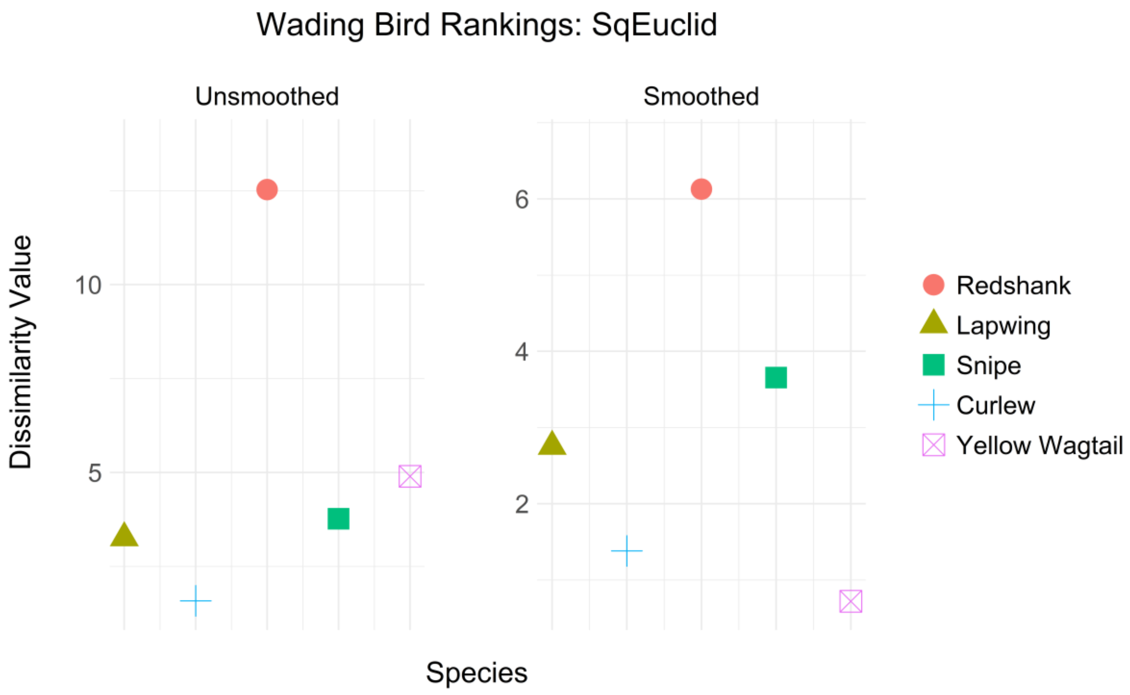

Fig. S79. Dissimilarity values for trend comparisons of five wading bird species using the Squared Euclidean Distance. Trends within reserves were compared with counterfactual trends from outside of reserves. Values on the left were from comparisons of the unsmoothed trends, while values on the right were calculated after applying LOESS smoothing with a span setting of 0.75. The dataset is from a study of conservation impact of wet grassland reserves on breeding birds in the UK (Jellesmark et al., 2021).

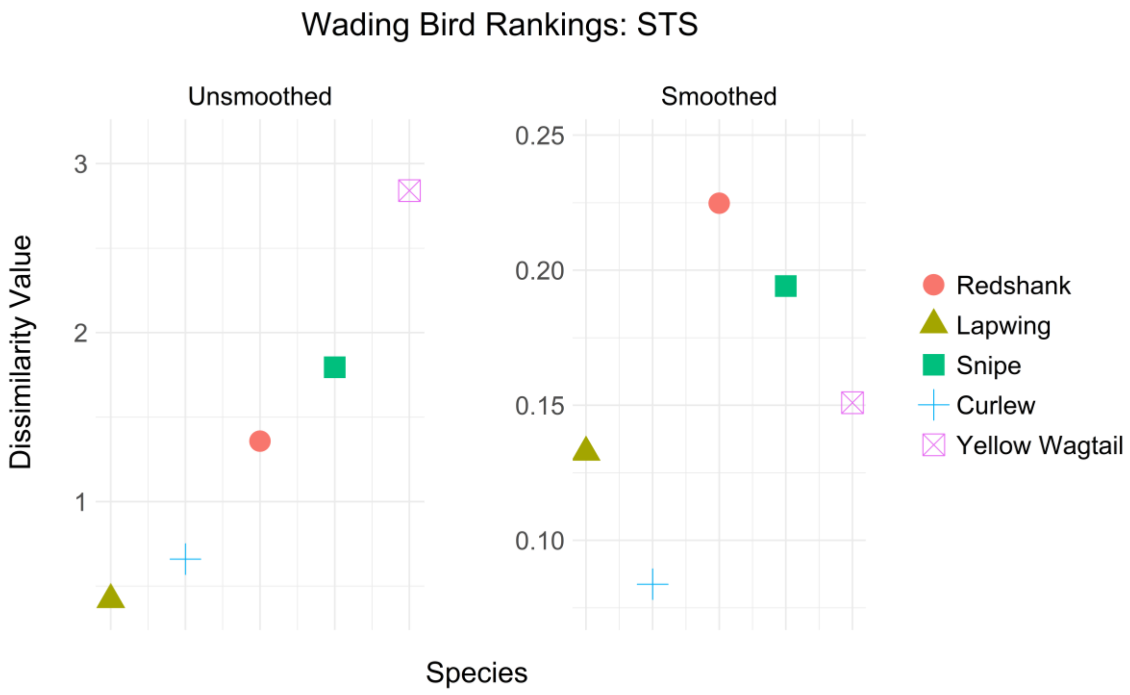

Fig. S80. Dissimilarity values for trend comparisons of five wading bird species using the Short Time Series Distance. Trends within reserves were compared with counterfactual trends from outside of reserves. Values on the left were from comparisons of the unsmoothed trends, while values on the right were calculated after applying LOESS smoothing with a span setting of 0.75. The dataset is from a study of conservation impact of wet grassland reserves on breeding birds in the UK (Jellesmark et al., 2021).

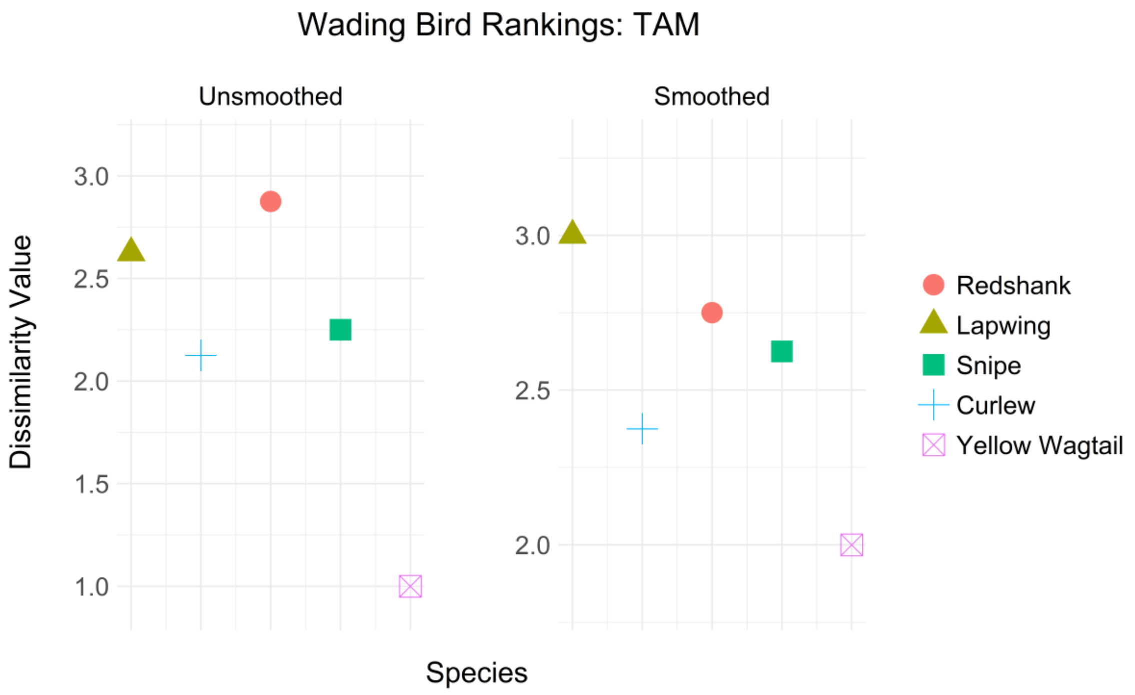

Fig. S81. Dissimilarity values for trend comparisons of five wading bird species using the Time Alignment Measurement Distance. Trends within reserves were compared with counterfactual trends from outside of reserves. Values on the left were from comparisons of the unsmoothed trends, while values on the right were calculated after applying LOESS smoothing with a span setting of 0.75. The dataset is from a study of conservation impact of wet grassland reserves on breeding birds in the UK (Jellesmark et al., 2021).

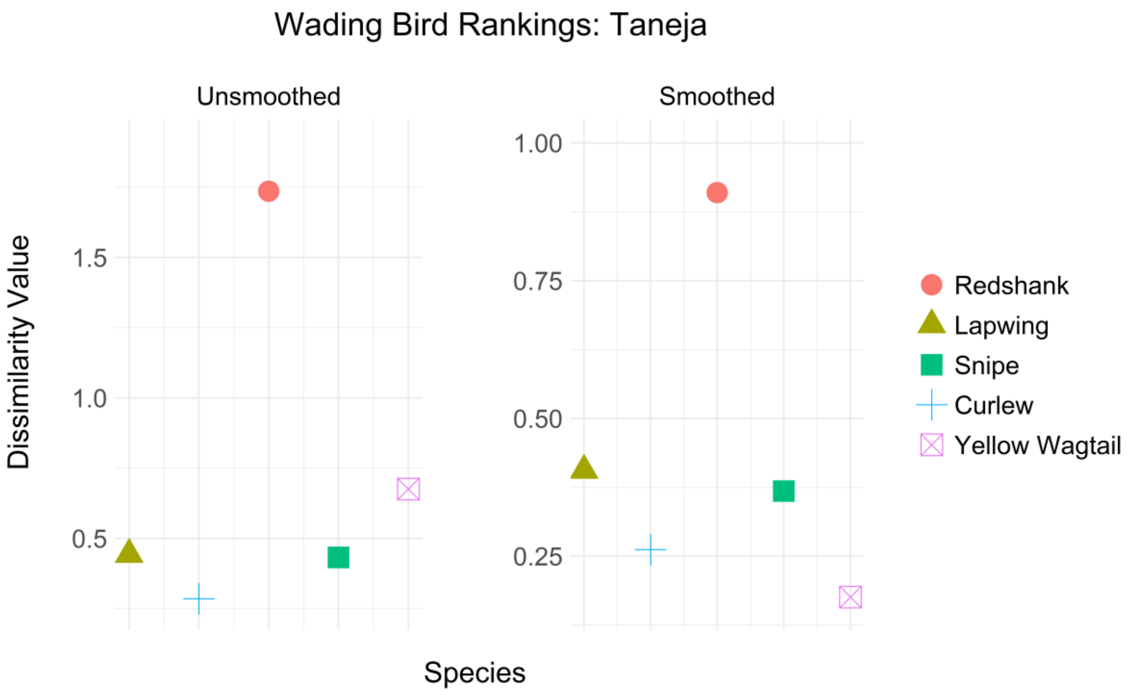

Fig. S82. Dissimilarity values for trend comparisons of five wading bird species using the Taneja Difference. Trends within reserves were compared with counterfactual trends from outside of reserves. Values on the left were from comparisons of the unsmoothed trends, while values on the right were calculated after applying LOESS smoothing with a span setting of 0.75. The dataset is from a study of conservation impact of wet grassland reserves on breeding birds in the UK (Jellesmark et al., 2021).

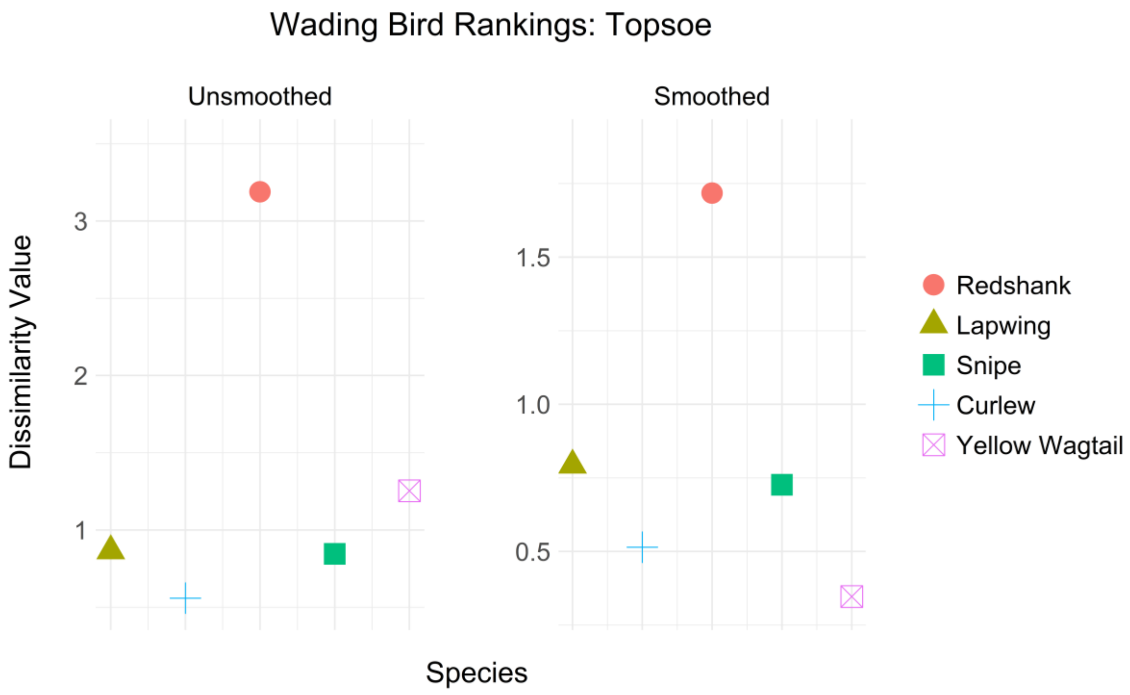

Fig. S83. Dissimilarity values for trend comparisons of five wading bird species using the Topsoe Distance. Trends within reserves were compared with counterfactual trends from outside of reserves. Values on the left were from comparisons of the unsmoothed trends, while values on the right were calculated after applying LOESS smoothing with a span setting of 0.75. The dataset is from a study of conservation impact of wet grassland reserves on breeding birds in the UK (Jellesmark et al., 2021).

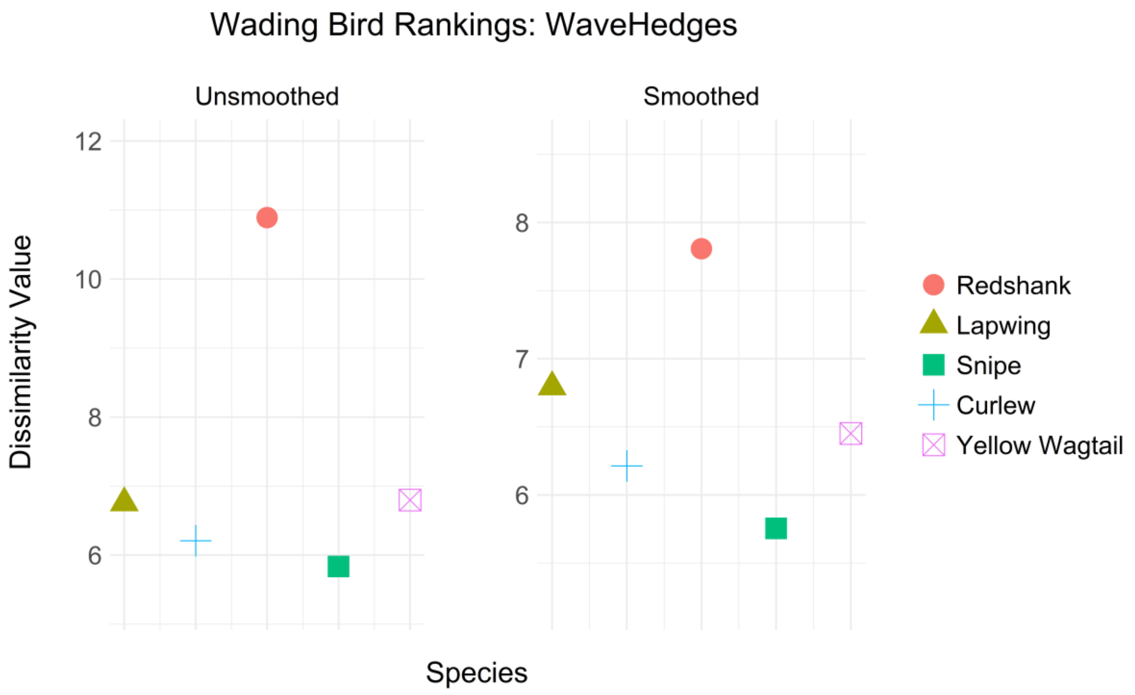

Fig. S84. Dissimilarity values for trend comparisons of five wading bird species using the Wave-Hedges Distance. Trends within reserves were compared with counterfactual trends from outside of reserves. Values on the left were from comparisons of the unsmoothed trends, while values on the right were calculated after applying LOESS smoothing with a span setting of 0.75. The dataset is from a study of conservation impact of wet grassland reserves on breeding birds in the UK (Jellesmark et al., 2021).
